# RNA-sequencing-based comparative analysis of human hepatic progenitor cells and their niche from alcoholic steatohepatitis livers

**DOI:** 10.1038/cddis.2017.543

**Published:** 2017-11-02

**Authors:** An Ceulemans, Stefaan Verhulst, Matthias Van Haele, Olivier Govaere, Juan-Jose Ventura, Leo A van Grunsven, Tania Roskams

**Affiliations:** 1 Department of Imaging and Pathology, Translational Cell and Tissue Research, KU Leuven and University Hospitals, Leuven, Belgium; 2 Liver Cell Biology Lab, Department of Basic Biomedical Sciences, Vrije Universiteit Brussel, Brussels, Belgium; 3 Laboratory for Translational Cell and Tissue Research, Department of Imaging and Pathology, KU Leuven – University of Leuven, Leuven, Belgium; 4 Institute of Cellular Medicine, Newcastle University, Newcastle upon Tyne, UK

## Abstract

Hepatic progenitor cells (HPCs) are small cells with a relative large oval nucleus and a scanty cytoplasm situated in the canals of Hering that express markers of (immature) hepatocytes and cholangiocytes. HPCs are present in large numbers in alcoholic steatohepatitis (ASH), one of the leading causes of chronic liver disease. To date, the mechanisms responsible for proliferation and differentiation of human HPCs are still poorly understood and the role of HPCs in ASH development is unknown. In this study, we aimed to characterise human HPCs and their interactions with other cells through comparison, on both protein and RNA level, of HPC-enriched cell populations from adult human liver tissue using different isolation methods. Fresh human liver tissue was collected from ASH explant livers and HPC-enriched cell populations were obtained via four different isolation methods: side population (SP), epithelial cell adhesion molecule (EpCAM) and trophoblast antigen 2 (TROP-2) membrane marker isolation and laser capture microdissection. Gene expression profiles of fluorescent-activated cell-sorted HPCs, whole liver extracts and laser microdissected HPC niches were determined by RNA-sequencing. Immunohistochemical evaluation of the isolated populations indicated the enrichment of HPCs in the SP, EpCAM^+^ and TROP-2^+^ cell populations. Pathway analysis of the transcription profiles of human HPCs showed an enrichment and activation of known HPC pathways like Wnt/*β*-catenin, TWEAK and HGF. Integration of the HPC niche profile suggests autocrine signalling by HPCs (TNF*α*, PDGFB and VEGFA) as well as paracrine signalling from the surrounding niche cells including MIF and IGF-1. In addition, we identified IL-17 A signalling as a potentially novel pathway in HPC biology. In conclusion, we provide the first RNA-seq-based, comparative transcriptome analysis of isolated human HPCs from ASH patients and revealed active signalling between HPCs and their surrounding niche cells in ASH livers and suggest that HPCs can actively contribute to liver inflammation.

The adult human liver, as part of the gastrointestinal tract, has a high capacity to regenerate and to restore its liver mass after liver damage.^1^ Mild cell loss can be compensated by proliferation of the epithelial cell compartment, the hepatocytes (HCs) and the cholangiocytes, followed by proliferation of the mesenchymal and endothelial cells.^2^ However, when cell loss is too severe and/or the regenerative capacity of the HCs is impaired, which is the case in many chronic liver diseases, a third epithelial cell compartment becomes active: the hepatic progenitor cells (HPCs), which can differentiate either into HCs or cholangiocytes dependent on the most damaged cell compartment.^2^

The widely spread use of alcohol and alcohol abuse in the world makes alcoholic steatohepatitis (ASH) one of the leading causes of chronic liver disease and liver failure. The pathology of ASH ranges from fatty liver, septal fibrosis to cirrhotic liver and hepatocellular carcinoma. During disease progression there is increased damaging of HCs and HC growth arrest, impairing the hepatocellular regeneration of the liver.^3, 4^ Besides a high activation and proliferation of HPCs, the ASH liver is also characterised with an inflammatory response and rapid progression of fibrosis.^5^ Little is known about the HPC identity and the mechanisms controlling HPC activation during ASH development.

HPCs are small cells with a relative large oval nucleus and a scanty cytoplasm.^6^ They are situated in the canals of Hering, the terminal branches of the biliary tree, in the portal tracts.^7^ Phenotypically, HPCs express markers of (immature) HCs (e.g. *α*-fetoprotein) and markers of cholangiocytes (e.g. cytokeratin K7 and K19). A specific HPC marker has yet to be found, which is one of the technical hurdles slowing down human HPC research as it is hindering the development of good isolation methods.^6^ Several different approaches have been reported to obtain HPC-enriched cell populations from animal livers and human liver tumour tissue. The side population (SP) technique, for instance, isolates an HPC-enriched population based on the efflux of the fluorescent dye Hoechst-33342, due to the high expression of ABC (ATP-binding cassette) transporters.^8, 9^ Another method to sort/isolate HPCs is based on the epithelial membrane marker EpCAM (epithelial cell adhesion molecule).^10^ Still, EpCAM and other cell markers are not uniquely expressed by HPCs and therefore the obtained cell populations also contain other cell types like cholangiocytes and HPC-derived intermediate HCs that express EpCAM as well.

Trophoblast antigen 2 (TROP-2) is a relatively new epithelial marker and is specifically expressed by activated progenitor cells in mouse models of liver disease.^11^ TROP-2 is an epithelial membrane protein also known as tumour-associated calcium signal transducer 2 (TACSTD2). It is the only other *tacstd* gene family member of TACSTD1, also known as EpCAM.^12^ During liver injury in murine models, HPCs (aka oval cells) start to express TROP-2 and can be used to isolate oval cells from animal models of liver disease.^11^ TROP-2 expression in human HPCs has been described within our group.^13, 14^ To isolate human HPCs, TROP-2 expression and isolation potential has not been tested yet in alcoholic liver.

In this study, we explored three different HPC isolation approaches and determined the transcription profiles of the obtained SP, EpCAM^+^ and TROP-2^+^ cell populations of human adult ASH explant liver tissue by RNA-sequencing. To gain further insight into the mechanisms involved in HPC activation, we isolated the HPCs and their niche via laser capture microdissection (LMD) and compared their transcriptional profile with HPCs.

## Results

### TROP-2 expression in healthy and diseased human liver

**Figure 1:**
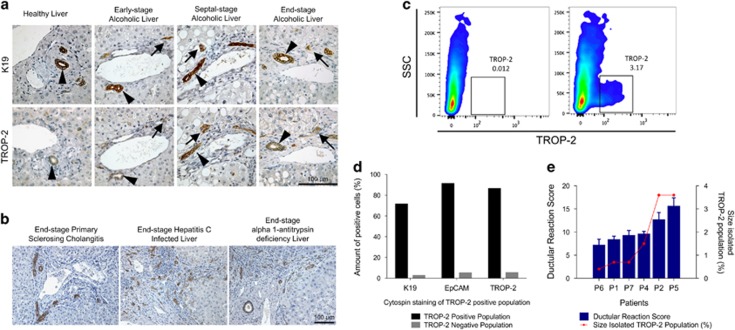
Evaluation of TROP-2 expression in human liver and isolation of TROP-2^+^ cells. (**a**) Immunohistochemical evaluation of TROP-2 in healthy, early, septal and end-stage alcoholic liver, compared with the expression of K19 (arrows = TROP-2+ ductular reaction; arrowheads = TROP-2+ cholangiocytes). (**b**) TROP-2 expression in other end-stage liver diseases: primary sclerosing cholangitis, hepatitis C and *α*1-antitrypsin deficiency. (**c**) FACS analysis of TROP-2^+^ cell population. (**d**) Immunohistochemical analysis of cytospins of TROP-2^+^ population. (**e**) Correlation between the ductular reaction score and percentage of the isolated population, shown for TROP-2^+^ populations

**Figure 2:**
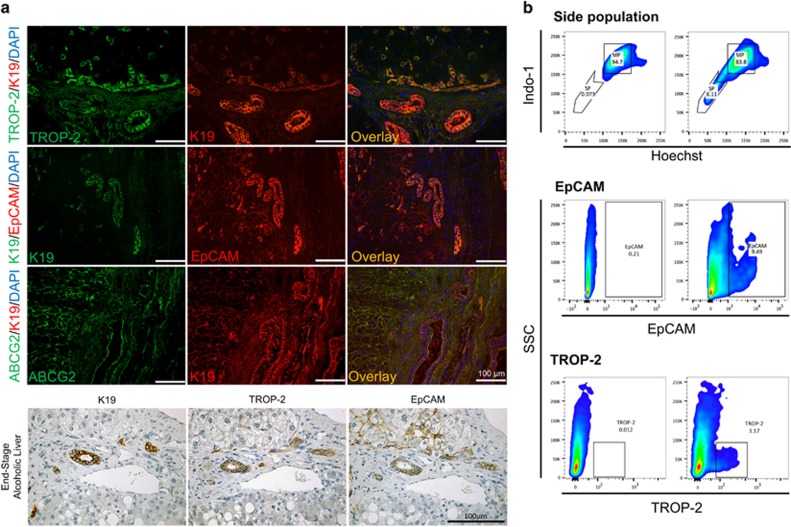
Isolation and evaluation of SP, EpCAM^+^ and TROP-2^+^ population. (**a**) Immunofluorescent double staining of human ASH liver: TROP-2/K19, K19/EpCAM and ABCG2/K19. Immunohistochemical evaluation of K19, TROP-2 and EpCAM expression in ASH liver. (**b**) FACS profiles of SP, EpCAM^+^ and TROP-2^+^ populations

TROP-2 has been used previously to isolate HPC populations from murine livers, without contamination of cholangiocytes or intermediate HCs.^15^ To evaluate the suitability of TROP-2 for the isolation of human HPC populations, the expression of this membrane protein was validated with immunohistochemistry in healthy and diseased human livers. The expression of TROP-2 was compared with K19 expression, a known and validated HPC marker,^13^ in different stages of alcoholic liver disease. In healthy human liver, low expression of TROP-2 was detected in cholangiocytes, while K19 was strongly expressed in these cells (Figure 1a). As the disease proceeded, cholangiocytes and the activated HPCs started to express more TROP-2 with the highest expression of TROP-2 in the cholangiocytes and ductular reaction of the end-stage diseased liver. Comparison of the expression of TROP-2 and K19 indicated that both proteins are expressed by the same cells, namely cholangiocytes, HPCs and some intermediate HCs. This colocalisation was later confirmed by fluorescent immunohistochemical double staining of K19/TROP-2 (Figure 2a). The same expression pattern could be detected in end-stage livers with other aetiologies like primary sclerosing cholangitis, hepatitis C viral infection and *α*1-antitrypsin deficiency (Figure 1b). Fluorescent-activated cell sorting (FACS) analysis showed a clear TROP-2^+^ population when a fluorophore-conjugated TROP-2 antibody and ASH liver cell suspension were used (Figure 1c). Analysis of the TROP-2^+^ cell population via immunohistochemical validation of cytospins demonstrated that this population consisted of 86% TROP-2^+^, 91% EpCAM^+^ and 71% K19^+^ cells. (Figure 1d). The degree of ductular reaction, or the activation of HPCs, in the different ASH samples, could be correlated with the percentage of TROP-2^+^ cells isolated by FACS (Figure 1e); the more activated HPCs, the higher the ductular reaction score, which resulted in higher amount of sorted TROP-2^+^ cells. Taken together, these results demonstrate that human HPCs express TROP-2 and that it can be used for the isolation of HPCs from human livers.

### Isolation of HPC-enriched populations from ASH livers based on SP, EpCAM and TROP-2 positivity

The two membrane markers EpCAM and TROP-2 are expressed in HPCs and cholangiocytes in human explant livers, demonstrated by coexpression with K19 on fluorescent immunohistochemical double staining of TROP-2/K19 and EpCAM/K19 (Figure 2a). TROP-2 was mainly located in ductular reactions and cholangiocytes, while EpCAM was also expressed in a large part of the intermediate HC population resulting in a bigger EpCAM^+^ population than a TROP-2^+^ population (Figure 2a). The SP method is a functional isolation method based on the efflux of Hoechst-33342 by ABC transporters, like ABCG2. This membrane transporter was expressed throughout the liver, but although ABCG2 and other ABC transporters were expressed in HCs, the expression was higher in HPCs (Figure 2a). To characterise human HPCs and identify underlying proliferation and differentiation mechanisms, single-cell suspensions of four different ASH livers were used to isolate TROP-2^+^, EpCAM^+^ or SP cells from each liver by FACS. Cells were sorted excluding debris, duplets, CD45^+^ cells (only in SP) and dead cells using forward scatter, side scatter, CD45 antibody and propidium iodide gating (Figure 2b). A schematic overview of the followed workflow can be found in Supplementary Figure 3. Immunohistochemical analysis of FACS-sorted HPCs showed that the isolated HPCs contained relatively low number of cells not expressing TROP-2, EpCAM or K19, indicating a successful isolation of human HPCs with all methods used (Supplementary Figure 4). Sorted cell populations were processed for RNA-sequencing to compare the transcriptome of the different isolated HPCs with each other and the total liver cell population (referred to as total liver).

### Transcriptome profiling of SP, EpCAM^+^ and TROP-2^+^ HPCs from ASH livers

**Figure 3:**
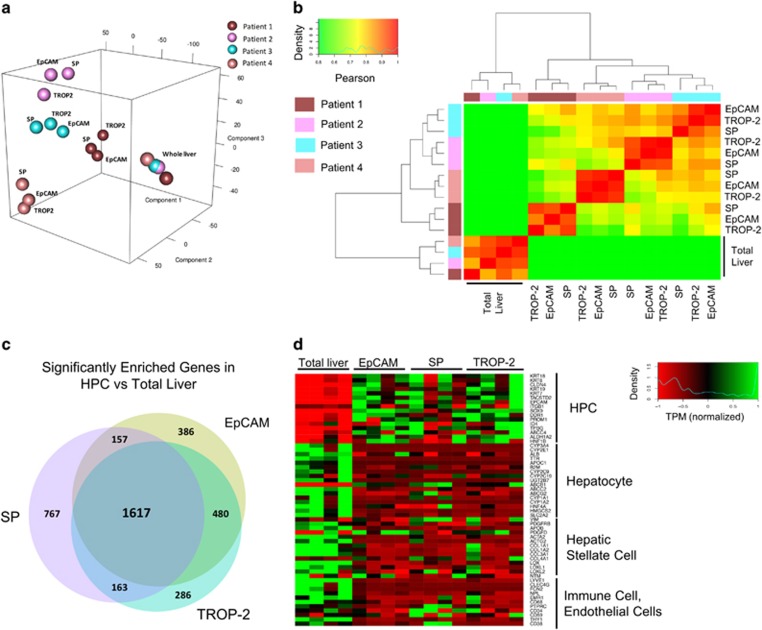
Evaluation of SP, EpCAM^+^ and TROP-2^+^ population. (**a**) PCA analysis of the different patients and isolation methods. (**b**) Clustering of the different isolated groups. (**c**) Overview of the amount of significant enriched genes in the different isolated groups *versus* total liver. (**d**) Marker clustering analysis of different cell types in total liver *versus* SP, EpCAM^+^ and TROP-2^+^ population

Principal component analysis (PCA) showed that the total liver samples were clustered together, completely separated from the HPC-isolated groups (Figures 3a and b). Surprisingly, the isolated groups were clustered based on patient variety, not on the isolation method. This result was also supported by Pearson's correlation on the same groups. Although there were still large similarities between the patients, one patient sample (patient 1) clearly diverged from the others, which was not observed in the total liver samples from all the corresponding patients (Figure 3a).

To identify a possible human HPC gene signature, the significantly enriched genes from all three isolated groups were compared with total liver (Figure 3c). A total of 1617 genes or 41.9% of the total number of enriched genes were common between the three HPC populations, indicating a high similarity between the HPC-enriched groups. The similarities between the EpCAM^+^ and TROP-2^+^ groups were the highest (a total of 2097 enriched genes in common or 67.9% of all enriched genes in EpCAM and TROP-2 groups).

Based on the PCA, Pearson's clustering and the amount of corresponding genes common in all groups, the three FACS isolation methods seemed to result in highly similar populations. A more detailed analysis of these isolated groups showed that HPC markers like *K19*, *SOX9* and *PROM1* were highly enriched in HPC groups, whereas HC markers such as *ALB*, *AFP* and *HNF4A* were clearly deprived. In the total liver group the opposite could be detected: a lower expression of HPC markers and a higher expression of HC markers. Analysis of other cell-type-specific genes did not indicate the presence of immune cells, stellate cells or endothelial cells in the HPC-enriched groups (Figure 3d). In support of our study, we performed qPCR validation of the FACS isolated groups and compared the fold expression with the RNA-sequencing. Comparison of qPCR and RNA-seq of the same samples gave the same expression pattern with an enrichment of HPC markers in SP, EpCAM^+^ and TROP-2^+^ populations and enrichment of HC, stellate cell and immune cell markers in total liver samples (Supplementary Figure 5).

**Figure 4:**
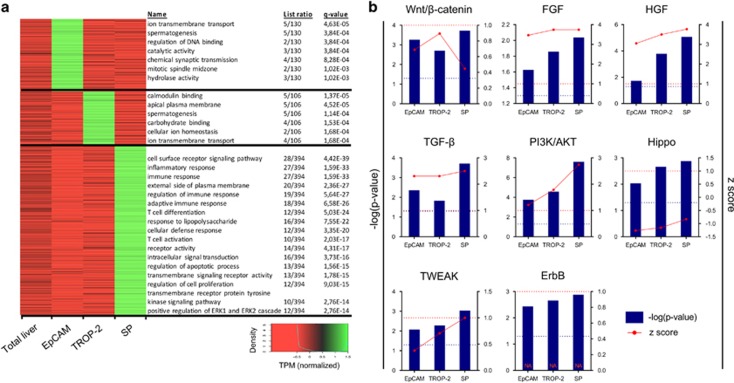
Pathway analysis of differently expressed genes of the FACS isolated HPC-enriched populations. (**a**) Differences in significant pathways in the different HPC-enriched groups and total liver. (**b**) Different significant (−log(*P*-value)) pathways linked with HPC activation/proliferation and their activation (*z*-score) in the different isolated groups *versus* total liver. Blue dotted line=significant *P*-level (0.05). Red dotted line=*z*-score limit of 1 (*z*-score>1 is activation, *z*-score<1 is inhibition of pathway)

Gene ontology (GO) analysis on the differential expressed genes showed variances in GO terms between the differently isolated populations (Figure 4a). Unique enriched genes for EpCAM (130 genes) or TROP-2 (106 genes) populations were characterised by metabolic GO terms that only contain up to five genes. In the SP group (394 genes), at least seven GO terms linked with immune response could be detected, next to cell metabolism-related pathways. These highly significant immune-related GOs could indicate the presence of immune cells within this HPC group, even though CD45^+^ cells were excluded during FACS isolation. Immunohistochemical analysis of cytospins indicated that 3% of the isolated cells were expressing CD45. The absence of these GOs in the TROP-2^+^ and EpCAM^+^ group in combination with only few, low significant GOs suggested a higher purity was achieved with these methods. The presence of some immune cells in SP could also explain the large set of enriched genes specific for SP (Figure 3c). Nevertheless, more than 1600 enriched genes (when compared with total liver) were shared with EpCAM and TROP-2, indicating that the SP contained mainly HPCs. Taken together, this transcriptome comparison indicated that different HPC-enriched populations were being isolated from the human livers using SP, TROP-2 or EpCAM selection, with EpCAM- and TROP-2-based isolations as the most selective methods (Figure 3d). Complete gene lists and extra information of the graphs in Figures 3 and 4a can be found in the supplementary excel file.

### Analysis of HPCs from ASH livers confirms involvement of established activation pathways

IPA analysis was performed on the differential expressed genes of the HPC-enriched populations *versus* total liver in search for pathways involved in human HPC activation. Previous work of Spee *et al.*^16^ already demonstrated that the presence of pathways first identified in animal models of liver disease can be confirmed in human livers, showing, for instance, the activation of Wnt signalling in proliferating HPCs isolated through LMD. Here, pathway analysis of RNA-seq data indicated a highly significant activation of the Wnt/*β*-catenin pathway in human HPCs isolated from ASH livers (Figure 4b). Several other pathways, described in animal models to steer HPC activation, were also significantly activated in all human HPC groups like HGF, FGF and TWEAK signalling^17, 18, 19, 20^ (Figure 4b). We also confirmed pathways that are less well documented so far for their involvement in HPC regulation like TGF-*β*, ErbB and the Hippo pathway.^21, 22^ These data suggested that these pathways, mainly described for mouse HPC biology, were present and activated in HPCs in human ASH livers. The IPA analysis resulted also in a list of significant pathways not previously associated with HPC biology such as PI3K/Akt and integrin-linked kinase signalling. A list of the common top upregulated pathways can be found in Supplementary Table 2.

### RNA-seq analysis of the HPC niche reveals cell-to-cell communication with HPCs

**Figure 5:**
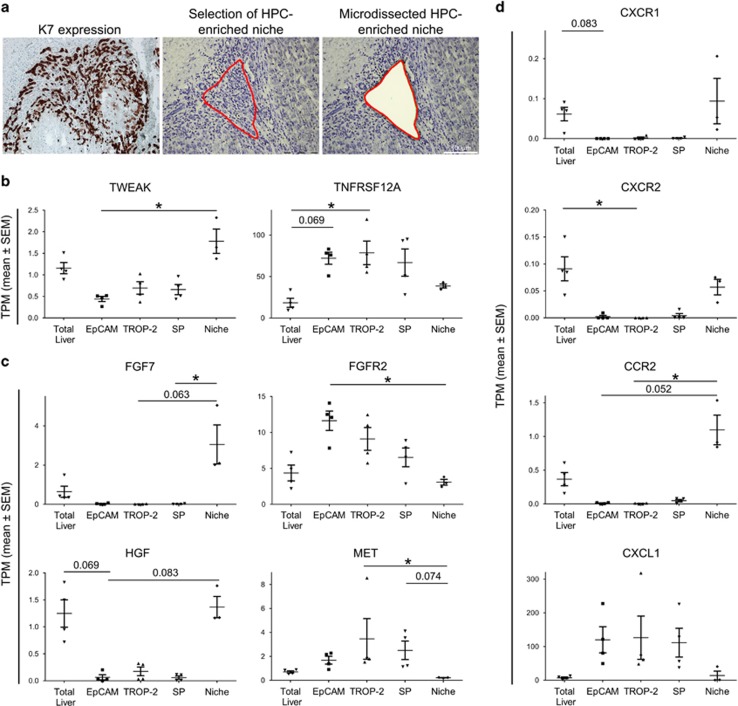
Comparison of isolation via LMD and FACS isolation. (**a**) Example of serial sections of K7 positivity and LMD (before and after dissection) of HPC-enriched regions. (**b**) Median gene expression (TPM±S.E.M.) of TWEAK and TNFRSF12A. (**c**) Median gene expression (TPM±S.E.M) of FGF7, FGFR2, HGF and MET. (**d**) Median gene expression (TPM±S.E.M.) of CXCR1, CXCR2, CCR2 and CXCL1 (*P<0.05; mean±S.E.M.)

We hypothesised that the activated pathways identified by IPA analysis in the isolated HPCs could be directly influenced by the surrounding niche cells.^16^ LMD was performed to isolate HPCs and their surrounding niche based on their morphology and K7 immunohistochemistry (Figure 5a). Regions with a high ductular reaction, excluding lobular ductuli and HCs, were selected (referred to as the niche). As such, the presence of certain expression profiles in the close neighbourhood of the HPCs could shed light on potential interactions between the niche and the HPCs.

We confirmed that *TWEAK* was expressed in the niche (*P*=0.0112) and that HPCs could respond to this through the TWEAK receptor, *TNFRSF12A*, present in all HPC populations (*P*=0.0209; Figure 5b).^23^ A similar pattern could be detected when evaluating the expression of *FGF7* and its receptor *FGFR2* (resp. *P*=0.0087; *P*=0.0248), and *HGF* and its receptor *MET* (resp. *P*=0.0079; *P*=0.0090; Figure 5c). *FGF7* and *HGF* are expressed in the niche but not by the progenitor cells themselves, while the HPCs showed enriched expression of the receptors *FGFR2* and *MET* (Figure 5c). Potential candidates in the niche are activated stellate cells, known to express HGF and FGF7.^23^ Enriched expression of the neutrophil markers *CXCR1*, *CXCR2* and *CCR2* in the niche, combined with the expression of neutrophil attractant *CXCL1* by HPCs suggested that HPCs were capable of attracting and recruiting neutrophils into the liver (resp. *P*=0.0048; *P*=0.0048; *P*=0.0048; *P*=0.0162; Figure 5d).

**Figure 6:**
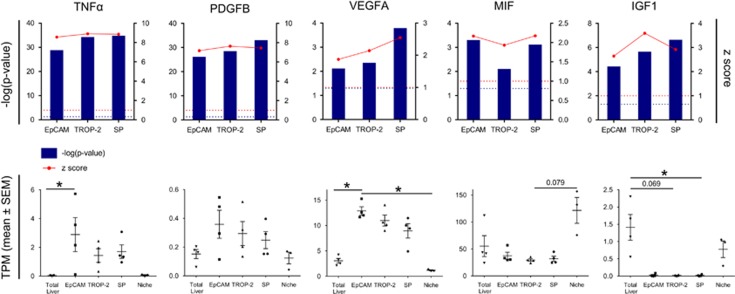
Identification of cytokines and growth factors expressed in human HPCs or in the niche. Upper panel: Different significant (−log(*P*-value)) cytokines and growth factors linked with HPC and their activation (*z*-score) in the different isolated groups *versus* total liver. Blue dotted line=significant *P*-level (0.05). Red dotted line=*z*-score limit of 1 (*z*-score>1 is activation, *z*-score<1 is inhibition of pathway). Lower panel: Median gene expression (TPM±S.E.M.) of TNF*α*, PDGFB, VEGFA, MIF and IGF-1 (*P<0.05; mean±S.E.M.)

To identify novel cytokines and growth factors that can influence HPC activation we searched for potential upstream regulators using IPA analysis. We identified several cytokine pathways that were significantly activated in the HPCs and verified their respective cytokine expression in HPCs and the niche (Figure 6). A first group consisted of *TNFα*, *PDGFB* and *VEGFA*, which are cytokines produced by the HPCs and act as significant upstream regulators in all isolated HPC populations (resp. *P*=0.0107; *P*=0.3895; *P*=0.0036). *MIF* and *IGF-1* formed a second group, whose expression was enriched in the niche and their pathways were activated in the HPCs, suggesting that niche cells produced MIF and IGF-1 to stimulate HPCs (resp. *P*=0.0641; *P*=0.0108; Figure 6).

### The IL-17 A signalling as a new pathway involved in HPC metabolism

**Figure 7:**
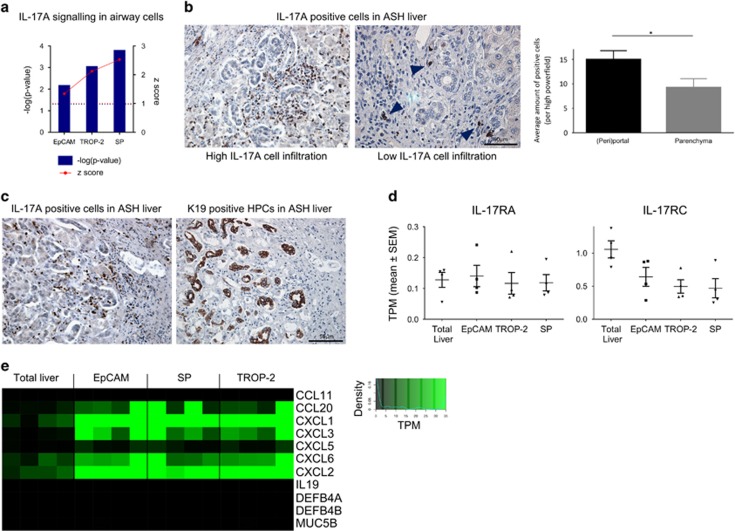
Interleukin-17A (IL-17 A) pathway as a potential player in human HPC activation/proliferation. (**a**) Significant (−log(*P*-value)) IL-17 A pathway and the activation (*z*-score) in the different isolated groups *versus* total liver. Blue dotted line=significant *P*-level (0.05). Red dotted line=*z*-score limit of 1 (*z*-score>1 is activation, *z*-score<1 is inhibition of pathway). (**b**) Immunohistochemical evaluation of IL-17 A^+^ cells in alcoholic liver: high *versus* low (arrowheads) infiltration. Comparison of the amount of IL-17 A^+^ cells in (peri)portal and parenchyma regions of the liver (average amount of IL-17 A^+^ cells per high powerfield in parenchyma or (peri)portal region) (*P<0.05; mean±S.E.M.). (**c**) IL-17 A^+^ cells and K19^+^ cells in the same region on serial slices. (**d**) Median gene expression (TPM±S.E.M.) of IL-17RA and IL-17RC (*P<0.05; mean±S.E.M.). (**e**) Median gene expression of downstream targets of IL-17 A signalling in airway cells

One of the top canonical pathways activated in all three HPC-enriched populations was the IL-17 A pathway (Figure 7a). When looking at IL-17 A mRNA expression in the liver, hardly any *IL-17 A* expression could be detected in the total liver cell population indicating low expression. On protein level, IL-17 A-producing cells could be detected in the portal tracts via immunohistochemical staining. The amount of IL-17 A^+^ cells varied between patients but the distribution showed great similarities: the cells were scattered throughout the liver but mainly present in the portal tracts, closely to the HPCs (Figures 7b and c). IL-17 A can activate the IL-17 A pathway through binding with the receptors IL-17RA and IL-17RC inducing a signalling cascade resulting in the production/activation of c-JUN, c-FOS and the expression of chemokines and interleukins. There were no differences detected in the expression of *IL-17RA* and a slightly lower expression of *IL-17RC* could be seen in HPCs when compared with total liver (resp. *P*=0.7817; *P*=0.0687; Figure 7d) not excluding a possible IL-17 A interaction with HPCs. The expression of the downstream targets of the IL-17 A pathway was higher in the HPC-enriched groups in comparison with the total liver (Figure 7e). Induced genes like *CCL20*, *CXCL1* and *CXCL2* are involved in chemoattraction of immune cells like macrophages, Th17 cells and neutrophils,^24, 25, 26^ suggesting that HPCs might be induced through IL-17 A to stimulate the immune cell response in the liver.

## Discussion

In this paper, we used different approaches to isolate human HPCs and its niche from ASH livers and performed the first, RNA-seq-based, comparative transcriptome analysis of isolated human HPCs. We demonstrated that (i) in humans, TROP-2 is expressed in cholangiocytes and HPCs during liver disease and can be used to isolate human HPCs; (ii) TROP-2^+^ and EpCAM^+^ HPC populations from ASH livers have very similar transcriptome profiles; (iii) unbiased analysis of the transcriptome data identified novel pathways involved in cellular communication between the HPCs and its niche.

As described by Okabe *et al.*,^15^ TROP-2 can be used to isolate activated mouse HPC populations also known as oval cells, as in mice, TROP-2 is only expressed in activated oval cells when liver disease is induced. In contrast, in humans, we detected the expression of TROP-2 in cholangiocytes and HPCs in healthy livers. When the liver disease progresses and fibrosis develops, TROP-2 is expressed in cholangiocytes and in activating and proliferating HPCs. Thus, we demonstrate that in human livers, EpCAM and TROP-2 are both markers of cholangiocytes and activated HPCs.

Marker clustering, PCA/Pearson's analysis and immunohistochemical validation indicated the successful isolation of HPC-enriched populations from human ASH livers, which were used for further analysis. EpCAM and TROP-2 are two closely related proteins with slightly different expression in the human liver. Although EpCAM is also expressed in a large population of intermediate HCs, TROP-2 and EpCAM gene expression profiles are highly similar, probably because of the exclusion of most of the larger cells through Percoll gradient centrifugation. RNA-seq analysis revealed great similarities between the EpCAM^+^ and TROP-2^+^ populations (2097 commonly enriched genes), while SP cells differed substantially from these two HPC populations. This lower transcriptional profile similarity between SP and EpCAM or TROP-2 isolated cells is most likely due to the difference in isolation method: SP is a functional assay based on the presence and functionality of ABC transporters, while EpCAM and TROP-2 isolations are based on the presence of membrane proteins. Still, all HPC-enriched populations are successfully separated by PCA from their respective total livers. Differences in activated/inhibited pathways between the isolation methods could be an indication of the presence of different sub-populations of HPCs in the human liver. The high amount of commonly activated genes, however, indicates that the different populations were highly similar and only different in some metabolic pathways or immune pathways, perhaps because of an incomplete exclusion of CD45^+^ cells in the SP fraction. Furthermore, SP is technically challenging: an expensive UV laser is needed, much optimisation is required, long incubation periods that challenge the cells, the potential toxicity of Hoechst and verapamil and the relative short action span of verapamil blocking. The potential Hoechst toxicity could prevent the use of the isolated cells in subsequent *in vitro* assays. Isolations based on the presence of membrane proteins like EpCAM and TROP-2 are less challenging and less prone to contamination. So, taken into account the potential difficulties with SP (technically, immune cell contamination) and EpCAM (intermediate HCs) isolation, we prefer the TROP-2 isolation as it is a relatively easy technique without many pitfalls and is most likely to result in the most pure and viable HPC populations.

IPA analysis of the three isolated groups confirmed the role of several pathways involved in the activation and differentiation of HPCs like the Wnt, TWEAK, TGF-*β*, HGF and FGF pathway.^17, 18, 19, 20, 27^ The Wnt/*β*-catenin pathway is involved in the proliferation of human HPCs as activation of the pathway leads to the expansion of HPCs.^16^ TGF-*β* signalling results in the release of *β*-catenin, thereby activating the Wnt pathway and regulating the activation and differentiation of HPCs.^28^ TWEAK, HGF and FGF are known inducers of HPC activation.^23^ Macrophages and other immune cells like CXCR4^+^ T cells can be a source of the elevated TWEAK levels in diseased livers. Studies have shown that the activation of TNFRSF12A (FN14, CD266) by TWEAK can lead to activation and expansion of HPCs.^29, 30^ Takase *et al.*^19^ showed that in FGF7-deficient mice HPC expansion after liver injury was deprived. In contrast to EGFR-mediated NOTCH1 signalling, which controls the biliary commitment of HPCs, HGF controls the differentiation of HPCs toward HCs via MET-driven AKT/STAT3 activity.^31^ The activation of HC lineage committing pathways, like HGF and Wnt signalling, can be correlated with the HC regeneration inhibitory effects of alcohol.^32, 33^ We confirmed the enriched expression of the receptors in the isolated HPC populations while their ligands were enriched in the niche (Figure 5), thereby confirming the importance of these pathways in ASH livers.

In addition to already known HPC pathways, we were able to identify autocrine signals thus far not associated with HPC biology (Figure 6). One of these autocrine signals is PDGFB, which is known to modulate neovascogenesis and osteogenesis by stimulating mesenchymal stem cells to differentiate into epithelial cells or osteoprogenitor cells.^34, 35^ VEGFA, another autocrine signal, has been described to contribute to the immobilisation of progenitor cells and the maintenance of stem cells,^36, 37, 38^ while TNF*α*, a well-known proinflammatory cytokine, can promote osteogenic differentiation of mesenchymal stem cells.^39^ Gene expression of these cytokines is enriched in human HPCs (and not in niche cells) and can differentially regulate their respective signalling pathways in HPCs. While HPC-specific knockout studies of these cytokines are necessary to unambiguously establish a role for these cytokines in HPC biology, our data strongly suggests that PDGFB, TNF*α* and VEGFA are part of a autocrine signalling pathway in HPCs that stimulate activation of these cells.

We also revealed paracrine signals, like MIF and IGF-1, that are produced by the niche and stimulate respective pathways in HPCs. Studies have shown that IGF-1 is crucial for the terminal osteoblast differentiation of mesenchymal stem cells^40^ and can even stimulate the differentiation of mesenchymal stem cells towards HC-like cells.^41^ This suggests that, in ASH livers, IGF-1 could be released by niche cells to favour the differentiation of HPCs towards HCs. MIF, on the other hand, could be important for the expansion of HPCs in ASH as MIF can promote cell survival, proliferation and self-renewal of other stem cells such as neural stem cells.^42^

Modulation of these novel auto- and paracrine HPC signals in animal models of liver disease will show whether these cytokines can have an impact on disease progression or HPC activation. With respect to HPC activation, the TNF*α* antagonist Infliximab could inhibit HPC activation and fibrosis in both a mouse model of steatosis and cholestasis.^43^ However, while early clinical trials with alcoholic hepatitis patients have shown promising results with the single treatment with Infliximab,^44, 45^ randomised trials using either IFX or Etanercept (soluble TNF receptor) were disappointing.^46, 47^

Expression profiling of the HPCs and their niche also suggests an active participation of HPCs in immunomodulation. HPCs express immune cell chemoattractant genes like *CXCL1*, while the niche is enriched in immune cell markers, like *CXCR1* and *CCR2*, indicative of a recruitment due to these chemoattractants. We furthermore show that the IL-17 A pathway is upregulated in all isolated HPCs. Many studies have found a link between IL-17 A and fibrogenesis. In a mouse model of biliary atresias, IL-17 A was highly produced by *γδ* cells in the liver, thereby inducing inflammation and destruction of the biliary system.^48^ In a study of Meng *et al.*,^49^ mouse models and cell lines were used to demonstrate that IL-17 A could activate Küpffer cells and stellate cells through the activation of Stat3 signalling and as such induce fibrosis and inflammation in the diseased liver. This was confirmed by Tan *et al.*^50^ who showed that knocking out IL-17RA in mice reduced inflammation and fibrosis in a CCl_4_ liver disease model. In our study, human HPCs showed an enhanced IL-17 A pathway, indicating a potential interaction between HPCs and immune cells. Previous studies on colonic epithelial cells have shown that IL-17 A represses TNF*α*-induced expression of CXCL10, CXCL11 and CCL5, but, on the other hand, synergised with TNF*α* for the induction of genes like *CXCL1*.^51^ Our results show a similar trend; HPCs express and respond to TNF*α* (Figure 5), while the expression of *CXCL10*, *CXCL11* and *CCL5* (resp. *P*=0.7340; *P*=0.4520; *P*=0.0146) are not elevated in HPCs in comparison with total liver while CXCL1 is elevated (Figure 4d and Supplementary Figure 6). This could suggest that in ASH livers IL-17 A might regulate the expression of TNF*α*-induced genes in HPCs. Although our data in combination with literature indicates a potential role for IL-17 A signalling, further evidence from *in vitro/in vivo* functional work is needed to proof and unravel the interaction with HPCs.

In conclusion, we successfully isolated three distinct HPC cell populations from human ASH livers. RNA sequence analysis of HPCs with their niche revealed thus far unknown signalling cascades involved in HPC activation and cell-to-cell communications between the niche and HPCs in ASH livers and suggest that HPCs can actively contribute to liver inflammation.

## Materials and methods

### Human liver samples

Seven explant livers of patients diagnosed with ASH between 2009 and 2015 at the University Hospitals in Leuven (Belgium) were included in this study. From these seven patients, the SP, EpCAM^+^ and TROP-2^+^ populations and whole liver extracts from four samples, which met the RIN (≥7) and yield (≥200 ng/*μ*l) requirements, were used for RNA-sequencing analysis. The same samples were used for the LMD part, although one of the samples did not meet the required quality and was excluded from LMD isolation. Immediately after surgical removal of the diseased liver during orthotopic liver transplantation, tissue biopsies were taken and fixed in 6% formalin-fixed, paraffin-embedded (FFPE) samples or snap frozen in isopentane-cooled liquid nitrogen. Another part of the explant liver was cut in small blocks of 1 mm by 1 mm to be frozen overnight in Recovery Cell Culture Freezing Medium (Life Technologies, Paisley, UK) on −80 °C, after which the samples were stored in liquid nitrogen. Only end-stage ASH livers without tumour nodules and previous chemo and/or RFA treatment were selected in the study. FFPE samples of healthy liver, early, septal and late-stage alcoholic livers were collected from the University Hospitals' databank. The study was approved by the ethical committee of the University Hospitals. Detailed background information of the patients used for RNA-sequencing can be found in Supplementary Table 1.

### Immunohistochemistry

Immunohistochemistry was performed on paraffin-embedded sections (5 *μ*m) or cryostat cut sections (4 *μ*m). The snap-frozen sections were fixed in acetone for 10 min. Epitope retrieval was performed on the paraffin-embedded sections in a pre-treatment module (Dako, Heverlee, Belgium) according to the manufacturer’s instructions in citrate buffer (pH 6) or in EDTA-Tris buffer (pH 9). After blocking the endogenous peroxidase activity with Dual Endogenous-Enzyme Blocking Reagent (Dako, Heverlee, Belgium), the sections were incubated with the primary antibody against TROP-2 (1/20; Abcam, Cambridge, UK), EpCAM (Ber-Ep4, ready-to-use; Dako), keratin K19 (1/100; Dako), ABCG2 (BCRP; 1/5; Monosan, Sanbio, Uden, Belgium), K7 (ready-to-use; Dako) for 30 min at RT. Following PBS washing (pH 7.2), the slides were incubated with horseradish peroxidase-labelled EnVision FLEX System (Dako) for 30 min at RT. IL-17 A (1/50; R&D Systems, Minneapolis, MN, USA) had an overnight incubation, followed by 1 h incubation of horseradish peroxidase-labelled goat anti-serum (1/20; Dako). After washing with PBS, the peroxidase activity in FFPE sections was detected using 3.3′-diaminobenzidinen, DAB (Dako) as a substrate, revealing a brown reaction product, and in frozen sections using 3-amino-9-ethylcarbazole, AEC (Dako), revealing a red reaction product. Finally, the sections were counterstained with Mayer’s haematoxylin and mounted. Pictures were taken by Leica DMLB (Leica Microsystems, Herborn, Germany).

### Immunofluorescence

To identify the expression profile of TROP-2^+^, EpCAM^+^ and ABCG2^+^ cells, immunofluorescent double staining was performed on 4 *μ*m cryosections. In series, the primary antibodies were incubated against K19 (1/100; Dako) and EpCAM (Ber-Ep4, ready-to-use; Dako), K19 (1/100; Dako) and TROP-2 (1/20; Abcam), K19 (1/100; Dako) and ABCG2 (BCRP; 1/10; Monosan). Alexa Fluor 488 goat anti-mouse and Alexa Fluor 568 goat anti-mouse (Life Technologies, Brussels, Belgium) were used as secondary antibodies and were incubated for 30 min at RT. Counterstain was performed with 4',6-diamidino-2-phenylindole (1/10 000; Invitrogen, Breda, The Netherlands) for 15 min. PBS was used for the rinsing steps and the slides were covered by ProLong Gold Antifade Reagent (Life Technologies, UK). Pictures were taken by Zeiss Axioplan (Carl Zeiss, Oberkochen, Germany).

### Tissue processing and cell isolation

#### Tissue dissociation

Tissue dissociation and SP isolation were done as described previously.^8^ The samples stored in liquid nitrogen were thawed at 37 °C and washed with Hank’s balanced salt solution (Life Technologies, UK). After dissociation using Liberase Blendzyme 3 (Roche, Basel, Switzerland) at a concentration of 0.8 Wunsch unit/ml during 1 h at 37 °C, the samples were filtered with a 70 *μ*m nylon mesh filter (BD Biosciences, Erembodegem, Belgium) and gradient centrifugation based on 15% Percoll/Hank’s balanced salt solution (100 × *g* for 15 min). Finally, the pelleted cells were resuspended in HepatoZYME-SFM (Life Technologies, UK) with 1% penicillin/streptomycin (Life Technologies, UK) added to obtain a single-cell suspension.

#### Cell isolation based on FACS

To isolate the SP of the single-cell suspension, the cells were incubated with 5 *μ*g/ml Hoechst-33342 (0.1 mg/ml; Sigma-Aldrich, St. Louis MO, USA) for 90 min at 37 °C exactly under continuous agitation. A control sample was obtained by incubating the cells with the transport blocker Verapamil (100 *μ*M; Sigma-Aldrich) 20 min before the Hoechst-33342 incubation. As such, the active efflux and SP phenotype could be assessed. To exclude CD45^+^ cells from isolation, the sample was incubated with an antibody against CD45 conjugated with R-phycoerythrin for 30 min (10 *μ*l per million cells; Ab Serotec, Bio-Rad, Puchheim, Germany).

To isolate EpCAM^+^ and TROP-2^+^ cells from the cell suspension, the samples were separately incubated, respectively, with a PE-conjugated antibody against EpCAM (10 *μ*l per million cells; R&D Systems, Minneapolis, MN, USA) or an allophycocyanin-conjugated antibody against TROP-2 (10 *μ*l per million cells; R&D Systems) for 30 min on ice. Aspecific binding of the antibodies was prevented by incubating the samples for 10 min with Fc Blocking Reagent (1/10; BioLegend, London, UK) before antibody incubation.

Propidium iodide (2 *μ*g/ml; Sigma-Aldrich) was added just before FACS analysis to all samples to exclude dead cells. The cell samples were analysed and sorted with a FACSAriaII (BD Biosciences) excluding debris, duplets, CD45^+^ and dead cells by forward scatter, side scatter, CD45 antibody positivity and propidium iodide gating (Supplementary Figure 1). The sorted cells (SP, EpCAM^+^ and TROP-2^+^ cells) were collected in HepatoZYME-SFM (Life Technologies, UK). A part of the sorted cells was lysed in RTL plus buffer (Qiagen, Hilden, Germany) with 1% *β*-mercaptoethanol (Sigma-Aldrich) and stored in −80 °C until mRNA extraction.

#### Validation of FACS isolated cells via cytospin and correlation with DR score

Part of the sorted cells was fixed in BD CytoRich System (BD Biosciences) and processed into cytospins for immunohistochemical validation. The size of the isolated populations was correlated with the ductular reaction in each of the corresponding samples. The ductular reaction or the activation of the HPCs of each sample was assessed by the Ductular Reaction Score, which is the average amount of HPCs (visualised with TROP-2 staining) counted per ten high powerfields.

#### Cell isolation with LMD

Snap-frozen liver samples were used to obtain 10-*μ*m-thick liver sections with cryostat microtomy, which were mounted on DNase- and RNase-free PET-membrane-coated metal frame slides (1.4 *μ*m; Leica, Herborn, Germany). The sections were stored at −80 °C. Cryosections were stained with a fast cresyl violet staining. Based on this cresyl violet staining (cresyl violet, 0.2 g/100 ml; Sigma-Aldrich) and a keratin 7 immunohistochemical staining of a consecutive slide, HPC-rich areas in the liver were visualised and identified. After which, the areas were microdissected using a Leica LMD 6500 system (Leica Microsystems). The dissected fragments were collected on the cap of PCR tubes filled with 20 *μ*l RLT plus buffer (Qiagen) with 1% 2-mercaptoethanol (Sigma-Aldrich). After dissection, the samples were vortexed for 30 s and stored at −80 °C until further processing. The total handling time needed per slide was kept under 20 min.

### mRNA extraction and RNA sequence analysis

Besides mRNA extraction from the FACS and LMD groups, 10-*μ*m-thick whole liver tissue slides, sectioned from frozen tissue, were homogenised and lysed in RTL plus buffer with 1% 2-mercaptoethanol. Total mRNA from whole liver, FACS and microdissection samples was extracted using the RNeasy Plus Micro Kit (Qiagen) according to the manufacturer’s instructions. An Agilent Bioanalyzer (Agilent Technologies, Santa Clara, CA, USA), using RNA Pico Chip, was used to analyse the quantity and quality of the mRNA. Four patients of the FACS isolation part and three patients of the LMD isolation part with a high yield and an RIN value between 6 and 8 were chosen to be used for high-throughput RNA sequence analysis. Quality control and RNA-sequencing were performed at the VIB Nucleomics Core (http://www.nucleomics.be; Leuven, Belgium).

### RNA-seq data processing

First, quality control on all fastq files was performed using FastQC (http://www.bioinformatics.babraham.ac.uk/projects/fastqc). Each fastq file was trimmed and filtered to remove adaptors, polyN nucleotides and low-quality sequences and subsequently confirmed by performing a second quality control. All reads were mapped by Tophat2 on the *Homo sapiens* genome h19, which was downloaded from the UCSC website (http://hgdownload.cse.ucsc.edu). Transcript compilation was performed using Cufflinks with homo sapiens hg19 UCSC transcript annotation. Differential expression was calculated by Cuffdiff and subsequently we converted FPKM to TPM (median gene expression) using RStudio. The data were further analysed with IPA (Inguinity Pathway Analysis) and Bioconductor.^52^ Tophat2, Cufflinks and Cuffdiff were performed within Genepattern (Supplementary Figure 2).

### Data visualisation

Heatmaps, Venn diagrams, Pearson's correlation, PCA and GO analysis was performed using the Bioconductor. FACS analysis plots were carried out with FlowJo (FLOWJO LLC, Ashland, OR, USA). Photoshop (version CC 2017; Adobe, San Jose, CA, USA) was used to brighten and sharpen the immunohistochemical pictures.

### Pathway analysis

The resulting data set of the high-throughput RNA-sequencing was analysed in IPA. Only enriched genes (fold change >2 and *P*<0.05) in EpCAM, TROP-2 and SP data sets compared with the total liver were imported. Within IPA, the canonical pathways, regulatory effects and upstream regulators were analysed.

### Statistical analysis

Significance of all the RNA-seq data was calculated by a nonparametric Kruskal–Wallis one-way ANOVA. Significance of the IL-17 A^+^ cells in parenchyma *versus* (peri-)portal regions was calculated by a Mann–Whitney *U*-test. Statistical analysis was performed with Statistica (version 9, StatSoft. Inc., Tulsa, OK, USA) with a significance level of *α*<0.05.

## Supplementary Material

Supplementary Tables and Figures

Supplementary Gene lists
