# Supplementary material for: RNA-sequencing-based comparative analysis of human hepatic progenitor cells and their niche from alcoholic steatohepatitis livers
Source: Cell Death Dis. 2017 Nov 2;8(11):e3164–. doi: 10.1038/cddis.2017.543 (PMC5775409; doi:10.1038/cddis.2017.543)
Supplement: Supplementary Tables and Figures [file cddis2017543x1.pdf]

Supplementary Table 1: Table with background information of the patients used for the isolation and RNA sequencing. (M=male; F=female; ASH=alcoholic steatotic hepatitis; Y=yes; N=no)

	Gender	*Age at transplantation*	*Transplantation date*	*Diagnosis*	*Stopped drinking since*	*Child-Pugh score*	*MELD score*	*Pathology*	*Smoker*	*Medical non-hepatology background*
Patient 1	M	70	14th March 2011	ASH	2010	B9	7	Micronodular cirrhosis, Lymphocyte infiltration, sinusoidal dilatation, Steatosis	Y (46PY/ stopped since 2006)	Diabetes mellitus since 2008
Patient 2	F	65	18th December 2013	ASH	2012	B9	14	Cirrhosis, sinusoidal dilatation, cholate stasis	Y (50PY/ stopped since 2013)	Diabetes mellitus type 2
Patient 3	M	47	24th April 2014	ASH	October 2013	C12	28	Micro and macronodular cirrhosis, Steatosis	N	/
Patient 4	M	48	27th October 2014	ASH	March 2013	C10	18	Micronodular cirrhosis, Lymphocyte infiltration, sinusoidal dilatation, Steatosis	Y (stopped since March 2013)	/

Supplementary Table 2: List of the common top upregulated pathways.

Pathways	TROP-2	EpCAM	SP
PI3K/AKT Signaling	3.61E-05	2.47E-04	3.47E-08
ILK Signaling	3.45E-05	1.99E-04	1.78E-06
HMGB1 Signaling	4.80E-04	3.00E-03	1.87E-08
RAR Activation	6.75E-05	3.45E-04	3.86E-06
ERK5 Signaling	1.78E-05	6.86E-04	9.35E-06
PPAR Signaling	6.44E-04	1.07E-04	3.65E-05
Toll-like Receptor Signaling	1.35E-04	8.15E-05	2.53E-04
IL-6 Signaling	9.51E-04	1.29E-02	7.27E-06
iNOS Signaling	8.71E-04	1.06E-03	1.34E-04
PTEN Signaling	4.80E-04	3.00E-03	8.83E-05
IL-8 Signaling	5.25E-04	1.02E-02	9.84E-05
IL-10 Signaling	6.10E-04	4.35E-03	3.63E-04
NRF2-mediated Oxidative Stress Response	6.51E-04	6.93E-03	2.92E-04
AMPK Signaling	2.85E-03	1.63E-03	6.56E-04

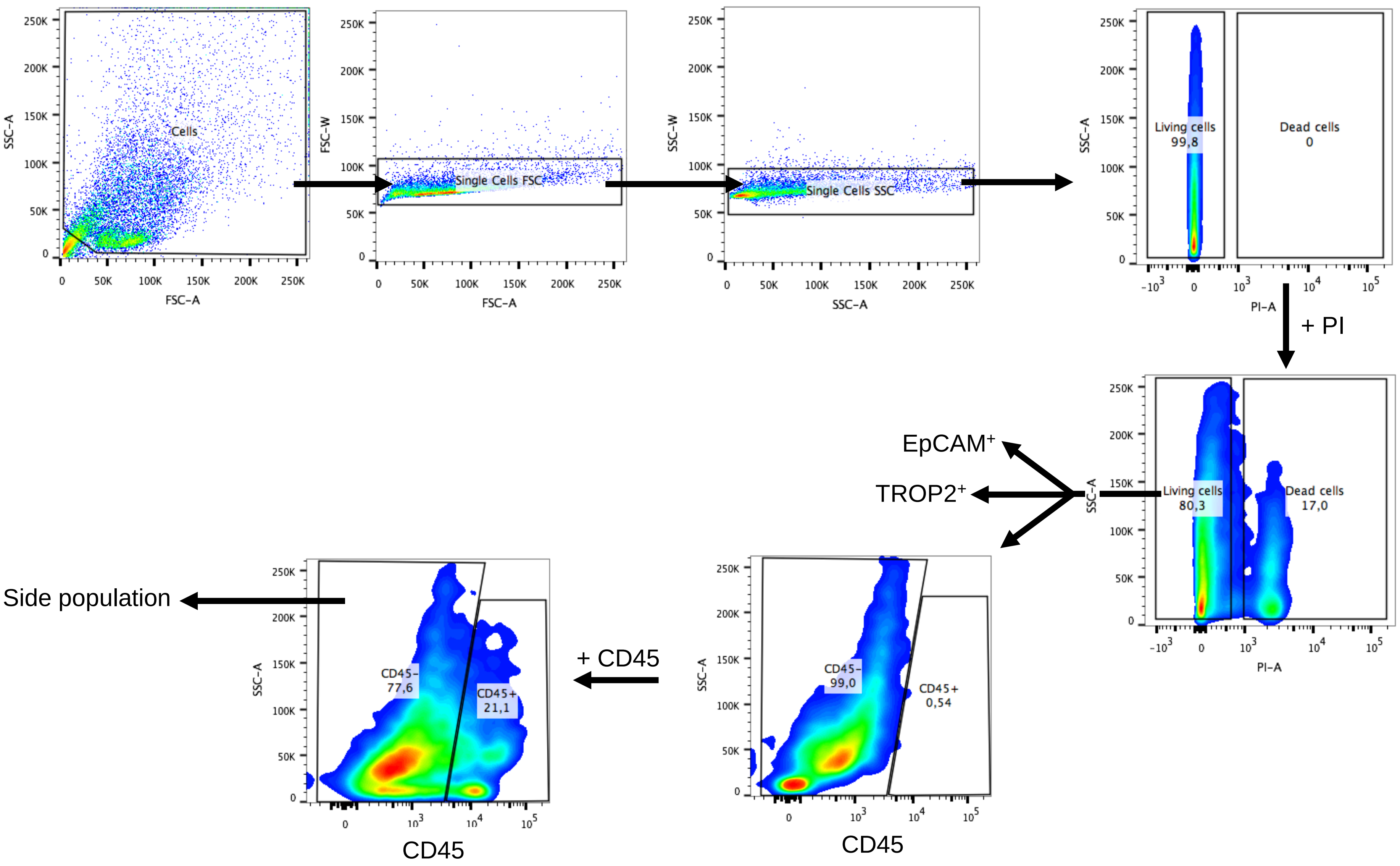

Supplementary figure 1: Overview of the used FACS isolation strategy of the liver samples.

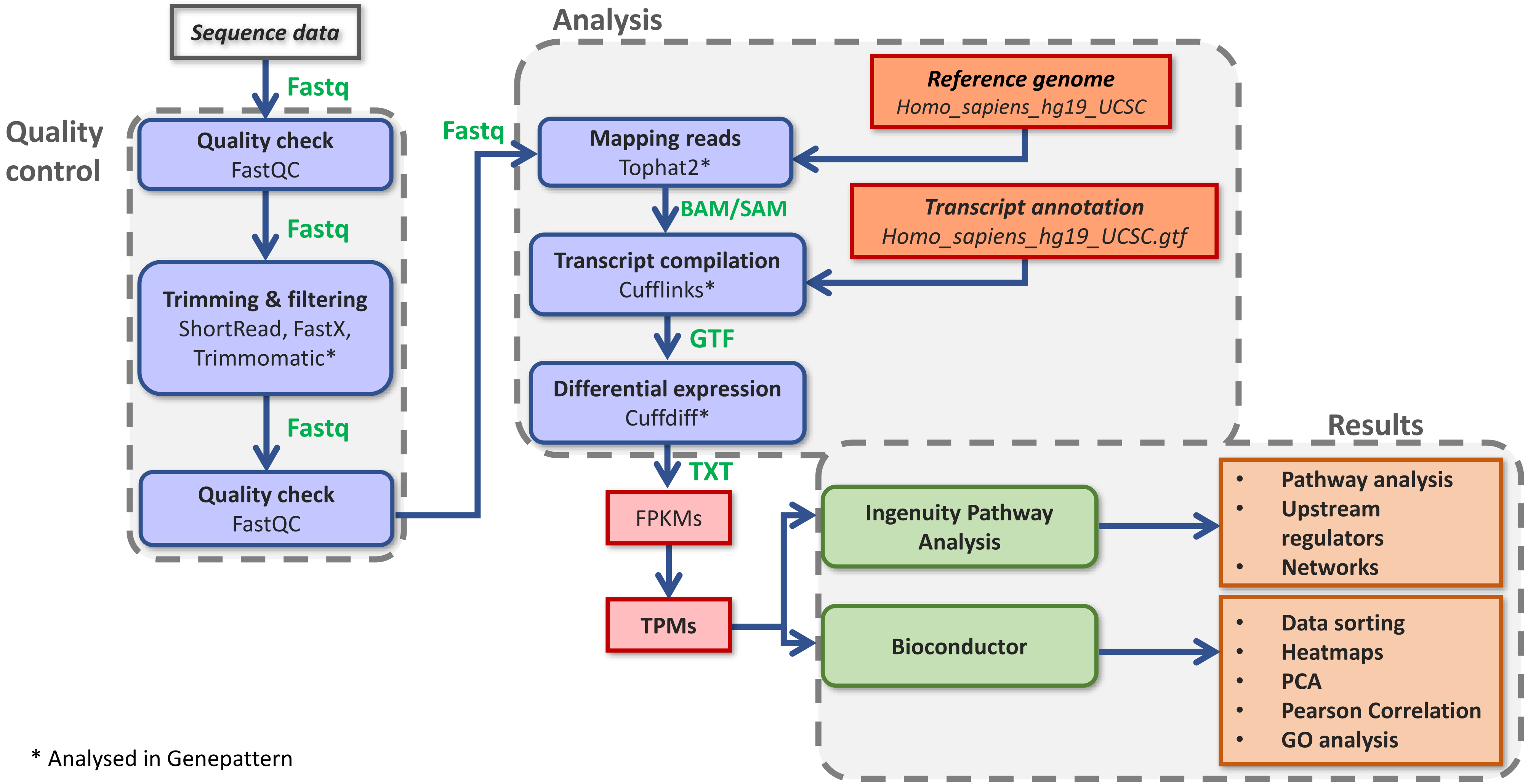

Supplementary figure 2: Overview of the used analysis strategy of the RNA sequence data.

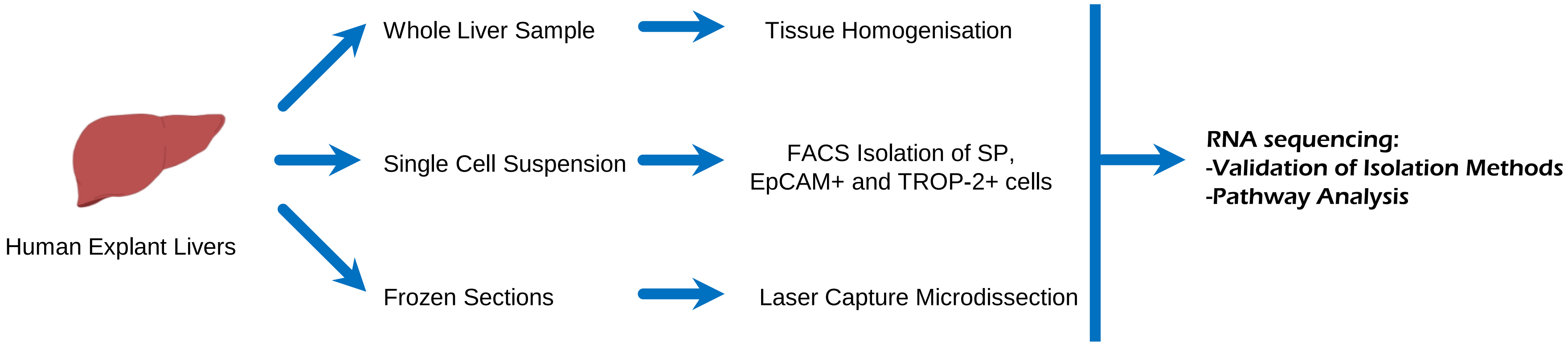

Supplementary figure 3: Overview of the used workflow in this study.

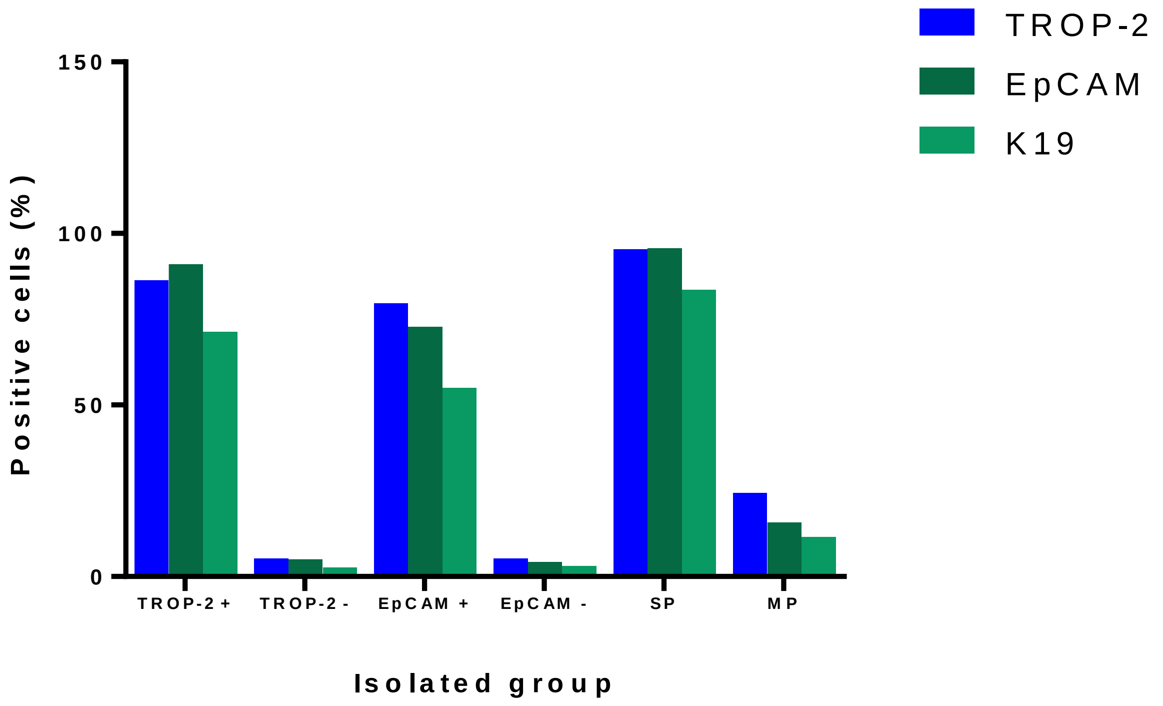

Supplementary figure 4: Evaluation of TROP-2, EpCAM and K19 expression in the FACS isolated groups (TROP-2 positive, EpCAM positive groups and SP), resp. compared with the TROP-2, EpCAM negative population and main population (MP).

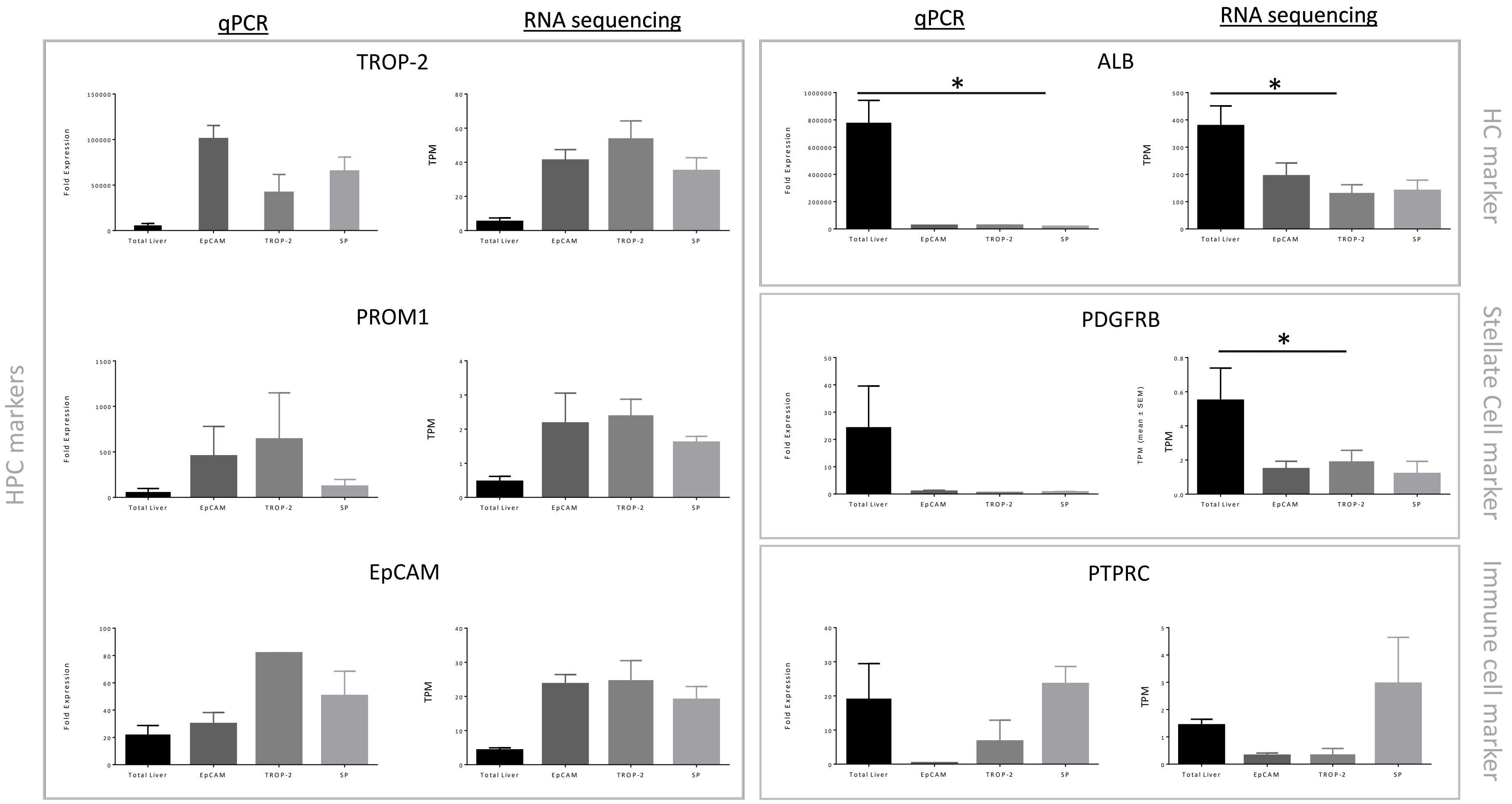

Supplementary figure 5: Comparison of the fold expression (qPCR) and the median gene expression (TPM, RNA-seq) of some selected cell markers of the same samples, indicating a correlation between qPCR and RNA sequencing data. qPCR was done on the same samples of the RNA-seq after amplification and conversion into cDNA with the WT-Ovation™ Pico RNA amplification System (NuGEN Technologies, Bemmel, The Netherlands). qPCR assays were performed using Fast SYBR Green Master Mix (Applied Biosystems, Foster City, CA, USA). Data were analysed using the comparative cycle threshold method with normalisation of the raw data to reference genes glyceraldehyde-3-phosphate dehydrogenase and ribosomal protein L19 were used to normalise the cycle threshold data and fold expression was calculated based on the 2^(- ΔΔCt) method. p<0.05; mean±SEM)

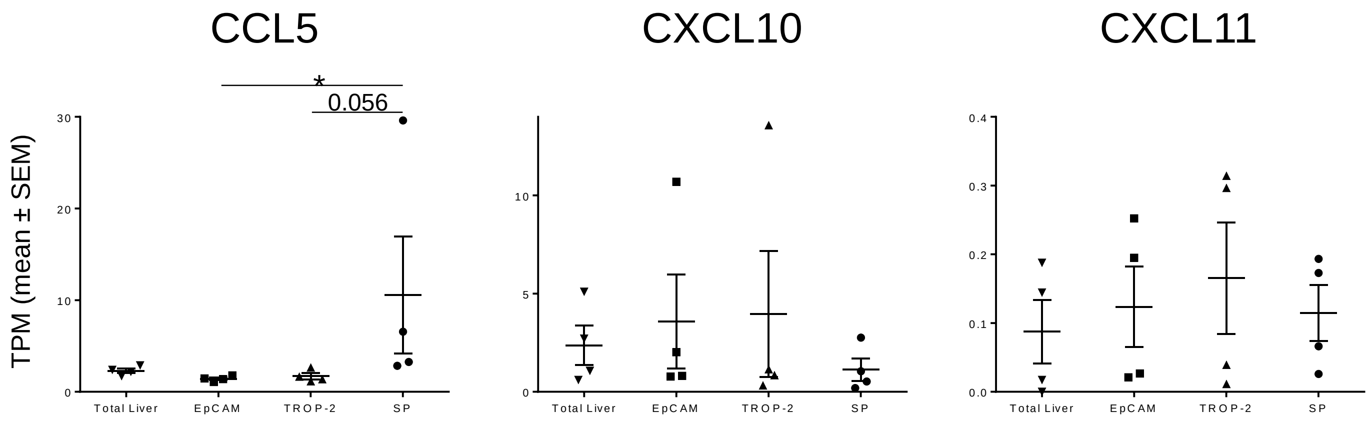

*Supplementary figure 6: Median gene expression (TPM±SEM) of CCL5, CXCL10 and CXCL11. (*p<0.05; mean±SEM)*
