# Supplementary material for: RNA-sequencing-based comparative analysis of human hepatic progenitor cells and their niche from alcoholic steatohepatitis livers
Source: Cell Death Dis. 2017 Nov 2;8(11):e3164–. doi: 10.1038/cddis.2017.543 (PMC5775409; doi:10.1038/cddis.2017.543)
Supplement: Supplementary Gene lists [file cddis2017543x2.pdf]

Supplementary data of Fig. 3a: PCA values

Principle Component analysis

	PC1	PC2	PC3
**liver_p1**	-125,6076301	3,443413208	0,884815243
**liver_p2**	-128,3564567	1,534485632	-0,268848989
**liver_p3**	-115,8280064	6,276526299	5,518648572
**liver_p4**	-130,9739206	-10,54668262	-12,50825106
**Epcam_p1**	34,95257891	-2,617781875	7,211184626
**Epcam_p2**	40,6613363	8,705044457	58,76799032
**Epcam_p3**	46,25387683	44,6405357	-45,88064953
**Epcam_p4**	36,93036445	-63,42242618	-30,15384477
**SP_p1**	47,96029919	-62,04487749	-24,09018779
**SP_p2**	37,01474379	35,99864984	18,97748175
**SP_p3**	38,32935749	-16,09421389	56,38716828
**SP_p4**	45,34418995	53,39580773	-28,61420428
**TROP2_p1**	36,52765888	14,84233855	17,66737048
**TROP2_p2**	41,97465279	18,98963881	43,34048781
**TROP2_p3**	47,84599541	50,87159993	-51,91266317
**TROP2_p4**	46,97095983	-83,9720581	-15,3264975

Supplementary data of Fig. 3b: specific genes in all groups of the clustering

Gene clustering

**Genes only upregulated in:**	**SP**	**EpCAM**	**Trop-2**
	ABCA7	AADACL4	A2MP1
	ABCC8	ABO	ABCA13
	ACAP1	ACCS	ABCA17P
	ADAMTSL1	AGER	ACAN
	AGAP2	ALS2CR11	ACCN2
	AIM1	AMZ1	ACCN4
	AKNA	ANKRD22	ACPP
	ALAS2	ANO7	ADGB
	ALG1L2	ATP1A4	ANGPTL5
	ANKRD19P	AURKB	ANO2
	ANKRD28	B4GALT2	ARHGAP11B
	ANLN	BEGAIN	B3GALT5
	APBA2	BOLL	C11orf16
	APOBEC3D	BPY2B	C19orf45
	APOBEC3G	BTBD18	C1orf98
	APOLD1	C11orf36	C2orf50
	AQP8	C11orf42	C6orf164
	ARHGAP9	C11orf45	CA13
	ARHGEF3	C14orf176	CATSPER3
	ATP1A3	C1QL3	CBLN2
	ATP1B3	C1QTNF3	CCDC160
	ATP2A3	C1orf129	CCL7
	B4GALNT1	C1orf227	CD164L2
	BATF	C22orf26	CD9
	BCL2A1	C2orf63	CHTOP
	BIRC2	C2orf71	CLDN16
	BLM	C4orf37	CLEC18C
	BMX	C6orf163	CMTM5
	BTBD11	C8orf31	CRB1
	BVES-AS1	CACNB4	CSMD2
	C10orf2	CALB2	CXorf65
	C12orf68	CALHM3	CYP27C1
	C16orf54	CASC2	CYP2W1
	C17orf72	CCDC150	DLG2
	C1orf177	CCDC158	EIF3CL
	C1orf96	CCDC62	FAM177B

C20orf166-AS1	CD1A	FAM22F
C20orf195	CDKL4	FAM48B1
C20orf201	CELA3A	FAM70A
C21orf91-OT1	CERS1	FAM83A
C3orf62	CHRFAM7A	FDCSP
C4orf44	CHST6	FHAD1
C5orf52	CKLF-CMTM1	FLJ12334
C6orf195	COL4A5	FNDC8
C9orf163	CRIPAK	FZD10
C9orf169	CST4	GALNT9
C9orf50	CXorf57	GCOM1
CA1	CYCSP52	GEM
CA10	CYP4F22	GJA9
CARD11	DAZ4	GPR77
CASP8	DKFZP434A062	HAP1
CCDC116	DKFZp451B082	HORMAD1
CCDC88B	DNAJC27-AS1	HOXD9
CCDC88C	DPY19L2P3	HTR3C
CCIN	DUSP9	IMPG1
CCL5	EBLN2	ISL2
CCL8	EGFEM1P	KIFC1
CCND2	EIF2C1	KRT85
CCR6	FAM13A-AS1	LAMP3
CD160	FAM156A	LEFTY2
CD200	FAM186B	LGALS7B
CD244	FAM19A1	LGALS9B
CD247	FAM209B	LINC00470
CD28	FAM24B	LINC00538
CD69	FAM47E-STBD1	LOC100130357
CD7	FAM90A10	LOC100132287
CD96	FAM90A13	LOC100134015
CDHR1	FAM90A14	LOC100144595
CENPF	FAM90A5	LOC100289211
CHST8	FAM90A8	LOC100506035
CITED2	FAM90A9	LOC100506655
CLCA3P	FAM98B	LOC100507240
CLIC3	FANK1	LOC283392
CLNK	FER1L6-AS1	LOC286190
CNR2	FHIT	LOC286297
CORO7	FLJ35946	LOC339807
CPLX1	FLJ40852	LOC407835
CPNE7	GAGE10	LOC439990
CRCT1	GNAZ	LOC441461

CRTAM	GPR20	LOC641746
CST7	GRIK4	LOC643339
CTSW	H2AFY2	LOC643802
CXCR3	HCG23	LOC647323
CXCR4	HJURP	LOC728723
CXCR6	HRASLS	LRRC4
CYP26C1	HRH4	LYPD2
CYTIP	HSD17B7P2	MAGEB10
DBF4	KIF24	MAP6D1
DERL3	KRT19P2	MED12L
DGKA	LINC00235	MEF2B
DKK2	LINC00535	MESTIT1
DLL1	LOC100128682	MIR671
DNAH17	LOC100129345	MS4A8B
DOC2B	LOC100129858	MYBL2
DOCK10	LOC100130417	MYBPC2
DOK2	LOC100132724	MYH7
DRD1	LOC100190940	MYO1A
DTHD1	LOC100288842	NEBL
DUSP26	LOC100505839	NKX2-3
E2F1	LOC100506025	NOS2
ELK2AP	LOC100506469	NPPB
EME2	LOC100507086	OCM
ENSA	LOC100507091	OLIG1
EOMES	LOC100507373	OR10A6
ERP27	LOC100507424	OR2A42
FAM107A	LOC100507589	PCA3
FAM117A	LOC151174	PCDHB9
FAM124B	LOC282980	PEAK1
FAM179A	LOC284260	PLXNA1
FAM212B	LOC285889	POM121L1P
FAM83D	LOC388906	PPFIA2
FAM90A1	LOC390660	PPP1R27
FAS-AS1	LOC400958	PRAMEF4
FASLG	LOC401431	PRSS1
FBLN2	LOC440288	PRSS42
FBN3	LOC643201	PRTG
FBXO5	LOC649330	PTPRN
FCRL3	LOC728373	RFPL1-AS1
FCRL6	LOC728405	RHCG
FEV	LRFN5	RNF186
FHL5	LRRC37A3	ROPN1
FLJ27354	LRRN4CL	RPS10-NUDT3

FNBP1	MAGEA10	SELP
FNDC9	MAP9	SERINC4
FRMD1	METTL21CP1	SERPINB8
FSBP	MGC45922	SFMBT1
FSD1	MIR22	SLC12A3
FXYD7	MIR2276	SLC12A5
FYN	MS4A14	SLC47A2
GAGE12I	MTRNR2L3	SLED1
GALR2	MUC16	SNORA31
GATA2	MYBPC1	SNORA5C
GATA3	MYO15A	SOX2
GBP5	NAA40	TAF7L
GDF1	NCCRP1	TAS2R14
GDF6	NEFH	TCEAL6
GEMIN8P4	NEUROG3	TCEB3CL
GFI1	NKX6-1	TEKT1
GGN	NLRP12	TM4SF19-TCTEX1D2
GHRLOS	NME9	TMEM145
GK3P	NTN3	TP73
GLCCI1	OOEP	TRPC6
GNG4	OR52A5	TTTY6
GNLY	OSM	UCP3
GPCPD1	PANX3	VN1R2
GPR111	PAPL	XAGE2B
GPR114	PCBP4	ZBTB20-AS1
GPR132	PCDHB1	ZNF213
GPR174	PCDHGA9	ZNF284
GPR18	PDE1C	ZNF419
GPR19	PKD2L1	ZNF732
GPR55	PLD5	
GPR68	PLSCR5	
GPR97	POMZP3	
GRASP	POU5F1P4	
GRIK1-AS1	PPIEL	
GRM7	PRR4	
GSG2	PRRT3	
GTF2A1L	PRRX2	
GUCY2D	RABL2B	
GYPA	REG1A	
GZMM	RNF113B	
HAUS3	SCARNA9	
HBM	SEC1	
HCG26	SEC14L4	

HELB	SHOX2
HEMGN	SIAH3
HERC1	SNORA8
HES6	SNORD89
HIST1H2BE	SNX31
HIST1H3H	SOX15
HIST2H2AA4	SPATC1
HMGB2	SPDYE8P
HMHA1	SPEF1
HOTAIRM1	ST8SIA5
HOXA1	SYN3
HOXA10	TAS1R3
HOXA2	TDGF1P3
HOXA3	TDH
HOXA7	TEX12
HOXC4	THUMPD2
HOXD-AS1	TIAF1
HSF5	TM4SF19
IDI2	TMEM180
IDS	TMEM52
IGLL5	TNN
IKZF3	TRIM6-TRIM34
IL12RB1	TRIM64C
IL12RB2	TSPAN10
IL18RAP	TSSK3
IL2	TTL
IL23R	USP17L6P
IL2RA	UTS2D
IL2RB	VWA3B
IL2RG	XCR1
IL4I1	ZFR2
IL7R	ZNF221
INHA	ZNF280B
INPP4A	ZNF605
ITGAD	ZNF664-FAM101A
ITGAL	ZNF676
ITK	ZNF774
ITPKB	ZNF883
IVNS1ABP	ZRANB2-AS2
IZUMO4	
JAKMIP3	
JAM2	
JSRP1	

KCNA3
KCNH7
KCNQ5
KIAA0415
KIAA0922
KIAA1024
KIAA1257
KIAA1751
KIF20B
KIF21B
KIR2DL1
KIR2DL3
KIR2DL4
KIR3DL1
KIR3DL2
KIT
KLF1
KLHL6
KLK13
KLRB1
KLRC1
KLRC2
KLRC3
KLRC4
KLRC4-KLRK1
KLRD1
KLRF1
KLRK1
KRT27
LAT2
LAX1
LCN6
LDHAL6A
LFNG
LINC00204A
LINC00260
LINC00426
LINC00548
LINGO3
LIPE
LIPJ
LOC100129924
LOC100130231

LOC100270804
LOC100287314
LOC100287814
LOC100289092
LOC100506779
LOC149773
LOC154761
LOC283553
LOC283710
LOC283888
LOC285740
LOC339666
LOC340508
LOC344967
LOC375295
LOC389458
LOC439949
LOC641518
LOC642826
LOC646862
LOC646999
LOC653061
LOC653075
LOC653653
LPAR3
LPXN
LRMP
LRRC16B
LRRC3B
LRRC71
LRRC73
LRRN3
LTA
LURAP1
LY9
M1
MADD
MAP1LC3B
MAPKAPK2
MATK
MCTP1
MCTP2
MEIG1

MIR137HG
MIR181A2HG
MIR4687
MIR4707
MIR564
MIR650
MLC1
MYBL1
MYO1G
NBEAP1
NCR1
NCR3
NKG7
NLRC3
NLRP1
NLRP6
NOG
NT5C1B-RDH14
NTRK1
NXPH4
OR2W3
OSR2
OVCH1
P2RX5
PAGE5
PARP15
PBXIP1
PCDH9
PDCD1
PDE4B
PENK
PHF12
PIK3CD
PIK3CG
PIM2
PITPNC1
PLCB2
POTEH
PPP1R16B
PRDM1
PREX1
PREX2
PRF1

PRKCH
PRSS57
PTGDR
PTGER2
PTK2B
PTPN22
PTPN7
PTPRC
PTPRE
PTTG1
PWRN1
PYHIN1
RAD51AP2
RAET1K
RASGRP1
RASL10A
RASSF5
RGS2
RHAG
RIBC2
RLTPR
RNF168
ROPN1L
RORA
RPL13AP6
RTKN2
RUNX1-IT1
RUNX2
RUNX3
S1PR5
SAMSN1
SAPCD2
SATB1
SCARNA10
SCARNA22
SCG3
SEMA3G
SEMA4D
SEMA6B
sep/01
SERF1A
SERHL
SERPINE3

SFMBT2
SH2D1A
SH2D1B
SH2D2A
SHCBP1
SIDT1
SIGLECP3
SKIL
SLA
SLA2
SLAMF1
SLAMF7
SLC16A3
SLC17A7
SLC17A8
SLC1A4
SLC38A5
SLC4A1
SLC4A10
SLFN5
SMC4
SMCHD1
SNAI3
SNHG1
SNORA14A
SNORA32
SNORA41
SNORA53
SNORA5A
SNORA61
SNORD83B
SNORD90
SOST
SPINK2
SPN
SPOCK2
STAM
STAP1
STARD9
STAT4
STK10
STK17B
SUV39H1

SYNGAP1
SYT4
SYT5
SYTL1
SYTL3
TARP
TBX21
TCF7
TFIP11
TGFB1
TGIF2-C20ORF24
THAP2
TIGIT
TMC8
TMEM31
TMIGD2
TNFRSF11B
TNFRSF25
TNFRSF4
TNFSF14
TOX2
TRAIP
TRIM39
TRIM7
TSC22D3
TSPAN32
TTC24
TTTY10
TXK
VAMP1
VAX2
VCPIP1
VIPR2
WDR49
WNT1
XCL1
XCL2
YPEL5
ZAP70
ZBED2
ZBP1
ZBTB12
ZBTB45

ZDHHC18
ZFP57
ZIK1
ZNF331
ZNF416
ZNF548
ZNF750
ZNF786
ZNF80
ZNF831
ZNF876P
ZNF890P

Supplementary data of Fig. 3c: Gene lists from venn diagram

Genes for Venn Diagram

EpCAM	TROP-2	SP	EpCAM AND TROP-2	EpCAM AND SP	SP AND TROP-2	EpCAM AND SP AND TROP-2
ACTN4	PELI2	AACS	C9orf152	ZNF646	MITF	C5orf42
AHDC1	SCAF8	TFIP11	FARP1	KBTBD8	BTN2A1	CTD-2636A23.2
ATP13A1	UBB	AP000295.9	C10orf90	RP11-174G6.5	USP47	ABCA3
HSPD1P5	CYP4F23P	ERCC5	RALYL	FGFR1	AATK	VEZF1P1
COL4A5	CEACAM6	ZNF785	TACC2	MED13L	AC018633.4	CEP250
SLC4A8	RPL4P6	RP11-439L18.2	GDF15	C10orf12	UTRN	SAMD4A
NUDT17	SPA17P1	CRTAM	LYG1	HS3ST1	RELT	BTG2
RP11-408H1.3	KIAA1009	RNVU1-14	SH3BP4	RP5-1025A1.2	CAPN10-AS1	ITGA5
CXADR	C15orf41	ZNF131	ARRDC2	PLEKHG7	CTD-2020K17.1	MUM1L1
C11orf93	LHFPL2	CCDC88B	EPN2	AL357673.1	KPNA2	EDN1
PGBD5	HES1	AC007285.6	AK7	TJAP1	GTPBP1	HSPB1
CCDC57	CLDN16	CYTH1	NINL	TMEM156	PRDM2	INPP5E
A2ML1	GDPD5	SEMA4D	CAPS	LA16c-380H5.5	FAM63B	PFKP
CEP170	NEIL1	RP11-360L9.8	LINC00693	RP11-351J23.1	HMGA1P2	PBX4
RIBC1	VWA5A	HIST1H4E	TEAD1	BARX2	RNPS1P1	AEN
PLEKHG4	SERPINB9	RP11-417L14.1	HOOK3	POLG	RP11-48B3.3	CLK4
FOXA2	KNTC1	NR4A2	EIF4G3	POU6F1	RP5-1085F17.3	CXCL3
LCOR	HCG21	ZFYVE1	CRHR1-IT1	SPRY2	PPP2R3B	GALNT3
FRMD5	PRDM16	MTMR1	SEL1L3	ANKLE2	ULK4	CHD8
KIAA0100	USP3-AS1	LAX1	WHSC1	GPR132	SLC6A6	RBM15
SORT1	FAM110C	RPS6KA5	RNF223	RP4-761J14.8	RP5-940J5.6	AHSA2
RP11-680E19.1	RPL31P47	RP11-493K19.3	SPIRE2	HNRNPA0	ATAD3B	TMC4
SH2B2	CAPN1	MAPKAPK2	ARHGAP39	ACAP3	ULBP3	FRAS1
SBNO2	GRIK2	ATG2B	GATA6	SPAG9	EIF4A3	DCAF5
RSPH4A	RP11-73B2.6	ELOVL5	SYT13	USP9Y	FAM189B	MMP7
RICTOR	DOHH	PTPN22	TRIM2	MIR17HG	CENPJ	EP300
C19orf55	RP11-4K3__A.5	ZNF66	GPN2	DAGLB	RP11-93B14.5	HIC2
ANXA8L2	IGFL4	ZNF623	TRAPPC9	DGKQ	NFRKB	KIAA0895L
STX19	GALNT18	AC006129.2	LINC00605	YJEFN3	FANCA	CHORDC1
GLYATL1P4	CEP85	CDC14A	MEIS2	GNGT1	TUBA1A	DDX5
PBX1	ANXA2P2	BAZ1B	MCU	NPHP3	HSPA2	FNBP1L
KRT8P41	RTN4RL1	LMNB2	MYO15B	KRT86	PIGG	ZNF446
PTPRH	CTD-2666L21.1	CD160	ALCAM	CCR7	ENO2	ZSCAN9
RGSL1	GOLGA2B	FOXK2	HPCA	AGPAT6	CLK3	TRIM33
UNC5CL	OPA3	TAF5	THADA	ASH1L-AS1	TRIM56	PPP1R13B
FAM229A	ARHGEF16	ANLN	PDZD3	RP4-717I23.3	ARHGAP12	ERV3-1
ASXL2	MICALL1	DLX2	RP11-755F10.3	EIF5B	ZNF767	CCDC28B
RP11-1023L17.1	ZMIZ1-AS1	GPR55	MMP15	IL17RD	TEX10	RAB3IP
CYP3A5	ARHGAP42	RP11-536C5.7	DNAH11	CCDC117	SLC5A3	MET
S100A3	ZDHHC11B	TNFRSF9	DNAH12	UNC119B	SPATA2	THRAP3
SLC35F1	RP11-244H3.1	MIB2	HLA-J	UBN1	SNURF	TOB2
RN7SKP69	TMEM241	AP000253.1	PAQR8	SNHG15	MEGF6	TET3
FHDC1	CXorf23	TIAL1	STMND1	CTD-3137H5.1	CTD-2555K7.4	BBC3
AC114730.7	MTRNR2L8	MSH6	POF1B	MAML1	CASC7	SFTA2
UPK3B	LRP5L	AP4B1-AS1	GLIS2	CHML	DHX9P1	IGSF3
LRRC56	EPHA7	RNF4	ATF7	CTD-2006C1.2	ALOXE3	LRRN1
FHIT	MARVELD3	HDAC7	LRRC48	RP11-169K16.9	AC017099.3	EVC
RP11-19P22.8	RP11-864J10.4	MFSD11	FUT4	RPL32P3	TEP1	HERC2P2
TNNT2	LINC00887	CHMP1B	DHX16	WDR74	AP001062.7	RP11-215G15.5
C6orf52	MED25	TMEM120B	INPP5J	MCM7	SF3A1	ARL6IP1
KIAA0753	SDR16C5	FZD9	NEBL	OSBPL7	RASGEF1B	CHD1
LINC00174	JRK	CHFR	GPATCH2	ACKR6	ZNF317	TMEM184A
GK5	RP11-582J16.5	VIM-AS1	CBFA2T2	TRIM11	RP4-530I15.9	BZRAP1
RP4-655J12.4	OLR1	ZBTB11	GSTP1	S100A2	DDX3Y	SPPL2B
TTC28-AS1	TLR5	CNOT6LP1	NAALADL2	RHOT2	C2CD4D	ARL4A

ACLY	RP11-710F7.2	CDC42EP3	ARHGEF38	CTB-89H12.4	SYNCRIP	ITGA3
NPIPP1	RP11-383C5.7	FSIP1	LPIN3	CDK5RAP3	NRGN	HERC2
PCED1A	CEP164	RP4-800J21.3	RBPMS	KIAA1731	SLC6A8	SLC12A4
KRT18P12	RP11-18H7.1	RP1-68D18.4	RP11-37B2.1	LRRC8B	C16orf72	SLK
RP11-58E21.3	KIAA0368	CDK17	NMNAT2	GLIPR1L2	RSBN1	LUZP1
PP13439	LUZP2	SUZ12P	SORBS2	ZNF594	RBM5	POU3F1
ERC2	SLC7A11	RP11-30K9.6	RAB38	RP11-386G11.5	BAHD1	NASP
AC017076.5	MAN2C1	RASSF5	CAPN6	PRPF38AP2	HEY1	KRT18P16
ASB1	AC002429.5	ZBTB18	RP11-417F21.1	ZNF252P-AS1	U73166.2	CDK20
C22orf23	MAVS	ARIH1	CDSN	USP12	NPIPB4	COL9A2
C19orf44	PCYT1B	IL6R	PPM1H	CTD-3126B10.1	ABHD17B	ANXA3
RSPH3	B4GALT4	HIST1H2BJ	RP11-23P13.6	CTC-518B2.12	SNHG1	PPP1R15B
ZDHHC8P1	PDK4	AKNA	RALGPS1	RP11-214O1.2	MICAL1	TENM4
MARK2P8	MMP1	MIS18BP1	VWA2	SLCO5A1	NFKB1	VGLL3
TRMT11	CTC-529P8.1	EPHA4	SYTL4	DMWD	CAPN15	DCP1A
BEND5	SSBP2	MAFA	PACSIN3	AFTPH	CTB-118N6.2	HSPA1L
MMP12	PIFO	RP11-572M11.4	MUC5B	RAD54L2	C11orf84	C15orf52
LRRC36	AJAP1	LINC00943	NOXA1	KLF13	UBAP2	EGOT
GUCA2A	TRIP11	NOP58	C1orf198	NBPF14	CEP128	BCLAF1
FBXO15	SYP	PHF1	WDR91	RSF1	MCM3AP-AS1	FOXJ1
GAPDHP68	MMP11	ZNF432	TRIM45	GCN1L1	LBH	TRA2B
C14orf164	CTD-2517M14.5	LONRF2	ANKRD26	SPTSSB	CENPT	ABR
TRIM24	C9orf116	RP1-59D14.3	PDGFD	MMP10	PNP	STK17A
MIPEP	LYPD6B	DOM3Z	BEX2	ESR2	SRSF12	NCOR2
IFT74	CCDC162P	RP5-1073O3.7	RPS20P22	ZKSCAN2	ZNF554	C2CD3
RP11-484L7.1	ARHGAP32	C11orf68	CSPP1	SIK1	FBXL12	RIPK4
CNTROB	IP6K2	HCFC1	ZMYM5	ARID1A	SETD2	DUSP4
CTD-2005D20.1	CCDC74B	IL27RA	RP11-175P13.3	MGC20647	GPR3	HSP90AA4P
RP11-128A6.3	PCDHA5	GFI1	CSMD3	PVRL1	RGS16	RP11-285F7.2
DOCK1	RNF31	SCN11A	DLGAP1	MACF1	RP11-258C19.5	WHSC1L1
ZNF676	RP11-92C4.3	CDH24	LRBA	DDX47	7SK	CORO2A
LINC00704	PDLIM1	FAM126B	NTN4	SPATA17	EZH2	FBXL14
LRRC63	AC025335.1	E4F1	TMPRSS4	KLF16	ACRC	FBXO11
DTX2	EGFR	DDX24	BARD1	RP11-203J24.8	FOXC1	DAPK3
FLJ14082	RP11-64B16.4	TTC21B	ICA1L	HIST1H2AE	TMEM217	NRARP
MTND5P25	LRRC49	IL23R	HCG4P3	VAMP1	ZNF609	HSPD1
C9orf129	CPSF6	NRF1	AGER	AC004893.11	TMCC2	F2RL1
AC004941.5	C1QTNF6	UBQLN2	MAP4K3	USP49	LRRFIP1P1	B3GNT7
BPIFB1	ZNRF3	YWHAZ	IL20RA	FAM105B	RP11-1336O20.2	KLHL36
hsa-mir-8072	ZNF211	RP1-111C20.4	RP11-678G14.3	SOS2	SCNN1D	DNM1
TSIX	NISCH	KLRC1	STK3	GPATCH2L	EMP1	GCC2
LINC00320	C14orf79	TAGAP	C11orf49	SPICE1	PROSER1	OTUD4
PCDHB14	DBNDD1	IL1RL2	VEZT	CATSPERG	FAM222B	LLGL2
ZDHHC7	PRDM10	IDS	SLC17A4	MIR4453	FAM110A	RFX3
RP13-225O21.2	DUSP27	CDHR1	TPPP3	PAN2	UBR5	HSP90AB2P
SRRM1P2	MKL2	ZCCHC12	STRN3	RNF144A	CBLB	OTUD3
Z83851.1	PIWIL2	PRPF39	EPHB3	VTRNA2-1	MORC3	FBXL19-AS1
HSD17B3	DDX43	PSMD6-AS2	ZKSCAN1	MSX2	ZRANB1	BCL2
RP11-696L21.2	TNFRSF14	TBX21	LRIG1	IFIT2	C5orf49	SRSF11
POLL	HDAC11	MSNP1	RNF43	KSR1	DGKB	NFKB2
PRKD2	MAP3K4	RP11-78A19.3	GAL3ST1	ZXDA	NPIPA1	ARL13B
AKAP6	RBPMS-AS1	CCDC6	RAB25	FZD7	SPTBN5	TNFRSF10A
TTC39A	CDK2	TRGC1	FGFR4	SF3A2	OBSCN	IFT140
TRAPPC11	RP11-294O2.2	ZNF324	MIR4435-1HG	RAD52	PNRC1	KIAA1324
HIST1H4D	HTR1D	TTC13	CCDC14	HELZ2	CREBZF	MAFK
PDX1	BFSP1	YWHAH	GYLTL1B	H1FX-AS1	PPP1R12C	SON
PRICKLE4	ZNF532	RP11-146F11.1	PPP4R1	NT5DC3	SCEL	FAM76B
MYT1L	NEURL4	C17orf66	KCNQ1	C1orf52	CCNT2	MLF1
AF011889.5	RP11-61I13.3	RP11-46H11.12	MMP24	ODF2L	H2AFX	TRIM29
QRICH2	CLSPN	RP5-1129J21.3	CTTNBP2	RND3	CCDC130	DUSP5

KIAA1377	LRRIQ1	AC003075.4	TP53I3	ZNF653	PRR14	C12orf75
SFI1	ACOXL	ZNF384	RP11-553L6.5	PRPF38B	CEP95	RP4-647C14.3
TRPV4	ZMYM2	DSTYK	ARHGAP8	CCDC30	AC005154.6	PXDN
PLEKHG5	DNAH3	KLHL28	RERE	HNRNPH3	SUV420H2	CDS1
NCOR1	TET1	UBALD1	SLC22A5	ZNF292	DYRK1B	PPP1R9A
CRIPAK	CD47	IRF7	MYO1E	MED23	UBE2SP2	TOMM34
RP11-46B11.5	RP11-311D14.1	MAP1LC3B	CAPN8	SF1	AC004510.2	FKRP
CALN1	CAV2	KIT	ANXA2	TMEM67	RBM12	FUS
LRP2	AC073052.1	TMIGD2	RP11-345P4.9	EML2	KRT8P30	TOX3
SHB	ACTBL2	TARSL2	INPPL1	NCAPH	STX1A	RNF39
RP3-400N23.6	RP11-519G16.5	HYPK	PDZK1IP1	FOXP4	FOXO4	TSPYL4
EXTL3	LONRF1	RP11-944L7.4	AP1S3	LETM2	DIP2B	USP42
LIG1	SEC63P2	LINC00863	SOGA1	CNNM1	NBPF10	RGS4
RP11-482E14.1	RP11-512F24.1	YWHAZP2	OBSL1	DENND2C	HES6	WDR19
KANSL3	TTLL5	HOXA5	LINC00340	RP11-138A9.1	TBPL1	NIPBL
HDGFRP2	RP11-423O2.5	IL21R	SLC12A7	BCORL1	TDG	POGZ
PJA1	EIF3IP1	CCNK	FOXP2	SSH1	IFNG	RP11-737O24.3
ARSD	TFF1	DNMT3A	HNRNPD	LATS1	CNTRL	EFHC1
ZNF160	GJB4	BTBD9	EFNA5	MED1	GTF2IRD2	ZZEF1
PLXNB2	AP001258.4	NCKAP5L	ZDHHC1	DDX6	ZNF841	RP11-66N24.3
TTC22	AMER1	SLC7A6OS	ATP2C2	CAV1	HK2	BAMBI
STPG2	AGR2	IL4I1	SLC44A3	ZDHHC23	ASB6	IRF3
STXBP4	RP4-773N10.4	TRIM35	HDAC4	ANKRD29	EFNB2	PRRC2B
CATSPERB	CCDC13	CCL4	CTNNA1	TMEM131	NLGN1	RHBDF1
VTRNA1-1	TMPRSS11A	SMCHD1	STK36	RP11-496N12.9	RN7SL473P	C3orf52
IL4R	RP4-773A18.4	TOX	SH3TC2	H1FX	TEC	TTLL4
RP3-393E18.2	TBC1D19	RP4-742C19.12	KRTCAP3	RP11-83A24.1	SNHG10	MYO5C
RGS12	RP11-440L14.1	AC002055.4	WDFY3	WRN	KCNC4	LIF
EPS8L2	TVP23C	EDA2R	PYGB	WTAP	ACIN1	BACE2
RAPGEF5	MPZL2	FAM107B	SH3YL1	SPSB3	YLPM1	OXTR
MORF4L2-AS1	AC073218.2	PDE4D	AK8	CTD-2547E10.2	RP11-459E5.1	RP11-762H8.4
SULF1	MAST2	ITK	PTPRF	HIST4H4	CREBRF	INO80D
ILDR2	NBEAL1	ZNF551	IZUMO1	BTBD7	SMG1P1	KDM2A
SNORA76	GLIS3-AS1	GS1-72M22.1	DZIP1	SNORD104	C10orf111	H3F3B
DAGLA	STX18-AS1	HOTAIRM1	TIAM2	GATA6-AS1	MAPK15	KDM6A
LAMP2	AL020996.1	PHF12	FAM150A	CTB-31O20.2	RPGR	NCAM1
RP4-663N10.1	RP11-16E12.1	RP11-679C8.2	PALM3	GTF3C1	TLL1	EZR
RP4-694B14.5	ABHD4	BCL6	RP11-76E16.2	EPS15L1	FAM160B2	OFD1
GHRHR	CACNB3	ELL	ARMC9	HIST1H3A	TMEM2	FGFR3
FOXH1	DNAAF3	ACTRT3	GBP1	SCAF11	CDC5L	PI4KAP1
RGS17	MUC3A	EOMES	SLC25A27	RP11-465B22.3	TULP3	DYNC1H1
RP11-361K17.2	MUC16	WDR26	XIST	CTB-131B5.2	WAC	PDP1
SYCE1L	SOCS6	NONOP2	TMC5		ADCY9	C17orf85
WRAP53	C9orf37	MCTP2	RBBP8		ARHGAP27	WDR6
ATP8A2	TFCP2L1	SUPT16H	CAMSAP2		NSUN5P2	UBN2
PMS1	THBS3	ULBP1	ZNF433		ATP2B1	NFIL3
CNNM3	TTLL1	GPR18	MAPK13		FCHSD1	CAPN12
CNOT3	MFI2-AS1	RNPC3	SYT8		CSF2	VTCN1
GAK	ESYT3	RP11-192H23.7	FER			GLG1
AGBL2	SWT1	DPY19L2P2	PRKRIP1			CLDN6
AC107016.1	RCOR3	VCP	ST5			PKHD1
AC025442.3	RP11-39C10.1	TM4SF1-AS1	RP11-145A3.1			GLA
MYL6B	PCDHB11	RAVER1	NME5			HSPA8
FARP1-AS1	CST1	SRSF3	SMIM6			DIDO1
BSPRY	RBMS2	FSD1	RP11-860B13.1			RARB
FUT2	NPSR1-AS1	LRRFIP1	ILDR1			DHX15
CDC42EP1	MAPT	CNOT2	CASR			CNOT6
RP11-510M2.5	RCC2	PTTG1	RP11-350G8.5			ZBTB20
C3orf55	RP11-629G13.1	CTDP1	CRMP1			NEU1
SLC26A7	VILL	IL18RAP	LRP12			BRD8

C9orf3	CTF1	TNFRSF25	CSNK1G1	TPBG
KLHDC9	LINC00857	CWC25	KRT7	APOBEC3B
PPP2R3A	FAAH	PTGER4	SOX6	SLAIN1
PLEKHA6	ZBED3-AS1	ZIK1	RIC8B	MED12
CTD-2302E22.3	PKD1L2	CTD-2089O24.1	KRT18P28	SLC9A3
WNT7B	RP11-627G23.1	RNVU1-6	TRPM3	NKTR
PHLDB2	CHL1	CKS2	STK32B	TLE2
FLII	RSPH1	PRDM1	CDC42EP4	METTL12
GS1-124K5.11	AC019186.1	GADD45B	CABYR	REM2
CALCOCO1	PTK7	FMNL1	SMG7	hsa-mir-1199
KRT18P10	MS4A8	GLCCI1	SMIM5	FKBP4
CTTN	ZNF518A	CCR4	HNF1B	AHI1
LINC00472	TPH1	GNLY	PTGFRN	NFYA
RP11-325F22.5	SLC36A4	USP38	LURAP1L	FUBP1
AC092835.2	PARP6	RP11-727A23.7	LINC00479	DNAAF1
ZNF251	RP11-71N10.1	URB2	GTF2I	AHNAK
RP11-276H7.3	CRIM1	XCL1	SSPN	KRT15
AC011747.7	SH3GLB2	HERC1	CTNND1	HEATR5B
RP3-523E19.2	ADAMTS20	MYBL1	MBOAT4	DUSP18
RPTOR	RNFT2	HIST1H4B	TAF1C	KRT17
ADRA2C	MAP3K10	CTR9	ZNF135	AHR
ZPLD1	RIMKLB	CTD-2031P19.3	DLG5	PLEKHM1P
WWC2	TRAV39	RP3-395M20.12	VSIG2	CBX6
DNAH14	ARL6IP1P2	RP11-489E7.4	AC004540.5	ZNF711
CAPN3	SLC6A20	HNRNPA3P6	NSUN7	ASH1L
SMARCC2	CACNA1F	RNVU1-13	LRRC27	RBM38
CLHC1	ARHGAP19	ADRBK1	ARHGAP24	CAMSAP1
KCNAB3	KIAA1875	BPGM	ALMS1	ATP1A1
AMBRA1	C8orf34	CITED2	PWWP2B	RBM6
C7orf55-LUC7L2	IGSF5	KPNA4	ELMO3	SERTAD2
NUP214	IRG1	SIRT7	DOCK7	PLXNB3
MFSD6	SOAT1	INO80B	PLEKHA5	KLF4
B4GALNT4	ACHE	ATP11B	MMP19	HEXIM1
RP11-411K7.1	ZNF267	YWHAG	BRD3	PGM2L1
ZNF506	RP11-1O2.1	CCR6	KCNJ16	RBBP6
BCO2	CCDC8	GATA3	ETV1	AC008440.5
EIF3J-AS1	UBE2D1	LINC00441	FSD1L	TJP3
RP11-204N11.1	FAM120C	WBP2NL	SESTD1	HID1
AC002451.3	RP11-452K12.4	GFOD1	CCDC148	FXYD2
DSCAML1	ROCK1	AC058791.2	FTLP14	GABBR1
RP11-84C10.4	WDR31	BRPF1	AC083884.8	RIC3
GAN	ACYP1	HNRNPUP1	NOL10	TLE1P1
AL358781.1	RP1-130H16.16	GPR97	GULP1	PPL
TRIM31	ASXL3	ETV3	SAV1	LAMB3
HSPBAP1	FUT1	G3BP2	VGLL4	RRAD
CHEK1	B3GNT3	TLE3	YPEL2	FMN1
MBOAT1	SLC36A1	RAET1L	TMEM54	RP1-225E12.2
ZNF117	ICAM4	ITGAD	TMEM63A	RNF182
RP5-1158E12.3	KCNS1	EPC2	CCDC24	EXOC1
AC068538.2	HIST1H2BG	FAM117A	MOB3B	BRD4
FAM135B	RP11-1C1.4	SIK3-IT1	COG7	CLDN9
RP11-213G2.3	NME7	RP11-24N18.1	SLC44A4	RASSF4
KRTAP5-9	RTTN	PCDH20	SPIB	PLA2G6
ZNF670	LIMCH1	SPTY2D1	EPB41L4A	MTG1
HYI	JUNB	TYK2	MAATS1	PRSS22
FOLR1	EPHA6	NDRG1	RASSF7	SOX9
LRRC69	IFI30	PTOV1-AS1	DEPDC1B	WDR96
PRRG4	RP11-114L10.2	CDK13	ITIH5	RRP7B
PSPN	DBF4B	BCR	PACSIN2	PIEZO2
RP1-122O8.7	AL049829.1	ZNF132	IQCK	IER3

AEBP2	LRRC7	ZNF10	CTB-118N6.3	RP11-138I1.4
RP11-806O11.1	VWA7	ZNF786	C17orf67	TNRC18
CCDC13-AS1	PCDHB16	C10orf2	FAM201A	JUND
MRE11A	AB019439.68	RP3-337H4.9	VSTM2L	SVIL
GJB3	SPNS3	RASA2	VEPH1	CMYA5
C2orf81	SETDB2	C4orf32	ZNF219	TNF
RNF103	KLRAP1	DHX38	FAM83H-AS1	EEPD1
CTA-398F10.1	AC004381.6	AC098824.6	SPAG16	IL23A
PBRM1	KRTAP2-3	CD28	RP11-375N15.2	RP11-103H7.5
CC2D1A	CEACAM7	LINC00299	INTU	NSD1
RP4-742J24.2	WBSCR27	LRP8	RP11-297N6.4	ANO9
ANXA9	SERAC1	EML6	FAM150B	NEDD4L
RN7SL368P	DZIP3	TGFBRAP1	NPHP1	TCP1
ARNTL2	RP11-159D12.2	TMEM43	ANXA13	PAWR
C14orf105	SYNPR	MARK2	TMEM30B	AGO2
IQUB	CEP41	AMMECR1L	CAMSAP3	CREBBP
SIM1	AC090587.2	CEP85L	ATP11A	KLHL21
C2CD4C	ZC3H11A	SKI	EXOC6B	LDLR
SNAP25	RP11-706O15.1	IPCEF1	PLEKHB1	SCRN1
VANGL2	SRGAP3	RP3-486L4.4	EVC2	RBM26
CLDN23	DENND4C	ATXN7L3	GREB1	PROSER2
GLB1L2	MORN4	RP4-536B24.2	WDFY3-AS1	AC141586.5
MYH3	CSRP2	TRA2A	KIFAP3	KLF2
AC004837.5	ZNF488	TTI1	CNKSR3	GPATCH8
TMEM253	EIF4A2	DNAJC27	ZC3H3	HSPA8P7
RP1-40E16.12	GUSBP2	CCNE2	SEMA3E	TANC2
CYP51P1	MT-TT	PDE7A	LINC00511	CPD
KRT18P65	TECRL	LMBR1L	PCDHGA7	KCTD13
FGFR2	GTF2IRD1	CABIN1	MUC1	IL8
ITGAV	RP11-1143G9.2	RP13-820C6.2	C21orf88	DNAJB4
BAGE2	RP11-675F6.3	AC079305.10	C8orf4	EPS8L3
RP11-432F4.2	RP11-334E6.3	CD247	TNFSF15	FAM193A
RP5-1142A6.2	ST13P18	KIAA0922	RAP1GAP	AC093110.3
SHPRH	AC006486.1	SOCS1	SPOPL	TBC1D8
SIPA1L1	CTD-2008P7.9	LRMP	CCHCR1	SYDE2
AC016831.7	ING1	C22orf24	FGD4	UCA1
PAK3	AHCTF1P1	IST1	GSN-AS1	RP11-779O18.1
RP11-509J21.1	AP001816.1	ID2	MORN1	NFKBIZ
RP11-181K3.4	PCNXL2	TRIM28	ZMYND8	RP11-433O3.1
AL162759.1	SYNPR-AS1	ZNRD1-AS1	PIGR	OSBPL3
MAP7D2	EVA1C	DDX21	CDH2	SERTAD1
DDA1	DGCR2	TNIP1	EHMT2	PHC1P1
RP11-298J20.4	FAM25C	CELF2	DEGS2	NSMF
SCGB3A1	CLDN18	TOX2	CCDC102A	TMPRSS13
NSUN5P1	MTMR11	SH2D1B	EPHA5	CACYBPP2
RP11-54D18.2	ATP10B	AC079779.4	ERMP1	MIR3687
RP11-298I3.4	MTND4P11	NABP1	ANKS6	SPP1
AC009061.1	C17orf47	RUNX2	ATP2C1	LINC00842
DYNLRB2	PPWD1	ODC1	EPB41L1	CA9
INTS6P1	ST18	QRSL1P3	DLG3	RP11-727A23.5
AC004471.9	SPOCK1	PHTF1	ABCC4	BDP1
AC078941.1		TNFRSF18	USP54	RP11-373L24.1
FBXL2		TAF3	DNALI1	RAB11FIP4
ANXA5		TMEM243	POLA1	C6orf141
AC073410.1		CRIP1	ACCS	SQLE
MTX3		RP4-738P11.3	RP11-793H13.3	RP1-261G23.7
DUOX1		RNPS1	SLC27A1	REXO1
LYPD6		CCT6P2	KIAA1407	BCL7A
BX322557.10		CCL5	P2RY1	EME2
NEK11		ADAM8	KIAA1217	NPIPB5

SLC25A25	CCDC157	RAB27B	GABRE
AC002550.5	MIR4720	GSTO2	ANKRD17
GNL3L	ZCCHC2	INF2	ELF1
KB-1615E4.2	KBTBD2	DPCR1	38777
KRT8P46	SDE2	PLS1	TGIF2
GRB10	SUCO	CHST4	CD83
RP11-56G10.2	SLC7A5P1	SENP6	RP6-218J18.2
SEMA5B	PBXIP1	RIF1	RP1-67K17.3
ZDHHC8	ZNF615	S100A14	SAFB2
LAYN	ZNF746	MAML3	IL18
VWDE	RP11-247I13.11	SCGN	NCK2
AGAP8	FAM214B	ETV4	ITGA6
HOMER2	USP36	BEND7	BAG3
DSCAM	S100PBP	STXBP5	AP001615.9
SLX4	N4BP1	PKP4	USP11
AC114755.3	ZNF37BP	CYTH2	NOTCH2
MBD6	PRKCH	CPM	NEURL3
PSORS1C1	KIAA0226	BCAR1	CHTF18
NPC1L1	HINFP	ADAM10	KDM6B
AL009178.1	STAG3L2	RABGAP1	ATXN2
CIC	TTC14	C11orf35	RASSF9
TTC28	WDR37	SETD5	BMP2
NLRP2	MTCP1	TNKS	NET1
MOK	RAB9A	MIR4454	CLDN10
MIR616	ICOS	NR6A1	USP43
CTD-3092A11.1	AREL1	WDR60	NFAT5
ZNF300	KDM2B	USP2	TAF1D
RHOB	IGF2R	KLC3	GIGYF2
RP11-196H14.3	POM121C	UBD	TNFRSF19
OR2B11	SKIL	KRTAP5-AS1	MAB21L2
TNFAIP8	TOP2B	VPS13A	VPRBP
PKD1L1	NOD2	RAB36	IRS2
RBM47	HTR2A	CTA-293F17.1	RHOV
PNMAL1	ARMC5	PICK1	GPRIN2
ABCA17P	AMPD2	BEX4	PIM3
ZNF256	HAUS3	HYDIN	PTPN14
IKBKE	LPIN1	ZG16B	CUL3
BEX1	PITPNC1	ANKRD9	FAM160A2
CCDC169	MKRN1	ZBTB20-AS1	MUC20
CYP51A1	CCP110	RP11-334A14.2	ARHGAP26
TMPRSS11F	AC104134.2	DROSHA	ITGB8
MSH2	RP11-157G21.2	OSGIN2	CDK16
EFCAB6	SBNO1	RAP2B	NPNT
PHF14	AF127577.11	PVRL2	KRT8P33
AQP1	PRPF40B	ZNF629	IMPDH1P10
HEXIM2	RP3-508I15.18	KRBA2	CDKN2B
PCDHB3	MAP1LC3B2	KDM3B	WSB1
ZBTB20-AS2	B4GALT5	LIMK2	CDR2L
RP11-18B16.2	TTC4	INTS3	RP11-325F22.4
RP4-798A10.2	BACH2	ARHGEF40	CFLAR-AS1
PGC	SETD4	C7orf13	VEGFA
RP11-844P9.2	MAP2K3	FAM60A	SREBF2
RP11-434I12.2	SYT16	ZNF618	LAMA1
AMY2B	TRIM36	PKN2	CSRNP1
PLEKHS1	RP6-99M1.2	SH3RF2	RP11-796E2.4
TCTN2	HGS	PRKAA2	SETBP1
TYW1B	DDX20	SGPP2	ITGA2
RP11-265N7.1	NDEL1	MUC19	CTD-2228K2.7
RP5-997D24.3	RBAK	RP11-532F12.5	TEX26
Y_RNA	PIM2	ARHGEF37	MAST4

RGMB-AS1	KMT2C	KRT18P38	RP11-354M1.2
SEC14L4	CPNE7	DSC3	ZBED6
GMNN	ZNF235	SERTAD3	MYC
LINC00265	PDE4A	EBF4	MFI2
RP11-680F8.3	SATB1	C8orf47	SLC37A1
SEMA5A	SLC5A8	MYO3A	GGT6
DUSP16	XYLT1	WBP5	ZC3H12C
LRRC16A	RP11-597A11.4	NFIB	RP11-388M20.6
IFT57	BTAF1	KRTAP3-1	TMEM132A
WDR52	COLQ	SPNS2	HLA-V
CES4A	AGO3	PCDH19	BAIAP2L2
TDRD1	RYBP	CLINT1	RP11-405A12.1
RP13-516M14.4	KLRB1	FGF9	MAGI1
CSRNP2	ZBTB17	FOXA1	KNOP1
GATAD2B	CD3EAP	ERBB3	LAMA3
METTL3	NLRC3	DDIT3	ARID2
THRAP3P1	FNBP1	CYS1	SMG1
TAOK1	ZNF224	FLJ11235	SP4
VSIG10	IL2RG	RP11-728F11.3	KIF1B
RP11-173P15.5	AFF3	PRSS50	HUNK
USP44	ESM1	SLC6A19	GNA13
DPP10	KHNYN	NPEPL1	VPS37B
LHX9	RP11-325P15.1	TMEM198B	AMOTL2
TUBB4A	TSPAN32	MDK	TNFAIP3
PISD	VAMP2	SPTB	FOXO1
SEMA3C	ZAP70	CTD-2033D15.1	KIF5C
SCARNA17	HECA	ALOX12P2	NAA40
MNX1	AC108676.1	ANKRD50	DUSP10
DPCD	AC017104.6	C21orf62	TRIP10
RP11-390F4.6	MATK	PATZ1	NEK10
ZNF833P	CCDC59	KIAA1456	TSEN54
	NUDT4P1	CA2	ARGLU1
	ANXA1	PPM1L	RP11-1033A18.1
	RP11-427H3.2	RP11-1220K2.2	NCOA6
	APOBEC3D	FRK	KIAA0556
	AXIN1	PAPSS1	UPF1
	RP11-286O18.1	HIF1A	ZSWIM4
	MATR3	RP11-473M20.5	TARBP1
	TM9SF1	SLX4IP	FBXO30
	AC009495.2	PKMYT1	EFCAB4A
	CHMP4BP1	ADCY6	TRIM16
	ZNF764	C16orf46	RP1-43E13.2
	VCPIP1	TGFA	SLC2A1
	MIR181A1HG	CFTR	MT-RNR1
	FBXL6	ARHGAP44	EPCAM
	PTK2B	THOC6	RECQL5
	NUP210L	KLHL29	RP11-1007O24.3
	MSN	RHBDL2	TRIM47
	RHOF	RNF214	WEE1
	USP20	NIPA1	SMURF2
	RAB8B	RP11-328C8.4	ARHGEF5
	HSPB1P1	APCDD1	GPBP1
	PHLPP2	PRSS16	ZNF862
	ARHGAP17	FAM84A	SLC26A6
	NOM1	CNKSR1	FAM84B
	STAM	TMEM163	CCDC64B
	MECP2	FAM189A1	TULP2
	ZNF202	ZNF185	TNRC6B
	LINC00936	DOCK5	SRRM1
	RP11-159G9.5	IFT81	ZNF608

CD55	PLEKHA8	PABPN1
CMIP	PLLP	GBA2
LNPEP	PHF13	FBRS
CARD11	COBL	ECT2
PLEKHA8P1	AMOTL1	KIAA1109
PTGDR	KRT8P45	ZNF165
CDK12	NFKBIE	PPARD
DDX27	TTC6	HMGCR
LINC00484	BRPF3	KRT18P17
AKIRIN2	SOWAHC	MCF2L
CYSLTR2	FAM227A	PNISR
CTD-2368P22.1	CDK19	SCTR
RP11-727F15.9	PARP3	ZNF577
CLEC2B	NRBP2	NPAS2
CCDC137	CTD-3080P12.3	EGR4
MGAT5	SLC34A2	IGHMBP2
SLC2A3	CRTAC1	MAP3K14-AS1
MATN1-AS1	KLHL17	FAM73B
TNFRSF11B	CITED4	VIL1
RNF34	SSX2IP	KCNH3
MYH9	CGN	LMO7
NKRF	ARRDC1	HNRNPH1
CLK2	BCL11A	KDM5B
C12orf61	CBR3-AS1	SRCAP
FAM133B	RFX4	PPP1R13L
GNG10	LCN2	GOLGA7B
CTSW	WWP2	UBASH3B
NEFM	FZD1	PPP1R15A
NOP2	FBXL18	ZCCHC6
GPCPD1	ADAM22	AC004623.2
ANKRD28	F3	MDC1
KATNA1	C1orf186	GRAMD1B
STAT4	RP11-388M20.2	ANKRD18B
TSC22D3	RP11-103C16.2	FEM1C
CASP8	HKDC1	DYRK3
TAP1	HNRNPA1P33	TCP1P1
FCRL3	PARD3B	RCOR1
FLNC	TESC	MKNK2
ZRSR2	ANXA4	PCDP1
PLCH2	KCNJ15	RP11-317J10.2
ITSN2	PARD3	TRMT1
LINC00338	ONECUT2	IGF2BP2
FBRSL1	NRG1	RP11-356I2.4
PRDM4	SNHG14	URGCP
SLC1A5	GAB1	TRAF6
LPXN	TRNP1	KRT80
DLL1	RND1	PLD2
SMC4	SPINT2	DNAH7
DBF4	RASSF6	CSNK1E
PPTC7	FXYD3	TUBB2B
HOXA3	PRKCA	BBS9
RAB21	DNAH5	PLCH1
PDCD7	ATP8B1	FAM83B
FOXJ2	BHLHE40-AS1	NBPF18P
KLRC2	PDGFC	IFT172
ZFP36L2	ANXA11	FAM102A
FBXO33	TOB1	IRAK2
DYRK2	WDR66	CBX4
STAP1	PHF21A	AGRN
NKG7	CDK10	ZSCAN12P1

IL17A	RP11-867G2.8		GOLGB1
NOTCH2NL	NPHP3-AS1		ATP9A
CDKN2D	AC078883.4		CHD7
HIST3H2A	KLHL24		HNRNPA2B1
TNFRSF4	SLC39A6		GADD45A
C17orf107			TSC1
CDK6			AC004447.2
AP4B1			CTA-29F11.1
KLLN			MESDC1
HIST1H2BN			SBF1
TIGIT			RP11-473M20.7
KLRK1			AC093838.4
LYST			OCLN
AIM1			NELFA
KIF20B			MYSM1
P2RX5			MMP14
SMARCA5			RBM14
NCR3			EIF3A
GMIP			THSD7A
SYNRG			KIF12
ZBTB16			KLHL15
VIM			CXCL2
ARHGEF3			NBEA
B4GALT6			CLDN3
CSNK1G2			BTN2A2
CD7			MUC6
RHEBL1			ADAM28
NLRP7			CARD10
PDPK1			B4GALNT3
DENND4B			AFAP1
IL22			FCHO1
AE000661.37			TMEM136
STX11			ZFP36L1
PRKX			PAPD7
AC005253.2			CAPN2
RP13-1032I1.7			NKX3-1
RP4-778K6.3			CHD2
GNG4			ANKRD36C
CTD-2105E13.13			NR4A1
CD97			ARHGEF28
RP11-1094M14.4			TSC22D2
ITPRIPL1			C6orf222
MADD			NEO1
PCDHGB2			TMPRSS3
RNF168			FUT3
SMEK1			ZNF142
CSNK1D			ZHX2
CCNJL			KRT19P1
SNHG7			SMARCA4
AP000783.1			BAZ2B
RNU4ATAC			MCOLN3
ADRB2			ZBTB21
AC022182.3			NFE2L3
GABPB1			MPRIP-AS1
RP11-540A21.2			BOD1L1
CENPC			PRPF8
RP11-383J24.5			CTSE
GNL1			GLIS3
RIOK3			TNKS1BP1
RP11-431M7.3			TCERG1

TTC7A
KAT6A
H2AFZ
PNPLA8
RP2
KRI1
RNF138
SKIV2L
ZNF184
HEG1
KRT81
PRNP
RP11-1407O15.2
PSME4
HMGB2
MTMR9
CTC-308K20.1
HMGA2
ARIH2OS
XCL2
TOPORS
KCNJ14
ZC3H18
LINC00861
NUP210
ZNF831
EFCAB4B
XXbac-BPG299F13.17
SLC4A10
ACAP1
SAP30L-AS1
CXCR5
RIN1
ITGA4
SPINK2
IRF4
SLCO4A1
RGS2
AP5Z1
FASLG
MOB3A
ZBTB42
PTMA
PRDM11
SNHG3
HLA-F
IVNS1ABP
ZNF394
CLSTN1
ZNF701
DENND5A
NUP98
HIST1H3H
ZNF276
FTH1P4
RP11-58H15.1
RORA
IL2RA
TBCC
TBC1D13
UBE2O
ANKRD36
KLF5
DPH7
LAD1
EHD4
GABRP
PDE5A
TACSTD2
CPEB4
FAM179B
RP11-69M1.4
TCTEX1D4
THBS1
TUBA1C
ABL1
RAI2
KIF27
SRGAP1
TNFRSF21
UBE2H
RP11-344B2.2
KIAA1967
SMPD3
STIP1
C15orf39
HSP90AB1
PMAIP1
SEMA6D
SLC3A2
IER5
SYNE1
XXbac-BPG252P9.10
CMTM4
NCL
UVSSA
KRT8P36
ADCY10P1
WDR27
MACC1
C10orf118
ZBTB10
CEP192
CRAMP1L
KIAA0895
GUSBP11
ARHGAP5
EZR-AS1
MZF1
PHLDA2
WFDC2
GPR56
PAN3
REPS1
FBXW7
MED15
MTA1
ZBED4
SBK1
SETD1B

ARHGAP9	C16orf93
ZNF597	SLC25A36
LONRF3	PPP2CA
NCS1	RAB11FIP1
SNORD13	PHF15
TNFSF4	POLR2A
YWHAZP3	PPRC1
NOP56	HSPA8P8
LINC00528	LUC7L3
NUDT11	CAMTA2
TXK	HIP1R
CLIC3	FILIP1L
C17orf96	CA3
IL12RB2	LRRC1
NLRC5	ESRP1
GLTSCR1	KRT19
PIK3CG	AKAP8L
SNHG5	FLRT3
RP11-44M6.3	NR2C1
RP11-110I1.12	RP11-713P17.3
ZNF876P	VCAN
IRGM	ZDHHC21
AC064852.4	THAP9-AS1
SEMA6C	GLS
MBNL1	TGIF1
NUP160	HSPA7
IL2	RP3-395M20.8
ARRDC3	MAFG
ZNF844	SPAG1
SLC1A4	PIDD
AL353898.2	BICD2
IQGAP1	TM4SF1
CCDC65	SHANK2
HSPA5	JUP
RP11-474I11.7	SLC41A1
PUS7	MAMDC4
MIR142	DZIP1L
FGF2	C3orf58
EPG5	PAXBP1
DPH2	SETD1A
PPP4R1L	PURB
MICB	ZCCHC11
NCR1	RELB
DNAJC2	LAMB2
MTRF1L	KIF3A
CAPRIN2	EHD1
PCM1	MARCKSL1
GTF3C4	RAB11FIP5
FANCI	BRD2
SLC16A3	SLC7A1
ATP1B3-AS1	PTPRM
FGFR1OP2	KAAG1
RP11-247A12.2	CUL7
SNHG17	LAMC2
ETS1	SPSB1
CIRBP-AS1	RP11-224O19.2
C9orf131	SEMA4F
U2AF1L4	ABCA2
MIR155HG	APOBEC3B-AS1
RP11-57H12.3	SLC35F2

TCIRG1
CDK5R1
HSH2D
EID3
FCRL4
HOXA9
NPPC
TMEM201
DKK1
NRIP3
LRRN3
RP11-465L10.10
EFTUD1
ARID3B
MAP9
BRD7P3
RP5-1073O3.2
IL7R
SRSF2
PCDH9
HAUS5
CCDC88C
GGNBP2
CD96
DENND1C
RP11-432J9.5
EWSR1
ORAI2
INTS4
MST4
TBC1D10B
PPP1R16B
ZNF337
CTNNAL1
OXSR1
RPL41
SLC38A1
PHF20L1
HMMR
PPP2R2B
PKM
GBAP1
S1PR5
RP11-436I24.1
NOLC1
CCND2
ZBED2
ABCA7
SART3
APOBEC3G
C6orf25
GPRIN3
MYO1G
PGBD2
RP1-1J6.2
RALGAPA1
SIK3
CTC-548K16.5
MAK
TRGV9
RP11-256P1.1
AC024940.1
SNHG12
CFLAR
AP006621.8
RASSF8
HELZ
TRAK1
ZMIZ2
PTPRJ
ADM
RHPN2
TCF20
MAPK8IP3
ANKRD33B
ELMSAN1
MXD1
ASXL1
TUBB4B
NUAK2
PER1
RBM39
WDR90
KLC2
SH3RF1
WHAMM
CLDN1
RP6-145B8.3
SIRT1
ABCC1
FOSB
MED26
FAM46C
OVOL1
UPF3B
TPPP
AC011841.1
DDX39B
DEDD2
HMGCS1
SNRNP200
TLE4
NFKBIA
RP11-161H23.5
RP11-145F16.2
HNRNPLP2
ZNF821
RPL23AP79
MPRIP
ZFAND2A
FAM117B
UPF2
GSE1
KRT8P3
JMJD7-PLA2G4B
MIR24-2
CKMT2
NCOA7
ANKRD18A
ERRFI1

ZNF282
HIST2H2BF
37135
TCF7
RP11-275I14.4
FANCE
MAP4K4
DOCK3
ZNF436
KIF21B
RP11-229C3.2
RP11-338C15.5
ZNFX1
CD69
LTA
TRIM59
TSNAXIP1
MGEA5
CYP46A1
RRNAD1
VPS13C
CASP2
RANBP2
snoU13
C4orf26
RP11-545M17.1
TNFSF14
FBXO32
DDX3X
TRDC
POLR3D
AC009404.2
SYTL3
IL2RB
SNAP91
SLC52A2
DDX3YP3
ZNF600
TRAF3IP1
CTC1
HIPK1
STK17B
RP11-809N8.2
KCTD9P2
GPR61
SERHL
SPTY2D1-AS1
OFD1P17
ZNF622
RP11-799D4.4
ZNHIT6
HIST1H2BF

INADL
ENGASE
DENND6B
ADNP2
RBM25
UBE2FP1
ARHGEF19
TPR
MAP1S
BCL10
INO80
DTX3
AC006128.2
GSTM2
RP4-816N1.7
LDOC1
TNPO2
UGT8
COL27A1
SAMD4B
HNRNPU
PLAUR
ATXN2L
SLC4A4
SCAF4
MUM1
BHLHE41
CLASRP
MAP7D1
BRAF
FAM222A
SRRM2
AAK1
CYR61
DTNA
RP11-496I9.1
MAFF
GIGYF1
PVRL4
MYBBP1A
TCOF1
RGL4
ZNF503
ARSJ
NR1D1
SYNGR1
AC009505.2
PARD6B
HIST2H2BE
SLC38A11
PPM1D
TP53BP2
ZBTB40
MEDAG
CHST3
SPC24
MAP3K9
ABLIM1
NCR3LG1
SLC19A2

PVR
B3GNT2
SAFB
RFX2
MLLT4
TMTC2
MYADM
ANKZF1
DUSP8
L3MBTL1
LBR
PLXNA3
RLF
MLXIP
BAIAP2L1
B3GNT5
MAP4
C1orf106
OTUD1
HIVEP1
ATRX
TNIK
SOX7
IER2
NMB
CTD-2369P2.8
ZSCAN18
TGFB2
KMT2E
TRAF3IP2
WNK2
HSPB8
EHF
CTNND2
NUFIP2
TAF4B
RP11-530C5.1
SLTM
SLC28A3
RP11-498C9.12
TRIO
MEX3B
TC2N
RP11-617D20.1
TICAM1
RDH13
RP4-541C22.5
KCNF1
ZBTB34
FOXQ1
SYNE2
POLG2
DSP
TUG1
IRF1
MIR3188
IRF2BP2
SOX4
DNAJB1
ALPK3

FLVCR1
RALGAPA1P
PROM2
BIK
SOGA2
RC3H1
AC015971.2
PKD1
ATAD2B
SFPQ
KIAA1522
AKAP7
CROCC
CLDN4
TOP3B
RP11-554I8.2
ARL14
FAM46B
SNIP1
HLA-H
KIAA1683
EPHA2
MAML2
CLCN6
NFATC1
SAMD12
SNRNP70
RP11-203M5.8
PLK3
YOD1
SMAD3
KDM5C
ATF3
PLP2
ANKRD10
SLC16A7
ZNF43
ITGB4
CCDC141
DVL2
RBM33
BTG1
RP3-510L9.1
CNOT4
DUOXA2
FLYWCH1
THOC2
MYO6
DNAJA4
RP11-745A24.1
RAP1GAP2
TRPV6
RP11-708J19.1
ARL5B
RBM24
GRB7
GON4L
SWAP70
C1orf63
PM20D2

HBEGF
RP1-313I6.12
ZNF574
RASAL1
CSF1
CEP170B
TIPARP
CRYAB
PTHLH
MAP3K14
USP24
NUP153
CCSER1
SLCO3A1
DUSP2
ZNF692
SEC31B
SUPT5H
PPAP2C
ELF3
SRRM1P3
MYEF2
SLC4A3
BCL9
MAGI3
ZBTB2
GALNT5
PLXNB1
CHD3
SFSWAP
REC8
CTSV
DUOX2
TSC2
NRIP1
IFFO2
CPSF7
RP11-61J19.4
TBC1D22B
ARID5B
TSGA10
SPTAN1
ARL4C
SCD5
RP11-770G2.5
NCEH1
DSG2
JAG1
DCUN1D3
IRF6
HSPA4
CCRN4L
NEAT1
PRKCD
DSC2
ATP13A4
MYO10
ZNF407
TTC18
BCL9L

SUN1
RBM19
RUNX1
HSPA4L
ENAH
LIPH
FOXO6
RASEF
EGR1
SLC12A2
ESRRG
RIPK2
HDAC9
PTGFR
APLP1
GRHL2
STOX2
DNAH1
CRY1
SELE
KIAA1549L
ABCB1
FAM171A1
NAA16
HECTD4
ELOVL7
PIM1
USP9X
ZBTB43
PCLO
KLF6
USPL1
RP11-1149O23.3
ATHL1
TMEM171
ZC3HAV1
ANKRD42
PDE9A
LSMEM1
EPS8L1
KCNQ1OT1
SERPINB2
CCNL2
NPTX2
SNAPC4
CLCN2
SLC9A7
ZFHX4
LRIG3
ABTB2
ZNF236
TSPYL2
TUFT1
MYOF
ZC3H12A
LUC7L
C2CD4B
MAP3K8
FA2H
RP11-463O12.3

DDX26B
EGR2
ZNF281
RAD51-AS1
WWC1
PLEKHG3
PRORSD1P
SMOX
UNC13D
SMAD7
ZNF516
HSPA8P1
ITGB6
HIST1H2BB
CDC42BPG
RNF19B
GOLGA8A
RP11-732M18.3
RGL3
ABCC3
RP11-849H4.4
PIP5K1A
CSF3
REV3L
EXOC8
KIAA1244
RP11-152K4.2
HSPA6
C11orf95
SLC38A2
SYNJ2
SLC25A29
CD2AP
CCL20
FAM83E
SPHK1
ATN1
ADAM9
RP1-102K2.6
DNAJA1
HSF4
GPR176
ZNF296
TNK2
ZSWIM8
DENND4A
ANKRD11
LRRN4
NICN1
HLA-K
DUSP1
SEZ6L2
NFKBID
OR2I1P
H3F3C
PLEKHA7
CNNM4
SIPA1L3
SPEN
ENTHD2

GPRC5A
GBP2
ZNF217
AC034220.3
AGPAT4
KANSL1
FAM53C
RP4-816N1.6
GALNT12
FLNB
MFSD10
CRTC2
ZBTB7A
PRRC2C
STK33
ARHGEF18
HSPA1A
PFKFB3
EREG
DIRAS2
MCAM
CDH6
MIR3648
SLC5A9
ZSWIM6
RP3-416H24.1
AC016629.8
SLC52A3
SFN
PPP1R10
TRIB1
TJP1
LCA5
LENG8
RALGDS
ASPHD1
PER2
SLC26A11
SETD5-AS1
DFNB31
NXF1
DNAJA1P3
SLC4A7
PPP1R26
SEMA3B
CCDC40
ADCY8
PTCH1
S100A6
TP53BP1
RP11-611E13.2
TFRC
AC139100.2
DNAJB6
EPC1
DHX34
TES
BCOR
PELI1
ILF3

MLLT3
HSPA8P9
LARP6
RP11-98I9.4
SLC35F3
DCDC2
JOSD1
KAT2A
DYNC2H1
AKAP17A
LEMD3
KLHL25
CHKA
RP11-153M3.1
SLC35E2B
PIEZO1
PKD1P6
RP11-611L7.1
MAP3K1
MST1L
MB21D1
ATG16L2
39873
MAP4K5
LAMA5
TNFRSF12A
DIS3L2
EGR3
FREM2
IFRD1
KCTD9
HSPA8P5
MCL1
S100A11
LAMC1
DOT1L
NEB
EIF2AK3
RPRD2
CHMP4B
AC066593.1
TMPRSS2
IL6
RP11-73E17.2
CCDC93
CTGF
ENKD1
AC008937.2
PTBP1
BRD1
LPAR2
KRTAP1-1
ZNF335
LMTK2
ATF7IP
SEMA6A
SNORD12C
FOXK1
RP11-779O18.2
TRPM4

ZCCHC14
ISYNA1
CREB5
MLK4
HSP90AA1
ZNF316
RNF213
RP4-781K5.2
DHX9
CLK1
WDR47
DNAJC6
DALRD3
THAP2
HSPD1P6
JAG2
KIFC3
STX3
PTPRK
MST1R
MFSD6L
CLUHP3
PHRF1
CACNA1D
VEZF1
ACSS1
FOSL2
OGT
MRPL18
MSL2
RNF207
EPPK1
RHPN1
CCDC84
MYLIP
PHIP
RP11-676M6.1
LRIF1
NBEAL2
SCAPER
HSPB1P2
TMEM158
AC005785.2
TSPAN15
NPHP4
SFRP5
IKZF2
TNC
MYH14
RP1-163G9.2
PILRB
TUBB2A
PAPD5
SACS
WWTR1-AS1
SERPINH1
UBE2S
SH2D3A
TLE1
FAM83G

PTPRD
CDYL2
PLK2
SGK223
KIAA0247
SMURF1
ONECUT1
CCNL1
BCL2L11
PRSS12
RP11-54F2.1
INHBA
KLF7
AHSA1
DMKN
CPSF1
USP31
RAI1
LRRC24
HIVEP2
KCNH8
MYRF
CPEB2
BPTF
NPEPPS
RP11-75N4.2
MIR3654
MLLT6
PIWIL4
JMJD6
BX470102.3
WIPF2
AL358113.1
FAM60CP
PCF11
IQCA1
SLC7A5
MED17
RP4-734G22.3
PIGA
RREB1
PABPC1L
FNBP4
DNMBP
CBL
STXBP6
CTD-3149D2.4
ANXA8L1
SLC35E4
MGA
EIF4A1
AP1G2
JMY
ADORA1
PLEKHN1
KDM4C
DAB2IP
ZFC3H1
SDK1
RP11-802E16.3

HNRNPDL
FAM46A
HOMER1
KIFC2
CDH1
FOS
CAD
BAZ2A
FADS3
HOOK2
PDZD8
ABCC10
TFAP2C
HNRNPM
TET2
PLEKHM1
MSX1
WNT10A
AKAP9
SLC20A1
CX3CL1
ARAP2
SF3B1
ICAM1
SIX4
ABHD3
JARID2
USP34
SHISA9
SPINT1
PAQR5
SHROOM3
AC116366.6
MICAL3
SLC9A1
MSI2
PRR7
PLEKHG6
PLEC
LINC00271
AC093673.5
RP11-510N19.5
WNT2B
EDC4
ASNS
MPZL3
UBAP2L
CTD-2165H16.4
CASZ1
CDKN2AIP
CXCL1
BCAS1
RP11-144L1.8
EXD3
SGMS2
MON2
CNTNAP2
TNFRSF10B
RP11-286E11.1
CHD4

BICC1
RASD1
TRRAP
TRAF1
DBN1
SLC45A4
MIR600HG
ADAP1
SLFN13
RP11-473M20.16
MAP2
PROM1
CTB-59C6.3
FLRT2
DST
KRT8P12
ATP1A1OS
TTLL3
KMT2A
ST14
CYLD
RHBDF2
KIAA1468
JMJD1C
XXbac-BPG181M17.6
PRRC2A
STK35
S100A10
NOS1AP
FAM189A2
SAP30BP
RUSC2
BAZ1A
RRP12
ABHD11
ZCCHC8
ARHGEF1
CDKN1C
HIN1L
HSPH1
RP11-96K19.2
ARID4A
RP11-64B16.2
OTUD7B
KIF13B
SUGP2
ANKRD12
PI4KAP2
DDX51
GBF1
VCL
TTYH1
IRGQ
UBQLN1
RAVER2
SMC5
FAM211B
POU2F3
RARG
BACH1

ARC
LINC00671
CUL9
C11orf63
LINC00152
REL
SLC29A2
MPP3
TTN
GLRB
RP5-821D11.7
YBX3P1
AKAP13
WWTR1
ALS2CL
C6orf132
TNFAIP2
RASAL2
KLK10
RP11-66N24.4
AL136376.1
PTBP2
TCF7L2
NEDD9
ME3
HSPA1B
SMPDL3B
CACYBP
FAM221A
CXCL5
GPD2
AP1M2
MMP25
SCNN1A
IDUA
ZMIZ1
CHERP
FSTL3
ZNF462
HSP90AB3P
CDC42BPB
CXCL6
SGSM2
CAPN10
FOXP1
CUEDC1
SLC5A1
EPHB6
TJP2
SCRIB
RSRC2
PAPLN
SLC9A8
CDCP1
EDN2
YBX3
SHISA2
ITPKC
MALAT1
KSR2

ENC1
POMT2
ACD
SPATA13
CEP152
SREK1
TUBGCP6
ADAMTS10
GAREM
PRDM15
RP11-638I2.6
PRSS8
GPRC5B
NUMA1
VTRNA1-3
CD24P4
SUPT6H
ARID4B
GKAP1
FAM193B
AC012487.2
KDM5D
RNF19A
ZNF326
RP11-1100L3.8
AGAP1
ANKRD1
FREM1
DDR1
ZNF592
PMEPA1
TNFSF9
STX16
THSD4
ZNF266
LMNA
PRICKLE1
PEG10
HES4
KCNK5
FOXO3
ZBTB5
B3GALNT2
SRSF5
ZXDB
37681
RP5-1142A6.9
PRR26
TTN-AS1
MCF2L2
KLK11
KIAA0754
MFHAS1
MEX3C
CTC-250I14.6
HMGA1
TFPI2
YTHDC1
ERN1
JUN

UBE2SP1
KDM3A
AZI1
AC002117.1
MLPH
C1orf116
POM121
STRN
PNN
FAM81A
MTMR3
TRIM25
SESN2
HNRNPL
RP11-552F3.9
CLCF1
FGD6
HSPG2
FLNA
NOTCH1
SULT1C4
PTPN13
FAM160A1
DAPK1
LRRC14
RP11-349A22.5
TNFRSF10D
FOSL1
NPC1
USH1C
SRF
MDH1B
AC005540.3
LRIG2
SIN3B
AC004510.3
CLIC6
RP11-69I8.3
WAPAL
KAT6B
BTG3
NUSAP1
HSPD1P1
PNPLA2
PCNT
MDM4
CCDC9
VPS13B
MAMLD1
FRMD4B
UBC
N4BP3
RANGAP1
PUM1
ARNT2
ITPR3
GOLGA8B
BIRC3
TOP1
SPEF2

RPS27
FAM166A

Supplementary data of Fig. 3d: gene list used in clustering Fig. 3d

Clustering of cell specific genes

KRT19	HPC marker
KRT8	HPC marker
CLDN4	HPC marker
KRT19	HPC marker
KRT17	HPC marker
TACSTD2	HPC marker
EPCAM	HPC marker
ITGB1	HPC marker
SOX9	HPC marker
DDR1	HPC marker
PROM1	HPC marker
ID4	HPC marker
TPBG	HPC marker
ABCC4	HPC marker
ALDH1A2	HPC marker
HNF1B	HPC marker
CYP3A4	Hepatocyte marker
CYP2E1	Hepatocyte marker
ALB	Hepatocyte marker
TTR	Hepatocyte marker
APOC1	Hepatocyte marker
B2M	Hepatocyte marker
CYP2C9	Hepatocyte marker
CYP2C19	Hepatocyte marker
UGT2B7	Hepatocyte marker
ABCB1	Hepatocyte marker
ABCC2	Hepatocyte marker
ABCG2	Hepatocyte marker
CYP1A1	Hepatocyte marker
CYP1A2	Hepatocyte marker
HNF4A	Hepatocyte marker
HMGCS2	Hepatocyte marker
SLC2A2	Hepatocyte marker
VIM	Hepatic stellate cell marker
PDGFRB	Hepatic stellate cell marker
APOB	Hepatic stellate cell marker
PDGFD	Hepatic stellate cell marker
ACTA2	Hepatic stellate cell marker
ACTG2	Hepatic stellate cell marker
COL1A1	Hepatic stellate cell marker
COL1A2	Hepatic stellate cell marker
COL3A1	Hepatic stellate cell marker
COL4A1	Hepatic stellate cell marker
LOX	Hepatic stellate cell marker
LOXL1	Hepatic stellate cell marker
LOXL2	Hepatic stellate cell marker
NTM	Hepatic stellate cell marker
LYVE1	Endothelial cell marker
CLEC4G	Immune cell marker
FCN2	Immune cell marker
NPL	Immune cell marker
EMR1	Immune cell marker
CD68	Immune cell marker
PTPRC	Immune cell marker
CD34	Immune cell marker
CD59	Immune cell marker
THY1	Immune cell marker
CD38	Immune cell marker

Supplementary data of Fig. 4a: IPA pathway analysis results Fig. 4a

IPA ingenuity analysis: data used for pathway analysis and upstream regulators

Genes enriched in EpCAM

Fold change vs liver	ID	Symbol	Entrez Gene Name
1,158	ENSG00000107331	ABCA2	ATP binding cassette subfamily A member 2
1,918	ENSG00000085563	ABCB1	ATP binding cassette subfamily B member 1
1,491	ENSG00000103222	ABCC1	ATP binding cassette subfamily C member 1
1,482	ENSG00000124574	ABCC10	ATP binding cassette subfamily C member 10
1,661	ENSG00000108846	ABCC3	ATP binding cassette subfamily C member 3
1,237	ENSG00000125257	ABCC4	ATP binding cassette subfamily C member 4
1,23	ENSG00000097007	ABL1	ABL proto-oncogene 1, non-receptor tyrosine kinase
1,172	ENSG00000099204	ABLIM1	actin binding LIM protein 1
1,051	ENSG00000159842	ABR	active BCR-related
1,262	ENSG00000087085	ACHE	acetylcholinesterase (Cartwright blood group)
1,335	ENSG00000154930	ACSS1	acyl-CoA synthetase short-chain family member 1
1,062	ENSG00000119640	ACYP1	acylphosphatase 1
1,184	ENSG00000137845	ADAM10	ADAM metallopeptidase domain 10
1,088	ENSG00000042980	ADAM28	ADAM metallopeptidase domain 28
1,216	ENSG00000151651	ADAM8	ADAM metallopeptidase domain 8
1,702	ENSG00000168615	ADAM9	ADAM metallopeptidase domain 9
1,689	ENSG00000105963	ADAP1	ArfGAP with dual PH domains 1
1,433	ENSG00000174233	ADCY6	adenylate cyclase 6
1,35	ENSG00000155897	ADCY8	adenylate cyclase 8
1,564	ENSG00000205336	ADGRG1	adhesion G protein-coupled receptor G1
1,794	ENSG00000148926	ADM	adrenomedullin
1,756	ENSG00000163485	ADORA1	adenosine A1 receptor
1,178	ENSG00000150594	ADRA2A	adrenoceptor alpha 2A
1,91	ENSG00000181026	AEN	apoptosis enhancing nuclease
1,306	ENSG00000196526	AFAP1	actin filament associated protein 1
2,08	ENSG00000130396	AFDN	afadin, adherens junction formation factor
1,662	ENSG00000157985	AGAP1	ArfGAP with GTPase domain, ankyrin repeat and PH domain 1
1,036	ENSG00000204149	AGAP6 (incl	ArfGAP with GTPase domain, ankyrin repeat and PH domain 5
1,005	ENSG00000026652	AGPAT4	1-acylglycerol-3-phosphate O-acyltransferase 4
1,883	ENSG00000106541	AGR2	anterior gradient 2, protein disulphide isomerase family member
1,029	ENSG00000173467	AGR3	anterior gradient 3, protein disulphide isomerase family member
1,211	ENSG00000135541	AHI1	Abelson helper integration site 1
1,057	ENSG00000106546	AHR	aryl hydrocarbon receptor
1,673	ENSG00000173209	AHSA2	activator of HSP90 ATPase homolog 2
1,213	ENSG00000196581	AJAP1	adherens junctions associated protein 1
1,22	ENSG00000140057	AK7	adenylate kinase 7
1,425	ENSG00000197976	AKAP17A	A-kinase anchoring protein 17A
2,002	ENSG00000118507	AKAP7	A-kinase anchoring protein 7
1,385	ENSG00000011243	AKAP8L	A-kinase anchoring protein 8 like
1,708	ENSG00000128918	ALDH1A2	aldehyde dehydrogenase 1 family member A2
2,099	ENSG00000196711	ALKAL1	ALK and LTK ligand 1
1,285	ENSG00000189292	ALKAL2	ALK and LTK ligand 2
1,324	ENSG00000262943	ALOX12P2	arachidonate 12-lipoxygenase pseudogene 2
1,503	ENSG00000178038	ALS2CL	ALS2 C-terminal like
2,674	ENSG00000114019	AMOTL2	angiomotin like 2
1,548	ENSG00000240038	AMY2B	amylase, alpha 2B (pancreatic)
1,242	ENSG00000254996	ANKHD1/AI	ankyrin repeat and KH domain containing 1
1,107	ENSG00000176915	ANKLE2	ankyrin repeat and LEM domain containing 2
1,163	ENSG00000088448	ANKRD10	ankyrin repeat domain 10
1,149	ENSG00000167522	ANKRD11	ankyrin repeat domain 11
2,027	ENSG00000180071	ANKRD18A	ankyrin repeat domain 18A
1,334	ENSG00000230453	ANKRD18B	ankyrin repeat domain 18B
2,001	ENSG00000164236	ANKRD33B	ankyrin repeat domain 33B
1,806	ENSG00000135976	ANKRD36	ankyrin repeat domain 36
1,508	ENSG00000196912	ANKRD36B	ankyrin repeat domain 36B
1,223	ENSG00000137494	ANKRD42	ankyrin repeat domain 42
1,052	ENSG00000163516	ANKZF1	ankyrin repeat and zinc finger domain containing 1
1,624	ENSG00000185101	ANO9	anoctamin 9
1,171	ENSG00000122359	ANXA11	annexin A11
2,045	ENSG00000104537	ANXA13	annexin A13
2,056	ENSG00000138772	ANXA3	annexin A3
1,66	ENSG00000196975	ANXA4	annexin A4
1,17	ENSG00000143412	ANXA9	annexin A9
2,077	ENSG00000213983	AP1G2	adaptor related protein complex 1 gamma 2 subunit
1,969	ENSG00000129354	AP1M2	adaptor related protein complex 1 mu 2 subunit
1,318	ENSG00000154856	APCDD1	APC down-regulated 1
1,61	ENSG00000105290	APLP1	amyloid beta precursor like protein 1
2,663	ENSG00000179750	APOBEC3B	apolipoprotein B mRNA editing enzyme catalytic subunit 3B
1,653	ENSG00000047365	ARAP2	ArfGAP with RhoGAP domain, ankyrin repeat and PH domain 2
4,291	ENSG00000198576	ARC	activity regulated cytoskeleton associated protein
3,318	ENSG00000109321	AREG	amphiregulin
1,344	ENSG00000134884	ARGLU1	arginine and glutamate rich 1
1,283	ENSG00000138639	ARHGAP24	Rho GTPase activating protein 24
1,263	ENSG00000145819	ARHGAP26	Rho GTPase activating protein 26
2,026	ENSG00000248405	ARHGAP8/F	Rho GTPase activating protein 8
1,565	ENSG00000130762	ARHGEF16	Rho guanine nucleotide exchange factor 16
1,058	ENSG00000142632	ARHGEF19	Rho guanine nucleotide exchange factor 19
1,454	ENSG00000236699	ARHGEF38	Rho guanine nucleotide exchange factor 38
1,406	ENSG00000165801	ARHGEF40	Rho guanine nucleotide exchange factor 40
1,171	ENSG00000050327	ARHGEF5	Rho guanine nucleotide exchange factor 5
1,588	ENSG00000150347	ARID5B	AT-rich interaction domain 5B
3,468	ENSG00000179674	ARL14	ADP ribosylation factor like GTPase 14
1,124	ENSG00000122644	ARL4A	ADP ribosylation factor like GTPase 4A
1,746	ENSG00000188042	ARL4C	ADP ribosylation factor like GTPase 4C
1,329	ENSG00000196503	ARL9	ADP ribosylation factor like GTPase 9
1,654	ENSG00000135931	ARMC9	armadillo repeat containing 9
1,8	ENSG00000172379	ARNT2	aryl hydrocarbon receptor nuclear translocator 2
1,146	ENSG00000029153	ARNTL2	aryl hydrocarbon receptor nuclear translocator like 2
1,388	ENSG00000250151	ARPC4-TTLL	ARPC4-TTLL3 readthrough
1,304	ENSG00000105643	ARRDC2	arrestin domain containing 2
1,719	ENSG00000180801	ARSJ	arylsulfatase family member J
1,337	ENSG00000065802	ASB1	ankyrin repeat and SOCS box containing 1
1,073	ENSG00000116539	ASH1L	ASH1 like histone lysine methyltransferase
1,023	ENSG00000070669	ASNS	asparagine synthetase (glutamine-hydrolyzing)
1,832	ENSG00000174939	ASPHD1	aspartate beta-hydroxylase domain containing 1
1,572	ENSG00000148219	ASTN2	astrotactin 2
4,237	ENSG00000162772	ATF3	activating transcription factor 3
1,063	ENSG00000068650	ATP11A	ATPase phospholipid transporting 11A
1,325	ENSG00000127249	ATP13A4	ATPase 13A4
1,789	ENSG00000163399	ATP1A1	ATPase Na+/K+ transporting subunit alpha 1
1,356	ENSG00000203865	ATP1A1-AS	ATP1A1 antisense RNA 1
1,914	ENSG00000248919	ATP5J2-PTC	ATP5J2-PTCD1 readthrough
1,256	ENSG00000054793	ATP9A	ATPase phospholipid transporting 9A (putative)
1,227	ENSG00000204842	ATXN2	ataxin 2
1,267	ENSG00000168488	ATXN2L	ataxin 2 like
1,377	ENSG00000184809	B3GALT5-AS	B3GALT5 antisense RNA 1
1,025	ENSG00000179913	B3GNT3	UDP-GlcNAc:betaGal beta-1,3-N-acetylglucosaminyltransferase 3
3,022	ENSG00000176597	B3GNT5	UDP-GlcNAc:betaGal beta-1,3-N-acetylglucosaminyltransferase 5
1,236	ENSG00000156966	B3GNT7	UDP-GlcNAc:betaGal beta-1,3-N-acetylglucosaminyltransferase 7
1,677	ENSG00000139044	B4GALNT3	beta-1,4-N-acetyl-galactosaminyltransferase 3
1,177	ENSG00000158470	B4GALT5	beta-1,4-galactosyltransferase 5
1,355	ENSG00000182240	BACE2	beta-site APP-cleaving enzyme 2
5,482	ENSG00000151929	BAG3	BCL2 associated athanogene 3
1,245	ENSG00000006453	BAIAP2L1	BAI1 associated protein 2 like 1
1,253	ENSG00000128298	BAIAP2L2	BAI1 associated protein 2 like 2
2,181	ENSG00000095739	BAMBI	BMP and activin membrane bound inhibitor
1,456	ENSG00000138376	BARD1	BRCA1 associated RING domain 1
1,273	ENSG00000043039	BARX2	BARX homeobox 2
1,716	ENSG00000198604	BAZ1A	bromodomain adjacent to zinc finger domain 1A
1,084	ENSG00000076108	BAZ2A	bromodomain adjacent to zinc finger domain 2A
1,101	ENSG00000123636	BAZ2B	bromodomain adjacent to zinc finger domain 2B
3,366	ENSG00000105327	BBC3	BCL2 binding component 3
1,531	ENSG00000050820	BCAR1	BCAR1, Cas family scaffolding protein
1,629	ENSG00000142867	BCL10	B-cell CLL/lymphoma 10
2,18	ENSG00000171791	BCL2	BCL2, apoptosis regulator
1,09	ENSG00000171552	BCL2L1	BCL2 like 1
1,328	ENSG00000153094	BCL2L11	BCL2 like 11

Genes enriched in Trop-2

Fold change vs liver	ID	Symbol	Entrez Gene Name
1,119	ENSG00000128274	A4GALT	alpha 1,4-galactosyltransferase (P blood group)
1,154	ENSG00000167972	ABCA3	ATP binding cassette subfamily A member 3
2,304	ENSG00000085563	ABCB1	ATP binding cassette subfamily B member 1
1,607	ENSG00000103222	ABCC1	ATP binding cassette subfamily C member 1
1,002	ENSG00000124574	ABCC10	ATP binding cassette subfamily C member 10
1,516	ENSG00000108846	ABCC3	ATP binding cassette subfamily C member 3
1,166	ENSG00000125257	ABCC4	ATP binding cassette subfamily C member 4
1,223	ENSG00000136754	ABI1	abl interactor 1
1,413	ENSG00000097007	ABL1	ABL proto-oncogene 1, non-receptor tyrosine kinase
1,505	ENSG00000099204	ABLIM1	actin binding LIM protein 1
1,124	ENSG00000159842	ABR	active BCR-related
2,516	ENSG00000166016	ABTB2	ankyrin repeat and BTB domain containing 2
1,1	ENSG00000182827	ACBD3	acyl-CoA binding domain containing 3
1,102	ENSG00000130234	ACE2	angiotensin I converting enzyme 2
1,97	ENSG00000087085	ACHE	acetylcholinesterase (Cartwright blood group)
1,539	ENSG00000213088	ACKR1	atypical chemokine receptor 1 (Duffy blood group)
1,143	ENSG00000154930	ACSS1	acyl-CoA synthetase short-chain family member 1
1,077	ENSG00000119640	ACYP1	acylphosphatase 1
1,299	ENSG00000137845	ADAM10	ADAM metallopeptidase domain 10
1,349	ENSG00000151651	ADAM8	ADAM metallopeptidase domain 8
2,097	ENSG00000168615	ADAM9	ADAM metallopeptidase domain 9
1,137	ENSG00000154734	ADAMTS1	ADAM metallopeptidase with thrombospondin type 1 motif 1
2,074	ENSG00000158859	ADAMTS4	ADAM metallopeptidase with thrombospondin type 1 motif 4
1,295	ENSG00000163638	ADAMTS9	ADAM metallopeptidase with thrombospondin type 1 motif 9
1,217	ENSG00000105963	ADAP1	ArfGAP with dual PH domains 1
1,347	ENSG00000174233	ADCY6	adenylate cyclase 6
1,192	ENSG00000155897	ADCY8	adenylate cyclase 8
1,046	ENSG00000153292	ADGRF1	adhesion G protein-coupled receptor F1
1,832	ENSG00000205336	ADGRG1	adhesion G protein-coupled receptor G1
2,228	ENSG00000148926	ADM	adrenomedullin
1,473	ENSG00000101544	ADNP2	ADNP homeobox 2
1,139	ENSG00000163485	ADORA1	adenosine A1 receptor
1,062	ENSG00000170425	ADORA2B	adenosine A2b receptor
1,142	ENSG00000150594	ADRA2A	adrenoceptor alpha 2A
1,153	ENSG00000184160	ADRA2C	adrenoceptor alpha 2C
1,073	ENSG00000169252	ADRB2	adrenoceptor beta 2
2,528	ENSG00000181026	AEN	apoptosis enhancing nuclease
1,27	ENSG00000196526	AFAP1	actin filament associated protein 1
1,931	ENSG00000130396	AFDN	afadin, adherens junction formation factor
1,147	ENSG00000198221	AFDN-AS1	AFDN antisense RNA 1 (head to head)
1,642	ENSG00000157985	AGAP1	ArfGAP with GTPase domain, ankyrin repeat and PH domain 1
1,198	ENSG00000204149	AGAP6 (includ	ArfGAP with GTPase domain, ankyrin repeat and PH domain 5
1,852	ENSG00000106541	AGR2	anterior gradient 2, protein disulphide isomerase family member
1,079	ENSG00000135541	AHI1	Abelson helper integration site 1
1,583	ENSG00000106546	AHR	aryl hydrocarbon receptor
1,096	ENSG00000100591	AHSA1	activator of HSP90 ATPase activity 1
1,301	ENSG00000173209	AHSA2	activator of HSP90 ATPase homolog 2
2,214	ENSG00000131016	AKAP12	A-kinase anchoring protein 12
1,32	ENSG00000170776	AKAP13	A-kinase anchoring protein 13
1,544	ENSG00000197976	AKAP17A	A-kinase anchoring protein 17A
1,539	ENSG00000241978	AKAP2	A-kinase anchoring protein 2
1,231	ENSG00000118507	AKAP7	A-kinase anchoring protein 7
1,718	ENSG00000011243	AKAP8L	A-kinase anchoring protein 8 like
2,009	ENSG00000128918	ALDH1A2	aldehyde dehydrogenase 1 family member A2
1,778	ENSG00000184254	ALDH1A3	aldehyde dehydrogenase 1 family member A3
1,509	ENSG00000196711	ALKAL1	ALK and LTK ligand 1
1,371	ENSG00000262943	ALOX12P2	arachidonate 12-lipoxygenase pseudogene 2
2,706	ENSG00000179148	ALOXE3	arachidonate lipoxygenase 3
1,948	ENSG00000178038	ALS2CL	ALS2 C-terminal like
1,056	ENSG00000144233	AMMECR1L	AMMECR1 like
1,468	ENSG00000166025	AMOTL1	angiomotin like 1
3,063	ENSG00000114019	AMOTL2	angiomotin like 2
1,138	ENSG00000240038	AMY2B	amylase, alpha 2B (pancreatic)
1,585	ENSG00000254996	ANKHD1/ANKI	ankyrin repeat and KH domain containing 1
1,745	ENSG00000176915	ANKLE2	ankyrin repeat and LEM domain containing 2
1,089	ENSG00000088448	ANKRD10	ankyrin repeat domain 10
1,115	ENSG00000167522	ANKRD11	ankyrin repeat domain 11
1,109	ENSG00000101745	ANKRD12	ankyrin repeat domain 12
1,666	ENSG00000180071	ANKRD18A	ankyrin repeat domain 18A
1,469	ENSG00000230453	ANKRD18B	ankyrin repeat domain 18B
2,306	ENSG00000164236	ANKRD33B	ankyrin repeat domain 33B
1,89	ENSG00000135976	ANKRD36	ankyrin repeat domain 36
1,567	ENSG00000196912	ANKRD36B	ankyrin repeat domain 36B
1,273	ENSG00000137494	ANKRD42	ankyrin repeat domain 42
1,519	ENSG00000185101	ANO9	anoctamin 9
1,301	ENSG00000122359	ANXA11	annexin A11
1,647	ENSG00000104537	ANXA13	annexin A13
1,372	ENSG00000182718	ANXA2	annexin A2
1,179	ENSG00000231991	ANXA2P2	annexin A2 pseudogene 2
2,171	ENSG00000138772	ANXA3	annexin A3
1,243	ENSG00000196975	ANXA4	annexin A4
1,513	ENSG00000164111	ANXA5	annexin A5
1,784	ENSG00000213983	AP1G2	adaptor related protein complex 1 gamma 2 subunit
1,776	ENSG00000129354	AP1M2	adaptor related protein complex 1 mu 2 subunit
1,411	ENSG00000152056	AP1S3	adaptor related protein complex 1 sigma 3 subunit
1,071	ENSG00000254470	AP5B1	adaptor related protein complex 5 beta 1 subunit
1,067	ENSG00000154856	APCDD1	APC down-regulated 1
1,048	ENSG00000105290	APLP1	amyloid beta precursor like protein 1
3,398	ENSG00000179750	APOBEC3B	apolipoprotein B mRNA editing enzyme catalytic subunit 3B
2,497	ENSG00000047365	ARAP2	ArfGAP with RhoGAP domain, ankyrin repeat and PH domain 2
5,201	ENSG00000198576	ARC	activity regulated cytoskeleton associated protein
3,854	ENSG00000109321	AREG	amphiregulin
1,395	ENSG00000134884	ARGLU1	arginine and glutamate rich 1
1,337	ENSG00000165322	ARHGAP12	Rho GTPase activating protein 12
1,09	ENSG00000138639	ARHGAP24	Rho GTPase activating protein 24
1,427	ENSG00000145819	ARHGAP26	Rho GTPase activating protein 26
1,583	ENSG00000165895	ARHGAP42	Rho GTPase activating protein 42
1,074	ENSG00000006740	ARHGAP44	Rho GTPase activating protein 44
1,104	ENSG00000100852	ARHGAP5	Rho GTPase activating protein 5
1,977	ENSG00000241484	ARHGAP8/PRR	Rho GTPase activating protein 8
1,165	ENSG00000130762	ARHGEF16	Rho guanine nucleotide exchange factor 16
1,013	ENSG00000142632	ARHGEF19	Rho guanine nucleotide exchange factor 19
1,07	ENSG00000183111	ARHGEF37	Rho guanine nucleotide exchange factor 37
1,665	ENSG00000236699	ARHGEF38	Rho guanine nucleotide exchange factor 38
1,067	ENSG00000165801	ARHGEF40	Rho guanine nucleotide exchange factor 40
1,382	ENSG00000050327	ARHGEF5	Rho guanine nucleotide exchange factor 5
1,429	ENSG00000179361	ARID3B	AT-rich interaction domain 3B
1,016	ENSG00000054267	ARID4B	AT-rich interaction domain 4B
2,05	ENSG00000150347	ARID5B	AT-rich interaction domain 5B
1,013	ENSG00000166233	ARIH1	ariadne RBR E3 ubiquitin protein ligase 1
4,065	ENSG00000179674	ARL14	ADP ribosylation factor like GTPase 14
1,345	ENSG00000122644	ARL4A	ADP ribosylation factor like GTPase 4A
1,828	ENSG00000188042	ARL4C	ADP ribosylation factor like GTPase 4C
1,238	ENSG00000165997	ARL5B	ADP ribosylation factor like GTPase 5B
2,031	ENSG00000172379	ARNT2	aryl hydrocarbon receptor nuclear translocator 2
1,608	ENSG00000029153	ARNTL2	aryl hydrocarbon receptor nuclear translocator like 2
1,321	ENSG00000250151	ARPC4-TTLL3	ARPC4-TTLL3 readthrough
1,323	ENSG00000105643	ARRDC2	arrestin domain containing 2
1,997	ENSG00000180801	ARSJ	arylsulfatase family member J
1,647	ENSG00000065802	ASB1	ankyrin repeat and SOCS box containing 1
1,062	ENSG00000116539	ASH1L	ASH1 like histone lysine methyltransferase
1,559	ENSG00000070669	ASNS	asparagine synthetase (glutamine-hydrolyzing)
1,92	ENSG00000174939	ASPHD1	aspartate beta-hydroxylase domain containing 1
1,238	ENSG00000148219	ASTN2	astrotactin 2
1,104	ENSG00000171456	ASXL1	additional sex combs like 1, transcriptional regulator
3,85	ENSG00000162772	ATF3	activating transcription factor 3
1,31	ENSG00000128272	ATF4	activating transcription factor 4

1,588	ENSG00000110987	BCL7A	BCL tumor suppressor 7A
1,303	ENSG00000236824	BCYRN1	brain cytoplasmic RNA 1
1,005	ENSG00000165626	BEND7	BEN domain containing 7
2,082	ENSG00000133169	BEX1	brain expressed X-linked 1
1,334	ENSG00000133134	BEX2	brain expressed X-linked 2
1,259	ENSG00000102409	BEX4	brain expressed X-linked 4
2,7	ENSG00000123095	BHLHE41	basic helix-loop-helix family member e41
1,796	ENSG00000122870	BICC1	BicC family RNA binding protein 1
2,292	ENSG00000162069	BICDL2	BICD family like cargo adaptor 2
2,871	ENSG00000100290	BIK	BCL2 interacting killer
2,942	ENSG00000023445	BIRC3	baculoviral IAP repeat containing 3
2,786	ENSG00000125845	BMP2	bone morphogenetic protein 2
1,121	ENSG00000204177	BMS1P5	BMS1, ribosome biogenesis factor pseudogene 5
1,223	ENSG00000157764	BRAF	B-Raf proto-oncogene, serine/threonine kinase
1,084	ENSG00000100425	BRD1	bromodomain containing 1
1,416	ENSG00000141867	BRD4	bromodomain containing 4
1,286	ENSG00000119411	BSPRY	B-box and SPRY domain containing
1,208	ENSG00000133639	BTG1	BTG anti-proliferation factor 1
2,808	ENSG00000159388	BTG2	BTG anti-proliferation factor 2
1,434	ENSG00000154640	BTG3	BTG anti-proliferation factor 3
1,04	ENSG00000176236	C10orf111	chromosome 10 open reading frame 111
1,109	ENSG00000149179	C11orf49	chromosome 11 open reading frame 49
1,601	ENSG00000109944	C11orf63	chromosome 11 open reading frame 63
1,103	ENSG00000205177	C11orf91	chromosome 11 open reading frame 91
1,638	ENSG00000235162	C12orf75	chromosome 12 open reading frame 75
1,051	ENSG00000100557	C14orf105	chromosome 14 open reading frame 105
1,33	ENSG00000140104	C14orf79	chromosome 14 open reading frame 79
1,048	ENSG00000186073	C15orf41	chromosome 15 open reading frame 41
2,305	ENSG00000188549	C15orf52	chromosome 15 open reading frame 52
1,434	ENSG00000167644	C19orf33	chromosome 19 open reading frame 33
1,041	ENSG00000105072	C19orf44	chromosome 19 open reading frame 44
2,491	ENSG00000163362	C1orf106	chromosome 1 open reading frame 106
2,18	ENSG00000182795	C1orf116	chromosome 1 open reading frame 116
1,35	ENSG00000119280	C1orf198	chromosome 1 open reading frame 198
1,155	ENSG00000221953	C1orf229	chromosome 1 open reading frame 229
1,646	ENSG00000131094	C1QL1	complement C1q like 1
1,969	ENSG00000205502	C2CD4B	C2 calcium dependent domain containing 4B
1,189	ENSG00000183186	C2CD4C	C2 calcium dependent domain containing 4C
1,235	ENSG00000225556	C2CD4D	C2 calcium dependent domain containing 4D
3,777	ENSG00000114529	C3orf52	chromosome 3 open reading frame 52
1,17	ENSG00000181744	C3orf58	chromosome 3 open reading frame 58
1,032	ENSG00000205129	C4orf47	chromosome 4 open reading frame 47
1,03	ENSG00000197603	C5orf42	chromosome 5 open reading frame 42
1,138	ENSG00000178776	C5orf46	chromosome 5 open reading frame 46
1,054	ENSG00000188112	C6orf132	chromosome 6 open reading frame 132
3,28	ENSG00000197261	C6orf141	chromosome 6 open reading frame 141
1,607	ENSG00000189325	C6orf222	chromosome 6 open reading frame 222
1,243	ENSG00000137434	C6orf52	chromosome 6 open reading frame 52
1,623	ENSG00000176907	C8orf4	chromosome 8 open reading frame 4
1,714	ENSG00000160345	C9orf116	chromosome 9 open reading frame 116
1,306	ENSG00000204352	C9orf129	chromosome 9 open reading frame 129
2,169	ENSG00000164879	CA3	carbonic anhydrase 3
2,058	ENSG00000107159	CA9	carbonic anhydrase 9
1,47	ENSG00000116161	CACYBP	calcyclin binding protein
1,096	ENSG00000084774	CAD	carbamoyl-phosphate synthetase 2, aspartate transcarbamylase, an
1,143	ENSG00000270419	CAHM	colon adenocarcinoma hypermethylated (non-protein coding)
1,504	ENSG00000130559	CAMSAP1	calmodulin regulated spectrin associated protein 1
1,258	ENSG00000076826	CAMSAP3	calmodulin regulated spectrin associated protein family member 3
1,167	ENSG00000108509	CAMTA2	calmodulin binding transcription activator 2
1,119	ENSG00000042493	CAPG	capping actin protein, gelsolin like
1,333	ENSG00000182472	CAPN12	calpain 12
1,114	ENSG00000162909	CAPN2	calpain 2
1,383	ENSG00000077274	CAPN6	calpain 6
1,669	ENSG00000105519	CAPS	calcyphosine
1,097	ENSG00000100065	CARD10	caspase recruitment domain family member 10
1,497	ENSG00000036828	CASR	calcium sensing receptor
2,287	ENSG00000130940	CASZ1	castor zinc finger 1
1,025	ENSG00000078699	CBFA2T2	CBFA2/RUNX1 translocation partner 2
1,631	ENSG00000141582	CBX4	chromobox 4
1,532	ENSG00000183741	CBX6	chromobox 6
1,04	ENSG00000158941	CCAR2	cell cycle and apoptosis regulator 2
1,226	ENSG00000135736	CCDC102A	coiled-coil domain containing 102A
1,377	ENSG00000167131	CCDC103	coiled-coil domain containing 103
1,38	ENSG00000151773	CCDC122	coiled-coil domain containing 122
1,171	ENSG00000175455	CCDC14	coiled-coil domain containing 14
1,84	ENSG00000163492	CCDC141	coiled-coil domain containing 141
1,126	ENSG00000153237	CCDC148	coiled-coil domain containing 148
1,05	ENSG00000165813	CCDC186	coiled-coil domain containing 186
1,547	ENSG00000196118	CCDC189	coiled-coil domain containing 189
1,99	ENSG00000159214	CCDC24	coiled-coil domain containing 24
1,329	ENSG00000160050	CCDC28B	coiled-coil domain containing 28B
1,595	ENSG00000152076	CCDC74B	coiled-coil domain containing 74B
1,122	ENSG00000105321	CCDC9	coiled-coil domain containing 9
2,586	ENSG00000108691	CCL2	C-C motif chemokine ligand 2
3,477	ENSG00000115009	CCL20	C-C motif chemokine ligand 20
1,446	ENSG00000151882	CCL28	C-C motif chemokine ligand 28
2,01	ENSG00000163660	CCNL1	cyclin L1
1,23	ENSG00000221978	CCNL2	cyclin L2
2,679	ENSG00000126353	CCR7	C-C motif chemokine receptor 7
1,124	ENSG00000117877	CD3EAP	CD3e molecule associated protein
2,615	ENSG00000112149	CD83	CD83 molecule
1,237	ENSG00000198752	CDC42BPB	CDC42 binding protein kinase beta
1,042	ENSG00000171219	CDC42BPG	CDC42 binding protein kinase gamma
1,138	ENSG00000128283	CDC42EP1	CDC42 effector protein 1
1,297	ENSG00000179604	CDC42EP4	CDC42 effector protein 4
2,241	ENSG00000163814	CDCP1	CUB domain containing protein 1
2,025	ENSG00000039068	CDH1	cadherin 1
2,265	ENSG00000113361	CDH6	cadherin 6
1,073	ENSG00000008128	CDK11A	cyclin dependent kinase 11A
1,243	ENSG00000156345	CDK20	cyclin dependent kinase 20
1,639	ENSG00000124762	CDKN1A	cyclin dependent kinase inhibitor 1A
1,556	ENSG00000129757	CDKN1C	cyclin dependent kinase inhibitor 1C
1,221	ENSG00000168564	CDKN2AIP	CDKN2A interacting protein
2,418	ENSG00000147883	CDKN2B	cyclin dependent kinase inhibitor 2B
1,001	ENSG00000109089	CDR2L	cerebellar degeneration related protein 2 like
1,655	ENSG00000163624	CDS1	CDP-diacylglycerol synthase 1
2,041	ENSG00000086548	CEACAM6	carcinoembryonic antigen related cell adhesion molecule 6
1,292	ENSG00000102901	CENPT	centromere protein T
1,28	ENSG00000141577	CEP131	centrosomal protein 131
1,026	ENSG00000121289	CEP89	centrosomal protein 89
2,026	ENSG00000163075	CFAP221	cilia and flagella associated protein 221
1,451	ENSG00000226312	CFLAR-AS1	CFLAR antisense RNA 1
2,852	ENSG00000001626	CFTR	cystic fibrosis transmembrane conductance regulator
1,256	ENSG00000143375	CGN	cingulin
1,051	ENSG00000159259	CHAF1B	chromatin assembly factor 1 subunit B
1,079	ENSG00000153922	CHD1	chromodomain helicase DNA binding protein 1
1,951	ENSG00000173575	CHD2	chromodomain helicase DNA binding protein 2
1,472	ENSG00000170004	CHD3	chromodomain helicase DNA binding protein 3
1,883	ENSG00000110721	CHKA	choline kinase alpha
1,207	ENSG00000203668	CHML	CHM like, Rab escort protein 2
1,447	ENSG00000101421	CHMP4B	charged multivesicular body protein 4B
1,867	ENSG00000110172	CHORDC1	cysteine and histidine rich domain containing 1
1,296	ENSG00000133019	CHRM3	cholinergic receptor muscarinic 3
1,101	ENSG00000122863	CHST3	carbohydrate sulfotransferase 3
1,372	ENSG00000140835	CHST4	carbohydrate sulfotransferase 4
1,066	ENSG00000127586	CHTF18	chromosome transmission fidelity factor 18
1,381	ENSG00000179862	CITED4	Cbp/p300 interacting transactivator with Glu/Asp rich carboxy-term
1,1	ENSG00000166165	CKB	creatine kinase B
1,044	ENSG00000223572	CKMT1A/CK	creatine kinase, mitochondrial 1B
1,875	ENSG00000131730	CKMT2	creatine kinase, mitochondrial 2
1,145	ENSG00000104859	CLASRP	CLK4 associating serine/arginine rich protein
3,396	ENSG00000175505	CLCF1	cardiotrophin like cytokine factor 1
1,115	ENSG00000114859	CLCN2	chloride voltage-gated channel 2
1,024	ENSG00000011021	CLCN6	chloride voltage-gated channel 6
1,827	ENSG00000163347	CLDN1	claudin 1

1,13	ENSG00000068650	ATP11A	ATPase phospholipid transporting 11A
1,287	ENSG00000133657	ATP13A3	ATPase 13A3
1,42	ENSG00000127249	ATP13A4	ATPase 13A4
1,66	ENSG00000163399	ATP1A1	ATPase Na+/K+ transporting subunit alpha 1
1,277	ENSG00000203865	ATP1A1-AS1	ATP1A1 antisense RNA 1
1,008	ENSG00000174437	ATP2A2	ATPase sarcoplasmic/endoplasmic reticulum Ca2+ transporting 2
1,275	ENSG00000248919	ATP5J2-PTCD1	ATP5J2-PTCD1 readthrough
1,386	ENSG00000054793	ATP9A	ATPase phospholipid transporting 9A (putative)
1,218	ENSG00000204842	ATXN2	ataxin 2
1,072	ENSG00000168488	ATXN2L	ataxin 2 like
1,336	ENSG00000184809	B3GALT5-AS1	B3GALT5 antisense RNA 1
1,098	ENSG00000176022	B3GALT6	beta-1,3-galactosyltransferase 6
1,06	ENSG00000170340	B3GNT2	UDP-GlcNAc:betaGal beta-1,3-N-acetylglucosaminyltransferase 2
1,057	ENSG00000179913	B3GNT3	UDP-GlcNAc:betaGal beta-1,3-N-acetylglucosaminyltransferase 3
3,624	ENSG00000176597	B3GNT5	UDP-GlcNAc:betaGal beta-1,3-N-acetylglucosaminyltransferase 5
1,209	ENSG00000156966	B3GNT7	UDP-GlcNAc:betaGal beta-1,3-N-acetylglucosaminyltransferase 7
1,432	ENSG00000139044	B4GALNT3	beta-1,4-N-acetyl-galactosaminyltransferase 3
1,264	ENSG00000158470	B4GALT5	beta-1,4-galactosyltransferase 5
1,211	ENSG00000182240	BACE2	beta-site APP-cleaving enzyme 2
1,514	ENSG00000156273	BACH1	BTB domain and CNC homolog 1
5,877	ENSG00000151929	BAG3	BCL2 associated athanogene 3
1,041	ENSG00000140320	BAHD1	bromo adjacent homology domain containing 1
1,718	ENSG00000006453	BAIAP2L1	BAI1 associated protein 2 like 1
1,172	ENSG00000128298	BAIAP2L2	BAI1 associated protein 2 like 2
2,057	ENSG00000095739	BAMBI	BMP and activin membrane bound inhibitor
1,418	ENSG00000138376	BARD1	BRCA1 associated RING domain 1
1,435	ENSG00000043039	BARX2	BARX homeobox 2
2,167	ENSG00000198604	BAZ1A	bromodomain adjacent to zinc finger domain 1A
1,147	ENSG00000076108	BAZ2A	bromodomain adjacent to zinc finger domain 2A
3,098	ENSG00000105327	BBC3	BCL2 binding component 3
1,089	ENSG00000122507	BBS9	Bardet-Biedl syndrome 9
1,755	ENSG00000050820	BCAR1	BCAR1, Cas family scaffolding protein
1,214	ENSG00000064787	BCAS1	breast carcinoma amplified sequence 1
2,665	ENSG00000142867	BCL10	B-cell CLL/lymphoma 10
1,797	ENSG00000171791	BCL2	BCL2, apoptosis regulator
1,338	ENSG00000153094	BCL2L11	BCL2 like 11
1,303	ENSG00000110987	BCL7A	BCL tumor suppressor 7A
1,09	ENSG00000186716	BCR	BCR, RhoGEF and GTPase activating protein
1,297	ENSG00000236824	BCYRN1	brain cytoplasmic RNA 1
1,087	ENSG00000100739	BDKRB1	bradykinin receptor B1
1,34	ENSG00000165626	BEND7	BEN domain containing 7
1,91	ENSG00000133169	BEX1	brain expressed X-linked 1
1,012	ENSG00000133134	BEX2	brain expressed X-linked 2
1,133	ENSG00000102409	BEX4	brain expressed X-linked 4
2,855	ENSG00000123095	BHLHE41	basic helix-loop-helix family member e41
1,619	ENSG00000122870	BICC1	BicC family RNA binding protein 1
2,187	ENSG00000162069	BICDL2	BICD family like cargo adaptor 2
2,927	ENSG00000100290	BIK	BCL2 interacting killer
3,449	ENSG00000023445	BIRC3	baculoviral IAP repeat containing 3
2,852	ENSG00000125845	BMP2	bone morphogenetic protein 2
1,545	ENSG00000157764	BRAF	B-Raf proto-oncogene, serine/threonine kinase
1,355	ENSG00000100425	BRD1	bromodomain containing 1
1,473	ENSG00000141867	BRD4	bromodomain containing 4
1,185	ENSG00000096070	BRPF3	bromodomain and PHD finger containing 3
1,153	ENSG00000119411	BSPRY	B-box and SPRY domain containing
1,213	ENSG00000133639	BTG1	BTG anti-proliferation factor 1
2,471	ENSG00000159388	BTG2	BTG anti-proliferation factor 2
1,777	ENSG00000154640	BTG3	BTG anti-proliferation factor 3
1,78	ENSG00000109944	C11orf63	chromosome 11 open reading frame 63
1,453	ENSG00000175573	C11orf68	chromosome 11 open reading frame 68
1,711	ENSG00000205177	C11orf91	chromosome 11 open reading frame 91
1,496	ENSG00000235162	C12orf75	chromosome 12 open reading frame 75
1,295	ENSG00000140104	C14orf79	chromosome 14 open reading frame 79
2,195	ENSG00000188549	C15orf52	chromosome 15 open reading frame 52
1,25	ENSG00000182831	C16orf72	chromosome 16 open reading frame 72
1,562	ENSG00000167644	C19orf33	chromosome 19 open reading frame 33
1,05	ENSG00000105072	C19orf44	chromosome 19 open reading frame 44
3,198	ENSG00000163362	C1orf106	chromosome 1 open reading frame 106
3,401	ENSG00000182795	C1orf116	chromosome 1 open reading frame 116
1,003	ENSG00000163263	C1orf189	chromosome 1 open reading frame 189
1,596	ENSG00000119280	C1orf198	chromosome 1 open reading frame 198
1,02	ENSG00000131094	C1QL1	complement C1q like 1
1,102	ENSG00000198535	C2CD4A	C2 calcium dependent domain containing 4A
3,362	ENSG00000205502	C2CD4B	C2 calcium dependent domain containing 4B
1,215	ENSG00000225556	C2CD4D	C2 calcium dependent domain containing 4D
4,965	ENSG00000114529	C3orf52	chromosome 3 open reading frame 52
1,271	ENSG00000181744	C3orf58	chromosome 3 open reading frame 58
1,324	ENSG00000178776	C5orf46	chromosome 5 open reading frame 46
1,158	ENSG00000188112	C6orf132	chromosome 6 open reading frame 132
4,776	ENSG00000197261	C6orf141	chromosome 6 open reading frame 141
1,873	ENSG00000189325	C6orf222	chromosome 6 open reading frame 222
1,403	ENSG00000176907	C8orf4	chromosome 8 open reading frame 4
1,089	ENSG00000160345	C9orf116	chromosome 9 open reading frame 116
1,362	ENSG00000204352	C9orf129	chromosome 9 open reading frame 129
1,019	ENSG00000185015	CA13	carbonic anhydrase 13
1,855	ENSG00000164879	CA3	carbonic anhydrase 3
1,999	ENSG00000107159	CA9	carbonic anhydrase 9
1,555	ENSG00000116161	CACYBP	calcyclin binding protein
1,207	ENSG00000084774	CAD	carbamoyl-phosphate synthetase 2, aspartate transcarbamylase, and dihydroorotase
1,504	ENSG00000130559	CAMSAP1	calmodulin regulated spectrin associated protein 1
1,33	ENSG00000076826	CAMSAP3	calmodulin regulated spectrin associated protein family member 3
1,167	ENSG00000042493	CAPG	capping actin protein, gelsolin like
1,116	ENSG00000182472	CAPN12	calpain 12
1,297	ENSG00000162909	CAPN2	calpain 2
1,247	ENSG00000077274	CAPN6	calpain 6
1,127	ENSG00000203697	CAPN8	calpain 8
1,286	ENSG00000100065	CARD10	caspase recruitment domain family member 10
1,526	ENSG00000132357	CARD6	caspase recruitment domain family member 6
2,518	ENSG00000130940	CASZ1	castor zinc finger 1
1,634	ENSG00000105974	CAV1	caveolin 1
1,16	ENSG00000114423	CBLB	Cbl proto-oncogene B
1,459	ENSG00000141582	CBX4	chromobox 4
1,408	ENSG00000183741	CBX6	chromobox 6
1,357	ENSG00000158941	CCAR2	cell cycle and apoptosis regulator 2
1,079	ENSG00000135736	CCDC102A	coiled-coil domain containing 102A
1,04	ENSG00000151773	CCDC122	coiled-coil domain containing 122
1,624	ENSG00000163492	CCDC141	coiled-coil domain containing 141
1,931	ENSG00000153237	CCDC148	coiled-coil domain containing 148
1,157	ENSG00000165813	CCDC186	coiled-coil domain containing 186
1,315	ENSG00000196118	CCDC189	coiled-coil domain containing 189
1,739	ENSG00000159214	CCDC24	coiled-coil domain containing 24
1,025	ENSG00000108091	CCDC6	coiled-coil domain containing 6
1,045	ENSG00000163040	CCDC74A	coiled-coil domain containing 74A
1,301	ENSG00000152076	CCDC74B	coiled-coil domain containing 74B
1,017	ENSG00000169515	CCDC8	coiled-coil domain containing 8
1,632	ENSG00000175602	CCDC85B	coiled-coil domain containing 85B
1,168	ENSG00000205476	CCDC85C	coiled-coil domain containing 85C
1,291	ENSG00000105321	CCDC9	coiled-coil domain containing 9
1,244	ENSG00000125633	CCDC93	coiled-coil domain containing 93
2,17	ENSG00000108691	CCL2	C-C motif chemokine ligand 2
4,782	ENSG00000115009	CCL20	C-C motif chemokine ligand 20
2,147	ENSG00000163660	CCNL1	cyclin L1
1,056	ENSG00000221978	CCNL2	cyclin L2
2,394	ENSG00000126353	CCR7	C-C motif chemokine receptor 7
1,317	ENSG00000117877	CD3EAP	CD3e molecule associated protein
2,625	ENSG00000112149	CD83	CD83 molecule
1,405	ENSG00000198752	CDC42BPB	CDC42 binding protein kinase beta
1,931	ENSG00000149798	CDC42EP2	CDC42 effector protein 2
1,219	ENSG00000179604	CDC42EP4	CDC42 effector protein 4
2,351	ENSG00000163814	CDCP1	CUB domain containing protein 1
2,411	ENSG00000039068	CDH1	cadherin 1
2,208	ENSG00000113361	CDH6	cadherin 6
1,17	ENSG00000156345	CDK20	cyclin dependent kinase 20
1,02	ENSG00000008086	CDKL5	cyclin dependent kinase like 5
2,329	ENSG00000124762	CDKN1A	cyclin dependent kinase inhibitor 1A

1,929	ENSG00000134873	CLDN10	claudin 10
1,183	ENSG00000253958	CLDN23	claudin 23
1,207	ENSG00000184697	CLDN6	claudin 6
1,154	ENSG00000181885	CLDN7	claudin 7
2,465	ENSG00000213937	CLDN9	claudin 9
1,007	ENSG00000038532	CLEC16A	C-type lectin domain containing 16A
1,945	ENSG00000159212	CLIC6	chloride intracellular channel 6
1,047	ENSG00000113282	CLINT1	clathrin interactor 1
2,27	ENSG00000013441	CLK1	CDC like kinase 1
1,543	ENSG00000153551	CMTM7	CKLF like MARVEL transmembrane domain containing 7
2,203	ENSG00000105427	CNFN	cornifelin
1,624	ENSG00000142675	CNKSR1	connector enhancer of kinase suppressor of Ras 1
1,593	ENSG00000158158	CNNM4	cyclin and CBS domain divalent metal cation transport mediator 4
1,058	ENSG00000080802	CNOT4	CCR4-NOT transcription complex subunit 4
1,246	ENSG00000170037	CNTROB	centrobin, centriole duplication and spindle assembly protein
1,339	ENSG00000106078	COBL	cordon-bleu WH2 repeat protein
1,819	ENSG00000196739	COL27A1	collagen type XXVII alpha 1 chain
1,473	ENSG00000134871	COL4A2	collagen type IV alpha 2 chain
2,175	ENSG00000049089	COL9A2	collagen type IX alpha 2 chain
1,53	ENSG00000214290	COLCA2	colorectal cancer associated 2
1,108	ENSG00000135678	CPM	carboxypeptidase M
2,099	ENSG00000205560	CPT1B	carnitine palmitoyltransferase 1B
1,817	ENSG00000177685	CRACR2B	calcium release activated channel regulator 2B
3,085	ENSG00000146592	CREB5	cAMP responsive element binding protein 5
1,16	ENSG00000005339	CREBBP	CREB binding protein
1,095	ENSG00000058453	CROCC	ciliary rootlet coiled-coil, rootletin
1,367	ENSG00000095713	CRTAC1	cartilage acidic protein 1
1,397	ENSG00000008405	CRY1	cryptochrome circadian clock 1
1,46	ENSG00000184371	CSF1	colony stimulating factor 1
2,293	ENSG00000108342	CSF3	colony stimulating factor 3
1,277	ENSG00000104218	CSPP1	centrosome and spindle pole associated protein 1
3,026	ENSG00000144655	CSRNP1	cysteine and serine rich nuclear protein 1
1,647	ENSG00000170373	CST1	cystatin SN
2,02	ENSG00000118523	CTGF	connective tissue growth factor
1,01	ENSG00000044115	CTNNA1	catenin alpha 1
1,318	ENSG00000198561	CTNND1	catenin delta 1
2,439	ENSG00000169862	CTNND2	catenin delta 2
1,375	ENSG00000077063	CTTNBP2	cortactin binding protein 2
1,024	ENSG00000178531	CTXN1	cortexin 1
1,692	ENSG00000180891	CUEDC1	CUE domain containing 1
1,4	ENSG00000044090	CUL7	cullin 7
2,101	ENSG00000006210	CX3CL1	C-X3-C motif chemokine ligand 1
4,043	ENSG00000163739	CXCL1	C-X-C motif chemokine ligand 1
3,846	ENSG00000081041	CXCL2	C-X-C motif chemokine ligand 2
4,81	ENSG00000163734	CXCL3	C-X-C motif chemokine ligand 3
2,604	ENSG00000163735	CXCL5	C-X-C motif chemokine ligand 5
2,487	ENSG00000124875	CXCL6	C-X-C motif chemokine ligand 6
3,5	ENSG00000169429	CXCL8	C-X-C motif chemokine ligand 8
1,073	ENSG00000008283	CYB561	cytochrome b561
1,245	ENSG00000235700	CYCSP52	cytochrome c, somatic pseudogene 52
3,026	ENSG00000142871	CYR61	cysteine rich angiogenic inducer 61
1,342	ENSG00000205795	CYS1	cystin 1
1,521	ENSG00000105443	CYTH2	cytohesin 2
1,24	ENSG00000222041	CYTOR	cytoskeleton regulator RNA
1,14	ENSG00000134780	DAGLA	diacylglycerol lipase alpha
1,404	ENSG00000178149	DALRD3	DALR anticodon binding domain containing 3
1,385	ENSG00000196730	DAPK1	death associated protein kinase 1
1,802	ENSG00000167657	DAPK3	death associated protein kinase 3
1,753	ENSG00000113758	DBN1	drebrin 1
1,086	ENSG00000003249	DBNDD1	dysbindin domain containing 1
2,168	ENSG00000146038	DCDC2	doublecortin domain containing 2
1,011	ENSG00000215301	DDX3X	DEAD-box helicase 3, X-linked
1,122	ENSG00000185163	DDX51	DEAD-box helicase 51
2,309	ENSG00000160570	DEDD2	death effector domain containing 2
2,015	ENSG00000203970	DEFB110	defensin beta 110
1,324	ENSG00000168350	DEGS2	delta 4-desaturase, sphingolipid 2
1,098	ENSG00000035499	DEPDC1B	DEP domain containing 1B
1,059	ENSG00000109606	DHX15	DEAH-box helicase 15
1,153	ENSG00000134815	DHX34	DExH-box helicase 34
1,275	ENSG00000135829	DHX9	DExH-box helicase 9
1,02	ENSG00000101191	DIDO1	death inducer-obliterator 1
1,487	ENSG00000165023	DIRAS2	DIRAS family GTPase 2
1,059	ENSG00000144535	DIS3L2	DIS3 like 3'-5' exoribonuclease 2
1,072	ENSG00000151208	DLG5	discs large MAGUK scaffold protein 5
1,024	ENSG00000170579	DLGAP1	DLG associated protein 1
1,112	ENSG00000100206	DMC1	DNA meiotic recombinase 1
1,269	ENSG00000161249	DMKN	dermokine
1,045	ENSG00000185800	DMWD	dystrophia myotonica, WD repeat containing
1,503	ENSG00000256061	DNAAF4	dynein axonemal assembly factor 4
2,467	ENSG00000086061	DNAJA1	DnaJ heat shock protein family (Hsp40) member A1
3,069	ENSG00000140403	DNAJA4	DnaJ heat shock protein family (Hsp40) member A4
5,728	ENSG00000132002	DNAJB1	DnaJ heat shock protein family (Hsp40) member B1
4,088	ENSG00000162616	DNAJB4	DnaJ heat shock protein family (Hsp40) member B4
1,807	ENSG00000244115	DNAJC25-G	DNAJC25-GNG10 readthrough
1,032	ENSG00000106976	DNM1	dynamin 1
1,133	ENSG00000107554	DNMBP	dynamin binding protein
1,241	ENSG00000147459	DOCK5	dedicator of cytokinesis 5
1,011	ENSG00000129932	DOHH	deoxyhypusine hydroxylase
1,187	ENSG00000175920	DOK7	docking protein 7
1,675	ENSG00000104885	DOT1L	DOT1 like histone lysine methyltransferase
1,061	ENSG00000166171	DPCD	deleted in primary ciliary dyskinesia homolog (mouse)
1,173	ENSG00000171962	DRC3	dynein regulatory complex subunit 3
1,658	ENSG00000134755	DSC2	desmocollin 2
1,084	ENSG00000134762	DSC3	desmocollin 3
1,786	ENSG00000046604	DSG2	desmoglein 2
1,873	ENSG00000096696	DSP	desmoplakin
1,209	ENSG00000091073	DTX2	deltex E3 ubiquitin ligase 2
1,286	ENSG00000178498	DTX3	deltex E3 ubiquitin ligase 3
1,288	ENSG00000140279	DUOX2	dual oxidase 2
1,813	ENSG00000140274	DUOXA2	dual oxidase maturation factor 2
2,567	ENSG00000120129	DUSP1	dual specificity phosphatase 1
1,721	ENSG00000143507	DUSP10	dual specificity phosphatase 10
1,058	ENSG00000111266	DUSP16	dual specificity phosphatase 16
1,175	ENSG00000167065	DUSP18	dual specificity phosphatase 18
2,849	ENSG00000158050	DUSP2	dual specificity phosphatase 2
2,449	ENSG00000120875	DUSP4	dual specificity phosphatase 4
2,574	ENSG00000138166	DUSP5	dual specificity phosphatase 5
2,878	ENSG00000184545	DUSP8	dual specificity phosphatase 8
1,185	ENSG00000197102	DYNC1H1	dynein cytoplasmic 1 heavy chain 1
1,381	ENSG00000134874	DZIP1	DAZ interacting zinc finger protein 1
1,881	ENSG00000158163	DZIP1L	DAZ interacting zinc finger protein 1 like
1,056	ENSG00000088881	EBF4	early B-cell factor 4
1,388	ENSG00000038358	EDC4	enhancer of mRNA decapping 4
4,159	ENSG00000078401	EDN1	endothelin 1
2,536	ENSG00000127129	EDN2	endothelin 2
1,253	ENSG00000101210	EEF1A2	eukaryotic translation elongation factor 1 alpha 2
1,549	ENSG00000122547	EEPD1	endonuclease/exonuclease/phosphatase family domain containing
1,129	ENSG00000096093	EFHC1	EF-hand domain containing 1
1,112	ENSG00000090776	EFNB1	ephrin B1
1,2	ENSG00000146648	EGFR	epidermal growth factor receptor
3,348	ENSG00000235947	EGOT	eosinophil granule ontogeny transcript (non-protein coding)
3,87	ENSG00000120738	EGR1	early growth response 1
2,148	ENSG00000179388	EGR3	early growth response 3
1,344	ENSG00000135625	EGR4	early growth response 4
1,755	ENSG00000110047	EHD1	EH domain containing 1
1,2	ENSG00000103966	EHD4	EH domain containing 4
2,526	ENSG00000135373	EHF	ETS homologous factor
1,015	ENSG00000172071	EIF2AK3	eukaryotic translation initiation factor 2 alpha kinase 3
1,001	ENSG00000141543	EIF4A3	eukaryotic translation initiation factor 4A3
2,156	ENSG00000163435	ELF3	E74 like ETS transcription factor 3
1,165	ENSG00000102890	ELMO3	engulfment and cell motility 3
1,637	ENSG00000164181	ELOVL7	ELOVL fatty acid elongase 7
1,303	ENSG00000134531	EMP1	epithelial membrane protein 1
1,41	ENSG00000154380	ENAH	enabled homolog (Drosophila)
1,493	ENSG00000171617	ENC1	ectodermal-neural cortex 1
1,21	ENSG00000129757	CDKN1C	cyclin dependent kinase inhibitor 1C
1,421	ENSG00000168564	CDKN2AIP	CDKN2A interacting protein
3,042	ENSG00000147883	CDKN2B	cyclin dependent kinase inhibitor 2B
1,317	ENSG00000140743	CDR2	cerebellar degeneration related protein 2
1,052	ENSG00000109089	CDR2L	cerebellar degeneration related protein 2 like
1,583	ENSG00000163624	CDS1	CDP-diacylglycerol synthase 1
1,367	ENSG00000091527	CDV3	CDV3 homolog
1,01	ENSG00000166446	CDYL2	chromodomain Y like 2
2,54	ENSG00000086548	CEACAM6	carcinoembryonic antigen related cell adhesion molecule 6
1,429	ENSG00000102901	CENPT	centromere protein T
1,457	ENSG00000163075	CFAP221	cilia and flagella associated protein 221
1,573	ENSG00000003402	CFLAR	CASP8 and FADD like apoptosis regulator
2,247	ENSG00000226312	CFLAR-AS1	CFLAR antisense RNA 1
2,449	ENSG00000001626	CFTR	cystic fibrosis transmembrane conductance regulator
1,238	ENSG00000143375	CGN	cingulin
1,14	ENSG00000153922	CHD1	chromodomain helicase DNA binding protein 1
2,569	ENSG00000173575	CHD2	chromodomain helicase DNA binding protein 2
1,554	ENSG00000170004	CHD3	chromodomain helicase DNA binding protein 3
1,069	ENSG00000111642	CHD4	chromodomain helicase DNA binding protein 4
1,092	ENSG00000109220	CHIC2	cysteine rich hydrophobic domain 2
1,981	ENSG00000110721	CHKA	choline kinase alpha
1,016	ENSG00000203668	CHML	CHM like, Rab escort protein 2
1,768	ENSG00000101421	CHMP4B	charged multivesicular body protein 4B
2,274	ENSG00000110172	CHORDC1	cysteine and histidine rich domain containing 1
1,305	ENSG00000133019	CHRM3	cholinergic receptor muscarinic 3
1,437	ENSG00000122863	CHST3	carbohydrate sulfotransferase 3
1,048	ENSG00000140835	CHST4	carbohydrate sulfotransferase 4
1,346	ENSG00000127586	CHTF18	chromosome transmission fidelity factor 18
1,007	ENSG00000136425	CIB2	calcium and integrin binding family member 2
1,2	ENSG00000179862	CITED4	Cbp/p300 interacting transactivator with Glu/Asp rich carboxy-terminal domain 4
1,11	ENSG00000166165	CKB	creatine kinase B
1,504	ENSG00000131730	CKMT2	creatine kinase, mitochondrial 2
1,101	ENSG00000104859	CLASRP	CLK4 associating serine/arginine rich protein
3,585	ENSG00000175505	CLCF1	cardiotrophin like cytokine factor 1
1,074	ENSG00000114859	CLCN2	chloride voltage-gated channel 2
2,211	ENSG00000163347	CLDN1	claudin 1
1,503	ENSG00000134873	CLDN10	claudin 10
1,451	ENSG00000253958	CLDN23	claudin 23
1,837	ENSG00000184697	CLDN6	claudin 6
1,129	ENSG00000181885	CLDN7	claudin 7
2,319	ENSG00000213937	CLDN9	claudin 9
2,049	ENSG00000159212	CLIC6	chloride intracellular channel 6
1,174	ENSG00000113282	CLINT1	clathrin interactor 1
2,264	ENSG00000013441	CLK1	CDC like kinase 1
1,117	ENSG00000179335	CLK3	CDC like kinase 3
1,259	ENSG00000183723	CMTM4	CKLF like MARVEL transmembrane domain containing 4
1,418	ENSG00000153551	CMTM7	CKLF like MARVEL transmembrane domain containing 7
1,362	ENSG00000105427	CNFN	cornifelin
1,504	ENSG00000142675	CNKSR1	connector enhancer of kinase suppressor of Ras 1
1,18	ENSG00000153721	CNKSR3	CNKSR family member 3
1,884	ENSG00000158158	CNNM4	cyclin and CBS domain divalent metal cation transport mediator 4
1,464	ENSG00000080802	CNOT4	CCR4-NOT transcription complex subunit 4
1,098	ENSG00000113300	CNOT6	CCR4-NOT transcription complex subunit 6
1,64	ENSG00000106078	COBL	cordon-bleu WH2 repeat protein
1,699	ENSG00000196739	COL27A1	collagen type XXVII alpha 1 chain
1,728	ENSG00000187498	COL4A1	collagen type IV alpha 1 chain
1,811	ENSG00000134871	COL4A2	collagen type IV alpha 2 chain
2,139	ENSG00000049089	COL9A2	collagen type IX alpha 2 chain
1,534	ENSG00000214290	COLCA2	colorectal cancer associated 2
1,005	ENSG00000144524	COPS7B	COP9 signalosome subunit 7B
1,868	ENSG00000110880	CORO1C	coronin 1C
1,463	ENSG00000137449	CPEB2	cytoplasmic polyadenylation element binding protein 2
1,206	ENSG00000135678	CPM	carboxypeptidase M
1,797	ENSG00000205560	CPT1B	carnitine palmitoyltransferase 1B
1,968	ENSG00000143320	CRABP2	cellular retinoic acid binding protein 2
1,908	ENSG00000177685	CRACR2B	calcium release activated channel regulator 2B
1,035	ENSG00000007545	CRAMP1	cramped chromatin regulator homolog 1
3,955	ENSG00000146592	CREB5	cAMP responsive element binding protein 5
1,567	ENSG00000095713	CRTAC1	cartilage acidic protein 1
1,047	ENSG00000160741	CRTC2	CREB regulated transcription coactivator 2
2,325	ENSG00000008405	CRY1	cryptochrome circadian clock 1
2,049	ENSG00000184371	CSF1	colony stimulating factor 1
1,997	ENSG00000164400	CSF2	colony stimulating factor 2
4,365	ENSG00000108342	CSF3	colony stimulating factor 3
1,175	ENSG00000141551	CSNK1D	casein kinase 1 delta
1,129	ENSG00000213923	CSNK1E	casein kinase 1 epsilon
3,191	ENSG00000144655	CSRNP1	cysteine and serine rich nuclear protein 1
2,127	ENSG00000170373	CST1	cystatin SN
1,054	ENSG00000175029	CTBP2	C-terminal binding protein 2
2,046	ENSG00000118523	CTGF	connective tissue growth factor
1,402	ENSG00000044115	CTNNA1	catenin alpha 1
1,501	ENSG00000119326	CTNNAL1	catenin alpha like 1
1,614	ENSG00000198561	CTNND1	catenin delta 1
2,393	ENSG00000169862	CTNND2	catenin delta 2
1,231	ENSG00000077063	CTTNBP2	cortactin binding protein 2
1,065	ENSG00000143079	CTTNBP2NL	CTTNBP2 N-terminal like
1,629	ENSG00000180891	CUEDC1	CUE domain containing 1
1,034	ENSG00000036257	CUL3	cullin 3
1,126	ENSG00000044090	CUL7	cullin 7
2,187	ENSG00000006210	CX3CL1	C-X3-C motif chemokine ligand 1
4,118	ENSG00000163739	CXCL1	C-X-C motif chemokine ligand 1
3,847	ENSG00000081041	CXCL2	C-X-C motif chemokine ligand 2
4,703	ENSG00000163734	CXCL3	C-X-C motif chemokine ligand 3
2,529	ENSG00000163735	CXCL5	C-X-C motif chemokine ligand 5
2,4	ENSG00000124875	CXCL6	C-X-C motif chemokine ligand 6
3,92	ENSG00000169429	CXCL8	C-X-C motif chemokine ligand 8
1,039	ENSG00000008283	CYB561	cytochrome b561
1,767	ENSG00000083799	CYLD	CYLD lysine 63 deubiquitinase
3,612	ENSG00000142871	CYR61	cysteine rich angiogenic inducer 61
1,368	ENSG00000205795	CYS1	cystin 1
1,74	ENSG00000105443	CYTH2	cytohesin 2
1,614	ENSG00000222041	CYTOR	cytoskeleton regulator RNA
1,142	ENSG00000136848	DAB2IP	DAB2 interacting protein
1,177	ENSG00000178149	DALRD3	DALR anticodon binding domain containing 3
1,306	ENSG00000196730	DAPK1	death associated protein kinase 1
2,199	ENSG00000167657	DAPK3	death associated protein kinase 3
1,827	ENSG00000113758	DBN1	drebrin 1
1,162	ENSG00000003249	DBNDD1	dysbindin domain containing 1
1,926	ENSG00000146038	DCDC2	doublecortin domain containing 2
1,077	ENSG00000188215	DCUN1D3	defective in cullin neddylation 1 domain containing 3
1,166	ENSG00000165732	DDX21	DExD-box helicase 21
1,066	ENSG00000089737	DDX24	DEAD-box helicase 24
1,333	ENSG00000215301	DDX3X	DEAD-box helicase 3, X-linked
1,438	ENSG00000067048	DDX3Y	DEAD-box helicase 3, Y-linked
1,442	ENSG00000080007	DDX43	DEAD-box helicase 43
1,087	ENSG00000185163	DDX51	DEAD-box helicase 51
2,278	ENSG00000160570	DEDD2	death effector domain containing 2
1,218	ENSG00000177243	DEFB103A/DEI	defensin beta 103B
1,368	ENSG00000168350	DEGS2	delta 4-desaturase, sphingolipid 2
1,306	ENSG00000175984	DENND2C	DENN domain containing 2C
1,541	ENSG00000174485	DENND4A	DENN domain containing 4A
1,172	ENSG00000184014	DENND5A	DENN domain containing 5A
1,099	ENSG00000070413	DGCR2	DiGeorge syndrome critical region gene 2
1,704	ENSG00000109606	DHX15	DEAH-box helicase 15
1,137	ENSG00000134815	DHX34	DExH-box helicase 34
1,561	ENSG00000135829	DHX9	DExH-box helicase 9
1,243	ENSG00000101191	DIDO1	death inducer-obliterator 1
1,189	ENSG00000066084	DIP2B	disco interacting protein 2 homolog B
1,091	ENSG00000165023	DIRAS2	DIRAS family GTPase 2
1,061	ENSG00000144535	DIS3L2	DIS3 like 3'-5' exoribonuclease 2
1,111	ENSG00000164741	DLC1	DLC1 Rho GTPase activating protein
1,36	ENSG00000170579	DLGAP1	DLG associated protein 1
1,106	ENSG00000161249	DMKN	dermokine
2,701	ENSG00000086061	DNAJA1	DnaJ heat shock protein family (Hsp40) member A1
3,526	ENSG00000140403	DNAJA4	DnaJ heat shock protein family (Hsp40) member A4

1,044	ENSG00000167280	ENGASE	endo-beta-N-acetylglucosaminidase
1,293	ENSG00000100393	EP300	E1A binding protein p300
1,059	ENSG00000088367	EPB41L1	erythrocyte membrane protein band 4.1 like 1
2,514	ENSG00000119888	EPCAM	epithelial cell adhesion molecule
1,077	ENSG00000183317	EPHA10	EPH receptor A10
2,966	ENSG00000142627	EPHA2	EPH receptor A2
1,11	ENSG00000177106	EPS8L2	EPS8 like 2
1,681	ENSG00000198758	EPS8L3	EPS8 like 3
3,051	ENSG00000124882	EREG	epiregulin
1,277	ENSG00000177459	ERICH5	glutamate rich 5
1,054	ENSG00000134398	ERN2	endoplasmic reticulum to nucleus signaling 2
1,615	ENSG00000116285	ERRFI1	ERBB receptor feedback inhibitor 1
1,622	ENSG00000213462	ERV3-1	endogenous retrovirus group 3 member 1, envelope
1,887	ENSG00000104413	ESRP1	epithelial splicing regulatory protein 1
1,06	ENSG00000196482	ESRRG	estrogen related receptor gamma
1,799	ENSG00000117036	ETV3	ETS variant 3
2,315	ENSG00000175832	ETV4	ETS variant 4
1,483	ENSG00000072840	EVC	EvC ciliary complex subunit 1
1,374	ENSG00000173040	EVC2	EvC ciliary complex subunit 2
1,167	ENSG00000187609	EXD3	exonuclease 3'-5' domain containing 3
1,098	ENSG00000144036	EXOC6B	exocyst complex component 6B
2,38	ENSG00000092820	EZR	ezrin
1,697	ENSG00000164251	F2RL1	F2R like trypsin receptor 1
1,555	ENSG00000117525	F3	coagulation factor III, tissue factor
2,271	ENSG00000103089	FA2H	fatty acid 2-hydroxylase
1,12	ENSG00000221968	FADS3	fatty acid desaturase 3
1,902	ENSG00000167106	FAM102A	family with sequence similarity 102 member A
1,159	ENSG00000184731	FAM110C	family with sequence similarity 110 member C
1,204	ENSG00000138439	FAM117B	family with sequence similarity 117 member B
1,579	ENSG00000164142	FAM160A1	family with sequence similarity 160 member A1
1,251	ENSG00000188163	FAM166A	family with sequence similarity 166 member A
1,569	ENSG00000148468	FAM171A1	family with sequence similarity 171 member A1
1,949	ENSG00000146067	FAM193B	family with sequence similarity 193 member B
1,076	ENSG00000204860	FAM201A	family with sequence similarity 201 member A
1,106	ENSG00000183844	FAM3B	family with sequence similarity 3 member B
1	ENSG00000198643	FAM3D	family with sequence similarity 3 member D
2,927	ENSG00000112773	FAM46A	family with sequence similarity 46 member A
2,298	ENSG00000158246	FAM46B	family with sequence similarity 46 member B
1,244	ENSG00000183508	FAM46C	family with sequence similarity 46 member C
2,124	ENSG00000120709	FAM53C	family with sequence similarity 53 member C
1,301	ENSG00000139146	FAM60A	family with sequence similarity 60 member A
1,418	ENSG00000168143	FAM83B	family with sequence similarity 83 member B
1,873	ENSG00000105523	FAM83E	family with sequence similarity 83 member E
2,746	ENSG00000188522	FAM83G	family with sequence similarity 83 member G
1,333	ENSG00000152767	FARP1	FERM, ARH/RhoGEF and pleckstrin domain protein 1
1,149	ENSG00000162458	FBLIM1	filamin binding LIM protein 1
1,146	ENSG00000156860	FBRS	fibrosin
2,239	ENSG00000171823	FBXL14	F-box and leucine rich repeat protein 14
1,054	ENSG00000130475	FCHO1	FCH domain only 1
1,062	ENSG00000197948	FCHSD1	FCH and double SH3 domains 1
1,602	ENSG00000145780	FEM1C	fem-1 homolog C
1,281	ENSG00000180263	FGD6	FYVE, RhoGEF and PH domain containing 6
1,386	ENSG00000162344	FGF19	fibroblast growth factor 19
1,029	ENSG00000138675	FGF5	fibroblast growth factor 5
1,406	ENSG00000066468	FGFR2	fibroblast growth factor receptor 2
2,122	ENSG00000068078	FGFR3	fibroblast growth factor receptor 3
1,001	ENSG00000160867	FGFR4	fibroblast growth factor receptor 4
2,356	ENSG00000168386	FILIP1L	filamin A interacting protein 1 like
1,963	ENSG00000004478	FKBP4	FK506 binding protein 4
1,845	ENSG00000136068	FLNB	filamin B
1,262	ENSG00000185070	FLRT2	fibronectin leucine rich transmembrane protein 2
2,014	ENSG00000125848	FLRT3	fibronectin leucine rich transmembrane protein 3
1,63	ENSG00000137942	FNBP1L	formin binding protein 1 like
1,392	ENSG00000109920	FNBP4	formin binding protein 4
1,507	ENSG00000110195	FOLR1	folate receptor 1
4,008	ENSG00000170345	FOS	Fos proto-oncogene, AP-1 transcription factor subunit
6,003	ENSG00000125740	FOSB	FosB proto-oncogene, AP-1 transcription factor subunit
4,17	ENSG00000175592	FOSL1	FOS like 1, AP-1 transcription factor subunit
1,985	ENSG00000075426	FOSL2	FOS like 2, AP-1 transcription factor subunit
1,404	ENSG00000129514	FOXA1	forkhead box A1
1,374	ENSG00000125798	FOXA2	forkhead box A2
1,98	ENSG00000129654	FOXJ1	forkhead box J1
1,345	ENSG00000150907	FOXO1	forkhead box O1
1,122	ENSG00000118689	FOXO3	forkhead box O3
1,172	ENSG00000114861	FOXP1	forkhead box P1
1,265	ENSG00000128573	FOXP2	forkhead box P2
2,905	ENSG00000164379	FOXQ1	forkhead box Q1
1,389	ENSG00000138759	FRAS1	Fraser extracellular matrix complex subunit 1
1,247	ENSG00000181274	FRAT2	FRAT2, WNT signaling pathway regulator
1,354	ENSG00000114541	FRMD4B	FERM domain containing 4B
1,252	ENSG00000106701	FSD1L	fibronectin type III and SPRY domain containing 1 like
2,087	ENSG00000070404	FSTL3	follistatin like 3
1,155	ENSG00000089280	FUS	FUS RNA binding protein
1,21	ENSG00000171124	FUT3	fucosyltransferase 3 (Lewis blood group)
1,745	ENSG00000196371	FUT4	fucosyltransferase 4
1,816	ENSG00000137731	FXYD2	FXYD domain containing ion transport regulator 2
1,284	ENSG00000089356	FXYD3	FXYD domain containing ion transport regulator 3
1,386	ENSG00000157240	FZD1	frizzled class receptor 1
1,499	ENSG00000102287	GABRE	gamma-aminobutyric acid type A receptor epsilon subunit
2,093	ENSG00000094755	GABRP	gamma-aminobutyric acid type A receptor pi subunit
1,487	ENSG00000116717	GADD45A	growth arrest and DNA damage inducible alpha
1,485	ENSG00000099860	GADD45B	growth arrest and DNA damage inducible beta
1,319	ENSG00000128242	GAL3ST1	galactose-3-O-sulfotransferase 1
1,678	ENSG00000119514	GALNT12	polypeptide N-acetylgalactosaminyltransferase 12
2,333	ENSG00000115339	GALNT3	polypeptide N-acetylgalactosaminyltransferase 3
1,486	ENSG00000257594	GALNT4	polypeptide N-acetylgalactosaminyltransferase 4
1,237	ENSG00000141448	GATA6	GATA binding protein 6
1,029	ENSG00000070610	GBA2	glucosylceramidase beta 2
1,055	ENSG00000107862	GBF1	golgi brefeldin A resistant guanine nucleotide exchange factor 1
1,096	ENSG00000117228	GBP1	guanylate binding protein 1
1,948	ENSG00000140297	GCNT3	glucosaminyl (N-acetyl) transferase 3, mucin type
2,12	ENSG00000130513	GDF15	growth differentiation factor 15
2,527	ENSG00000167741	GGT6	gamma-glutamyltransferase 6
1,195	ENSG00000106128	GHRHR	growth hormone releasing hormone receptor
1,085	ENSG00000146830	GIGYF1	GRB10 interacting GYF protein 1
1,62	ENSG00000188910	GJB3	gap junction protein beta 3
1,212	ENSG00000126603	GLIS2	GLIS family zinc finger 2
1,989	ENSG00000107249	GLIS3	GLIS family zinc finger 3
1,062	ENSG00000237009	GLIS3-AS1	GLIS3 antisense RNA 1
1,229	ENSG00000109738	GLRB	glycine receptor beta
1,203	ENSG00000120063	GNA13	G protein subunit alpha 13
1,978	ENSG00000215252	GOLGA8A/G	golgin A8 family member A
1,239	ENSG00000116580	GON4L	gon-4 like
1,037	ENSG00000186566	GPATCH8	G-patch domain containing 8
1,061	ENSG00000062194	GPBP1	GC-rich promoter binding protein 1
1,78	ENSG00000166073	GPR176	G protein-coupled receptor 176
1,265	ENSG00000169508	GPR183	G protein-coupled receptor 183
2,926	ENSG00000013588	GPRC5A	G protein-coupled receptor class C group 5 member A
2,376	ENSG00000167191	GPRC5B	G protein-coupled receptor class C group 5 member B
1,752	ENSG00000023171	GRAMD1B	GRAM domain containing 1B
2,575	ENSG00000141738	GRB7	growth factor receptor bound protein 7
1,062	ENSG00000196208	GREB1	growth regulation by estrogen in breast cancer 1
1,968	ENSG00000083307	GRHL2	grainyhead like transcription factor 2
1,03	ENSG00000084207	GSTP1	glutathione S-transferase pi 1
1,767	ENSG00000100226	GTPBP1	GTP binding protein 1
1,673	ENSG00000197273	GUCA2A	guanylate cyclase activator 2A
1,105	ENSG00000144366	GULP1	GULP, engulfment adaptor PTB domain containing 1
1,954	ENSG00000228315	GUSBP11	glucuronidase, beta pseudogene 11
1,068	ENSG00000241549	GUSBP2	glucuronidase, beta pseudogene 2
1,89	ENSG00000253203	GUSBP3	glucuronidase, beta pseudogene 3
1,204	ENSG00000184897	H1FX	H1 histone family member X
1,367	ENSG00000188486	H2AFX	H2A histone family member X
1,117	ENSG00000132475	H3F3A/H3F	H3 histone family member 3A
1,158	ENSG00000188375	H3F3C	H3 histone family member 3C
1,298	ENSG00000103044	HAS3	hyaluronan synthase 3

5,795	ENSG00000132002	DNAJB1	DnaJ heat shock protein family (Hsp40) member B1
4,024	ENSG00000162616	DNAJB4	DnaJ heat shock protein family (Hsp40) member B4
1,293	ENSG00000105993	DNAJB6	DnaJ heat shock protein family (Hsp40) member B6
1,175	ENSG00000244115	DNAJC25-GNG	DNAJC25-GNG10 readthrough
1,273	ENSG00000116675	DNAJC6	DnaJ heat shock protein family (Hsp40) member C6
1,831	ENSG00000107554	DNMBP	dynamin binding protein
1,156	ENSG00000147459	DOCK5	dedicator of cytokinesis 5
2,737	ENSG00000104885	DOT1L	DOT1 like histone lysine methyltransferase
1,125	ENSG00000148399	DPH7	diphthamide biosynthesis 7
1,903	ENSG00000134755	DSC2	desmocollin 2
1,383	ENSG00000134762	DSC3	desmocollin 3
1,884	ENSG00000046604	DSG2	desmoglein 2
1,956	ENSG00000096696	DSP	desmoplakin
1,054	ENSG00000151914	DST	dystonin
1,451	ENSG00000134769	DTNA	dystrobrevin alpha
1,135	ENSG00000091073	DTX2	deltex E3 ubiquitin ligase 2
1,516	ENSG00000140279	DUOX2	dual oxidase 2
2,384	ENSG00000140274	DUOXA2	dual oxidase maturation factor 2
2,309	ENSG00000120129	DUSP1	dual specificity phosphatase 1
1,929	ENSG00000143507	DUSP10	dual specificity phosphatase 10
1,154	ENSG00000167065	DUSP18	dual specificity phosphatase 18
2,763	ENSG00000158050	DUSP2	dual specificity phosphatase 2
2,555	ENSG00000120875	DUSP4	dual specificity phosphatase 4
3,3	ENSG00000138166	DUSP5	dual specificity phosphatase 5
2,79	ENSG00000184545	DUSP8	dual specificity phosphatase 8
1,222	ENSG00000197102	DYNC1H1	dynein cytoplasmic 1 heavy chain 1
1,157	ENSG00000127334	DYRK2	dual specificity tyrosine phosphorylation regulated kinase 2
1,163	ENSG00000134874	DZIP1	DAZ interacting zinc finger protein 1
1,728	ENSG00000158163	DZIP1L	DAZ interacting zinc finger protein 1 like
1,042	ENSG00000169016	E2F6	E2F transcription factor 6
1,766	ENSG00000038358	EDC4	enhancer of mRNA decapping 4
5,154	ENSG00000078401	EDN1	endothelin 1
1,861	ENSG00000127129	EDN2	endothelin 2
1,066	ENSG00000101210	EEF1A2	eukaryotic translation elongation factor 1 alpha 2
1,929	ENSG00000122547	EEPD1	endonuclease/exonuclease/phosphatase family domain containing 1
1,124	ENSG00000140598	EFL1	elongation factor like GTPase 1
1,318	ENSG00000090776	EFNB1	ephrin B1
1,599	ENSG00000125266	EFNB2	ephrin B2
1,154	ENSG00000146648	EGFR	epidermal growth factor receptor
3,365	ENSG00000235947	EGOT	eosinophil granule ontogeny transcript (non-protein coding)
3,692	ENSG00000120738	EGR1	early growth response 1
2,25	ENSG00000179388	EGR3	early growth response 3
1,093	ENSG00000135625	EGR4	early growth response 4
2,879	ENSG00000110047	EHD1	EH domain containing 1
1,581	ENSG00000103966	EHD4	EH domain containing 4
2,83	ENSG00000135373	EHF	ETS homologous factor
1,431	ENSG00000255150	EID3	EP300 interacting inhibitor of differentiation 3
1,718	ENSG00000172071	EIF2AK3	eukaryotic translation initiation factor 2 alpha kinase 3
1,323	ENSG00000107581	EIF3A	eukaryotic translation initiation factor 3 subunit A
1,297	ENSG00000141543	EIF4A3	eukaryotic translation initiation factor 4A3
1,291	ENSG00000120690	ELF1	E74 like ETS transcription factor 1
2,113	ENSG00000163435	ELF3	E74 like ETS transcription factor 3
1,691	ENSG00000105656	ELL	elongation factor for RNA polymerase II
2,384	ENSG00000164181	ELOVL7	ELOVL fatty acid elongase 7
2,512	ENSG00000134531	EMP1	epithelial membrane protein 1
1,303	ENSG00000154380	ENAH	enabled homolog (Drosophila)
1,33	ENSG00000171617	ENC1	ectodermal-neural cortex 1
1,342	ENSG00000100393	EP300	E1A binding protein p300
1,122	ENSG00000120616	EPC1	enhancer of polycomb homolog 1
2,58	ENSG00000119888	EPCAM	epithelial cell adhesion molecule
3,748	ENSG00000142627	EPHA2	EPH receptor A2
1,136	ENSG00000182580	EPHB3	EPH receptor B3
1,926	ENSG00000198758	EPS8L3	EPS8 like 3
2,933	ENSG00000124882	EREG	epiregulin
1,115	ENSG00000134398	ERN2	endoplasmic reticulum to nucleus signaling 2
2,114	ENSG00000116285	ERRFI1	ERBB receptor feedback inhibitor 1
1,325	ENSG00000213462	ERV3-1	endogenous retrovirus group 3 member 1, envelope
1,454	ENSG00000164283	ESM1	endothelial cell specific molecule 1
1,601	ENSG00000104413	ESRP1	epithelial splicing regulatory protein 1
1,189	ENSG00000196482	ESRRG	estrogen related receptor gamma
2,044	ENSG00000134954	ETS1	ETS proto-oncogene 1, transcription factor
1,539	ENSG00000157557	ETS2	ETS proto-oncogene 2, transcription factor
1,61	ENSG00000117036	ETV3	ETS variant 3
2,038	ENSG00000175832	ETV4	ETS variant 4
1,458	ENSG00000072840	EVC	EvC ciliary complex subunit 1
1,396	ENSG00000173040	EVC2	EvC ciliary complex subunit 2
1,063	ENSG00000182944	EWSR1	EWS RNA binding protein 1
1,26	ENSG00000187609	EXD3	exonuclease 3'-5' domain containing 3
1,142	ENSG00000144036	EXOC6B	exocyst complex component 6B
1,295	ENSG00000116903	EXOC8	exocyst complex component 8
1,286	ENSG00000106462	EZH2	enhancer of zeste 2 polycomb repressive complex 2 subunit
3,111	ENSG00000092820	EZR	ezrin
2,565	ENSG00000164251	F2RL1	F2R like trypsin receptor 1
2,027	ENSG00000117525	F3	coagulation factor III, tissue factor
2,849	ENSG00000103089	FA2H	fatty acid 2-hydroxylase
1,429	ENSG00000221968	FADS3	fatty acid desaturase 3
2,981	ENSG00000167106	FAM102A	family with sequence similarity 102 member A
1,164	ENSG00000065809	FAM107B	family with sequence similarity 107 member B
2,055	ENSG00000184731	FAM110C	family with sequence similarity 110 member C
1,105	ENSG00000082269	FAM135A	family with sequence similarity 135 member A
2,356	ENSG00000164142	FAM160A1	family with sequence similarity 160 member A1
1,146	ENSG00000051009	FAM160A2	family with sequence similarity 160 member A2
1,834	ENSG00000188163	FAM166A	family with sequence similarity 166 member A
1,054	ENSG00000152102	FAM168B	family with sequence similarity 168 member B
1,679	ENSG00000148468	FAM171A1	family with sequence similarity 171 member A1
1,178	ENSG00000125386	FAM193A	family with sequence similarity 193 member A
2,121	ENSG00000146067	FAM193B	family with sequence similarity 193 member B
1,176	ENSG00000005238	FAM214B	family with sequence similarity 214 member B
1,167	ENSG00000198643	FAM3D	family with sequence similarity 3 member D
2,907	ENSG00000112773	FAM46A	family with sequence similarity 46 member A
2,744	ENSG00000158246	FAM46B	family with sequence similarity 46 member B
1,385	ENSG00000183508	FAM46C	family with sequence similarity 46 member C
2,242	ENSG00000120709	FAM53C	family with sequence similarity 53 member C
1,474	ENSG00000167695	FAM57A	family with sequence similarity 57 member A
1,349	ENSG00000139146	FAM60A	family with sequence similarity 60 member A
1,86	ENSG00000168143	FAM83B	family with sequence similarity 83 member B
2,023	ENSG00000105523	FAM83E	family with sequence similarity 83 member E
3,435	ENSG00000188522	FAM83G	family with sequence similarity 83 member G
1,139	ENSG00000162981	FAM84A	family with sequence similarity 84 member A
1,485	ENSG00000168672	FAM84B	family with sequence similarity 84 member B
1,268	ENSG00000162458	FBLIM1	filamin binding LIM protein 1
1,32	ENSG00000156860	FBRS	fibrosin
2,581	ENSG00000171823	FBXL14	F-box and leucine rich repeat protein 14
1,431	ENSG00000153558	FBXL2	F-box and leucine rich repeat protein 2
1,055	ENSG00000138081	FBXO11	F-box protein 11
1,567	ENSG00000118496	FBXO30	F-box protein 30
1,074	ENSG00000072803	FBXW11	F-box and WD repeat domain containing 11
1,058	ENSG00000109670	FBXW7	F-box and WD repeat domain containing 7
1,997	ENSG00000145780	FEM1C	fem-1 homolog C
1,461	ENSG00000180263	FGD6	FYVE, RhoGEF and PH domain containing 6
2,096	ENSG00000162344	FGF19	fibroblast growth factor 19
1,486	ENSG00000077782	FGFR1	fibroblast growth factor receptor 1
1,048	ENSG00000066468	FGFR2	fibroblast growth factor receptor 2
1,813	ENSG00000068078	FGFR3	fibroblast growth factor receptor 3
2,173	ENSG00000168386	FILIP1L	filamin A interacting protein 1 like
1,4	ENSG00000179431	FJX1	four jointed box 1
2,161	ENSG00000004478	FKBP4	FK506 binding protein 4
1,103	ENSG00000181027	FKRP	fukutin related protein
1,033	ENSG00000177731	FLII	FLII, actin remodeling protein
1,826	ENSG00000136068	FLNB	filamin B
1,641	ENSG00000128591	FLNC	filamin C
1,322	ENSG00000185070	FLRT2	fibronectin leucine rich transmembrane protein 2
2,006	ENSG00000125848	FLRT3	fibronectin leucine rich transmembrane protein 3
1,842	ENSG00000137942	FNBP1L	formin binding protein 1 like
1,384	ENSG00000109920	FNBP4	formin binding protein 4

3,814	ENSG00000113070	HBEGF	heparin binding EGF like growth factor
1,084	ENSG00000048052	HDAC9	histone deacetylase 9
1,094	ENSG00000167674	HDGFL2	HDGF like 2
2,702	ENSG00000230267	HERC2P4	hect domain and RLD 2 pseudogene 4
1,005	ENSG00000114315	HES1	hes family bHLH transcription factor 1
1,902	ENSG00000188290	HES4	hes family bHLH transcription factor 4
1,454	ENSG00000186834	HEXIM1	hexamethylene bisacetamide inducible 1
1,139	ENSG00000100644	HIF1A	hypoxia inducible factor 1 alpha subunit
1,902	ENSG00000130787	HIP1R	huntingtin interacting protein 1 related
2,193	ENSG00000196866	HIST1H2AD	histone cluster 1 H2A family member d
1,409	ENSG00000124635	HIST1H2BJ	histone cluster 1 H2B family member j
2,318	ENSG00000233822	HIST1H2BN	histone cluster 1 H2B family member n
1,994	ENSG00000197409	HIST1H3D	histone cluster 1 H3 family member d
1,251	ENSG00000197153	HIST1H3J	histone cluster 1 H3 family member j
1,603	ENSG00000196890	HIST3H2BB	histone cluster 3 H2B family member b
1,456	ENSG00000197837	HIST4H4	histone cluster 4 H4
1,026	ENSG00000095951	HIVEP1	human immunodeficiency virus type I enhancer binding protein 1
1,831	ENSG00000010818	HIVEP2	human immunodeficiency virus type I enhancer binding protein 2
1,701	ENSG00000156510	HKDC1	hexokinase domain containing 1
2,612	ENSG00000137309	HMGA1	high mobility group AT-hook 1
1,443	ENSG00000113161	HMGCR	3-hydroxy-3-methylglutaryl-CoA reductase
1,823	ENSG00000112972	HMGCS1	3-hydroxy-3-methylglutaryl-CoA synthase 1
1,063	ENSG00000113716	HMGXB3	HMG-box containing 3
1,195	ENSG00000177733	HNRNPA0	heterogeneous nuclear ribonucleoprotein A0
1,352	ENSG00000169045	HNRNPH1	heterogeneous nuclear ribonucleoprotein H1
1,039	ENSG00000096746	HNRNPH3	heterogeneous nuclear ribonucleoprotein H3
1,595	ENSG00000153187	HNRNPU	heterogeneous nuclear ribonucleoprotein U
1,125	ENSG00000103942	HOMER2	homer scaffolding protein 2
7,313	ENSG00000257017	HP	haptoglobin
1,146	ENSG00000127252	HRASLS	HRAS like suppressor
1,477	ENSG00000002587	HS3ST1	heparan sulfate-glucosamine 3-sulfotransferase 1
1,022	ENSG00000099251	HSD17B7P2	hydroxysteroid 17-beta dehydrogenase 7 pseudogene 2
1,627	ENSG00000102878	HSF4	heat shock transcription factor 4
2,99	ENSG00000080824	HSP90AA1	heat shock protein 90 alpha family class A member 1
2,668	ENSG00000096384	HSP90AB1	heat shock protein 90 alpha family class B member 1
2,059	ENSG00000205940	HSP90AB2P	heat shock protein 90 alpha family class B member 2, pseudogene
1,081	ENSG00000170606	HSPA4	heat shock protein family A (Hsp70) member 4
1,319	ENSG00000164070	HSPA4L	heat shock protein family A (Hsp70) member 4 like
1,29	ENSG00000044574	HSPA5	heat shock protein family A (Hsp70) member 5
6,995	ENSG00000173110	HSPA6	heat shock protein family A (Hsp70) member 6
6,679	ENSG00000225217	HSPA7	heat shock protein family A (Hsp70) member 7
2,462	ENSG00000109971	HSPA8	heat shock protein family A (Hsp70) member 8
1,387	ENSG00000144381	HSPD1	heat shock protein family D (Hsp60) member 1
5,132	ENSG00000120694	HSPH1	heat shock protein family H (Hsp110) member 1
2,069	ENSG00000142149	HUNK	hormonally up-regulated Neu-associated kinase
1,038	ENSG00000157423	HYDIN	HYDIN, axonemal central pair apparatus protein
1,283	ENSG00000163596	ICA1L	islet cell autoantigen 1 like
2,229	ENSG00000090339	ICAM1	intercellular adhesion molecule 1
1,02	ENSG00000105371	ICAM4	intercellular adhesion molecule 4 (Landsteiner-Wiener blood group)
1,034	ENSG00000172201	ID4	inhibitor of DNA binding 4, HLH protein
2,429	ENSG00000232656	IDI2-AS1	IDI2 antisense RNA 1
1,349	ENSG00000127415	IDUA	iduronidase, alpha-L-
2,343	ENSG00000160888	IER2	immediate early response 2
3,488	ENSG00000162783	IER5	immediate early response 5
1,004	ENSG00000188483	IER5L	immediate early response 5 like
1,91	ENSG00000169991	IFFO2	intermediate filament family orphan 2
1,823	ENSG00000006652	IFRD1	interferon related developmental regulator 1
1,545	ENSG00000187535	IFT140	intraflagellar transport 140
1,056	ENSG00000138002	IFT172	intraflagellar transport 172
1,22	ENSG00000122970	IFT81	intraflagellar transport 81
1,495	ENSG00000073792	IGF2BP2	insulin like growth factor 2 mRNA binding protein 2
1,709	ENSG00000143061	IGSF3	immunoglobulin superfamily member 3
1,416	ENSG00000150782	IL18	interleukin 18
2,389	ENSG00000125538	IL1B	interleukin 1 beta
3,182	ENSG00000110944	IL23A	interleukin 23 subunit alpha
1,057	ENSG00000077238	IL4R	interleukin 4 receptor
2,2	ENSG00000136244	IL6	interleukin 6
1,345	ENSG00000145103	ILDR1	immunoglobulin like domain containing receptor 1
1,73	ENSG00000129351	ILF3	interleukin enhancer binding factor 3
1,323	ENSG00000203485	INF2	inverted formin, FH2 and WH2 domain containing
2,122	ENSG00000122641	INHBA	inhibin beta A subunit
1,32	ENSG00000148384	INPP5E	inositol polyphosphate-5-phosphatase E
1,312	ENSG00000164066	INTU	inturned planar cell polarity protein
1,017	ENSG00000161896	IP6K3	inositol hexakisphosphate kinase 3
2,335	ENSG00000132321	IQCA1	IQ motif containing with AAA domain 1
1,416	ENSG00000174628	IQCK	IQ motif containing K
1,083	ENSG00000164675	IQUB	IQ motif and ubiquitin domain containing
1,926	ENSG00000134070	IRAK2	interleukin 1 receptor associated kinase 2
1,855	ENSG00000125347	IRF1	interferon regulatory factor 1
1,064	ENSG00000168264	IRF2BP2	interferon regulatory factor 2 binding protein 2
1,628	ENSG00000117595	IRF6	interferon regulatory factor 6
1,124	ENSG00000167378	IRGQ	immunity related GTPase Q
2,15	ENSG00000185950	IRS2	insulin receptor substrate 2
1,812	ENSG00000105655	ISYNA1	inositol-3-phosphate synthase 1
1,497	ENSG00000164171	ITGA2	integrin subunit alpha 2
2,222	ENSG00000005884	ITGA3	integrin subunit alpha 3
1,317	ENSG00000161638	ITGA5	integrin subunit alpha 5
1,024	ENSG00000091409	ITGA6	integrin subunit alpha 6
1,949	ENSG00000132470	ITGB4	integrin subunit beta 4
1,493	ENSG00000115221	ITGB6	integrin subunit beta 6
2,461	ENSG00000105855	ITGB8	integrin subunit beta 8
1,874	ENSG00000123243	ITIH5	inter-alpha-trypsin inhibitor heavy chain family member 5
2,98	ENSG00000086544	ITPKC	inositol-trisphosphate 3-kinase C
1,333	ENSG00000096433	ITPR3	inositol 1,4,5-trisphosphate receptor type 3
1,074	ENSG00000171988	JMJD1C	jumonji domain containing 1C
1,973	ENSG00000070495	JMJD6	arginine demethylase and lysine hydroxylase
3,82	ENSG00000177606	JUN	Jun proto-oncogene, AP-1 transcription factor subunit
1,896	ENSG00000171223	JUNB	JunB proto-oncogene, AP-1 transcription factor subunit
3,112	ENSG00000130522	JUND	JunD proto-oncogene, AP-1 transcription factor subunit
2,83	ENSG00000146049	KAAG1	kidney associated antigen 1
1,851	ENSG00000162975	KCNF1	potassium voltage-gated channel modifier subfamily F member 1
1,212	ENSG00000182132	KCNIP1	potassium voltage-gated channel interacting protein 1
1,057	ENSG00000115474	KCNJ13	potassium voltage-gated channel subfamily J member 13
1,286	ENSG00000157551	KCNJ15	potassium voltage-gated channel subfamily J member 15
1,521	ENSG00000153822	KCNJ16	potassium voltage-gated channel subfamily J member 16
1,088	ENSG00000082482	KCNK2	potassium two pore domain channel subfamily K member 2
1,802	ENSG00000164626	KCNK5	potassium two pore domain channel subfamily K member 5
1,2	ENSG00000213859	KCTD11	potassium channel tetramerization domain containing 11
1,112	ENSG00000174943	KCTD13	potassium channel tetramerization domain containing 13
1,493	ENSG00000173120	KDM2A	lysine demethylase 2A
1,005	ENSG00000107077	KDM4C	lysine demethylase 4C
1,902	ENSG00000117139	KDM5B	lysine demethylase 5B
1,031	ENSG00000012817	KDM5D	lysine demethylase 5D
1,519	ENSG00000147050	KDM6A	lysine demethylase 6A
1,917	ENSG00000132510	KDM6B	lysine demethylase 6B
1,133	ENSG00000047578	KIAA0556	KIAA0556
1,054	ENSG00000164542	KIAA0895	KIAA0895
1,696	ENSG00000196123	KIAA0895L	KIAA0895 like
1,034	ENSG00000120549	KIAA1217	KIAA1217
1,984	ENSG00000162522	KIAA1522	KIAA1522
2,766	ENSG00000136883	KIF12	kinesin family member 12
1,136	ENSG00000197892	KIF13B	kinesin family member 13B
1,032	ENSG00000131437	KIF3A	kinesin family member 3A
1,571	ENSG00000167702	KIFC2	kinesin family member C2
1,041	ENSG00000151657	KIN	Kin17 DNA and RNA binding protein
1,051	ENSG00000174996	KLC2	kinesin light chain 2
1,584	ENSG00000104892	KLC3	kinesin light chain 3
1,818	ENSG00000155090	KLF10	Kruppel like factor 10
2,544	ENSG00000127528	KLF2	Kruppel like factor 2
2,324	ENSG00000136826	KLF4	Kruppel like factor 4
3,599	ENSG00000102554	KLF5	Kruppel like factor 5
3,471	ENSG00000067082	KLF6	Kruppel like factor 6
1,324	ENSG00000118263	KLF7	Kruppel like factor 7
1,037	ENSG00000174010	KLHL15	kelch like family member 15
1,351	ENSG00000187961	KLHL17	kelch like family member 17
1,536	ENSG00000110195	FOLR1	folate receptor 1
3,627	ENSG00000170345	FOS	Fos proto-oncogene, AP-1 transcription factor subunit
5,894	ENSG00000125740	FOSB	FosB proto-oncogene, AP-1 transcription factor subunit
5,878	ENSG00000175592	FOSL1	FOS like 1, AP-1 transcription factor subunit
2,21	ENSG00000075426	FOSL2	FOS like 2, AP-1 transcription factor subunit
1,502	ENSG00000129514	FOXA1	forkhead box A1
1,553	ENSG00000125798	FOXA2	forkhead box A2
2,03	ENSG00000129654	FOXJ1	forkhead box J1
1,174	ENSG00000198815	FOXJ3	forkhead box J3
1,103	ENSG00000164916	FOXK1	forkhead box K1
1,829	ENSG00000150907	FOXO1	forkhead box O1
1,072	ENSG00000118689	FOXO3	forkhead box O3
1,115	ENSG00000114861	FOXP1	forkhead box P1
3,162	ENSG00000164379	FOXQ1	forkhead box Q1
1,305	ENSG00000138759	FRAS1	Fraser extracellular matrix complex subunit 1
1,343	ENSG00000181274	FRAT2	FRAT2, WNT signaling pathway regulator
1,325	ENSG00000111816	FRK	fyn related Src family tyrosine kinase
1,114	ENSG00000172159	FRMD3	FERM domain containing 3
1,888	ENSG00000114541	FRMD4B	FERM domain containing 4B
1,455	ENSG00000139926	FRMD6	FERM domain containing 6
1,715	ENSG00000106701	FSD1L	fibronectin type III and SPRY domain containing 1 like
2,497	ENSG00000070404	FSTL3	follistatin like 3
1,09	ENSG00000162613	FUBP1	far upstream element binding protein 1
1,368	ENSG00000089280	FUS	FUS RNA binding protein
1,437	ENSG00000171124	FUT3	fucosyltransferase 3 (Lewis blood group)
1,533	ENSG00000196371	FUT4	fucosyltransferase 4
1,224	ENSG00000137731	FXYD2	FXYD domain containing ion transport regulator 2
1,248	ENSG00000089356	FXYD3	FXYD domain containing ion transport regulator 3
1,386	ENSG00000157240	FZD1	frizzled class receptor 1
1,046	ENSG00000109458	GAB1	GRB2 associated binding protein 1
1,22	ENSG00000102287	GABRE	gamma-aminobutyric acid type A receptor epsilon subunit
2,149	ENSG00000094755	GABRP	gamma-aminobutyric acid type A receptor pi subunit
2,012	ENSG00000116717	GADD45A	growth arrest and DNA damage inducible alpha
1,576	ENSG00000099860	GADD45B	growth arrest and DNA damage inducible beta
1,781	ENSG00000069482	GAL	galanin and GMAP prepropeptide
1,172	ENSG00000128242	GAL3ST1	galactose-3-O-sulfotransferase 1
1,796	ENSG00000119514	GALNT12	polypeptide N-acetylgalactosaminyltransferase 12
2,37	ENSG00000115339	GALNT3	polypeptide N-acetylgalactosaminyltransferase 3
1,33	ENSG00000257594	GALNT4	polypeptide N-acetylgalactosaminyltransferase 4
1,151	ENSG00000141448	GATA6	GATA binding protein 6
1,206	ENSG00000070610	GBA2	glucosylceramidase beta 2
1,377	ENSG00000107862	GBF1	golgi brefeldin A resistant guanine nucleotide exchange factor 1
1,327	ENSG00000117228	GBP1	guanylate binding protein 1
1,275	ENSG00000162645	GBP2	guanylate binding protein 2
2,079	ENSG00000140297	GCNT3	glucosaminyl (N-acetyl) transferase 3, mucin type
1,798	ENSG00000130513	GDF15	growth differentiation factor 15
1,84	ENSG00000164949	GEM	GTP binding protein overexpressed in skeletal muscle
2,261	ENSG00000167741	GGT6	gamma-glutamyltransferase 6
2,266	ENSG00000188910	GJB3	gap junction protein beta 3
1,071	ENSG00000189433	GJB4	gap junction protein beta 4
1,013	ENSG00000126603	GLIS2	GLIS family zinc finger 2
1,869	ENSG00000107249	GLIS3	GLIS family zinc finger 3
1,003	ENSG00000237009	GLIS3-AS1	GLIS3 antisense RNA 1
1,825	ENSG00000115419	GLS	glutaminase
1,176	ENSG00000101216	GMEB2	glucocorticoid modulatory element binding protein 2
1,962	ENSG00000120063	GNA13	G protein subunit alpha 13
1,035	ENSG00000156052	GNAQ	G protein subunit alpha q
1,169	ENSG00000134697	GNL2	G protein nucleolar 2
1,655	ENSG00000215252	GOLGA8A/GOI	golgin A8 family member A
1,111	ENSG00000116580	GON4L	gon-4 like
1,235	ENSG00000138678	GPAT3	glycerol-3-phosphate acyltransferase 3
1,009	ENSG00000158669	GPAT4	glycerol-3-phosphate acyltransferase 4
1,377	ENSG00000186566	GPATCH8	G-patch domain containing 8
1,28	ENSG00000062194	GPBP1	GC-rich promoter binding protein 1
1,064	ENSG00000115159	GPD2	glycerol-3-phosphate dehydrogenase 2
2,294	ENSG00000166073	GPR176	G protein-coupled receptor 176
1,004	ENSG00000170837	GPR27	G protein-coupled receptor 27
1,277	ENSG00000181773	GPR3	G protein-coupled receptor 3
3,516	ENSG00000013588	GPRC5A	G protein-coupled receptor class C group 5 member A
2,653	ENSG00000167191	GPRC5B	G protein-coupled receptor class C group 5 member B
1,938	ENSG00000023171	GRAMD1B	GRAM domain containing 1B
1,021	ENSG00000106070	GRB10	growth factor receptor bound protein 10
2,892	ENSG00000141738	GRB7	growth factor receptor bound protein 7
2,021	ENSG00000083307	GRHL2	grainyhead like transcription factor 2
1,046	ENSG00000084207	GSTP1	glutathione S-transferase pi 1
2,297	ENSG00000100226	GTPBP1	GTP binding protein 1
1,144	ENSG00000107937	GTPBP4	GTP binding protein 4
1,239	ENSG00000197273	GUCA2A	guanylate cyclase activator 2A
1,03	ENSG00000144366	GULP1	GULP, engulfment adaptor PTB domain containing 1
1,437	ENSG00000228315	GUSBP11	glucuronidase, beta pseudogene 11
1,126	ENSG00000253203	GUSBP3	glucuronidase, beta pseudogene 3
1,276	ENSG00000184897	H1FX	H1 histone family member X
1,813	ENSG00000188486	H2AFX	H2A histone family member X
1,393	ENSG00000132475	H3F3A/H3F3B	H3 histone family member 3A
1,365	ENSG00000188375	H3F3C	H3 histone family member 3C
1,467	ENSG00000140511	HAPLN3	hyaluronan and proteoglycan link protein 3
1,089	ENSG00000103044	HAS3	hyaluronan synthase 3
4,913	ENSG00000113070	HBEGF	heparin binding EGF like growth factor
1,097	ENSG00000061273	HDAC7	histone deacetylase 7
2,059	ENSG00000048052	HDAC9	histone deacetylase 9
2,365	ENSG00000230267	HERC2P4	hect domain and RLD 2 pseudogene 4
1,942	ENSG00000188290	HES4	hes family bHLH transcription factor 4
1,537	ENSG00000186834	HEXIM1	hexamethylene bisacetamide inducible 1
1,201	ENSG00000164683	HEY1	hes related family bHLH transcription factor with YRPW motif 1
1,209	ENSG00000185359	HGS	hepatocyte growth factor-regulated tyrosine kinase substrate
1,468	ENSG00000100644	HIF1A	hypoxia inducible factor 1 alpha subunit
1,796	ENSG00000130787	HIP1R	huntingtin interacting protein 1 related
1,224	ENSG00000124635	HIST1H2BJ	histone cluster 1 H2B family member j
2,215	ENSG00000233822	HIST1H2BN	histone cluster 1 H2B family member n
2,188	ENSG00000197409	HIST1H3D	histone cluster 1 H3 family member d
1,388	ENSG00000196890	HIST3H2BB	histone cluster 3 H2B family member b
1,476	ENSG00000095951	HIVEP1	human immunodeficiency virus type I enhancer binding protein 1
2,465	ENSG00000010818	HIVEP2	human immunodeficiency virus type I enhancer binding protein 2
1,135	ENSG00000159399	HK2	hexokinase 2
1,557	ENSG00000156510	HKDC1	hexokinase domain containing 1
3,751	ENSG00000137309	HMGA1	high mobility group AT-hook 1
1,522	ENSG00000113161	HMGCR	3-hydroxy-3-methylglutaryl-CoA reductase
1,363	ENSG00000112972	HMGCS1	3-hydroxy-3-methylglutaryl-CoA synthase 1
1,017	ENSG00000113716	HMGXB3	HMG-box containing 3
1,148	ENSG00000177733	HNRNPA0	heterogeneous nuclear ribonucleoprotein A0
1,046	ENSG00000138668	HNRNPD	heterogeneous nuclear ribonucleoprotein D
1,539	ENSG00000169045	HNRNPH1	heterogeneous nuclear ribonucleoprotein H1
1,502	ENSG00000096746	HNRNPH3	heterogeneous nuclear ribonucleoprotein H3
1,111	ENSG00000099783	HNRNPM	heterogeneous nuclear ribonucleoprotein M
1,943	ENSG00000153187	HNRNPU	heterogeneous nuclear ribonucleoprotein U
1,408	ENSG00000152413	HOMER1	homer scaffolding protein 1
6,273	ENSG00000257017	HP	haptoglobin
1,638	ENSG00000002587	HS3ST1	heparan sulfate-glucosamine 3-sulfotransferase 1
1,6	ENSG00000162040	HS3ST6	heparan sulfate-glucosamine 3-sulfotransferase 6
1,362	ENSG00000102878	HSF4	heat shock transcription factor 4
3,125	ENSG00000080824	HSP90AA1	heat shock protein 90 alpha family class A member 1
3,039	ENSG00000096384	HSP90AB1	heat shock protein 90 alpha family class B member 1
1,826	ENSG00000205940	HSP90AB2P	heat shock protein 90 alpha family class B member 2, pseudogene
1,388	ENSG00000126803	HSPA2	heat shock protein family A (Hsp70) member 2
1,523	ENSG00000170606	HSPA4	heat shock protein family A (Hsp70) member 4
1,704	ENSG00000164070	HSPA4L	heat shock protein family A (Hsp70) member 4 like
1,747	ENSG00000044574	HSPA5	heat shock protein family A (Hsp70) member 5
7,401	ENSG00000173110	HSPA6	heat shock protein family A (Hsp70) member 6
7,092	ENSG00000225217	HSPA7	heat shock protein family A (Hsp70) member 7
2,553	ENSG00000109971	HSPA8	heat shock protein family A (Hsp70) member 8
1,785	ENSG00000144381	HSPD1	heat shock protein family D (Hsp60) member 1
5,563	ENSG00000120694	HSPH1	heat shock protein family H (Hsp110) member 1
1,853	ENSG00000142149	HUNK	hormonally up-regulated Neu-associated kinase
1,095	ENSG00000157423	HYDIN	HYDIN, axonemal central pair apparatus protein
1,289	ENSG00000163596	ICA1L	islet cell autoantigen 1 like

3,227	ENSG00000162413	KLHL21	kelch like family member 21
1,503	ENSG00000119771	KLHL29	kelch like family member 29
1,493	ENSG00000129451	KLK10	kallikrein related peptidase 10
1,814	ENSG00000167757	KLK11	kallikrein related peptidase 11
1,082	ENSG00000005483	KMT2E	lysine methyltransferase 2E
1,023	ENSG00000171346	KRT15	keratin 15
4,214	ENSG00000128422	KRT17	keratin 17
1,259	ENSG00000111057	KRT18	keratin 18
2,836	ENSG00000171345	KRT19	keratin 19
1,097	ENSG00000185479	KRT6B	keratin 6B
2,644	ENSG00000135480	KRT7	keratin 7
1,573	ENSG00000170421	KRT8	keratin 8
2,57	ENSG00000167767	KRT80	keratin 80
1,931	ENSG00000205426	KRT81	keratin 81
1,802	ENSG00000170442	KRT86	keratin 86
1,158	ENSG00000187026	KRTAP21-2	keratin associated protein 21-2
1,066	ENSG00000157992	KRTCAP3	keratinocyte associated protein 3
2,084	ENSG00000159166	LAD1	ladinin 1
1,436	ENSG00000101680	LAMA1	laminin subunit alpha 1
1,326	ENSG00000053747	LAMA3	laminin subunit alpha 3
2,175	ENSG00000130702	LAMA5	laminin subunit alpha 5
1,504	ENSG00000172037	LAMB2	laminin subunit beta 2
2,997	ENSG00000196878	LAMB3	laminin subunit beta 3
2,117	ENSG00000135862	LAMC1	laminin subunit gamma 1
3,697	ENSG00000058085	LAMC2	laminin subunit gamma 2
1,573	ENSG00000165905	LARGE2	LARGE xylosyl- and glucuronyltransferase 2
1,961	ENSG00000166173	LARP6	La ribonucleoprotein domain family member 6
1,186	ENSG00000131023	LATS1	large tumor suppressor kinase 1
1,061	ENSG00000204381	LAYN	layilin
1,433	ENSG00000135338	LCA5	LCA5, lebercilin
1,622	ENSG00000148346	LCN2	lipocalin 2
1,047	ENSG00000243709	LEFTY1	left-right determination factor 1
1,085	ENSG00000174106	LEMD3	LEM domain containing 3
1,154	ENSG00000100079	LGALS2	galectin 2
1,342	ENSG00000145685	LHFPL2	lipoma HMGIC fusion partner-like 2
3,96	ENSG00000128342	LIF	LIF, interleukin 6 family cytokine
1,055	ENSG00000182541	LIMK2	LIM domain kinase 2
1,8	ENSG00000225880	LINC00115	long intergenic non-protein coding RNA 115
1,126	ENSG00000231028	LINC00271	long intergenic non-protein coding RNA 271
1,14	ENSG00000233237	LINC00472	long intergenic non-protein coding RNA 472
1,405	ENSG00000258169	LINC00485	long intergenic non-protein coding RNA 485
1,428	ENSG00000163898	LIPH	lipase H
1,797	ENSG00000073350	LLGL2	LLGL2, scribble cell polarity complex component
1,173	ENSG00000154359	LONRF1	LON peptidase N-terminal domain and ring finger 1
1,669	ENSG00000064547	LPAR2	lysophosphatidic acid receptor 2
1,837	ENSG00000139263	LRIG3	leucine rich repeats and immunoglobulin like domains 3
1,369	ENSG00000137269	LRRC1	leucine rich repeat containing 1
1,426	ENSG00000148814	LRRC27	leucine rich repeat containing 27
1,22	ENSG00000159708	LRRC36	leucine rich repeat containing 36
1,403	ENSG00000178026	LRRC75B	leucine rich repeat containing 75B
1,438	ENSG00000171017	LRRC8E	leucine rich repeat containing 8 family member E
2,434	ENSG00000175928	LRRN1	leucine rich repeat neuronal 1
1,692	ENSG00000125872	LRRN4	leucine rich repeat neuronal 4
1,296	ENSG00000007392	LUC7L	LUC7 like
1,241	ENSG00000153714	LURAP1L	leucine rich adaptor protein 1 like
1,69	ENSG00000150556	LYPD6B	LY6/PLAUR domain containing 6B
1,414	ENSG00000181541	MAB21L2	mab-21 like 2
1,089	ENSG00000173212	MAB21L3	mab-21 like 3
2,691	ENSG00000183742	MACC1	MACC1, MET transcriptional regulator
3,138	ENSG00000185022	MAFF	MAF bZIP transcription factor F
2,165	ENSG00000197063	MAFG	MAF bZIP transcription factor G
1,098	ENSG00000265688	MAFG-AS1	MAFG antisense RNA 1 (head to head)
1,949	ENSG00000198517	MAFK	MAF bZIP transcription factor K
1,362	ENSG00000187243	MAGED4/M	MAGE family member D4B
1,559	ENSG00000151276	MAGI1	membrane associated guanylate kinase, WW and PDZ domain conta
1,996	ENSG00000251562	MALAT1	metastasis associated lung adenocarcinoma transcript 1 (non-prote
1,278	ENSG00000184384	MAML2	mastermind like transcriptional coactivator 2
1,327	ENSG00000197769	MAP1LC3C	microtubule associated protein 1 light chain 3 gamma
1,492	ENSG00000130479	MAP1S	microtubule associated protein 1S
1,498	ENSG00000078018	MAP2	microtubule associated protein 2
1,157	ENSG00000034152	MAP2K3	mitogen-activated protein kinase kinase 3
1,524	ENSG00000095015	MAP3K1	mitogen-activated protein kinase kinase kinase 1
1,795	ENSG00000006062	MAP3K14	mitogen-activated protein kinase kinase kinase 14
2,519	ENSG00000107968	MAP3K8	mitogen-activated protein kinase kinase kinase 8
1,738	ENSG00000006432	MAP3K9	mitogen-activated protein kinase kinase kinase 9
1,027	ENSG00000047849	MAP4	microtubule associated protein 4
1,35	ENSG00000116871	MAP7D1	MAP7 domain containing 1
1,197	ENSG00000156711	MAPK13	mitogen-activated protein kinase 13
1,688	ENSG00000138834	MAPK8IP3	mitogen-activated protein kinase 8 interacting protein 3
1,198	ENSG00000173926	MARCH3	membrane associated ring-CH-type finger 3
1,385	ENSG00000145495	MARCH6	membrane associated ring-CH-type finger 6
1,464	ENSG00000175130	MARCKSL1	MARCKS like 1
1,133	ENSG00000072518	MARK2	microtubule affinity regulating kinase 2
1,031	ENSG00000140832	MARVELD3	MARVEL domain containing 3
1,136	ENSG00000164430	MB21D1	Mab-21 domain containing 1
1,057	ENSG00000166987	MBD6	methyl-CpG binding domain protein 6
1,11	ENSG00000172197	MBOAT1	membrane bound O-acyltransferase domain containing 1
2,004	ENSG00000177669	MBOAT4	membrane bound O-acyltransferase domain containing 4
1,199	ENSG00000258839	MC1R	melanocortin 1 receptor
1,568	ENSG00000076706	MCAM	melanoma cell adhesion molecule
1,158	ENSG00000126217	MCF2L	MCF.2 cell line derived transforming sequence like
2,387	ENSG00000143384	MCL1	MCL1, BCL2 family apoptosis regulator
1,202	ENSG00000055732	MCOLN3	mucolipin 3
1,081	ENSG00000156026	MCU	mitochondrial calcium uniporter
1,347	ENSG00000110492	MDK	midkine (neurite growth-promoting factor 2)
1,061	ENSG00000198625	MDM4	MDM4, p53 regulator
1,3	ENSG00000151376	ME3	malic enzyme 3
1,117	ENSG00000099917	MED15	mediator complex subunit 15
1,808	ENSG00000163975	MELTF	melanotransferrin
1,145	ENSG00000140406	MESDC1	mesoderm development candidate 1
1,068	ENSG00000166823	MESP1	mesoderm posterior bHLH transcription factor 1
1,178	ENSG00000105976	MET	MET proto-oncogene, receptor tyrosine kinase
2,038	ENSG00000214756	METTL12	methyltransferase like 12
1,04	ENSG00000144401	METTL21A	methyltransferase like 21A
1,495	ENSG00000176624	MEX3C	mex-3 RNA binding family member C
1,01	ENSG00000147324	MFHAS1	malignant fibrous histiocytoma amplified sequence 1
1,236	ENSG00000151690	MFSD6	major facilitator superfamily domain containing 6
1,086	ENSG00000185156	MFSD6L	major facilitator superfamily domain containing 6 like
1,789	ENSG00000243156	MICAL3	microtubule associated monooxygenase, calponin and LIM domain (
1,35	ENSG00000167470	MIDN	midnolin
1,28	ENSG00000266192	mir-1260b	microRNA 1260b
4,592	ENSG00000207614	mir-193	microRNA 193a
6,182	ENSG00000207980	mir-23	microRNA 23a
7,78	ENSG00000207617	mir-3074	microRNA 3074
6,127	ENSG00000263628	mir-3155	microRNA 3155a
6,805	ENSG00000199023	mir-339	microRNA 339
4,279	ENSG00000264462	mir-3648	microRNA 3648-1
6,201	ENSG00000199032	mir-425	microRNA 425
3,211	ENSG00000207589	mir-506	microRNA 508
4,469	ENSG00000207946	mir-515	microRNA 520c
2,186	ENSG00000207650	mir-548	microRNA 579
5,715	ENSG00000207716	mir-572	microRNA 572
6,782	ENSG00000207561	mir-635	microRNA 635
3,98	ENSG00000207703	mir-7	microRNA 7-1
1,054	ENSG00000215417	MIR17HG	miR-17-92a-1 cluster host gene
7,535	ENSG00000266533	MIR3619	microRNA 3619
3,905	ENSG00000265558	MIR3918	microRNA 3918
3,744	ENSG00000265442	MIR3941	microRNA 3941
5,905	ENSG00000264171	MIR4305	microRNA 4305
4,259	ENSG00000263583	MIR4522	microRNA 4522
6,184	ENSG00000263834	MIR4635	microRNA 4635
6,799	ENSG00000263575	MIR4665	microRNA 4665
2,27	ENSG00000099812	MISP	mitotic spindle positioning
1,982	ENSG00000178053	MLF1	myeloid leukemia factor 1
1,33	ENSG00000171843	MLLT3	MLLT3, super elongation complex subunit
2,82	ENSG00000090339	ICAM1	intercellular adhesion molecule 1
1,803	ENSG00000105371	ICAM4	intercellular adhesion molecule 4 (Landsteiner-Wiener blood group)
1,298	ENSG00000172201	ID4	inhibitor of DNA binding 4, HLH protein
1,984	ENSG00000232656	IDI2-AS1	IDI2 antisense RNA 1
1,455	ENSG00000127415	IDUA	iduronidase, alpha-L-
2,492	ENSG00000160888	IER2	immediate early response 2
3,46	ENSG00000162783	IER5	immediate early response 5
1,696	ENSG00000169991	IFFO2	intermediate filament family orphan 2
1,274	ENSG00000111537	IFNG	interferon gamma
1,983	ENSG00000006652	IFRD1	interferon related developmental regulator 1
1,253	ENSG00000187535	IFT140	intraflagellar transport 140
1,667	ENSG00000073792	IGF2BP2	insulin like growth factor 2 mRNA binding protein 2
1,707	ENSG00000143061	IGSF3	immunoglobulin superfamily member 3
1,349	ENSG00000150782	IL18	interleukin 18
1,206	ENSG00000115598	IL1RL2	interleukin 1 receptor like 2
3,618	ENSG00000110944	IL23A	interleukin 23 subunit alpha
1,298	ENSG00000077238	IL4R	interleukin 4 receptor
5,066	ENSG00000136244	IL6	interleukin 6
2,081	ENSG00000160712	IL6R	interleukin 6 receptor
1,123	ENSG00000145103	ILDR1	immunoglobulin like domain containing receptor 1
1,109	ENSG00000143621	ILF2	interleukin enhancer binding factor 2
1,561	ENSG00000129351	ILF3	interleukin enhancer binding factor 3
1,344	ENSG00000203485	INF2	inverted formin, FH2 and WH2 domain containing
2,306	ENSG00000122641	INHBA	inhibin beta A subunit
1,173	ENSG00000148384	INPP5E	inositol polyphosphate-5-phosphatase E
1,086	ENSG00000164066	INTU	inturned planar cell polarity protein
1,071	ENSG00000176095	IP6K1	inositol hexakisphosphate kinase 1
2,005	ENSG00000132321	IQCA1	IQ motif containing with AAA domain 1
1,36	ENSG00000174628	IQCK	IQ motif containing K
1,357	ENSG00000140575	IQGAP1	IQ motif containing GTPase activating protein 1
2,992	ENSG00000134070	IRAK2	interleukin 1 receptor associated kinase 2
1,783	ENSG00000125347	IRF1	interferon regulatory factor 1
1,349	ENSG00000168264	IRF2BP2	interferon regulatory factor 2 binding protein 2
1,647	ENSG00000117595	IRF6	interferon regulatory factor 6
1,144	ENSG00000167378	IRGQ	immunity related GTPase Q
2,171	ENSG00000185950	IRS2	insulin receptor substrate 2
1,603	ENSG00000105655	ISYNA1	inositol-3-phosphate synthase 1
2,25	ENSG00000164171	ITGA2	integrin subunit alpha 2
2,094	ENSG00000005884	ITGA3	integrin subunit alpha 3
1,799	ENSG00000161638	ITGA5	integrin subunit alpha 5
1,56	ENSG00000091409	ITGA6	integrin subunit alpha 6
1,199	ENSG00000169896	ITGAM	integrin subunit alpha M
1,401	ENSG00000138448	ITGAV	integrin subunit alpha V
2,037	ENSG00000132470	ITGB4	integrin subunit beta 4
1,85	ENSG00000115221	ITGB6	integrin subunit beta 6
2,671	ENSG00000105855	ITGB8	integrin subunit beta 8
1,449	ENSG00000123243	ITIH5	inter-alpha-trypsin inhibitor heavy chain family member 5
3,934	ENSG00000086544	ITPKC	inositol-trisphosphate 3-kinase C
1,418	ENSG00000096433	ITPR3	inositol 1,4,5-trisphosphate receptor type 3
1,12	ENSG00000008083	JARID2	jumonji and AT-rich interaction domain containing 2
1,286	ENSG00000171988	JMJD1C	jumonji domain containing 1C
2,052	ENSG00000070495	JMJD6	arginine demethylase and lysine hydroxylase
1,037	ENSG00000183340	JRKL	JRK-like
3,48	ENSG00000177606	JUN	Jun proto-oncogene, AP-1 transcription factor subunit
1,84	ENSG00000171223	JUNB	JunB proto-oncogene, AP-1 transcription factor subunit
3,047	ENSG00000130522	JUND	JunD proto-oncogene, AP-1 transcription factor subunit
2,578	ENSG00000146049	KAAG1	kidney associated antigen 1
1,42	ENSG00000170852	KBTBD2	kelch repeat and BTB domain containing 2
1,095	ENSG00000120696	KBTBD7	kelch repeat and BTB domain containing 7
1,614	ENSG00000162975	KCNF1	potassium voltage-gated channel modifier subfamily F member 1
1,353	ENSG00000135750	KCNK1	potassium two pore domain channel subfamily K member 1
1,678	ENSG00000164626	KCNK5	potassium two pore domain channel subfamily K member 5
1,218	ENSG00000104783	KCNN4	potassium calcium-activated channel subfamily N member 4
1,185	ENSG00000213859	KCTD11	potassium channel tetramerization domain containing 11
1,415	ENSG00000174943	KCTD13	potassium channel tetramerization domain containing 13
1,221	ENSG00000104756	KCTD9	potassium channel tetramerization domain containing 9
1,758	ENSG00000173120	KDM2A	lysine demethylase 2A
2,531	ENSG00000117139	KDM5B	lysine demethylase 5B
1,72	ENSG00000147050	KDM6A	lysine demethylase 6A
2,143	ENSG00000132510	KDM6B	lysine demethylase 6B
1,058	ENSG00000100441	KHNYN	KH and NYN domain containing
1,341	ENSG00000047578	KIAA0556	KIAA0556
1,24	ENSG00000164542	KIAA0895	KIAA0895
1,615	ENSG00000196123	KIAA0895L	KIAA0895 like
1,286	ENSG00000120549	KIAA1217	KIAA1217
1,688	ENSG00000162522	KIAA1522	KIAA1522
2,526	ENSG00000136883	KIF12	kinesin family member 12
1,068	ENSG00000131437	KIF3A	kinesin family member 3A
1,433	ENSG00000167702	KIFC2	kinesin family member C2
1,071	ENSG00000151657	KIN	Kin17 DNA and RNA binding protein
1,339	ENSG00000174996	KLC2	kinesin light chain 2
2,097	ENSG00000104892	KLC3	kinesin light chain 3
2,057	ENSG00000155090	KLF10	Kruppel like factor 10
2,596	ENSG00000127528	KLF2	Kruppel like factor 2
1,361	ENSG00000109787	KLF3	Kruppel like factor 3
2,628	ENSG00000136826	KLF4	Kruppel like factor 4
4,299	ENSG00000102554	KLF5	Kruppel like factor 5
3,757	ENSG00000067082	KLF6	Kruppel like factor 6
1,576	ENSG00000118263	KLF7	Kruppel like factor 7
1,107	ENSG00000174010	KLHL15	kelch like family member 15
1,216	ENSG00000187961	KLHL17	kelch like family member 17
4,872	ENSG00000162413	KLHL21	kelch like family member 21
1,086	ENSG00000183655	KLHL25	kelch like family member 25
1,499	ENSG00000119771	KLHL29	kelch like family member 29
1,719	ENSG00000129451	KLK10	kallikrein related peptidase 10
2,033	ENSG00000167757	KLK11	kallikrein related peptidase 11
1,026	ENSG00000256667	KLRA1P	killer cell lectin like receptor A1, pseudogene
1,203	ENSG00000005483	KMT2E	lysine methyltransferase 2E
1,307	ENSG00000185467	KPNA7	karyopherin subunit alpha 7
1,163	ENSG00000171346	KRT15	keratin 15
4,013	ENSG00000128422	KRT17	keratin 17
1,454	ENSG00000111057	KRT18	keratin 18
3,172	ENSG00000171345	KRT19	keratin 19
2,843	ENSG00000135480	KRT7	keratin 7
1,897	ENSG00000170421	KRT8	keratin 8
2,939	ENSG00000167767	KRT80	keratin 80
1,201	ENSG00000205426	KRT81	keratin 81
1,32	ENSG00000170442	KRT86	keratin 86
1,053	ENSG00000157992	KRTCAP3	keratinocyte associated protein 3
2,217	ENSG00000159166	LAD1	ladinin 1
1,243	ENSG00000101680	LAMA1	laminin subunit alpha 1
1,598	ENSG00000053747	LAMA3	laminin subunit alpha 3
2,272	ENSG00000130702	LAMA5	laminin subunit alpha 5
1,563	ENSG00000172037	LAMB2	laminin subunit beta 2
3,983	ENSG00000196878	LAMB3	laminin subunit beta 3
2,43	ENSG00000135862	LAMC1	laminin subunit gamma 1
5,145	ENSG00000058085	LAMC2	laminin subunit gamma 2
1,524	ENSG00000165905	LARGE2	LARGE xylosyl- and glucuronyltransferase 2
1,689	ENSG00000166173	LARP6	La ribonucleoprotein domain family member 6
1,309	ENSG00000131023	LATS1	large tumor suppressor kinase 1
1,001	ENSG00000204381	LAYN	layilin
1,443	ENSG00000143815	LBR	lamin B receptor
1,258	ENSG00000135338	LCA5	LCA5, lebercilin
1,094	ENSG00000148346	LCN2	lipocalin 2
1,309	ENSG00000243709	LEFTY1	left-right determination factor 1
1,222	ENSG00000174106	LEMD3	LEM domain containing 3
4,232	ENSG00000199133	let-7	microRNA let-7a-1
1,141	ENSG00000178934	LGALS7/LGALS	galectin 7
2,01	ENSG00000145685	LHFPL2	lipoma HMGIC fusion partner-like 2
4,578	ENSG00000128342	LIF	LIF, interleukin 6 family cytokine
1,446	ENSG00000225880	LINC00115	long intergenic non-protein coding RNA 115
1,345	ENSG00000233237	LINC00472	long intergenic non-protein coding RNA 472
1,038	ENSG00000258169	LINC00485	long intergenic non-protein coding RNA 485
1,453	ENSG00000101670	LIPG	lipase G, endothelial type
2,005	ENSG00000163898	LIPH	lipase H

2,395	ENSG00000196611	MMP1	matrix metallopeptidase 1
1,701	ENSG00000166670	MMP10	matrix metallopeptidase 10
1,057	ENSG00000157227	MMP14	matrix metallopeptidase 14
1,259	ENSG00000102996	MMP15	matrix metallopeptidase 15
1,368	ENSG00000123342	MMP19	matrix metallopeptidase 19
1,195	ENSG00000125966	MMP24	matrix metallopeptidase 24
2,578	ENSG00000137673	MMP7	matrix metallopeptidase 7
1,124	ENSG00000130675	MNX1	motor neuron and pancreas homeobox 1
1,446	ENSG00000120162	MOB3B	MOB kinase activator 3B
1,216	ENSG00000161647	MPP3	membrane palmitoylated protein 3
1,061	ENSG00000133030	MPRIP	myosin phosphatase Rho interacting protein
1,382	ENSG00000160588	MPZL3	myelin protein zero like 3
1,037	ENSG00000174579	MSL2	male-specific lethal 2 homolog (Drosophila)
1,649	ENSG00000163132	MSX1	msh homeobox 1
1,109	ENSG00000120149	MSX2	msh homeobox 2
1,797	ENSG00000256618	MTRNR2L1	MT-RNR2-like 1
1,654	ENSG00000256045	MTRNR2L1(	MT-RNR2-like 10
1,672	ENSG00000271043	MTRNR2L2	MT-RNR2-like 2
1,627	ENSG00000255823	MTRNR2L8	MT-RNR2-like 8
1,552	ENSG00000185499	MUC1	mucin 1, cell surface associated
1,553	ENSG00000176945	MUC20	mucin 20, cell surface associated
1,17	ENSG00000117983	MUC5B	mucin 5B, oligomeric mucus/gel-forming
3,706	ENSG00000184956	MUC6	mucin 6, oligomeric mucus/gel-forming
1,638	ENSG00000157502	MUM1L1	MUM1 like 1
2,732	ENSG00000059728	MXD1	MAX dimerization protein 1
2,2	ENSG00000179820	MYADM	myeloid associated differentiation marker
1,069	ENSG00000132382	MYBBP1A	MYB binding protein 1a
2,293	ENSG00000136997	MYC	MYC proto-oncogene, bHLH transcription factor
1,443	ENSG00000104177	MYEF2	myelin expression factor 2
1,423	ENSG00000105357	MYH14	myosin heavy chain 14
1,785	ENSG00000100345	MYH9	myosin heavy chain 9
2,022	ENSG00000007944	MYLIP	myosin regulatory light chain interacting protein
1,981	ENSG00000145555	MYO10	myosin X
1,007	ENSG00000197879	MYO1C	myosin IC
1,064	ENSG00000157483	MYO1E	myosin IE
1,324	ENSG00000095777	MYO3A	myosin IIIA
1,336	ENSG00000128833	MYO5C	myosin VC
1,155	ENSG00000196586	MYO6	myosin VI
1,196	ENSG00000138119	MYOF	myoferlin
1,202	ENSG00000263155	MYZAP	myocardial zonula adherens protein
1,396	ENSG00000099326	MZF1	myeloid zinc finger 1
1,46	ENSG00000172766	NAA16	N(alpha)-acetyltransferase 16, NatA auxiliary subunit
1,088	ENSG00000110583	NAA40	N(alpha)-acetyltransferase 40, NatD catalytic subunit
1,472	ENSG00000177694	NAALADL2	N-acetylated alpha-linked acidic dipeptidase like 2
1,111	ENSG00000172915	NBEA	neurobeachin
1,397	ENSG00000160796	NBEAL2	neurobeachin like 2
3,119	ENSG00000144959	NCEH1	neutral cholesterol ester hydrolase 1
1,98	ENSG00000071051	NCK2	NCK adaptor protein 2
2,972	ENSG00000111912	NCOA7	nuclear receptor coactivator 7
1,118	ENSG00000141027	NCOR1	nuclear receptor corepressor 1
1,536	ENSG00000245532	NEAT1	nuclear paraspeckle assembly transcript 1 (non-protein coding)
1,306	ENSG00000130202	NECTIN2	nectin cell adhesion molecule 2
2,153	ENSG00000143217	NECTIN4	nectin cell adhesion molecule 4
1,516	ENSG00000049759	NEDD4L	neural precursor cell expressed, developmentally down-regulated 4
2,514	ENSG00000111859	NEDD9	neural precursor cell expressed, developmentally down-regulated 9
1,026	ENSG00000114670	NEK11	NIMA related kinase 11
1,029	ENSG00000160602	NEK8	NIMA related kinase 8
1,238	ENSG00000067141	NEO1	neogenin 1
1,215	ENSG00000173848	NET1	neuroepithelial cell transforming 1
2,183	ENSG00000163121	NEURL3	neuralized E3 ubiquitin protein ligase 3
1,374	ENSG00000102908	NFAT5	nuclear factor of activated T-cells 5
2,113	ENSG00000131196	NFATC1	nuclear factor of activated T-cells 1
1,915	ENSG00000050344	NFE2L3	nuclear factor, erythroid 2 like 3
1,484	ENSG00000165030	NFIL3	nuclear factor, interleukin 3 regulated
1,517	ENSG00000109320	NFKB1	nuclear factor kappa B subunit 1
1,655	ENSG00000077150	NFKB2	nuclear factor kappa B subunit 2
1,589	ENSG00000167604	NFKBID	NFKB inhibitor delta
2,028	ENSG00000146232	NFKBIE	NFKB inhibitor epsilon
4,124	ENSG00000144802	NFKBIZ	NFKB inhibitor zeta
1,387	ENSG00000001167	NFYA	nuclear transcription factor Y subunit alpha
1,089	ENSG00000101004	NINL	ninein like
1,114	ENSG00000167034	NKX3-1	NK3 homeobox 1
1,116	ENSG00000165246	NLGN4Y	neuroligin 4, Y-linked
1,597	ENSG00000197696	NMB	neuromedin B
2,061	ENSG00000112981	NME5	NME/NM23 family member 5
1,374	ENSG00000151014	NOCT	nocturnin
1,501	ENSG00000188747	NOXA1	NADPH oxidase activator 1
1,161	ENSG00000170485	NPAS2	neuronal PAS domain protein 2
2,111	ENSG00000141458	NPC1	NPC intracellular cholesterol transporter 1
1,154	ENSG00000015520	NPC1L1	NPC1 like intracellular cholesterol transporter 1
1,164	ENSG00000215440	NPEPL1	aminopeptidase-like 1
1,201	ENSG00000141279	NPEPPS	aminopeptidase puromycin sensitive
1,314	ENSG00000183426	NPIPA7 (inc	nuclear pore complex interacting protein family member A7
1,601	ENSG00000169246	NPIPB5 (inc	nuclear pore complex interacting protein family member B4
1,683	ENSG00000168743	NPNT	nephronectin
1,551	ENSG00000106236	NPTX2	neuronal pentraxin 2
1,606	ENSG00000131910	NR0B2	nuclear receptor subfamily 0 group B member 2
1,249	ENSG00000126368	NR1D1	nuclear receptor subfamily 1 group D member 1
1,974	ENSG00000123358	NR4A1	nuclear receptor subfamily 4 group A member 1
1,052	ENSG00000153234	NR4A2	nuclear receptor subfamily 4 group A member 2
1,869	ENSG00000198435	NRARP	NOTCH-regulated ankyrin repeat protein
1,08	ENSG00000091129	NRCAM	neuronal cell adhesion molecule
1,378	ENSG00000157168	NRG1	neuregulin 1
1,199	ENSG00000180530	NRIP1	nuclear receptor interacting protein 1
1,014	ENSG00000147548	NSD3	nuclear receptor binding SET domain protein 3
1,537	ENSG00000179299	NSUN7	NOP2/Sun RNA methyltransferase family member 7
1,044	ENSG00000074527	NTN4	netrin 4
4,455	ENSG00000163545	NUAK2	NUAK family kinase 2
1,207	ENSG00000108256	NUFIP2	NUFIP2, FMR1 interacting protein 2
1,511	ENSG00000137497	NUMA1	nuclear mitotic apparatus protein 1
1,372	ENSG00000137804	NUSAP1	nucleolar and spindle associated protein 1
1,334	ENSG00000162231	NXF1	nuclear RNA export factor 1
1,049	ENSG00000167693	NXN	nucleoredoxin
1,601	ENSG00000124006	OBSL1	obscurin like 1
1,142	ENSG00000122417	ODF2L	outer dense fiber of sperm tails 2 like
1,332	ENSG00000046651	OFD1	OFD1, centriole and centriolar satellite protein
1,627	ENSG00000173391	OLR1	oxidized low density lipoprotein receptor 1
2,071	ENSG00000169856	ONECUT1	one cut homeobox 1
1,813	ENSG00000079482	OPHN1	oligophrenin 1
1,264	ENSG00000177535	OR2B11	olfactory receptor family 2 subfamily B member 11
1,163	ENSG00000115947	ORC4	origin recognition complex subunit 4
1,644	ENSG00000165312	OTUD1	OTU deubiquitinase 1
1,084	ENSG00000189401	OTUD6A	OTU deubiquitinase 6A
2,736	ENSG00000172818	OVOL1	ovo like transcriptional repressor 1
2,629	ENSG00000180914	OXTR	oxytocin receptor
1,541	ENSG00000169860	P2RY1	purinergic receptor P2Y1
1,42	ENSG00000101104	PABPC1L	poly(A) binding protein cytoplasmic 1 like
1,223	ENSG00000100836	PABPN1	poly(A) binding protein nuclear 1
1,283	ENSG00000165912	PACSIN3	protein kinase C and casein kinase substrate in neurons 3
1,288	ENSG00000077264	PAK3	p21 (RAC1) activated kinase 3
1,634	ENSG00000187867	PALM3	paralemmin 3
1,185	ENSG00000135473	PAN2	PAN2 poly(A) specific ribonuclease subunit
1,026	ENSG00000152520	PAN3	PAN3 poly(A) specific ribonuclease subunit
1,697	ENSG00000100767	PAPLN	papilin, proteoglycan like sulfated glycoprotein
1,346	ENSG00000138801	PAPSS1	3'-phosphoadenosine 5'-phosphosulfate synthase 1
1,027	ENSG00000162073	PAQR4	progestin and adipoQ receptor family member 4
1,475	ENSG00000137819	PAQR5	progestin and adipoQ receptor family member 5
1,07	ENSG00000148498	PARD3	par-3 family cell polarity regulator
1,088	ENSG00000116117	PARD3B	par-3 family cell polarity regulator beta
2,084	ENSG00000124171	PARD6B	par-6 family cell polarity regulator beta
1,203	ENSG00000041880	PARP3	poly(ADP-ribose) polymerase family member 3
1,739	ENSG00000132849	PATJ	PATJ, crumbs cell polarity complex component
1,31	ENSG00000177425	PAWR	pro-apoptotic WT1 regulator
1,085	ENSG00000120327	PCDHB14	protocadherin beta 14
1,524	ENSG00000165494	PCF11	PCF11 cleavage and polyadenylation factor subunit
1,57	ENSG00000073350	LLGL2	LLGL2, scribble cell polarity complex component
1,168	ENSG00000176619	LMNB2	lamin B2
1,443	ENSG00000136153	LMO7	LIM domain 7
1,216	ENSG00000164715	LMTK2	lemur tyrosine kinase 2
1,095	ENSG00000142178	LOC10272442ε	salt inducible kinase 1
1,269	ENSG00000154359	LONRF1	LON peptidase N-terminal domain and ring finger 1
1,777	ENSG00000064547	LPAR2	lysophosphatidic acid receptor 2
1,441	ENSG00000134324	LPIN1	lipin 1
1,121	ENSG00000173621	LRFN4	leucine rich repeat and fibronectin type III domain containing 4
1,124	ENSG00000144749	LRIG1	leucine rich repeats and immunoglobulin like domains 1
1,74	ENSG00000139263	LRIG3	leucine rich repeats and immunoglobulin like domains 3
1,269	ENSG00000137269	LRRC1	leucine rich repeat containing 1
1,313	ENSG00000254402	LRRC24	leucine rich repeat containing 24
1,207	ENSG00000137821	LRRC49	leucine rich repeat containing 49
1,194	ENSG00000178026	LRRC75B	leucine rich repeat containing 75B
1,548	ENSG00000197147	LRRC8B	leucine rich repeat containing 8 family member B
1,75	ENSG00000171017	LRRC8E	leucine rich repeat containing 8 family member E
1,079	ENSG00000124831	LRRFIP1	LRR binding FLII interacting protein 1
1,218	ENSG00000093167	LRRFIP2	LRR binding FLII interacting protein 2
2,118	ENSG00000175928	LRRN1	leucine rich repeat neuronal 1
1,465	ENSG00000125872	LRRN4	leucine rich repeat neuronal 4
1,48	ENSG00000007392	LUC7L	LUC7 like
1,362	ENSG00000153714	LURAP1L	leucine rich adaptor protein 1 like
1,043	ENSG00000169641	LUZP1	leucine zipper protein 1
1,658	ENSG00000150556	LYPD6B	LY6/PLAUR domain containing 6B
1,086	ENSG00000181541	MAB21L2	mab-21 like 2
3,369	ENSG00000183742	MACC1	MACC1, MET transcriptional regulator
1,223	ENSG00000182759	MAFA	MAF bZIP transcription factor A
3,447	ENSG00000185022	MAFF	MAF bZIP transcription factor F
2,676	ENSG00000197063	MAFG	MAF bZIP transcription factor G
1,079	ENSG00000265688	MAFG-AS1	MAFG antisense RNA 1 (head to head)
2,107	ENSG00000198517	MAFK	MAF bZIP transcription factor K
1,736	ENSG00000187243	MAGED4/MAC	MAGE family member D4B
1,584	ENSG00000151276	MAGI1	membrane associated guanylate kinase, WW and PDZ domain containing 1
1,079	ENSG00000081026	MAGI3	membrane associated guanylate kinase, WW and PDZ domain containing 3
1,449	ENSG00000111837	MAK	male germ cell associated kinase
1,211	ENSG00000147676	MAL2	mal, T-cell differentiation protein 2 (gene/pseudogene)
1,786	ENSG00000251562	MALAT1	metastasis associated lung adenocarcinoma transcript 1 (non-protein coding)
1,469	ENSG00000184384	MAML2	mastermind like transcriptional coactivator 2
1,043	ENSG00000197769	MAP1LC3C	microtubule associated protein 1 light chain 3 gamma
2,102	ENSG00000130479	MAP1S	microtubule associated protein 1S
1,421	ENSG00000078018	MAP2	microtubule associated protein 2
2,128	ENSG00000034152	MAP2K3	mitogen-activated protein kinase kinase 3
1,57	ENSG00000095015	MAP3K1	mitogen-activated protein kinase kinase kinase 1
2,1	ENSG00000006062	MAP3K14	mitogen-activated protein kinase kinase kinase 14
2,795	ENSG00000107968	MAP3K8	mitogen-activated protein kinase kinase kinase 8
1,795	ENSG00000006432	MAP3K9	mitogen-activated protein kinase kinase kinase 9
1,195	ENSG00000047849	MAP4	microtubule associated protein 4
1,571	ENSG00000011566	MAP4K3	mitogen-activated protein kinase kinase kinase kinase 3
1,261	ENSG00000071054	MAP4K4	mitogen-activated protein kinase kinase kinase kinase 4
1,841	ENSG00000012983	MAP4K5	mitogen-activated protein kinase kinase kinase kinase 5
1,811	ENSG00000116871	MAP7D1	MAP7 domain containing 1
1,137	ENSG00000156711	MAPK13	mitogen-activated protein kinase 13
1,493	ENSG00000138834	MAPK8IP3	mitogen-activated protein kinase 8 interacting protein 3
1,35	ENSG00000101367	MAPRE1	microtubule associated protein RP/EB family member 1
1,232	ENSG00000173926	MARCH3	membrane associated ring-CH-type finger 3
1,629	ENSG00000145495	MARCH6	membrane associated ring-CH-type finger 6
1,354	ENSG00000175130	MARCKSL1	MARCKS like 1
1,315	ENSG00000072518	MARK2	microtubule affinity regulating kinase 2
1,063	ENSG00000140832	MARVELD3	MARVEL domain containing 3
1,054	ENSG00000086015	MAST2	microtubule associated serine/threonine kinase 2
1,251	ENSG00000069020	MAST4	microtubule associated serine/threonine kinase family member 4
1,334	ENSG00000168906	MAT2A	methionine adenosyltransferase 2A
2,178	ENSG00000177669	MBOAT4	membrane bound O-acyltransferase domain containing 4
1,647	ENSG00000076706	MCAM	melanoma cell adhesion molecule
2,731	ENSG00000143384	MCL1	MCL1, BCL2 family apoptosis regulator
1,361	ENSG00000055732	MCOLN3	mucolipin 3
1,473	ENSG00000156026	MCU	mitochondrial calcium uniporter
1,239	ENSG00000110492	MDK	midkine (neurite growth-promoting factor 2)
1,189	ENSG00000151376	ME3	malic enzyme 3
1,31	ENSG00000099917	MED15	mediator complex subunit 15
1,543	ENSG00000042429	MED17	mediator complex subunit 17
1,113	ENSG00000214548	MEG3	maternally expressed 3 (non-protein coding)
1,437	ENSG00000163975	MELTF	melanotransferrin
1,388	ENSG00000228109	MELTF-AS1	MELTF antisense RNA 1
1,184	ENSG00000140406	MESDC1	mesoderm development candidate 1
2,189	ENSG00000105976	MET	MET proto-oncogene, receptor tyrosine kinase
1,789	ENSG00000214756	METTL12	methyltransferase like 12
1,93	ENSG00000176624	MEX3C	mex-3 RNA binding family member C
1,026	ENSG00000181588	MEX3D	mex-3 RNA binding family member D
1,168	ENSG00000147324	MFHAS1	malignant fibrous histiocytoma amplified sequence 1
1,141	ENSG00000109736	MFSD10	major facilitator superfamily domain containing 10
1,095	ENSG00000151690	MFSD6	major facilitator superfamily domain containing 6
1,912	ENSG00000243156	MICAL3	microtubule associated monooxygenase, calponin and LIM domain containing 3
1,544	ENSG00000167470	MIDN	midnolin
1,094	ENSG00000128923	MINDY2	MINDY lysine 48 deubiquitinase 2
4,568	ENSG00000221634	mir-1276	microRNA 1276
2,965	ENSG00000221792	mir-1282	microRNA 1282
4,969	ENSG00000207605	mir-191	microRNA 191
4,33	ENSG00000207614	mir-193	microRNA 193a
5,977	ENSG00000207624	mir-194	microRNA 194-1
4,494	ENSG00000199038	mir-210	microRNA 210
7,178	ENSG00000207808	mir-27	microRNA 27a
2,947	ENSG00000264397	mir-3180	microRNA 3180-3
5,542	ENSG00000199053	mir-324	microRNA 324
3,98	ENSG00000199023	mir-339	microRNA 339
4,007	ENSG00000264462	mir-3648	microRNA 3648-1
2,554	ENSG00000207635	mir-499	microRNA 499a
4,359	ENSG00000207925	mir-515	microRNA 520c
6,748	ENSG00000207716	mir-572	microRNA 572
5,162	ENSG00000207756	mir-580	microRNA 580
3,655	ENSG00000207952	mir-624	microRNA 624
4,711	ENSG00000207561	mir-635	microRNA 635
2,822	ENSG00000221288	mir-663	microRNA 663a
5,46	ENSG00000198976	mir-8	microRNA 200c
1,022	ENSG00000234883	MIR155HG	MIR155 host gene
1,165	ENSG00000215417	MIR17HG	miR-17-92a-1 cluster host gene
1,909	ENSG00000186594	MIR22HG	MIR22 host gene
1,145	ENSG00000266325	MIR3665	microRNA 3665
3,327	ENSG00000263389	MIR3973	microRNA 3973
5,606	ENSG00000265682	MIR4267	microRNA 4267
5,827	ENSG00000263575	MIR4665	microRNA 4665
2,791	ENSG00000099812	MISP	mitotic spindle positioning
1,029	ENSG00000187098	MITF	melanogenesis associated transcription factor
2,727	ENSG00000178053	MLF1	myeloid leukemia factor 1
1,56	ENSG00000171843	MLLT3	MLLT3, super elongation complex subunit
1,143	ENSG00000175727	MLXIP	MLX interacting protein
3,662	ENSG00000196611	MMP1	matrix metallopeptidase 1
2,143	ENSG00000166670	MMP10	matrix metallopeptidase 10
1,061	ENSG00000099953	MMP11	matrix metallopeptidase 11
1,075	ENSG00000157227	MMP14	matrix metallopeptidase 14
1,09	ENSG00000102996	MMP15	matrix metallopeptidase 15
1,375	ENSG00000123342	MMP19	matrix metallopeptidase 19
1,019	ENSG00000125966	MMP24	matrix metallopeptidase 24
2,752	ENSG00000137673	MMP7	matrix metallopeptidase 7
1,238	ENSG00000120162	MOB3B	MOB kinase activator 3B
1,119	ENSG00000139714	MORN3	MORN repeat containing 3
1,536	ENSG00000161647	MPP3	membrane palmitoylated protein 3
1,178	ENSG00000133030	MPRIP	myosin phosphatase Rho interacting protein
1,554	ENSG00000160588	MPZL3	myelin protein zero like 3
1,251	ENSG00000116062	MSH6	mutS homolog 6
1,126	ENSG00000153944	MSI2	musashi RNA binding protein 2
1,424	ENSG00000174579	MSL2	male-specific lethal 2 homolog (Drosophila)
1,126	ENSG00000147065	MSN	moesin
1,3	ENSG00000164078	MST1R	macrophage stimulating 1 receptor

1,146	ENSG00000186472	PCLO	piccolo presynaptic cytomatrix protein
1,002	ENSG00000183036	PCP4	Purkinje cell protein 4
1,059	ENSG00000138735	PDE5A	phosphodiesterase 5A
1,205	ENSG00000160191	PDE9A	phosphodiesterase 9A
1,434	ENSG00000145431	PDGFC	platelet derived growth factor C
1,292	ENSG00000170962	PDGFD	platelet derived growth factor D
1,607	ENSG00000164951	PDP1	pyruvate dehyrogenase phosphatase catalytic subunit 1
1,008	ENSG00000196696	PDXDC2P-N	nuclear pore complex-interacting protein
1,5	ENSG00000172367	PDZD3	PDZ domain containing 3
1,121	ENSG00000165650	PDZD8	PDZ domain containing 8
1,071	ENSG00000162366	PDZK1IP1	PDZK1 interacting protein 1
1,572	ENSG00000242265	PEG10	paternally expressed 10
1,307	ENSG00000197329	PELI1	pellino E3 ubiquitin protein ligase 1
1,419	ENSG00000179094	PER1	period circadian clock 1
1,837	ENSG00000132326	PER2	period circadian clock 2
1,814	ENSG00000109272	PF4V1	platelet factor 4 variant 1
1,103	ENSG00000178921	PFAS	phosphoribosylformylglycinamidine synthase
2,111	ENSG00000170525	PFKFB3	6-phosphofructo-2-kinase/fructose-2,6-biphosphatase 3
1,442	ENSG00000067057	PFKP	phosphofructokinase, platelet
4,017	ENSG00000096088	PGC	progastricsin
2,25	ENSG00000142102	PGGHG	protein-glucosylgalactosylhydroxylysine glucosidase
1,078	ENSG00000165434	PGM2L1	phosphoglucomutase 2 like 1
1,104	ENSG00000204138	PHACTR4	phosphatase and actin regulator 4
1,62	ENSG00000111752	PHC1	polyhomeotic homolog 1
1,179	ENSG00000116273	PHF13	PHD finger protein 13
1,103	ENSG00000146247	PHIP	pleckstrin homology domain interacting protein
2,794	ENSG00000181649	PHLDA2	pleckstrin homology like domain family A member 2
1,012	ENSG00000183506	PI4KAP2	phosphatidylinositol 4-kinase alpha pseudogene 2
1,352	ENSG00000103335	PIEZO1	piezo type mechanosensitive ion channel component 1
1,671	ENSG00000162896	PIGR	polymeric immunoglobulin receptor
1,547	ENSG00000121716	PILRB	paired immunoglobin-like type 2 receptor beta
1,718	ENSG00000137193	PIM1	Pim-1 proto-oncogene, serine/threonine kinase
1,653	ENSG00000198355	PIM3	Pim-3 proto-oncogene, serine/threonine kinase
1,176	ENSG00000143398	PIP5K1A	phosphatidylinositol-4-phosphate 5-kinase type 1 alpha
1,089	ENSG00000241878	PISD	phosphatidylserine decarboxylase
1,992	ENSG00000134627	PIWIL4	piwi like RNA-mediated gene silencing 4
1,076	ENSG00000181191	PJA1	praja ring finger ubiquitin ligase 1
1,15	ENSG00000008710	PKD1	polycystin 1, transient receptor potential channel interacting
1,906	ENSG00000170927	PKHD1	PKHD1, fibrocystin/polyductin
1,535	ENSG00000184363	PKP3	plakophilin 3
1,621	ENSG00000243708	PLA2G4B	phospholipase A2 group IVB
1,281	ENSG00000184381	PLA2G6	phospholipase A2 group VI
1,007	ENSG00000126003	PLAGL2	PLAG1 like zinc finger 2
2,616	ENSG00000011422	PLAUR	plasminogen activator, urokinase receptor
1,36	ENSG00000161714	PLCD3	phospholipase C delta 3
1,233	ENSG00000129219	PLD2	phospholipase D2
1,009	ENSG00000143850	PLEKHA6	pleckstrin homology domain containing A6
1,432	ENSG00000166689	PLEKHA7	pleckstrin homology domain containing A7
2,222	ENSG00000021300	PLEKHB1	pleckstrin homology domain containing B1
1,372	ENSG00000126822	PLEKHG3	pleckstrin homology and RhoGEF domain containing G3
1,64	ENSG00000008323	PLEKHG6	pleckstrin homology and RhoGEF domain containing G6
1,145	ENSG00000214176	PLEKHM1P1	pleckstrin homology and RUN domain containing M1 pseudogene 1
2,923	ENSG00000145632	PLK2	polo like kinase 2
2,553	ENSG00000173846	PLK3	polo like kinase 3
1,086	ENSG00000102934	PLLP	plasmolipin
2,077	ENSG00000141934	PLPP2	phospholipid phosphatase 2
1,927	ENSG00000164050	PLXNB1	plexin B1
1,011	ENSG00000196576	PLXNB2	plexin B2
4,301	ENSG00000141682	PMAIP1	phorbol-12-myristate-13-acetate-induced protein 1
2,268	ENSG00000124225	PMEPA1	prostate transmembrane protein, androgen induced 1
1,332	ENSG00000132424	PNISR	PNN interacting serine and arginine rich protein
1,206	ENSG00000100941	PNN	pinin, desmosome associated protein
1,4	ENSG00000177666	PNPLA2	patatin like phospholipase domain containing 2
1,39	ENSG00000146278	PNRC1	proline rich nuclear receptor coactivator 1
1,508	ENSG00000124429	POF1B	premature ovarian failure, 1B
1,225	ENSG00000143442	POGZ	pogo transposable element derived with ZNF domain
1,655	ENSG00000181222	POLR2A	RNA polymerase II subunit A
1,099	ENSG00000009830	POMT2	protein O-mannosyltransferase 2
1,055	ENSG00000137709	POU2F3	POU class 2 homeobox 3
1,559	ENSG00000185668	POU3F1	POU class 3 homeobox 1
1,233	ENSG00000106536	POU6F2	POU class 6 homeobox 2
1,221	ENSG00000112033	PPARD	peroxisome proliferator activated receptor delta
2,242	ENSG00000118898	PPL	periplakin
1,059	ENSG00000170836	PPM1D	protein phosphatase, Mg2+/Mn2+ dependent 1D
1,593	ENSG00000088808	PPP1R13B	protein phosphatase 1 regulatory subunit 13B
2,1	ENSG00000104881	PPP1R13L	protein phosphatase 1 regulatory subunit 13 like
4,549	ENSG00000087074	PPP1R15A	protein phosphatase 1 regulatory subunit 15A
1,493	ENSG00000158615	PPP1R15B	protein phosphatase 1 regulatory subunit 15B
1,592	ENSG00000196422	PPP1R26	protein phosphatase 1 regulatory subunit 26
1,147	ENSG00000158528	PPP1R9A	protein phosphatase 1 regulatory subunit 9A
1,139	ENSG00000156475	PPP2R2B	protein phosphatase 2 regulatory subunit Bbeta
1,103	ENSG00000124224	PPP4R1L	protein phosphatase 4 regulatory subunit 1 like (pseudogene)
1,775	ENSG00000148840	PPRC1	peroxisome proliferator-activated receptor gamma, coactivator-rela
1,148	ENSG00000196850	PPTC7	PTC7 protein phosphatase homolog
1,622	ENSG00000139174	PRICKLE1	prickle planar cell polarity protein 1
1,046	ENSG00000124593	PRICKLE4	prickle planar cell polarity protein 4
1,04	ENSG00000162409	PRKAA2	protein kinase AMP-activated catalytic subunit alpha 2
1,206	ENSG00000163932	PRKCD	protein kinase C delta
1,049	ENSG00000105287	PRKD2	protein kinase D2
2,385	ENSG00000007062	PROM1	prominin 1
1,413	ENSG00000110844	PRPF40B	pre-mRNA processing factor 40 homolog B
1,669	ENSG00000131188	PRR7	proline rich 7, synaptic
1,124	ENSG00000130723	PRRC2B	proline rich coiled-coil 2B
1,236	ENSG00000117523	PRRC2C	proline rich coiled-coil 2C
3,412	ENSG00000005001	PRSS22	protease, serine 22
1,771	ENSG00000206549	PRSS50	protease, serine 50
2,032	ENSG00000052344	PRSS8	protease, serine 8
1,399	ENSG00000125650	PSPN	persephin
1,086	ENSG00000011304	PTBP1	polypyrimidine tract binding protein 1
1,029	ENSG00000117569	PTBP2	polypyrimidine tract binding protein 2
1,442	ENSG00000244694	PTCHD4	patched domain containing 4
1,272	ENSG00000122420	PTGFR	prostaglandin F receptor
1,262	ENSG00000134247	PTGFRN	prostaglandin F2 receptor inhibitor
2,333	ENSG00000087494	PTHLH	parathyroid hormone like hormone
1,41	ENSG00000163629	PTPN13	protein tyrosine phosphatase, non-receptor type 13
1,031	ENSG00000152104	PTPN14	protein tyrosine phosphatase, non-receptor type 14
1,521	ENSG00000153707	PTPRD	protein tyrosine phosphatase, receptor type D
1,025	ENSG00000142949	PTPRF	protein tyrosine phosphatase, receptor type F
1,215	ENSG00000152894	PTPRK	protein tyrosine phosphatase, receptor type K
1,163	ENSG00000173482	PTPRM	protein tyrosine phosphatase, receptor type M
1,115	ENSG00000146676	PURB	purine rich element binding protein B
1,069	ENSG00000171813	PWWP2B	PWWP domain containing 2B
1,314	ENSG00000130508	PXDN	peroxidasin
1,547	ENSG00000100994	PYGB	glycogen phosphorylase B
2,867	ENSG00000156675	RAB11FIP1	RAB11 family interacting protein 1
1,234	ENSG00000135631	RAB11FIP5	RAB11 family interacting protein 5
1,167	ENSG00000146955	RAB19	RAB19, member RAS oncogene family
1,359	ENSG00000132698	RAB25	RAB25, member RAS oncogene family
1,109	ENSG00000100228	RAB36	RAB36, member RAS oncogene family
1,107	ENSG00000123892	RAB38	RAB38, member RAS oncogene family
1,738	ENSG00000127328	RAB3IP	RAB3A interacting protein
1,631	ENSG00000108557	RAI1	retinoic acid induced 1
1,493	ENSG00000131831	RAI2	retinoic acid induced 2
1,287	ENSG00000174373	RALGAPA1	Ral GTPase activating protein catalytic alpha subunit 1
1,442	ENSG00000160271	RALGDS	ral guanine nucleotide dissociation stimulator
1,249	ENSG00000136828	RALGPS1	Ral GEF with PH domain and SH3 binding motif 1
1,861	ENSG00000184672	RALYL	RALY RNA binding protein-like
1,355	ENSG00000100401	RANGAP1	Ran GTPase activating protein 1
1,327	ENSG00000076864	RAP1GAP	RAP1 GTPase activating protein
1,261	ENSG00000132359	RAP1GAP2	RAP1 GTPase activating protein 2
1,745	ENSG00000181467	RAP2B	RAP2B, member of RAS oncogene family
1,163	ENSG00000077092	RARB	retinoic acid receptor beta
1,513	ENSG00000172819	RARG	retinoic acid receptor gamma
1,332	ENSG00000111344	RASAL1	RAS protein activator like 1
1,495	ENSG00000108551	RASD1	ras related dexamethasone induced 1

2,21	ENSG00000163132	MSX1	msh homeobox 1
1,196	ENSG00000100330	MTMR3	myotubularin related protein 3
1,308	ENSG00000256618	MTRNR2L1	MT-RNR2-like 1
1,189	ENSG00000256045	MTRNR2L10	MT-RNR2-like 10
1,293	ENSG00000271043	MTRNR2L2	MT-RNR2-like 2
1,195	ENSG00000255823	MTRNR2L8	MT-RNR2-like 8
1,849	ENSG00000185499	MUC1	mucin 1, cell surface associated
1,409	ENSG00000176945	MUC20	mucin 20, cell surface associated
1,066	ENSG00000117983	MUC5B	mucin 5B, oligomeric mucus/gel-forming
2,877	ENSG00000184956	MUC6	mucin 6, oligomeric mucus/gel-forming
1,754	ENSG00000157502	MUM1L1	MUM1 like 1
1,064	ENSG00000013364	MVP	major vault protein
2,746	ENSG00000059728	MXD1	MAX dimerization protein 1
3,075	ENSG00000179820	MYADM	myeloid associated differentiation marker
1,135	ENSG00000132382	MYBBP1A	MYB binding protein 1a
3,108	ENSG00000136997	MYC	MYC proto-oncogene, bHLH transcription factor
1,523	ENSG00000104177	MYEF2	myelin expression factor 2
1,601	ENSG00000105357	MYH14	myosin heavy chain 14
2,383	ENSG00000100345	MYH9	myosin heavy chain 9
2,1	ENSG00000007944	MYLIP	myosin regulatory light chain interacting protein
2,094	ENSG00000145555	MYO10	myosin X
1,284	ENSG00000197879	MYO1C	myosin IC
1,574	ENSG00000157483	MYO1E	myosin IE
1,091	ENSG00000095777	MYO3A	myosin IIIA
1,23	ENSG00000167306	MYO5B	myosin VB
1,142	ENSG00000128833	MYO5C	myosin VC
1,448	ENSG00000196586	MYO6	myosin VI
1,424	ENSG00000138119	MYOF	myoferlin
1,308	ENSG00000263155	MYZAP	myocardial zonula adherens protein
1,473	ENSG00000099326	MZF1	myeloid zinc finger 1
1,936	ENSG00000145911	N4BP3	NEDD4 binding protein 3
1,543	ENSG00000172766	NAA16	N(alpha)-acetyltransferase 16, NatA auxiliary subunit
1,427	ENSG00000177694	NAALADL2	N-acetylated alpha-linked acidic dipeptidase like 2
1,398	ENSG00000160796	NBEAL2	neurobeachin like 2
3,463	ENSG00000144959	NCEH1	neutral cholesterol ester hydrolase 1
1,866	ENSG00000071051	NCK2	NCK adaptor protein 2
1,426	ENSG00000115053	NCL	nucleolin
2,831	ENSG00000111912	NCOA7	nuclear receptor coactivator 7
1,037	ENSG00000166579	NDEL1	nudE neurodevelopment protein 1 like 1
1,659	ENSG00000245532	NEAT1	nuclear paraspeckle assembly transcript 1 (non-protein coding)
1,056	ENSG00000100400	NECTIN1	nectin cell adhesion molecule 1
1,331	ENSG00000130202	NECTIN2	nectin cell adhesion molecule 2
2,062	ENSG00000143217	NECTIN4	nectin cell adhesion molecule 4
2,944	ENSG00000049759	NEDD4L	neural precursor cell expressed, developmentally down-regulated 4-like, E3 ubiquitin protein ligase
3,174	ENSG00000111859	NEDD9	neural precursor cell expressed, developmentally down-regulated 9
1,175	ENSG00000163491	NEK10	NIMA related kinase 10
1,347	ENSG00000173848	NET1	neuroepithelial cell transforming 1
2,53	ENSG00000163121	NEURL3	neuralized E3 ubiquitin protein ligase 3
1,798	ENSG00000102908	NFAT5	nuclear factor of activated T-cells 5
2,836	ENSG00000131196	NFATC1	nuclear factor of activated T-cells 1
2,02	ENSG00000101096	NFATC2	nuclear factor of activated T-cells 2
1,636	ENSG00000050344	NFE2L3	nuclear factor, erythroid 2 like 3
1,47	ENSG00000165030	NFIL3	nuclear factor, interleukin 3 regulated
2,436	ENSG00000109320	NFKB1	nuclear factor kappa B subunit 1
2,103	ENSG00000077150	NFKB2	nuclear factor kappa B subunit 2
1,9	ENSG00000167604	NFKBID	NFKB inhibitor delta
1,836	ENSG00000146232	NFKBIE	NFKB inhibitor epsilon
3,786	ENSG00000144802	NFKBIZ	NFKB inhibitor zeta
1,045	ENSG00000170322	NFRKB	nuclear factor related to kappaB binding protein
1,59	ENSG00000001167	NFYA	nuclear transcription factor Y subunit alpha
1,165	ENSG00000164190	NIPBL	NIPBL, cohesin loading factor
1,595	ENSG00000167034	NKX3-1	NK3 homeobox 1
1,161	ENSG00000165246	NLGN4Y	neuroligin 4, Y-linked
1,108	ENSG00000197696	NMB	neuromedin B
1,826	ENSG00000112981	NME5	NME/NM23 family member 5
1,827	ENSG00000151014	NOCT	nocturnin
1,317	ENSG00000166197	NOLC1	nucleolar and coiled-body phosphoprotein 1
1,185	ENSG00000188747	NOXA1	NADPH oxidase activator 1
1,122	ENSG00000170485	NPAS2	neuronal PAS domain protein 2
3,174	ENSG00000141458	NPC1	NPC intracellular cholesterol transporter 1
1,104	ENSG00000215440	NPEPL1	aminopeptidase-like 1
1,367	ENSG00000141279	NPEPPS	aminopeptidase puromycin sensitive
1,136	ENSG00000131697	NPHP4	nephrocystin 4
1,317	ENSG00000183426	NPIPA7 (incluc	nuclear pore complex interacting protein family member A7
1,6	ENSG00000169246	NPIPB5 (incluc	nuclear pore complex interacting protein family member B4
1,799	ENSG00000168743	NPNT	nephronectin
1,215	ENSG00000163273	NPPC	natriuretic peptide C
2,143	ENSG00000106236	NPTX2	neuronal pentraxin 2
1,56	ENSG00000131910	NR0B2	nuclear receptor subfamily 0 group B member 2
1,35	ENSG00000126368	NR1D1	nuclear receptor subfamily 1 group D member 1
2,114	ENSG00000123358	NR4A1	nuclear receptor subfamily 4 group A member 1
1,266	ENSG00000153234	NR4A2	nuclear receptor subfamily 4 group A member 2
2,073	ENSG00000198435	NRARP	NOTCH-regulated ankyrin repeat protein
1,029	ENSG00000106459	NRF1	nuclear respiratory factor 1
1,79	ENSG00000157168	NRG1	neuregulin 1
2,056	ENSG00000180530	NRIP1	nuclear receptor interacting protein 1
1,123	ENSG00000179299	NSUN7	NOP2/Sun RNA methyltransferase family member 7
1,263	ENSG00000111696	NT5DC3	5'-nucleotidase domain containing 3
1,065	ENSG00000074527	NTN4	netrin 4
4,222	ENSG00000163545	NUAK2	NUAK family kinase 2
1,352	ENSG00000108256	NUFIP2	NUFIP2, FMR1 interacting protein 2
1,486	ENSG00000137497	NUMA1	nuclear mitotic apparatus protein 1
1,222	ENSG00000124789	NUP153	nucleoporin 153
1,234	ENSG00000095319	NUP188	nucleoporin 188
1,623	ENSG00000110713	NUP98	nucleoporin 98
1,671	ENSG00000137804	NUSAP1	nucleolar and spindle associated protein 1
1,469	ENSG00000162231	NXF1	nuclear RNA export factor 1
1,535	ENSG00000124006	OBSL1	obscurin like 1
1,377	ENSG00000115758	ODC1	ornithine decarboxylase 1
1,552	ENSG00000046651	OFD1	OFD1, centriole and centriolar satellite protein
2,376	ENSG00000173391	OLR1	oxidized low density lipoprotein receptor 1
1,752	ENSG00000169856	ONECUT1	one cut homeobox 1
1,433	ENSG00000079482	OPHN1	oligophrenin 1
1,037	ENSG00000070882	OSBPL3	oxysterol binding protein like 3
1,272	ENSG00000164823	OSGIN2	oxidative stress induced growth inhibitor family member 2
1,719	ENSG00000165312	OTUD1	OTU deubiquitinase 1
1,2	ENSG00000169914	OTUD3	OTU deubiquitinase 3
2,605	ENSG00000164164	OTUD4	OTU deubiquitinase 4
2,919	ENSG00000172818	OVOL1	ovo like transcriptional repressor 1
1,169	ENSG00000125850	OVOL2	ovo like zinc finger 2
3,098	ENSG00000180914	OXTR	oxytocin receptor
1,428	ENSG00000169860	P2RY1	purinergic receptor P2Y1
1,495	ENSG00000101104	PABPC1L	poly(A) binding protein cytoplasmic 1 like
1,127	ENSG00000077264	PAK3	p21 (RAC1) activated kinase 3
1,467	ENSG00000187867	PALM3	paralemmin 3
1,092	ENSG00000152520	PAN3	PAN3 poly(A) specific ribonuclease subunit
1,489	ENSG00000110218	PANX1	pannexin 1
1,585	ENSG00000100767	PAPLN	papilin, proteoglycan like sulfated glycoprotein
1,28	ENSG00000138801	PAPSS1	3'-phosphoadenosine 5'-phosphosulfate synthase 1
1,771	ENSG00000137819	PAQR5	progestin and adipoQ receptor family member 5
1,254	ENSG00000148498	PARD3	par-3 family cell polarity regulator
1,064	ENSG00000116117	PARD3B	par-3 family cell polarity regulator beta
2,727	ENSG00000124171	PARD6B	par-6 family cell polarity regulator beta
1,622	ENSG00000132849	PATJ	PATJ, crumbs cell polarity complex component
1,053	ENSG00000100105	PATZ1	POZ/BTB and AT hook containing zinc finger 1
1,483	ENSG00000177425	PAWR	pro-apoptotic WT1 regulator
1,06	ENSG00000105717	PBX4	PBX homeobox 4
1,28	ENSG00000228288	PCAT6	prostate cancer associated transcript 6 (non-protein coding)
1,785	ENSG00000165494	PCF11	PCF11 cleavage and polyadenylation factor subunit
1,014	ENSG00000186472	PCLO	piccolo presynaptic cytomatrix protein
1,086	ENSG00000138735	PDE5A	phosphodiesterase 5A
1,193	ENSG00000145431	PDGFC	platelet derived growth factor C
1,179	ENSG00000107438	PDLIM1	PDZ and LIM domain 1
2,07	ENSG00000164951	PDP1	pyruvate dehyrogenase phosphatase catalytic subunit 1
1,452	ENSG00000172367	PDZD3	PDZ domain containing 3

2,469	ENSG00000165105	RASEF	RAS and EF-hand domain containing
1,578	ENSG00000107551	RASSF4	Ras association domain family member 4
1,16	ENSG00000169435	RASSF6	Ras association domain family member 6
1,492	ENSG00000123094	RASSF8	Ras association domain family member 8
3,392	ENSG00000198774	RASSF9	Ras association domain family member 9
1,12	ENSG00000161847	RAVER1	ribonucleoprotein, PTB binding 1
1,195	ENSG00000162437	RAVER2	ribonucleoprotein, PTB binding 2
1,791	ENSG00000122257	RBBP6	RB binding protein 6, ubiquitin ligase
1,046	ENSG00000101773	RBBP8	RB binding protein 8, endonuclease
1,097	ENSG00000239306	RBM14	RNA binding motif protein 14
1,554	ENSG00000162775	RBM15	RNA binding motif protein 15
1,091	ENSG00000122965	RBM19	RNA binding motif protein 19
1,053	ENSG00000119707	RBM25	RNA binding motif protein 25
1,549	ENSG00000132819	RBM38	RNA binding motif protein 38
1,283	ENSG00000131051	RBM39	RNA binding motif protein 39
1,27	ENSG00000004534	RBM6	RNA binding motif protein 6
1,112	ENSG00000076067	RBMS2	RNA binding motif single stranded interacting protein 2
1,043	ENSG00000157110	RBPMS	RNA binding protein with multiple splicing
1,198	ENSG00000135870	RC3H1	ring finger and CCCH-type domains 1
1,069	ENSG00000179051	RCC2	regulator of chromosome condensation 2
2,105	ENSG00000100918	REC8	REC8 meiotic recombination protein
1,936	ENSG00000162924	REL	REL proto-oncogene, NF-kB subunit
1,721	ENSG00000104856	RELB	RELB proto-oncogene, NF-kB subunit
1,525	ENSG00000139890	REM2	RRAD and GEM like GTPase 2
1,089	ENSG00000142599	RERE	arginine-glutamic acid dipeptide repeats
1,173	ENSG00000223638	RFPL4A/RFF	ret finger protein like 4A
1,678	ENSG00000087903	RFX2	regulatory factor X2
1,783	ENSG00000205517	RGL3	ral guanine nucleotide dissociation stimulator like 3
1,599	ENSG00000169629	RGPD4 (incl	RANBP2-like and GRIP domain containing 5
1,148	ENSG00000091844	RGS17	regulator of G protein signaling 17
2,87	ENSG00000117152	RGS4	regulator of G protein signaling 4
1,711	ENSG00000007384	RHBDF1	rhomboid 5 homolog 1
1,634	ENSG00000129667	RHBDF2	rhomboid 5 homolog 2
1,676	ENSG00000158315	RHBDL2	rhomboid like 2
1,695	ENSG00000143878	RHOB	ras homolog family member B
1,009	ENSG00000140983	RHOT2	ras homolog family member T2
2,817	ENSG00000104140	RHOV	ras homolog family member V
1,746	ENSG00000131941	RHPN2	rhophilin Rho GTPase binding protein 2
1,155	ENSG00000166405	RIC3	RIC3 acetylcholine receptor chaperone
1,711	ENSG00000111785	RIC8B	RIC8 guanine nucleotide exchange factor B
1,963	ENSG00000104312	RIPK2	receptor interacting serine/threonine kinase 2
2,824	ENSG00000183421	RIPK4	receptor interacting serine/threonine kinase 4
1,548	ENSG00000117000	RLF	rearranged L-myc fusion
1,131	ENSG00000172602	RND1	Rho family GTPase 1
1,884	ENSG00000034677	RNF19A	ring finger protein 19A, RBR E3 ubiquitin protein ligase
1,719	ENSG00000116514	RNF19B	ring finger protein 19B
1,538	ENSG00000158286	RNF207	ring finger protein 207
1,113	ENSG00000173821	RNF213	ring finger protein 213
1,03	ENSG00000237330	RNF223	ring finger protein 223
1,322	ENSG00000108375	RNF43	ring finger protein 43
1,16	ENSG00000185946	RNPC3	RNA binding region (RNP1, RRM) containing 3
3,754	ENSG00000166592	RRAD	RRAD, Ras related glycolysis inhibitor and calcium channel regulator
1,47	ENSG00000052749	RRP12	ribosomal RNA processing 12 homolog
1,042	ENSG00000182841	RRP7BP	ribosomal RNA processing 7 homolog B, pseudogene
1,356	ENSG00000160188	RSPH1	radial spoke head 1 homolog
1,054	ENSG00000130363	RSPH3	radial spoke 3 homolog
1,259	ENSG00000111011	RSRC2	arginine and serine rich coiled-coil 2
1,906	ENSG00000117616	RSRP1	arginine and serine rich protein 1
1,151	ENSG00000185924	RTN4RL1	reticulon 4 receptor like 1
1,156	ENSG00000159216	RUNX1	runt related transcription factor 1
1,026	ENSG00000160753	RUSC1	RUN and SH3 domain containing 1
2,041	ENSG00000198853	RUSC2	RUN and SH3 domain containing 2
1,323	ENSG00000197747	S100A10	S100 calcium binding protein A10
1,258	ENSG00000163191	S100A11	S100 calcium binding protein A11
1,736	ENSG00000189334	S100A14	S100 calcium binding protein A14
1,255	ENSG00000188015	S100A3	S100 calcium binding protein A3
1,703	ENSG00000197956	S100A6	S100 calcium binding protein A6
1,34	ENSG00000160633	SAFB	scaffold attachment factor B
1,027	ENSG00000130254	SAFB2	scaffold attachment factor B2
1,169	ENSG00000141858	SAMD1	sterile alpha motif domain containing 1
1,182	ENSG00000020577	SAMD4A	sterile alpha motif domain containing 4A
1,118	ENSG00000205307	SAP25	Sin3A associated protein 25
1,05	ENSG00000100241	SBF1	SET binding factor 1
1,145	ENSG00000188322	SBK1	SH3 domain binding kinase 1
1,054	ENSG00000213079	SCAF8	SR-related CTD associated factor 8
1,058	ENSG00000140386	SCAPER	S-phase cyclin A associated protein in the ER
1,55	ENSG00000252010	SCARNA5	small Cajal body-specific RNA 5
4,481	ENSG00000251733	SCARNA8	small Cajal body-specific RNA 8
1,878	ENSG00000145284	SCD5	stearoyl-CoA desaturase 5
1,616	ENSG00000079689	SCGN	secretagogin, EF-hand calcium binding protein
1,528	ENSG00000111319	SCNN1A	sodium channel epithelial 1 alpha subunit
1,922	ENSG00000136193	SCRN1	secernin 1
2,981	ENSG00000080293	SCTR	secretin receptor
1,044	ENSG00000124145	SDC4	syndecan 4
1,416	ENSG00000185485	SDHAP1	succinate dehydrogenase complex flavoprotein subunit A pseudoge
1,146	ENSG00000215837	SDHAP2	succinate dehydrogenase complex flavoprotein subunit A pseudoge
1,088	ENSG00000214491	SEC14L6	SEC14 like lipid binding 6
1,519	ENSG00000091490	SEL1L3	SEL1L family member 3
4,02	ENSG00000007908	SELE	selectin E
1,658	ENSG00000170381	SEMA3E	semaphorin 3E
1,686	ENSG00000092421	SEMA6A	semaphorin 6A
1,332	ENSG00000137872	SEMA6D	semaphorin 6D
3,54	ENSG00000197632	SERPINB2	serpin family B member 2
1,261	ENSG00000170542	SERPINB9	serpin family B member 9
2,277	ENSG00000149257	SERPINH1	serpin family H member 1
1,862	ENSG00000197019	SERTAD1	SERTA domain containing 1
1,964	ENSG00000179833	SERTAD2	SERTA domain containing 2
1,049	ENSG00000167565	SERTAD3	SERTA domain containing 3
1,534	ENSG00000130766	SESN2	sestrin 2
1,746	ENSG00000174938	SEZ6L2	seizure related 6 homolog like 2
1,177	ENSG00000104897	SF3A2	splicing factor 3a subunit 2
1,13	ENSG00000198089	SFI1	SFI1 centrin binding protein
2,669	ENSG00000175793	SFN	stratifin
1,422	ENSG00000116560	SFPQ	splicing factor proline and glutamine rich
2,381	ENSG00000120057	SFRP5	secreted frizzled related protein 5
2,729	ENSG00000164023	SGMS2	sphingomyelin synthase 2
2,379	ENSG00000163082	SGPP2	sphingosine-1-phosphate phosphatase 2
1,846	ENSG00000125731	SH2D3A	SH2 domain containing 3A
1,07	ENSG00000130147	SH3BP4	SH3 domain binding protein 4
1,13	ENSG00000148341	SH3GLB2	SH3 domain containing GRB2 like, endophilin B2
1,888	ENSG00000154447	SH3RF1	SH3 domain containing ring finger 1
1,708	ENSG00000156463	SH3RF2	SH3 domain containing ring finger 2
1,19	ENSG00000035115	SH3YL1	SH3 and SYLF domain containing 1
1,152	ENSG00000107338	SHB	SH2 domain containing adaptor protein B
1,301	ENSG00000180730	SHISA2	shisa family member 2
2,283	ENSG00000237515	SHISA9	shisa family member 9
1,958	ENSG00000138771	SHROOM3	shroom family member 3
1,295	ENSG00000096717	SIRT1	sirtuin 1
1,649	ENSG00000100625	SIX4	SIX homeobox 4
2,885	ENSG00000064651	SLC12A2	solute carrier family 12 member 2
1,249	ENSG00000124067	SLC12A4	solute carrier family 12 member 4
1,206	ENSG00000113504	SLC12A7	solute carrier family 12 member 7
1,726	ENSG00000118596	SLC16A7	solute carrier family 16 member 7
1,116	ENSG00000146039	SLC17A4	solute carrier family 17 member 4
1,393	ENSG00000117479	SLC19A2	solute carrier family 19 member 2
1,006	ENSG00000144136	SLC20A1	solute carrier family 20 member 1
1,336	ENSG00000153291	SLC25A27	solute carrier family 25 member 27
1,899	ENSG00000197119	SLC25A29	solute carrier family 25 member 29
1,111	ENSG00000130304	SLC27A1	solute carrier family 27 member 1
1,796	ENSG00000197506	SLC28A3	solute carrier family 28 member 3
1,823	ENSG00000174669	SLC29A2	solute carrier family 29 member 2
1,196	ENSG00000117394	SLC2A1	solute carrier family 2 member 1
2,006	ENSG00000157765	SLC34A2	solute carrier family 34 member 2
1,085	ENSG00000100036	SLC35E4	solute carrier family 35 member E4
2,022	ENSG00000110660	SLC35F2	solute carrier family 35 member F2

1,399	ENSG00000165650	PDZD8	PDZ domain containing 8
1,061	ENSG00000162366	PDZK1IP1	PDZK1 interacting protein 1
1,369	ENSG00000242265	PEG10	paternally expressed 10
1,928	ENSG00000197329	PELI1	pellino E3 ubiquitin protein ligase 1
1,12	ENSG00000139946	PELI2	pellino E3 ubiquitin protein ligase family member 2
1,252	ENSG00000179094	PER1	period circadian clock 1
2,264	ENSG00000132326	PER2	period circadian clock 2
1,538	ENSG00000109272	PF4V1	platelet factor 4 variant 1
3,227	ENSG00000170525	PFKFB3	6-phosphofructo-2-kinase/fructose-2,6-biphosphatase 3
1,944	ENSG00000067057	PFKP	phosphofructokinase, platelet
1,318	ENSG00000096088	PGC	progastricsin
2,374	ENSG00000142102	PGGHG	protein-glucosylgalactosylhydroxylysine glucosidase
1,024	ENSG00000204138	PHACTR4	phosphatase and actin regulator 4
1,569	ENSG00000111752	PHC1	polyhomeotic homolog 1
1,445	ENSG00000116273	PHF13	PHD finger protein 13
1,09	ENSG00000146247	PHIP	pleckstrin homology domain interacting protein
1,821	ENSG00000139289	PHLDA1	pleckstrin homology like domain family A member 1
3,492	ENSG00000181649	PHLDA2	pleckstrin homology like domain family A member 2
1,126	ENSG00000070047	PHRF1	PHD and ring finger domains 1
1,674	ENSG00000103335	PIEZO1	piezo type mechanosensitive ion channel component 1
1,037	ENSG00000174227	PIGG	phosphatidylinositol glycan anchor biosynthesis class G
1,667	ENSG00000162896	PIGR	polymeric immunoglobulin receptor
1,18	ENSG00000121879	PIK3CA	phosphatidylinositol-4,5-bisphosphate 3-kinase catalytic subunit alpha
1,31	ENSG00000121716	PILRB	paired immunoglobin-like type 2 receptor beta
1,925	ENSG00000137193	PIM1	Pim-1 proto-oncogene, serine/threonine kinase
1,839	ENSG00000198355	PIM3	Pim-3 proto-oncogene, serine/threonine kinase
1,703	ENSG00000143398	PIP5K1A	phosphatidylinositol-4-phosphate 5-kinase type 1 alpha
1,011	ENSG00000167103	PIP5KL1	phosphatidylinositol-4-phosphate 5-kinase like 1
1,285	ENSG00000241878	PISD	phosphatidylserine decarboxylase
1,679	ENSG00000134627	PIWIL4	piwi like RNA-mediated gene silencing 4
1,261	ENSG00000181191	PJA1	praja ring finger ubiquitin ligase 1
1,064	ENSG00000008710	PKD1	polycystin 1, transient receptor potential channel interacting
1,595	ENSG00000170927	PKHD1	PKHD1, fibrocystin/polyductin
1,458	ENSG00000184363	PKP3	plakophilin 3
1,412	ENSG00000243708	PLA2G4B	phospholipase A2 group IVB
1,18	ENSG00000184381	PLA2G6	phospholipase A2 group VI
1,233	ENSG00000126003	PLAGL2	PLAG1 like zinc finger 2
3,487	ENSG00000011422	PLAUR	plasminogen activator, urokinase receptor
1,965	ENSG00000161714	PLCD3	phospholipase C delta 3
1,091	ENSG00000182378	PLCXD1	phosphatidylinositol specific phospholipase C X domain containing 1
1,212	ENSG00000129219	PLD2	phospholipase D2
1,544	ENSG00000166689	PLEKHA7	pleckstrin homology domain containing A7
2	ENSG00000021300	PLEKHB1	pleckstrin homology domain containing B1
1,173	ENSG00000175895	PLEKHF2	pleckstrin homology and FYVE domain containing 2
1,846	ENSG00000126822	PLEKHG3	pleckstrin homology and RhoGEF domain containing G3
1,745	ENSG00000008323	PLEKHG6	pleckstrin homology and RhoGEF domain containing G6
1,369	ENSG00000214176	PLEKHM1P1	pleckstrin homology and RUN domain containing M1 pseudogene 1
1,241	ENSG00000187583	PLEKHN1	pleckstrin homology domain containing N1
3,356	ENSG00000145632	PLK2	polo like kinase 2
2,969	ENSG00000173846	PLK3	polo like kinase 3
1,153	ENSG00000102934	PLLP	plasmolipin
1,896	ENSG00000141934	PLPP2	phospholipid phosphatase 2
1,788	ENSG00000164050	PLXNB1	plexin B1
1,195	ENSG00000146281	PM20D2	peptidase M20 domain containing 2
4,651	ENSG00000141682	PMAIP1	phorbol-12-myristate-13-acetate-induced protein 1
2,495	ENSG00000124225	PMEPA1	prostate transmembrane protein, androgen induced 1
1,034	ENSG00000140464	PML	promyelocytic leukemia
1,193	ENSG00000132424	PNISR	PNN interacting serine and arginine rich protein
1,186	ENSG00000100941	PNN	pinin, desmosome associated protein
1,442	ENSG00000177666	PNPLA2	patatin like phospholipase domain containing 2
1,496	ENSG00000146278	PNRC1	proline rich nuclear receptor coactivator 1
1,258	ENSG00000124429	POF1B	premature ovarian failure, 1B
1,119	ENSG00000143157	POGK	pogo transposable element derived with KRAB domain
1,446	ENSG00000143442	POGZ	pogo transposable element derived with ZNF domain
1,136	ENSG00000100227	POLDIP3	DNA polymerase delta interacting protein 3
2,083	ENSG00000181222	POLR2A	RNA polymerase II subunit A
1,383	ENSG00000168495	POLR3D	RNA polymerase III subunit D
1,353	ENSG00000137709	POU2F3	POU class 2 homeobox 3
1,681	ENSG00000185668	POU3F1	POU class 3 homeobox 1
1,172	ENSG00000106536	POU6F2	POU class 6 homeobox 2
2,181	ENSG00000112033	PPARD	peroxisome proliferator activated receptor delta
2,507	ENSG00000118898	PPL	periplakin
1,212	ENSG00000170836	PPM1D	protein phosphatase, Mg2+/Mn2+ dependent 1D
1,819	ENSG00000088808	PPP1R13B	protein phosphatase 1 regulatory subunit 13B
2,097	ENSG00000104881	PPP1R13L	protein phosphatase 1 regulatory subunit 13 like
4,954	ENSG00000087074	PPP1R15A	protein phosphatase 1 regulatory subunit 15A
1,678	ENSG00000158615	PPP1R15B	protein phosphatase 1 regulatory subunit 15B
1,753	ENSG00000196422	PPP1R26	protein phosphatase 1 regulatory subunit 26
1,004	ENSG00000158528	PPP1R9A	protein phosphatase 1 regulatory subunit 9A
1,041	ENSG00000113575	PPP2CA	protein phosphatase 2 catalytic subunit alpha
1,319	ENSG00000154845	PPP4R1	protein phosphatase 4 regulatory subunit 1
2,848	ENSG00000148840	PPRC1	peroxisome proliferator-activated receptor gamma, coactivator-related 1
1,213	ENSG00000196850	PPTC7	PTC7 protein phosphatase homolog
1,175	ENSG00000019485	PRDM11	PR/SET domain 11
1,225	ENSG00000116731	PRDM2	PR/SET domain 2
1,04	ENSG00000110851	PRDM4	PR/SET domain 4
1,357	ENSG00000139174	PRICKLE1	prickle planar cell polarity protein 1
1,669	ENSG00000163932	PRKCD	protein kinase C delta
1,021	ENSG00000163558	PRKCI	protein kinase C iota
2,255	ENSG00000007062	PROM1	prominin 1
1,567	ENSG00000131188	PRR7	proline rich 7, synaptic
1,035	ENSG00000130723	PRRC2B	proline rich coiled-coil 2B
1,396	ENSG00000117523	PRRC2C	proline rich coiled-coil 2C
3,634	ENSG00000005001	PRSS22	protease, serine 22
1,24	ENSG00000206549	PRSS50	protease, serine 50
2,397	ENSG00000052344	PRSS8	protease, serine 8
1,256	ENSG00000011304	PTBP1	polypyrimidine tract binding protein 1
1,441	ENSG00000117569	PTBP2	polypyrimidine tract binding protein 2
1,01	ENSG00000244694	PTCHD4	patched domain containing 4
1,194	ENSG00000134247	PTGFRN	prostaglandin F2 receptor inhibitor
1,323	ENSG00000073756	PTGS2	prostaglandin-endoperoxide synthase 2
3,257	ENSG00000087494	PTHLH	parathyroid hormone like hormone
1,198	ENSG00000196396	PTPN1	protein tyrosine phosphatase, non-receptor type 1
1,376	ENSG00000163629	PTPN13	protein tyrosine phosphatase, non-receptor type 13
1,249	ENSG00000152104	PTPN14	protein tyrosine phosphatase, non-receptor type 14
1,406	ENSG00000153707	PTPRD	protein tyrosine phosphatase, receptor type D
1,078	ENSG00000080031	PTPRH	protein tyrosine phosphatase, receptor type H
1,311	ENSG00000152894	PTPRK	protein tyrosine phosphatase, receptor type K
1,167	ENSG00000173482	PTPRM	protein tyrosine phosphatase, receptor type M
1,553	ENSG00000134644	PUM1	pumilio RNA binding family member 1
1,162	ENSG00000080608	PUM3	pumilio RNA binding family member 3
1,11	ENSG00000146676	PURB	purine rich element binding protein B
1,169	ENSG00000073008	PVR	poliovirus receptor
1,36	ENSG00000130508	PXDN	peroxidasin
1,281	ENSG00000100994	PYGB	glycogen phosphorylase B
3,503	ENSG00000156675	RAB11FIP1	RAB11 family interacting protein 1
1,435	ENSG00000135631	RAB11FIP5	RAB11 family interacting protein 5
1,112	ENSG00000080371	RAB21	RAB21, member RAS oncogene family
1,169	ENSG00000132698	RAB25	RAB25, member RAS oncogene family
1,145	ENSG00000100228	RAB36	RAB36, member RAS oncogene family
1,088	ENSG00000123892	RAB38	RAB38, member RAS oncogene family
1,776	ENSG00000127328	RAB3IP	RAB3A interacting protein
1,001	ENSG00000166128	RAB8B	RAB8B, member RAS oncogene family
1,024	ENSG00000123595	RAB9A	RAB9A, member RAS oncogene family
1,091	ENSG00000154710	RABGEF1	RAB guanine nucleotide exchange factor 1
1,77	ENSG00000155918	RAET1L	retinoic acid early transcript 1L
1,48	ENSG00000108557	RAI1	retinoic acid induced 1
1,883	ENSG00000131831	RAI2	retinoic acid induced 2
1,175	ENSG00000174373	RALGAPA1	Ral GTPase activating protein catalytic alpha subunit 1
2,057	ENSG00000160271	RALGDS	ral guanine nucleotide dissociation stimulator
1,124	ENSG00000136828	RALGPS1	Ral GEF with PH domain and SH3 binding motif 1
1,734	ENSG00000184672	RALYL	RALY RNA binding protein-like
1,695	ENSG00000100401	RANGAP1	Ran GTPase activating protein 1
1,017	ENSG00000076864	RAP1GAP	RAP1 GTPase activating protein
1,304	ENSG00000132359	RAP1GAP2	RAP1 GTPase activating protein 2

1,197	ENSG00000183780	SLC35F3	solute carrier family 35 member F3
1,301	ENSG00000169507	SLC38A11	solute carrier family 38 member 11
1,667	ENSG00000168003	SLC3A2	solute carrier family 3 member 2
1,313	ENSG00000133065	SLC41A1	solute carrier family 41 member 1
1,438	ENSG00000143036	SLC44A3	solute carrier family 44 member 3
1,339	ENSG00000114923	SLC4A3	solute carrier family 4 member 3
2,101	ENSG00000080493	SLC4A4	solute carrier family 4 member 4
1,057	ENSG00000033867	SLC4A7	solute carrier family 4 member 7
1,66	ENSG00000100170	SLC5A1	solute carrier family 5 member 1
1,856	ENSG00000117834	SLC5A9	solute carrier family 5 member 9
2,472	ENSG00000174358	SLC6A19	solute carrier family 6 member 19
1,141	ENSG00000131389	SLC6A6	solute carrier family 6 member 6
2,202	ENSG00000139514	SLC7A1	solute carrier family 7 member 1
1,995	ENSG00000103257	SLC7A5	solute carrier family 7 member 5
2,005	ENSG00000260727	SLC7A5P1	solute carrier family 7 member 5 pseudogene 1
2,578	ENSG00000258186	SLC7A5P2	solute carrier family 7 member 5 pseudogene 2
1,426	ENSG00000090020	SLC9A1	solute carrier family 9 member A1
1,441	ENSG00000066230	SLC9A3	solute carrier family 9 member A3
1,055	ENSG00000180251	SLC9A4	solute carrier family 9 member A4
1,303	ENSG00000176463	SLCO3A1	solute carrier organic anion transporter family member 3A1
1,719	ENSG00000154760	SLFN13	schlafen family member 13
1,72	ENSG00000166949	SMAD3	SMAD family member 3
1,888	ENSG00000127616	SMARCA4	SWI/SNF related, matrix associated, actin dependent regulator of ch
3,569	ENSG00000172062	SMN1/SMN	survival of motor neuron 1, telomeric
2,194	ENSG00000088826	SMOX	spermine oxidase
1,598	ENSG00000103056	SMPD3	sphingomyelin phosphodiesterase 3
1,679	ENSG00000130768	SMPDL3B	sphingomyelin phosphodiesterase acid like 3B
1,634	ENSG00000198742	SMURF1	SMAD specific E3 ubiquitin protein ligase 1
1,412	ENSG00000132639	SNAP25	synaptosome associated protein 25
1,062	ENSG00000197989	SNHG12	small nucleolar RNA host gene 12
1,123	ENSG00000234912	SNHG20	small nucleolar RNA host gene 20
1,569	ENSG00000163877	SNIP1	Smad nuclear interacting protein 1
5,44	ENSG00000206811	SNORA10	small nucleolar RNA, H/ACA box 10
7,427	ENSG00000206910	SNORA29	small nucleolar RNA, H/ACA box 29
3,876	ENSG00000206612	SNORA2A	small nucleolar RNA, H/ACA box 2A
4,564	ENSG00000207313	SNORA2B	small nucleolar RNA, H/ACA box 2B
5,76	ENSG00000199785	SNORA52	small nucleolar RNA, H/ACA box 52
1,216	ENSG00000212443	SNORA53	small nucleolar RNA, H/ACA box 53
3,867	ENSG00000206693	SNORA56	small nucleolar RNA, H/ACA box 56
2,131	ENSG00000199266	SNORA60	small nucleolar RNA, H/ACA box 60
2,57	ENSG00000207088	SNORA7B	small nucleolar RNA, H/ACA box 7B
4,278	ENSG00000206633	SNORA80B	small nucleolar RNA, H/ACA box 80B
3,059	ENSG00000239183	SNORA84	small nucleolar RNA, H/ACA box 84
3,521	ENSG00000238917	SNORD10	small nucleolar RNA, C/D box 10
8,337	ENSG00000199753	SNORD104	small nucleolar RNA, C/D box 104
2,796	ENSG00000207014	SNORD116-	small nucleolar RNA, C/D box 116-3
5,891	ENSG00000239043	SNORD127	small nucleolar RNA, C/D box 127
2,638	ENSG00000207445	SNORD15B	small nucleolar RNA, C/D box 15B
5,671	ENSG00000200530	SNORD35B	small nucleolar RNA, C/D box 35B
5,249	ENSG00000202093	SNORD58C	small nucleolar RNA, C/D box 58C
6,486	ENSG00000209482	SNORD83A	small nucleolar RNA, C/D box 83A
1,719	ENSG00000212283	SNORD89	small nucleolar RNA, C/D box 89
2,627	ENSG00000221539	SNORD99	small nucleolar RNA, C/D box 99
1,253	ENSG00000144028	SNRNP200	small nuclear ribonucleoprotein U5 subunit 200
1,233	ENSG00000104852	SNRNP70	small nuclear ribonucleoprotein U1 subunit 70
1,254	ENSG00000159140	SON	SON DNA binding protein
1,12	ENSG00000198142	SOWAHC	sosondowah ankyrin repeat domain family member C
3,369	ENSG00000124766	SOX4	SRY-box 4
1,021	ENSG00000110693	SOX6	SRY-box 6
3,199	ENSG00000125398	SOX9	SRY-box 9
2,095	ENSG00000104450	SPAG1	sperm associated antigen 1
1,15	ENSG00000182957	SPATA13	spermatogenesis associated 13
1,136	ENSG00000152582	SPEF2	sperm flagellar 2
1,642	ENSG00000065526	SPEN	spen family transcriptional repressor
2,373	ENSG00000176170	SPHK1	sphingosine kinase 1
2,324	ENSG00000166145	SPINT1	serine peptidase inhibitor, Kunitz type 1
1,453	ENSG00000167642	SPINT2	serine peptidase inhibitor, Kunitz type 2
1,646	ENSG00000183018	SPNS2	sphingolipid transporter 2
1,615	ENSG00000118785	SPP1	secreted phosphoprotein 1
1,409	ENSG00000005206	SPPL2B	signal peptide peptidase like 2B
1,431	ENSG00000171621	SPSB1	splA/ryanodine receptor domain and SOCS box containing 1
1,199	ENSG00000197694	SPTAN1	spectrin alpha, non-erythrocytic 1
1,215	ENSG00000115306	SPTBN1	spectrin beta, non-erythrocytic 1
2,049	ENSG00000104549	SQLE	squalene epoxidase
1,048	ENSG00000197122	SRC	SRC proto-oncogene, non-receptor tyrosine kinase
1,214	ENSG00000198911	SREBF2	sterol regulatory element binding transcription factor 2
1,855	ENSG00000112658	SRF	serum response factor
1,365	ENSG00000196935	SRGAP1	SLIT-ROBO Rho GTPase activating protein 1
1,099	ENSG00000133226	SRRM1	serine and arginine repetitive matrix 1
1,709	ENSG00000167978	SRRM2	serine/arginine repetitive matrix 2
1,043	ENSG00000087087	SRRT	serrate, RNA effector molecule
1,27	ENSG00000154548	SRSF12	serine and arginine rich splicing factor 12
1,08	ENSG00000084112	SSH1	slingshot protein phosphatase 1
1,953	ENSG00000149418	ST14	suppression of tumorigenicity 14
1,123	ENSG00000166444	ST5	suppression of tumorigenicity 5
1,635	ENSG00000168439	STIP1	stress induced phosphoprotein 1
1,496	ENSG00000164543	STK17A	serine/threonine kinase 17a
2,118	ENSG00000130413	STK33	serine/threonine kinase 33
1,092	ENSG00000163482	STK36	serine/threonine kinase 36
1,097	ENSG00000198648	STK39	serine/threonine kinase 39
1,378	ENSG00000173320	STOX2	storkhead box 2
1,465	ENSG00000178750	STX19	syntaxin 19
1,747	ENSG00000166900	STX3	syntaxin 3
1,734	ENSG00000168952	STXBP6	syntaxin binding protein 6
1,5	ENSG00000064607	SUGP2	SURP and G-patch domain containing 2
1,894	ENSG00000198075	SULT1C4	sulfotransferase family 1C member 4
1,016	ENSG00000196235	SUPT5H	SPT5 homolog, DSIF elongation factor subunit
1,017	ENSG00000100647	SUSD6	sushi domain containing 6
1,23	ENSG00000197321	SVIL	supervillin
1,023	ENSG00000133789	SWAP70	SWAP switching B-cell complex subunit 70
1,762	ENSG00000205078	SYCE1L	synaptonemal complex central element protein 1 like
1,11	ENSG00000097096	SYDE2	synapse defective Rho GTPase homolog 2
1,02	ENSG00000054654	SYNE2	spectrin repeat containing nuclear envelope protein 2
1,251	ENSG00000100321	SYNGR1	synaptogyrin 1
1,42	ENSG00000078269	SYNJ2	synaptojanin 2
2,156	ENSG00000254806	SYS1-DBND	SYS1-DBNDD2 readthrough (NMD candidate)
1,387	ENSG00000019505	SYT13	synaptotagmin 13
1,593	ENSG00000149043	SYT8	synaptotagmin 8
3,062	ENSG00000184292	TACSTD2	tumor associated calcium signal transducer 2
1,037	ENSG00000103168	TAF1C	TATA-box binding protein associated factor, RNA polymerase I subu
1,63	ENSG00000166012	TAF1D	TATA-box binding protein associated factor, RNA polymerase I subu
1,057	ENSG00000106290	TAF6	TATA-box binding protein associated factor 6
1,025	ENSG00000213977	TAX1BP3	Tax1 binding protein 3
1,598	ENSG00000065491	TBC1D22B	TBC1 domain family member 22B
1,672	ENSG00000204634	TBC1D8	TBC1 domain family member 8
1,032	ENSG00000165929	TC2N	tandem C2 domains, nuclear
1,072	ENSG00000113649	TCERG1	transcription elongation regulator 1
1,025	ENSG00000071564	TCF3	transcription factor 3
1,285	ENSG00000148737	TCF7L2	transcription factor 7 like 2
1,406	ENSG00000134827	TCN1	transcobalamin 1
1,097	ENSG00000187079	TEAD1	TEA domain transcription factor 1
1,669	ENSG00000092850	TEKT2	tektin 2
1,226	ENSG00000261408	TEN1-CDK3	TEN1-CDK3 readthrough (NMD candidate)
2,407	ENSG00000135269	TES	testin LIM domain protein
1,639	ENSG00000088992	TESC	tescalcin
1,439	ENSG00000175664	TEX26	testis expressed 26
1,817	ENSG00000087510	TFAP2C	transcription factor AP-2 gamma
1,089	ENSG00000112561	TFEB	transcription factor EB
1,965	ENSG00000160182	TFF1	trefoil factor 1
1,713	ENSG00000160181	TFF2	trefoil factor 2
2,834	ENSG00000105825	TFPI2	tissue factor pathway inhibitor 2
1,373	ENSG00000163235	TGFA	transforming growth factor alpha
3,105	ENSG00000092969	TGFB2	transforming growth factor beta 2
1,524	ENSG00000177426	TGIF1	TGFB induced factor homeobox 1
1,493	ENSG00000118707	TGIF2	TGFB induced factor homeobox 2
1,826	ENSG00000181467	RAP2B	RAP2B, member of RAS oncogene family
1,108	ENSG00000173166	RAPH1	Ras association (RalGDS/AF-6) and pleckstrin homology domains 1
1,701	ENSG00000077092	RARB	retinoic acid receptor beta
1,835	ENSG00000172819	RARG	retinoic acid receptor gamma
1,76	ENSG00000155903	RASA2	RAS p21 protein activator 2
1,121	ENSG00000111344	RASAL1	RAS protein activator like 1
1,133	ENSG00000075391	RASAL2	RAS protein activator like 2
1,081	ENSG00000108551	RASD1	ras related dexamethasone induced 1
3,365	ENSG00000165105	RASEF	RAS and EF-hand domain containing
1,442	ENSG00000152689	RASGRP3	RAS guanyl releasing protein 3
1,518	ENSG00000107551	RASSF4	Ras association domain family member 4
1,25	ENSG00000169435	RASSF6	Ras association domain family member 6
1,819	ENSG00000123094	RASSF8	Ras association domain family member 8
3,439	ENSG00000198774	RASSF9	Ras association domain family member 9
1,044	ENSG00000162437	RAVER2	ribonucleoprotein, PTB binding 2
2,076	ENSG00000122257	RBBP6	RB binding protein 6, ubiquitin ligase
1,449	ENSG00000244462	RBM12	RNA binding motif protein 12
1,315	ENSG00000239306	RBM14	RNA binding motif protein 14
1,469	ENSG00000162775	RBM15	RNA binding motif protein 15
1,137	ENSG00000122965	RBM19	RNA binding motif protein 19
1,13	ENSG00000119707	RBM25	RNA binding motif protein 25
1,608	ENSG00000132819	RBM38	RNA binding motif protein 38
1,468	ENSG00000131051	RBM39	RNA binding motif protein 39
1,128	ENSG00000163694	RBM47	RNA binding motif protein 47
1,305	ENSG00000004534	RBM6	RNA binding motif protein 6
1,143	ENSG00000076067	RBMS2	RNA binding motif single stranded interacting protein 2
1,34	ENSG00000157110	RBPMS	RNA binding protein with multiple splicing
1,277	ENSG00000135870	RC3H1	ring finger and CCCH-type domains 1
1,031	ENSG00000179051	RCC2	regulator of chromosome condensation 2
1,002	ENSG00000089902	RCOR1	REST corepressor 1
1,881	ENSG00000100918	REC8	REC8 meiotic recombination protein
2,334	ENSG00000162924	REL	REL proto-oncogene, NF-kB subunit
1,077	ENSG00000173039	RELA	RELA proto-oncogene, NF-kB subunit
2,099	ENSG00000104856	RELB	RELB proto-oncogene, NF-kB subunit
1,707	ENSG00000054967	RELT	RELT, TNF receptor
1,707	ENSG00000139890	REM2	RRAD and GEM like GTPase 2
1,049	ENSG00000092871	RFFL	ring finger and FYVE like domain containing E3 ubiquitin protein ligase
1,312	ENSG00000223638	RFPL4A/RFPL4	ret finger protein like 4A
2,132	ENSG00000087903	RFX2	regulatory factor X2
1,285	ENSG00000080298	RFX3	regulatory factor X3
1,71	ENSG00000205517	RGL3	ral guanine nucleotide dissociation stimulator like 3
1,436	ENSG00000015568	RGPD4 (includ	RANBP2-like and GRIP domain containing 5
1,553	ENSG00000143333	RGS16	regulator of G protein signaling 16
2,407	ENSG00000091844	RGS17	regulator of G protein signaling 17
2,373	ENSG00000117152	RGS4	regulator of G protein signaling 4
2,07	ENSG00000007384	RHBDF1	rhomboid 5 homolog 1
1,519	ENSG00000129667	RHBDF2	rhomboid 5 homolog 2
1,096	ENSG00000158315	RHBDL2	rhomboid like 2
1,263	ENSG00000143878	RHOB	ras homolog family member B
1,336	ENSG00000139725	RHOF	ras homolog family member F, filopodia associated
2,761	ENSG00000104140	RHOV	ras homolog family member V
1,027	ENSG00000158106	RHPN1	rhophilin Rho GTPase binding protein 1
2,279	ENSG00000131941	RHPN2	rhophilin Rho GTPase binding protein 2
1,047	ENSG00000166405	RIC3	RIC3 acetylcholine receptor chaperone
1,451	ENSG00000111785	RIC8B	RIC8 guanine nucleotide exchange factor B
1,017	ENSG00000166532	RIMKLB	ribosomal modification protein rimK like family member B
2,284	ENSG00000104312	RIPK2	receptor interacting serine/threonine kinase 2
3,346	ENSG00000183421	RIPK4	receptor interacting serine/threonine kinase 4
2,056	ENSG00000117000	RLF	rearranged L-myc fusion
1,026	ENSG00000131263	RLIM	ring finger protein, LIM domain interacting
2,078	ENSG00000165799	RNASE7	ribonuclease A family member 7
1,428	ENSG00000172602	RND1	Rho family GTPase 1
2,325	ENSG00000034677	RNF19A	ring finger protein 19A, RBR E3 ubiquitin protein ligase
2,184	ENSG00000116514	RNF19B	ring finger protein 19B
1,463	ENSG00000158286	RNF207	ring finger protein 207
1,938	ENSG00000237330	RNF223	ring finger protein 223
1,278	ENSG00000101236	RNF24	ring finger protein 24
1,083	ENSG00000137075	RNF38	ring finger protein 38
1,014	ENSG00000063978	RNF4	ring finger protein 4
1,004	ENSG00000108375	RNF43	ring finger protein 43
1,035	ENSG00000223508	RPL23AP53	ribosomal protein L23a pseudogene 53
1,511	ENSG00000100784	RPS6KA5	ribosomal protein S6 kinase A5
3,55	ENSG00000166592	RRAD	RRAD, Ras related glycolysis inhibitor and calcium channel regulator
2,245	ENSG00000052749	RRP12	ribosomal RNA processing 12 homolog
1,093	ENSG00000182841	RRP7BP	ribosomal RNA processing 7 homolog B, pseudogene
1,28	ENSG00000160188	RSPH1	radial spoke head 1 homolog
1,522	ENSG00000111011	RSRC2	arginine and serine rich coiled-coil 2
1,775	ENSG00000117616	RSRP1	arginine and serine rich protein 1
1,491	ENSG00000188636	RTL6	retrotransposon Gag like 6
1,273	ENSG00000185924	RTN4RL1	reticulon 4 receptor like 1
1,499	ENSG00000102445	RUBCNL	RUN and cysteine rich domain containing beclin 1 interacting protein like
1,343	ENSG00000159216	RUNX1	runt related transcription factor 1
2,018	ENSG00000198853	RUSC2	RUN and SH3 domain containing 2
2,235	ENSG00000197747	S100A10	S100 calcium binding protein A10
1,66	ENSG00000163191	S100A11	S100 calcium binding protein A11
1,809	ENSG00000189334	S100A14	S100 calcium binding protein A14
1,511	ENSG00000196754	S100A2	S100 calcium binding protein A2
1,81	ENSG00000197956	S100A6	S100 calcium binding protein A6
1,453	ENSG00000160633	SAFB	scaffold attachment factor B
1,133	ENSG00000130254	SAFB2	scaffold attachment factor B2
1,004	ENSG00000141858	SAMD1	sterile alpha motif domain containing 1
1,058	ENSG00000020577	SAMD4A	sterile alpha motif domain containing 4A
1,209	ENSG00000179134	SAMD4B	sterile alpha motif domain containing 4B
1,186	ENSG00000161526	SAP30BP	SAP30 binding protein
1,144	ENSG00000100241	SBF1	SET binding factor 1
1,493	ENSG00000156304	SCAF4	SR-related CTD associated factor 4
1,479	ENSG00000213079	SCAF8	SR-related CTD associated factor 8
1,005	ENSG00000140386	SCAPER	S-phase cyclin A associated protein in the ER
1,669	ENSG00000145284	SCD5	stearoyl-CoA desaturase 5
1,379	ENSG00000124939	SCGB2A1	secretoglobin family 2A member 1
1,393	ENSG00000079689	SCGN	secretagogin, EF-hand calcium binding protein
1,548	ENSG00000111319	SCNN1A	sodium channel epithelial 1 alpha subunit
1,929	ENSG00000136193	SCRN1	secernin 1
2,462	ENSG00000080293	SCTR	secretin receptor
1,11	ENSG00000185485	SDHAP1	succinate dehydrogenase complex flavoprotein subunit A pseudogene 1
1,012	ENSG00000215837	SDHAP2	succinate dehydrogenase complex flavoprotein subunit A pseudogene 2
1,088	ENSG00000214491	SEC14L6	SEC14 like lipid binding 6
1,296	ENSG00000091490	SEL1L3	SEL1L family member 3
5,744	ENSG00000007908	SELE	selectin E
1,132	ENSG00000174175	SELP	selectin P
1,489	ENSG00000170381	SEMA3E	semaphorin 3E
1,006	ENSG00000185033	SEMA4B	semaphorin 4B
1,678	ENSG00000092421	SEMA6A	semaphorin 6A
1,932	ENSG00000137872	SEMA6D	semaphorin 6D
4,637	ENSG00000197632	SERPINB2	serpin family B member 2
1,019	ENSG00000166401	SERPINB8	serpin family B member 8
1,73	ENSG00000170542	SERPINB9	serpin family B member 9
1,517	ENSG00000106366	SERPINE1	serpin family E member 1
2,625	ENSG00000149257	SERPINH1	serpin family H member 1
2,046	ENSG00000197019	SERTAD1	SERTA domain containing 1
2,223	ENSG00000179833	SERTAD2	SERTA domain containing 2
1,918	ENSG00000130766	SESN2	sestrin 2
1,298	ENSG00000187231	SESTD1	SEC14 and spectrin domain containing 1
1,836	ENSG00000174938	SEZ6L2	seizure related 6 homolog like 2
3,196	ENSG00000175793	SFN	stratifin
2,164	ENSG00000116560	SFPQ	splicing factor proline and glutamine rich
2,024	ENSG00000120057	SFRP5	secreted frizzled related protein 5
4,581	ENSG00000164023	SGMS2	sphingomyelin synthase 2
2,66	ENSG00000163082	SGPP2	sphingosine-1-phosphate phosphatase 2
2,499	ENSG00000125731	SH2D3A	SH2 domain containing 3A
1,717	ENSG00000130147	SH3BP4	SH3 domain binding protein 4
2,45	ENSG00000154447	SH3RF1	SH3 domain containing ring finger 1
1,719	ENSG00000156463	SH3RF2	SH3 domain containing ring finger 2
1,39	ENSG00000107338	SHB	SH2 domain containing adaptor protein B
2,172	ENSG00000180730	SHISA2	shisa family member 2

1,118	ENSG00000115970	THADA	THADA, armadillo repeat containing
1,931	ENSG00000137801	THBS1	thrombospondin 1
1,059	ENSG00000054118	THRAP3	thyroid hormone receptor associated protein 3
1,814	ENSG00000187720	THSD4	thrombospondin type 1 domain containing 4
1,627	ENSG00000146426	TIAM2	T-cell lymphoma invasion and metastasis 2
2,36	ENSG00000127666	TICAM1	toll like receptor adaptor molecule 1
1,038	ENSG00000142910	TINAGL1	tubulointerstitial nephritis antigen like 1
2,046	ENSG00000163659	TIPARP	TCDD inducible poly(ADP-ribose) polymerase
1,521	ENSG00000104067	TJP1	tight junction protein 1
1,873	ENSG00000119139	TJP2	tight junction protein 2
1,165	ENSG00000196781	TLE1	transducin like enhancer of split 1
1,336	ENSG00000065717	TLE2	transducin like enhancer of split 2
1,662	ENSG00000106829	TLE4	transducin like enhancer of split 4
1,095	ENSG00000187554	TLR5	toll like receptor 5
2,519	ENSG00000169908	TM4SF1	transmembrane 4 L six family member 1
1,498	ENSG00000103534	TMC5	transmembrane channel like 5
1,368	ENSG00000133069	TMCC2	transmembrane and coiled-coil domain family 2
1,801	ENSG00000006118	TMEM132A	transmembrane protein 132A
2,327	ENSG00000181264	TMEM136	transmembrane protein 136
2,375	ENSG00000249992	TMEM158	transmembrane protein 158 (gene/pseudogene)
1,135	ENSG00000152128	TMEM163	transmembrane protein 163
2,239	ENSG00000157111	TMEM171	transmembrane protein 171
1,458	ENSG00000164855	TMEM184A	transmembrane protein 184A
1,245	ENSG00000182796	TMEM198B	transmembrane protein 198B (pseudogene)
1,892	ENSG00000186329	TMEM212	transmembrane protein 212
1,135	ENSG00000172738	TMEM217	transmembrane protein 217
1,195	ENSG00000182107	TMEM30B	transmembrane protein 30B
1,573	ENSG00000171729	TMEM51	transmembrane protein 51
1,226	ENSG00000121900	TMEM54	transmembrane protein 54
1,481	ENSG00000196187	TMEM63A	transmembrane protein 63A
1,009	ENSG00000137103	TMEM8B	transmembrane protein 8B
1,567	ENSG00000137747	TMPRSS13	transmembrane protease, serine 13
1,337	ENSG00000184012	TMPRSS2	transmembrane protease, serine 2
2,294	ENSG00000160183	TMPRSS3	transmembrane protease, serine 3
2,362	ENSG00000041982	TNC	tenascin C
2,899	ENSG00000185215	TNFAIP2	TNF alpha induced protein 2
3,713	ENSG00000118503	TNFAIP3	TNF alpha induced protein 3
1,621	ENSG00000104689	TNFRSF10A	TNF receptor superfamily member 10a
1,999	ENSG00000120889	TNFRSF10B	TNF receptor superfamily member 10b
2,265	ENSG00000173530	TNFRSF10D	TNF receptor superfamily member 10d
1,97	ENSG00000006327	TNFRSF12A	TNF receptor superfamily member 12A
2,551	ENSG00000127863	TNFRSF19	TNF receptor superfamily member 19
2,088	ENSG00000146072	TNFRSF21	TNF receptor superfamily member 21
1,221	ENSG00000243509	TNFRSF6B	TNF receptor superfamily member 6b
1,759	ENSG00000248871	TNFSF12-TN	TNFSF12-TNFSF13 readthrough
3,102	ENSG00000125657	TNFSF9	TNF superfamily member 9
1,452	ENSG00000149115	TNKS1BP1	tankyrase 1 binding protein 1
1,417	ENSG00000183864	TOB2	transducer of ERBB2, 2
1,144	ENSG00000025772	TOMM34	translocase of outer mitochondrial membrane 34
1,016	ENSG00000198900	TOP1	topoisomerase (DNA) I
1,158	ENSG00000103460	TOX3	TOX high mobility group box family member 3
1,625	ENSG00000067369	TP53BP1	tumor protein p53 binding protein 1
3,191	ENSG00000143514	TP53BP2	tumor protein p53 binding protein 2
1,346	ENSG00000115129	TP53I3	tumor protein p53 inducible protein 3
2,516	ENSG00000146242	TPBG	trophoblast glycoprotein
1,649	ENSG00000171368	TPPP	tubulin polymerization promoting protein
2,594	ENSG00000159713	TPPP3	tubulin polymerization promoting protein family member 3
2,265	ENSG00000056558	TRAF1	TNF receptor associated factor 1
1,359	ENSG00000182606	TRAK1	trafficking kinesin protein 1
3,412	ENSG00000173334	TRIB1	tribbles pseudokinase 1
1,012	ENSG00000130726	TRIM28	tripartite motif containing 28
2,339	ENSG00000137699	TRIM29	tripartite motif containing 29
1,345	ENSG00000134253	TRIM45	tripartite motif containing 45
1,857	ENSG00000132481	TRIM47	tripartite motif containing 47
1,05	ENSG00000038382	TRIO	trio Rho guanine nucleotide exchange factor
1,498	ENSG00000125733	TRIP10	thyroid hormone receptor interactor 10
1,495	ENSG00000253368	TRNP1	TMF1-regulated nuclear protein 1
1,026	ENSG00000130529	TRPM4	transient receptor potential cation channel subfamily M member 4
1,099	ENSG00000165699	TSC1	tuberous sclerosis 1
1,086	ENSG00000103197	TSC2	tuberous sclerosis 2
2,906	ENSG00000196428	TSC22D2	TSC22 domain family member 2
1,607	ENSG00000099282	TSPAN15	tetraspanin 15
1,611	ENSG00000005379	TSPOAP1	TSPO associated protein 1
1,09	ENSG00000187189	TSPYL4	TSPY like 4
1,738	ENSG00000214021	TTLL3	tubulin tyrosine ligase like 3
1,381	ENSG00000135912	TTLL4	tubulin tyrosine ligase like 4
1,329	ENSG00000167553	TUBA1C	tubulin alpha 1c
1,129	ENSG00000137267	TUBB2A	tubulin beta 2A class IIa
1,396	ENSG00000137285	TUBB2B	tubulin beta 2B class IIb
1,161	ENSG00000104833	TUBB4A	tubulin beta 4A class IVa
1,367	ENSG00000188229	TUBB4B	tubulin beta 4B class IVb
1,311	ENSG00000128159	TUBGCP6	tubulin gamma complex associated protein 6
2,317	ENSG00000143367	TUFT1	tuftelin 1
1,082	ENSG00000253352	TUG1	taurine up-regulated 1 (non-protein coding)
1,023	ENSG00000137073	UBAP2	ubiquitin associated protein 2
1,005	ENSG00000143569	UBAP2L	ubiquitin associated protein 2 like
2,519	ENSG00000150991	UBC	ubiquitin C
1,187	ENSG00000186591	UBE2H	ubiquitin conjugating enzyme E2 H
1,809	ENSG00000108106	UBE2S	ubiquitin conjugating enzyme E2 S
1,01	ENSG00000177414	UBE2U	ubiquitin conjugating enzyme E2 U (putative)
1,292	ENSG00000158062	UBXN11	UBX domain protein 11
2,131	ENSG00000214049	UCA1	urothelial cancer associated 1 (non-protein coding)
1,319	ENSG00000148154	UGCG	UDP-glucose ceramide glucosyltransferase
2,083	ENSG00000174607	UGT8	UDP glycosyltransferase 8
1,013	ENSG00000124602	UNC5CL	unc-5 family C-terminal like
1,339	ENSG00000005007	UPF1	UPF1, RNA helicase and ATPase
1,016	ENSG00000114638	UPK1B	uroplakin 1B
1,055	ENSG00000106608	URGCP	upregulator of cell proliferation
1,988	ENSG00000006611	USH1C	USH1 protein network component harmonin
1,242	ENSG00000102226	USP11	ubiquitin specific peptidase 11
1,815	ENSG00000154914	USP43	ubiquitin specific peptidase 43
1,194	ENSG00000132952	USPL1	ubiquitin specific peptidase like 1
1,08	ENSG00000168140	VASN	vasorin
1,143	ENSG00000171724	VAT1L	vesicle amine transport 1 like
1,21	ENSG00000038427	VCAN	versican
2,057	ENSG00000112715	VEGFA	vascular endothelial growth factor A
1,574	ENSG00000197415	VEPH1	ventricular zone expressed PH domain containing 1
1,087	ENSG00000136451	VEZF1	vascular endothelial zinc finger 1
1,221	ENSG00000102243	VGLL1	vestigial like family member 1
1,346	ENSG00000206538	VGLL3	vestigial like family member 3
2,071	ENSG00000127831	VIL1	villin 1
1,029	ENSG00000136059	VILL	villin like
2,414	ENSG00000139722	VPS37B	VPS37B, ESCRT-I subunit
1,208	ENSG00000019102	VSIG2	V-set and immunoglobulin domain containing 2
1,366	ENSG00000132821	VSTM2L	V-set and transmembrane domain containing 2 like
1,831	ENSG00000134258	VTCN1	V-set domain containing T-cell activation inhibitor 1
2,958	ENSG00000199990	VTRNA1-1	vault RNA 1-1
7,969	ENSG00000202515	VTRNA1-3	vault RNA 1-3
1,034	ENSG00000062650	WAPL	WAPL cohesin release factor
2,239	ENSG00000227232	WASH7P	WAS protein family homolog 7 pseudogene
1,191	ENSG00000099290	WASHC2A/W	WASH complex subunit 2C
1,07	ENSG00000157796	WDR19	WD repeat domain 19
1,537	ENSG00000184465	WDR27	WD repeat domain 27
1,206	ENSG00000085433	WDR47	WD repeat domain 47
1,88	ENSG00000178252	WDR6	WD repeat domain 6
1,222	ENSG00000152763	WDR78	WD repeat domain 78
1,634	ENSG00000161996	WDR90	WD repeat domain 90
1,194	ENSG00000105875	WDR91	WD repeat domain 91
3,19	ENSG00000166483	WEE1	WEE1 G2 checkpoint kinase
2,312	ENSG00000101443	WFDC2	WAP four-disulfide core domain 2
1,315	ENSG00000095397	WHRN	whirlin
2,225	ENSG00000165238	WNK2	WNK lysine deficient protein kinase 2
1,932	ENSG00000135925	WNT10A	Wnt family member 10A
1,167	ENSG00000188064	WNT7B	Wnt family member 7B
1,518	ENSG00000109046	WSB1	WD repeat and SOCS box containing 1

2,021	ENSG00000237515	SHISA9	shisa family member 9
1,94	ENSG00000138771	SHROOM3	shroom family member 3
1,565	ENSG00000160584	SIK3	SIK family kinase 3
1,037	ENSG00000197555	SIPA1L1	signal induced proliferation associated 1 like 1
1,79	ENSG00000096717	SIRT1	sirtuin 1
1,597	ENSG00000100625	SIX4	SIX homeobox 4
2,608	ENSG00000064651	SLC12A2	solute carrier family 12 member 2
1,306	ENSG00000124067	SLC12A4	solute carrier family 12 member 4
1,132	ENSG00000113504	SLC12A7	solute carrier family 12 member 7
1,624	ENSG00000118596	SLC16A7	solute carrier family 16 member 7
1,549	ENSG00000117479	SLC19A2	solute carrier family 19 member 2
1,534	ENSG00000105281	SLC1A5	solute carrier family 1 member 5
1,452	ENSG00000144136	SLC20A1	solute carrier family 20 member 1
1,132	ENSG00000197375	SLC22A5	solute carrier family 22 member 5
1,268	ENSG00000148339	SLC25A25	solute carrier family 25 member 25
1,417	ENSG00000153291	SLC25A27	solute carrier family 25 member 27
2,213	ENSG00000197119	SLC25A29	solute carrier family 25 member 29
1,27	ENSG00000091138	SLC26A3	solute carrier family 26 member 3
1,158	ENSG00000130304	SLC27A1	solute carrier family 27 member 1
2,107	ENSG00000197506	SLC28A3	solute carrier family 28 member 3
1,938	ENSG00000174669	SLC29A2	solute carrier family 29 member 2
1,942	ENSG00000117394	SLC2A1	solute carrier family 2 member 1
1,94	ENSG00000059804	SLC2A3	solute carrier family 2 member 3
1,847	ENSG00000157765	SLC34A2	solute carrier family 34 member 2
1,171	ENSG00000100036	SLC35E4	solute carrier family 35 member E4
2,367	ENSG00000110660	SLC35F2	solute carrier family 35 member F2
1,066	ENSG00000183780	SLC35F3	solute carrier family 35 member F3
1,502	ENSG00000111371	SLC38A1	solute carrier family 38 member 1
1,331	ENSG00000169507	SLC38A11	solute carrier family 38 member 11
2,534	ENSG00000168003	SLC3A2	solute carrier family 3 member 2
1,702	ENSG00000133065	SLC41A1	solute carrier family 41 member 1
1,099	ENSG00000143036	SLC44A3	solute carrier family 44 member 3
1,018	ENSG00000022567	SLC45A4	solute carrier family 45 member 4
1,201	ENSG00000114923	SLC4A3	solute carrier family 4 member 3
1,693	ENSG00000080493	SLC4A4	solute carrier family 4 member 4
1,54	ENSG00000033867	SLC4A7	solute carrier family 4 member 7
1,857	ENSG00000100170	SLC5A1	solute carrier family 5 member 1
1,64	ENSG00000154025	SLC5A10	solute carrier family 5 member 10
1,111	ENSG00000198743	SLC5A3	solute carrier family 5 member 3
1,724	ENSG00000117834	SLC5A9	solute carrier family 5 member 9
2,022	ENSG00000174358	SLC6A19	solute carrier family 6 member 19
1,782	ENSG00000131389	SLC6A6	solute carrier family 6 member 6
2,737	ENSG00000139514	SLC7A1	solute carrier family 7 member 1
3,116	ENSG00000103257	SLC7A5	solute carrier family 7 member 5
2,218	ENSG00000260727	SLC7A5P1	solute carrier family 7 member 5 pseudogene 1
2,687	ENSG00000258186	SLC7A5P2	solute carrier family 7 member 5 pseudogene 2
1,703	ENSG00000090020	SLC9A1	solute carrier family 9 member A1
1,116	ENSG00000066230	SLC9A3	solute carrier family 9 member A3
1,452	ENSG00000176463	SLCO3A1	solute carrier organic anion transporter family member 3A1
1,096	ENSG00000101187	SLCO4A1	solute carrier organic anion transporter family member 4A1
1,163	ENSG00000154760	SLFN13	schlafen family member 13
1,609	ENSG00000065613	SLK	STE20 like kinase
2,349	ENSG00000166949	SMAD3	SMAD family member 3
1,044	ENSG00000170545	SMAGP	small cell adhesion glycoprotein
1,822	ENSG00000127616	SMARCA4	SWI/SNF related, matrix associated, actin dependent regulator of chromatin, subfamily a, member 4
1,265	ENSG00000198887	SMC5	structural maintenance of chromosomes 5
1,14	ENSG00000116698	SMG7	SMG7, nonsense mediated mRNA decay factor
2,442	ENSG00000172062	SMN1/SMN2	survival of motor neuron 1, telomeric
2,404	ENSG00000088826	SMOX	spermine oxidase
1,431	ENSG00000103056	SMPD3	sphingomyelin phosphodiesterase 3
1,342	ENSG00000130768	SMPDL3B	sphingomyelin phosphodiesterase acid like 3B
2,266	ENSG00000198742	SMURF1	SMAD specific E3 ubiquitin protein ligase 1
1,069	ENSG00000132639	SNAP25	synaptosome associated protein 25
1,416	ENSG00000247092	SNHG10	small nucleolar RNA host gene 10
2,22	ENSG00000163877	SNIP1	Smad nuclear interacting protein 1
1,265	ENSG00000184602	SNN	stannin
3,97	ENSG00000206811	SNORA10	small nucleolar RNA, H/ACA box 10
3,539	ENSG00000238363	SNORA13	small nucleolar RNA, H/ACA box 13
6,212	ENSG00000206910	SNORA29	small nucleolar RNA, H/ACA box 29
2,204	ENSG00000206612	SNORA2A	small nucleolar RNA, H/ACA box 2A
4,454	ENSG00000207313	SNORA2B	small nucleolar RNA, H/ACA box 2B
3,011	ENSG00000238961	SNORA47	small nucleolar RNA, H/ACA box 47
1,282	ENSG00000201772	SNORA5C	small nucleolar RNA, H/ACA box 5C
3,094	ENSG00000206760	SNORA6	small nucleolar RNA, H/ACA box 6
3,971	ENSG00000201302	SNORA65	small nucleolar RNA, H/ACA box 65
2,946	ENSG00000206869	SNORA70F	small nucleolar RNA, H/ACA box 70F
1,804	ENSG00000200959	SNORA74A	small nucleolar RNA, H/ACA box 74A
3,925	ENSG00000207088	SNORA7B	small nucleolar RNA, H/ACA box 7B
4,227	ENSG00000206633	SNORA80B	small nucleolar RNA, H/ACA box 80B
2,333	ENSG00000239183	SNORA84	small nucleolar RNA, H/ACA box 84
5,748	ENSG00000199753	SNORD104	small nucleolar RNA, C/D box 104
3,687	ENSG00000251815	SNORD116-26	small nucleolar RNA, C/D box 116-26
5,897	ENSG00000207245	SNORD116-29	small nucleolar RNA, C/D box 116-29
4,893	ENSG00000207014	SNORD116-3	small nucleolar RNA, C/D box 116-3
1,658	ENSG00000207191	SNORD116-5	small nucleolar RNA, C/D box 116-5
7,213	ENSG00000212304	SNORD12	small nucleolar RNA, C/D box 12
7,032	ENSG00000238300	SNORD121B	small nucleolar RNA, C/D box 121B
8,772	ENSG00000209042	SNORD12C	small nucleolar RNA, C/D box 12C
4,438	ENSG00000206941	SNORD15A	small nucleolar RNA, C/D box 15A
4,435	ENSG00000199673	SNORD16	small nucleolar RNA, C/D box 16
4,392	ENSG00000200530	SNORD35B	small nucleolar RNA, C/D box 35B
5,632	ENSG00000202093	SNORD58C	small nucleolar RNA, C/D box 58C
5,119	ENSG00000207031	SNORD59A	small nucleolar RNA, C/D box 59A
4,454	ENSG00000223224	SNORD71	small nucleolar RNA, C/D box 71
4,716	ENSG00000200785	SNORD8	small nucleolar RNA, C/D box 8
5,79	ENSG00000209482	SNORD83A	small nucleolar RNA, C/D box 83A
4,078	ENSG00000208772	SNORD94	small nucleolar RNA, C/D box 94
1,235	ENSG00000144028	SNRNP200	small nuclear ribonucleoprotein U5 subunit 200
1,148	ENSG00000104852	SNRNP70	small nuclear ribonucleoprotein U1 subunit 70
2,119	ENSG00000184557	SOCS3	suppressor of cytokine signaling 3
1,215	ENSG00000159140	SON	SON DNA binding protein
1,614	ENSG00000198142	SOWAHC	sosondowah ankyrin repeat domain family member C
1,499	ENSG00000164736	SOX17	SRY-box 17
3,186	ENSG00000124766	SOX4	SRY-box 4
3,061	ENSG00000125398	SOX9	SRY-box 9
1,004	ENSG00000105866	SP4	Sp4 transcription factor
2,506	ENSG00000104450	SPAG1	sperm associated antigen 1
1,334	ENSG00000008294	SPAG9	sperm associated antigen 9
1,519	ENSG00000182957	SPATA13	spermatogenesis associated 13
1,136	ENSG00000158480	SPATA2	spermatogenesis associated 2
1,071	ENSG00000196141	SPATS2L	spermatogenesis associated serine rich 2 like
1,097	ENSG00000152582	SPEF2	sperm flagellar 2
2,118	ENSG00000065526	SPEN	spen family transcriptional repressor
3,235	ENSG00000176170	SPHK1	sphingosine kinase 1
2,403	ENSG00000166145	SPINT1	serine peptidase inhibitor, Kunitz type 1
1,476	ENSG00000167642	SPINT2	serine peptidase inhibitor, Kunitz type 2
1,858	ENSG00000183018	SPNS2	sphingolipid transporter 2
1,086	ENSG00000144228	SPOPL	speckle type BTB/POZ protein like
1,253	ENSG00000118785	SPP1	secreted phosphoprotein 1
1,252	ENSG00000005206	SPPL2B	signal peptide peptidase like 2B
1,109	ENSG00000198369	SPRED2	sprouty related EVH1 domain containing 2
1,238	ENSG00000136158	SPRY2	sprouty RTK signaling antagonist 2
2,337	ENSG00000171621	SPSB1	splA/ryanodine receptor domain and SOCS box containing 1
1,383	ENSG00000197694	SPTAN1	spectrin alpha, non-erythrocytic 1
1,54	ENSG00000115306	SPTBN1	spectrin beta, non-erythrocytic 1
2,22	ENSG00000104549	SQLE	squalene epoxidase
1,104	ENSG00000197122	SRC	SRC proto-oncogene, non-receptor tyrosine kinase
1,01	ENSG00000080603	SRCAP	Snf2 related CREBBP activator protein
1,553	ENSG00000198911	SREBF2	sterol regulatory element binding transcription factor 2
1,725	ENSG00000112658	SRF	serum response factor
1,103	ENSG00000196935	SRGAP1	SLIT-ROBO Rho GTPase activating protein 1
1,132	ENSG00000133226	SRRM1	serine and arginine repetitive matrix 1
1,893	ENSG00000167978	SRRM2	serine/arginine repetitive matrix 2
1,044	ENSG00000087087	SRRT	serrate, RNA effector molecule
1,773	ENSG00000154548	SRSF12	serine and arginine rich splicing factor 12

1,652	ENSG00000113645	WWC1	WW and C2 domain containing 1
1,249	ENSG00000198373	WWP2	WW domain containing E3 ubiquitin protein ligase 2
1,445	ENSG00000018408	WWTR1	WW domain containing transcription regulator 1
1,967	ENSG00000180667	YOD1	YOD1 deubiquitinase
1,355	ENSG00000175155	YPEL2	yippee like 2
1,658	ENSG00000205189	ZBTB10	zinc finger and BTB domain containing 10
1,518	ENSG00000109906	ZBTB16	zinc finger and BTB domain containing 16
1,809	ENSG00000181472	ZBTB2	zinc finger and BTB domain containing 2
2,039	ENSG00000181722	ZBTB20	zinc finger and BTB domain containing 20
1,355	ENSG00000177125	ZBTB34	zinc finger and BTB domain containing 34
1,114	ENSG00000184677	ZBTB40	zinc finger and BTB domain containing 40
2,362	ENSG00000169155	ZBTB43	zinc finger and BTB domain containing 43
1,268	ENSG00000178951	ZBTB7A	zinc finger and BTB domain containing 7A
4,604	ENSG00000163874	ZC3H12A	zinc finger CCCH-type containing 12A
1,272	ENSG00000105939	ZC3HAV1	zinc finger CCCH-type containing, antiviral 1
1,195	ENSG00000033030	ZCCHC8	zinc finger CCHC-type containing 8
1,105	ENSG00000159714	ZDHHC1	zinc finger DHHC-type containing 1
2,023	ENSG00000178381	ZFAND2A	zinc finger AN1-type containing 2A
1,408	ENSG00000133858	ZFC3H1	zinc finger C3H1-type containing
2,784	ENSG00000128016	ZFP36	ZFP36 ring finger protein
1,277	ENSG00000185650	ZFP36L1	ZFP36 ring finger protein like 1
2,543	ENSG00000162078	ZG16B	zymogen granule protein 16B
1,056	ENSG00000178764	ZHX2	zinc fingers and homeoboxes 2
1,475	ENSG00000108175	ZMIZ1	zinc finger MIZ-type containing 1
1,607	ENSG00000122515	ZMIZ2	zinc finger MIZ-type containing 2
1,151	ENSG00000115568	ZNF142	zinc finger protein 142
3,271	ENSG00000197279	ZNF165	zinc finger protein 165
1,758	ENSG00000171940	ZNF217	zinc finger protein 217
1,361	ENSG00000165804	ZNF219	zinc finger protein 219
1,079	ENSG00000159917	ZNF235	zinc finger protein 235
1,153	ENSG00000130856	ZNF236	zinc finger protein 236
1,307	ENSG00000152454	ZNF256	zinc finger protein 256
1,275	ENSG00000185947	ZNF267	zinc finger protein 267
1,307	ENSG00000162702	ZNF281	zinc finger protein 281
2,157	ENSG00000170684	ZNF296	zinc finger protein 296
1,023	ENSG00000130803	ZNF317	zinc finger protein 317
1,279	ENSG00000198026	ZNF335	zinc finger protein 335
1,239	ENSG00000197647	ZNF433	zinc finger protein 433
1,029	ENSG00000083838	ZNF446	zinc finger protein 446
1,679	ENSG00000105732	ZNF574	zinc finger protein 574
1,251	ENSG00000166716	ZNF592	zinc finger protein 592
1,352	ENSG00000168916	ZNF608	zinc finger protein 608
1,593	ENSG00000171163	ZNF692	zinc finger protein 692
1,531	ENSG00000147180	ZNF711	zinc finger protein 711
1,039	ENSG00000221874	ZNF816-ZNI	ZNF816-ZNF321P readthrough
1,375	ENSG00000257446	ZNF878	zinc finger protein 878
1,293	ENSG00000170044	ZPLD1	zona pellucida like domain containing 1
1,263	ENSG00000229956	ZRANB2-AS	ZRANB2 antisense RNA 2 (head to head)
1,46	ENSG00000219891	ZSCAN12P1	zinc finger and SCAN domain containing 12 pseudogene 1
1,044	ENSG00000121413	ZSCAN18	zinc finger and SCAN domain containing 18
2,171	ENSG00000132003	ZSWIM4	zinc finger SWIM-type containing 4
1,534	ENSG00000130449	ZSWIM6	zinc finger SWIM-type containing 6
1,487	ENSG00000198455	ZXDB	zinc finger, X-linked, duplicated B
1,092	ENSG00000159840	ZYX	zyxin

1,487	ENSG00000084112	SSH1	slingshot protein phosphatase 1
2,452	ENSG00000149418	ST14	suppression of tumorigenicity 14
1,55	ENSG00000133121	STARD13	StAR related lipid transfer domain containing 13
1,48	ENSG00000159167	STC1	stanniocalcin 1
2,084	ENSG00000168439	STIP1	stress induced phosphoprotein 1
2,296	ENSG00000164543	STK17A	serine/threonine kinase 17a
1,826	ENSG00000130413	STK33	serine/threonine kinase 33
1,195	ENSG00000125834	STK35	serine/threonine kinase 35
1,125	ENSG00000163482	STK36	serine/threonine kinase 36
1,222	ENSG00000198648	STK39	serine/threonine kinase 39
1,82	ENSG00000196182	STK40	serine/threonine kinase 40
1,298	ENSG00000173320	STOX2	storkhead box 2
2,462	ENSG00000166900	STX3	syntaxin 3
1,441	ENSG00000168952	STXBP6	syntaxin binding protein 6
1,54	ENSG00000064607	SUGP2	SURP and G-patch domain containing 2
2,022	ENSG00000198075	SULT1C4	sulfotransferase family 1C member 4
1,156	ENSG00000164828	SUN1	Sad1 and UNC84 domain containing 1
1,268	ENSG00000100647	SUSD6	sushi domain containing 6
1,116	ENSG00000264538	SUZ12P1	SUZ12 polycomb repressive complex 2 subunit pseudogene 1
1,586	ENSG00000197321	SVIL	supervillin
1,583	ENSG00000133789	SWAP70	SWAP switching B-cell complex subunit 70
1,656	ENSG00000205078	SYCE1L	synaptonemal complex central element protein 1 like
1,304	ENSG00000097096	SYDE2	synapse defective Rho GTPase homolog 2
1,034	ENSG00000100321	SYNGR1	synaptogyrin 1
2,225	ENSG00000078269	SYNJ2	synaptojanin 2
1,599	ENSG00000254806	SYS1-DBNDD2	SYS1-DBNDD2 readthrough (NMD candidate)
1,745	ENSG00000149043	SYT8	synaptotagmin 8
1,064	ENSG00000138162	TACC2	transforming acidic coiled-coil containing protein 2
3,458	ENSG00000184292	TACSTD2	tumor associated calcium signal transducer 2
1,135	ENSG00000103168	TAF1C	TATA-box binding protein associated factor, RNA polymerase I subunit C
1,721	ENSG00000166012	TAF1D	TATA-box binding protein associated factor, RNA polymerase I subunit D
1,331	ENSG00000141384	TAF4B	TATA-box binding protein associated factor 4b
1,135	ENSG00000099992	TBC1D10A	TBC1 domain family member 10A
1,868	ENSG00000065491	TBC1D22B	TBC1 domain family member 22B
1,921	ENSG00000204634	TBC1D8	TBC1 domain family member 8
1,146	ENSG00000165929	TC2N	tandem C2 domains, nuclear
1,553	ENSG00000113649	TCERG1	transcription elongation regulator 1
1,008	ENSG00000071564	TCF3	transcription factor 3
1,888	ENSG00000134827	TCN1	transcobalamin 1
1,11	ENSG00000120438	TCP1	t-complex 1
1,181	ENSG00000188396	TCTEX1D4	Tctex1 domain containing 4
1,219	ENSG00000180190	TDRP	testis development related protein
1,251	ENSG00000187079	TEAD1	TEA domain transcription factor 1
1,009	ENSG00000135605	TEC	tec protein tyrosine kinase
1,246	ENSG00000092850	TEKT2	tektin 2
3,201	ENSG00000135269	TES	testin LIM domain protein
1,231	ENSG00000088992	TESC	tescalcin
1,236	ENSG00000136891	TEX10	testis expressed 10
1,931	ENSG00000087510	TFAP2C	transcription factor AP-2 gamma
1,066	ENSG00000112561	TFEB	transcription factor EB
2,587	ENSG00000160182	TFF1	trefoil factor 1
1,992	ENSG00000160181	TFF2	trefoil factor 2
3,89	ENSG00000105825	TFPI2	tissue factor pathway inhibitor 2
1,525	ENSG00000163235	TGFA	transforming growth factor alpha
3,398	ENSG00000092969	TGFB2	transforming growth factor beta 2
1,792	ENSG00000177426	TGIF1	TGFB induced factor homeobox 1
1,729	ENSG00000118707	TGIF2	TGFB induced factor homeobox 2
2,103	ENSG00000137801	THBS1	thrombospondin 1
1,089	ENSG00000054118	THRAP3	thyroid hormone receptor associated protein 3
1,743	ENSG00000187720	THSD4	thrombospondin type 1 domain containing 4
1,884	ENSG00000146426	TIAM2	T-cell lymphoma invasion and metastasis 2
2,266	ENSG00000127666	TICAM1	toll like receptor adaptor molecule 1
1,213	ENSG00000142910	TINAGL1	tubulointerstitial nephritis antigen like 1
2,119	ENSG00000163659	TIPARP	TCDD inducible poly(ADP-ribose) polymerase
2,157	ENSG00000104067	TJP1	tight junction protein 1
2,941	ENSG00000119139	TJP2	tight junction protein 2
1,827	ENSG00000196781	TLE1	transducin like enhancer of split 1
1,556	ENSG00000065717	TLE2	transducin like enhancer of split 2
1,885	ENSG00000106829	TLE4	transducin like enhancer of split 4
1,366	ENSG00000187554	TLR5	toll like receptor 5
3,864	ENSG00000169908	TM4SF1	transmembrane 4 L six family member 1
1,763	ENSG00000103534	TMC5	transmembrane channel like 5
1,651	ENSG00000133069	TMCC2	transmembrane and coiled-coil domain family 2
1,959	ENSG00000006118	TMEM132A	transmembrane protein 132A
3,053	ENSG00000181264	TMEM136	transmembrane protein 136
2,496	ENSG00000249992	TMEM158	transmembrane protein 158 (gene/pseudogene)
1,053	ENSG00000152128	TMEM163	transmembrane protein 163
2,497	ENSG00000157111	TMEM171	transmembrane protein 171
1,279	ENSG00000164855	TMEM184A	transmembrane protein 184A
1,115	ENSG00000198792	TMEM184B	transmembrane protein 184B
1,245	ENSG00000226479	TMEM185B	transmembrane protein 185B
1,173	ENSG00000182796	TMEM198B	transmembrane protein 198B (pseudogene)
1,39	ENSG00000135048	TMEM2	transmembrane protein 2
1,662	ENSG00000186329	TMEM212	transmembrane protein 212
1,669	ENSG00000172738	TMEM217	transmembrane protein 217
2,881	ENSG00000262481	TMEM256-PLS	TMEM256-PLSCR3 readthrough (NMD candidate)
1,426	ENSG00000182107	TMEM30B	transmembrane protein 30B
1,013	ENSG00000170876	TMEM43	transmembrane protein 43
1,998	ENSG00000171729	TMEM51	transmembrane protein 51
1,202	ENSG00000121900	TMEM54	transmembrane protein 54
1,347	ENSG00000143001	TMEM61	transmembrane protein 61
1,674	ENSG00000196187	TMEM63A	transmembrane protein 63A
1,134	ENSG00000137103	TMEM8B	transmembrane protein 8B
1,727	ENSG00000137747	TMPRSS13	transmembrane protease, serine 13
1,738	ENSG00000184012	TMPRSS2	transmembrane protease, serine 2
2,076	ENSG00000160183	TMPRSS3	transmembrane protease, serine 3
2,187	ENSG00000041982	TNC	tenascin C
2,754	ENSG00000185215	TNFAIP2	TNF alpha induced protein 2
4,536	ENSG00000118503	TNFAIP3	TNF alpha induced protein 3
1,29	ENSG00000145779	TNFAIP8	TNF alpha induced protein 8
2,629	ENSG00000104689	TNFRSF10A	TNF receptor superfamily member 10a
2,615	ENSG00000120889	TNFRSF10B	TNF receptor superfamily member 10b
3,237	ENSG00000173530	TNFRSF10D	TNF receptor superfamily member 10d
2,095	ENSG00000006327	TNFRSF12A	TNF receptor superfamily member 12A
2,398	ENSG00000127863	TNFRSF19	TNF receptor superfamily member 19
2,082	ENSG00000146072	TNFRSF21	TNF receptor superfamily member 21
1,049	ENSG00000049249	TNFRSF9	TNF receptor superfamily member 9
3,095	ENSG00000125657	TNFSF9	TNF superfamily member 9
1,048	ENSG00000145901	TNIP1	TNFAIP3 interacting protein 1
1,034	ENSG00000173273	TNKS	tankyrase
1,704	ENSG00000149115	TNKS1BP1	tankyrase 1 binding protein 1
1,516	ENSG00000183864	TOB2	transducer of ERBB2, 2
1,971	ENSG00000025772	TOMM34	translocase of outer mitochondrial membrane 34
1,495	ENSG00000198900	TOP1	topoisomerase (DNA) I
1,25	ENSG00000103460	TOX3	TOX high mobility group box family member 3
1,359	ENSG00000067369	TP53BP1	tumor protein p53 binding protein 1
3,98	ENSG00000143514	TP53BP2	tumor protein p53 binding protein 2
1,147	ENSG00000115129	TP53I3	tumor protein p53 inducible protein 3
2,939	ENSG00000146242	TPBG	trophoblast glycoprotein
1,145	ENSG00000167460	TPM4	tropomyosin 4
1,685	ENSG00000171368	TPPP	tubulin polymerization promoting protein
2,608	ENSG00000159713	TPPP3	tubulin polymerization promoting protein family member 3
1,103	ENSG00000136527	TRA2B	transformer 2 beta homolog
3,207	ENSG00000056558	TRAF1	TNF receptor associated factor 1
1,371	ENSG00000056972	TRAF3IP2	TRAF3 interacting protein 2
1,333	ENSG00000182606	TRAK1	trafficking kinesin protein 1
3,439	ENSG00000173334	TRIB1	tribbles pseudokinase 1
1,106	ENSG00000130726	TRIM28	tripartite motif containing 28
3,172	ENSG00000137699	TRIM29	tripartite motif containing 29
1,032	ENSG00000197323	TRIM33	tripartite motif containing 33
1,426	ENSG00000152503	TRIM36	tripartite motif containing 36
1,336	ENSG00000134253	TRIM45	tripartite motif containing 45
2,363	ENSG00000132481	TRIM47	tripartite motif containing 47
1,099	ENSG00000169871	TRIM56	tripartite motif containing 56
1,156	ENSG00000038382	TRIO	trio Rho guanine nucleotide exchange factor

1,682	ENSG00000125733	TRIP10	thyroid hormone receptor interactor 10
1,03	ENSG00000104907	TRMT1	tRNA methyltransferase 1
1,706	ENSG00000253368	TRNP1	TMF1-regulated nuclear protein 1
1,078	ENSG00000165699	TSC1	tuberous sclerosis 1
1,187	ENSG00000103197	TSC2	tuberous sclerosis 2
3,235	ENSG00000196428	TSC22D2	TSC22 domain family member 2
1,781	ENSG00000099282	TSPAN15	tetraspanin 15
1,595	ENSG00000005379	TSPOAP1	TSPO associated protein 1
1,74	ENSG00000214021	TTLL3	tubulin tyrosine ligase like 3
1,282	ENSG00000135912	TTLL4	tubulin tyrosine ligase like 4
1,022	ENSG00000167552	TUBA1A	tubulin alpha 1a
2,392	ENSG00000167553	TUBA1C	tubulin alpha 1c
1,63	ENSG00000137267	TUBB2A	tubulin beta 2A class IIa
1,853	ENSG00000137285	TUBB2B	tubulin beta 2B class IIb
1,915	ENSG00000188229	TUBB4B	tubulin beta 4B class IVb
1,518	ENSG00000176014	TUBB6	tubulin beta 6 class V
1,178	ENSG00000128159	TUBGCP6	tubulin gamma complex associated protein 6
2,421	ENSG00000143367	TUFT1	tuftelin 1
1,059	ENSG00000078246	TULP3	tubby like protein 3
1,326	ENSG00000086712	TXLNG	taxilin gamma
1,644	ENSG00000198431	TXNRD1	thioredoxin reductase 1
1,1	ENSG00000165006	UBAP1	ubiquitin associated protein 1
1,24	ENSG00000137073	UBAP2	ubiquitin associated protein 2
1,161	ENSG00000143569	UBAP2L	ubiquitin associated protein 2 like
1,22	ENSG00000154127	UBASH3B	ubiquitin associated and SH3 domain containing B
2,854	ENSG00000150991	UBC	ubiquitin C
1,53	ENSG00000072401	UBE2D1	ubiquitin conjugating enzyme E2 D1
1,854	ENSG00000186591	UBE2H	ubiquitin conjugating enzyme E2 H
2,06	ENSG00000108106	UBE2S	ubiquitin conjugating enzyme E2 S
1,215	ENSG00000188021	UBQLN2	ubiquilin 2
1,329	ENSG00000158062	UBXN11	UBX domain protein 11
2,603	ENSG00000214049	UCA1	urothelial cancer associated 1 (non-protein coding)
1,953	ENSG00000148154	UGCG	UDP-glucose ceramide glucosyltransferase
1,916	ENSG00000174607	UGT8	UDP glycosyltransferase 8
1,02	ENSG00000131015	ULBP2	UL16 binding protein 2
1,369	ENSG00000131019	ULBP3	UL16 binding protein 3
1,322	ENSG00000005007	UPF1	UPF1, RNA helicase and ATPase
1,117	ENSG00000114638	UPK1B	uroplakin 1B
1,547	ENSG00000106608	URGCP	upregulator of cell proliferation
1,703	ENSG00000006611	USH1C	USH1 protein network component harmonin
1,452	ENSG00000102226	USP11	ubiquitin specific peptidase 11
1,511	ENSG00000152484	USP12	ubiquitin specific peptidase 12
1,041	ENSG00000124422	USP22	ubiquitin specific peptidase 22
1,461	ENSG00000170185	USP38	ubiquitin specific peptidase 38
1,309	ENSG00000106346	USP42	ubiquitin specific peptidase 42
2,068	ENSG00000154914	USP43	ubiquitin specific peptidase 43
1,125	ENSG00000170242	USP47	ubiquitin specific peptidase 47
1,283	ENSG00000148429	USP6NL	USP6 N-terminal like
1,216	ENSG00000132952	USPL1	ubiquitin specific peptidase like 1
1,39	ENSG00000168140	VASN	vasorin
1,011	ENSG00000125753	VASP	vasodilator-stimulated phosphoprotein
1,827	ENSG00000112715	VEGFA	vascular endothelial growth factor A
1,173	ENSG00000197415	VEPH1	ventricular zone expressed PH domain containing 1
1,476	ENSG00000136451	VEZF1	vascular endothelial zinc finger 1
1,989	ENSG00000206538	VGLL3	vestigial like family member 3
1,809	ENSG00000127831	VIL1	villin 1
1,351	ENSG00000136059	VILL	villin like
3,118	ENSG00000139722	VPS37B	VPS37B, ESCRT-I subunit
1,01	ENSG00000167987	VPS37C	VPS37C, ESCRT-I subunit
1,117	ENSG00000019102	VSIG2	V-set and immunoglobulin domain containing 2
1,239	ENSG00000132821	VSTM2L	V-set and transmembrane domain containing 2 like
1,777	ENSG00000134258	VTCN1	V-set domain containing T-cell activation inhibitor 1
2,033	ENSG00000199990	VTRNA1-1	vault RNA 1-1
7,264	ENSG00000202515	VTRNA1-3	vault RNA 1-3
1,124	ENSG00000062650	WAPL	WAPL cohesin release factor
2,273	ENSG00000227232	WASH7P	WAS protein family homolog 7 pseudogene
1,139	ENSG00000099290	WASHC2A/WA	WASH complex subunit 2C
1,631	ENSG00000184465	WDR27	WD repeat domain 27
1,029	ENSG00000163811	WDR43	WD repeat domain 43
1,849	ENSG00000085433	WDR47	WD repeat domain 47
1,862	ENSG00000178252	WDR6	WD repeat domain 6
1,543	ENSG00000161996	WDR90	WD repeat domain 90
1,069	ENSG00000105875	WDR91	WD repeat domain 91
3,748	ENSG00000166483	WEE1	WEE1 G2 checkpoint kinase
2,171	ENSG00000101443	WFDC2	WAP four-disulfide core domain 2
1,212	ENSG00000156232	WHAMM	WAS protein homolog associated with actin, golgi membranes and microtubules
1,265	ENSG00000095397	WHRN	whirlin
2,104	ENSG00000165238	WNK2	WNK lysine deficient protein kinase 2
1,882	ENSG00000135925	WNT10A	Wnt family member 10A
1,549	ENSG00000188064	WNT7B	Wnt family member 7B
1,812	ENSG00000109046	WSB1	WD repeat and SOCS box containing 1
1,107	ENSG00000146457	WTAP	WT1 associated protein
1,816	ENSG00000113645	WWC1	WW and C2 domain containing 1
1,746	ENSG00000018408	WWTR1	WW domain containing transcription regulator 1
2,344	ENSG00000180667	YOD1	YOD1 deubiquitinase
1,309	ENSG00000175155	YPEL2	yippee like 2
1,622	ENSG00000164924	YWHAZ	tyrosine 3-monooxygenase/tryptophan 5-monooxygenase activation protein zeta
1,804	ENSG00000205189	ZBTB10	zinc finger and BTB domain containing 10
1,069	ENSG00000109906	ZBTB16	zinc finger and BTB domain containing 16
2,177	ENSG00000181472	ZBTB2	zinc finger and BTB domain containing 2
2,161	ENSG00000181722	ZBTB20	zinc finger and BTB domain containing 20
1,728	ENSG00000177125	ZBTB34	zinc finger and BTB domain containing 34
1,008	ENSG00000184677	ZBTB40	zinc finger and BTB domain containing 40
2,962	ENSG00000169155	ZBTB43	zinc finger and BTB domain containing 43
1,317	ENSG00000168795	ZBTB5	zinc finger and BTB domain containing 5
1,215	ENSG00000178951	ZBTB7A	zinc finger and BTB domain containing 7A
4,645	ENSG00000163874	ZC3H12A	zinc finger CCCH-type containing 12A
1,307	ENSG00000149289	ZC3H12C	zinc finger CCCH-type containing 12C
1,857	ENSG00000105939	ZC3HAV1	zinc finger CCCH-type containing, antiviral 1
1,416	ENSG00000083223	ZCCHC6	zinc finger CCHC-type containing 6
1,544	ENSG00000033030	ZCCHC8	zinc finger CCHC-type containing 8
1,033	ENSG00000159714	ZDHHC1	zinc finger DHHC-type containing 1
1,952	ENSG00000178381	ZFAND2A	zinc finger AN1-type containing 2A
1,037	ENSG00000107372	ZFAND5	zinc finger AN1-type containing 5
1,571	ENSG00000133858	ZFC3H1	zinc finger C3H1-type containing
2,902	ENSG00000128016	ZFP36	ZFP36 ring finger protein
1,324	ENSG00000185650	ZFP36L1	ZFP36 ring finger protein like 1
2,143	ENSG00000162078	ZG16B	zymogen granule protein 16B
1,35	ENSG00000108175	ZMIZ1	zinc finger MIZ-type containing 1
1,396	ENSG00000122515	ZMIZ2	zinc finger MIZ-type containing 2
1,151	ENSG00000213762	ZNF134	zinc finger protein 134
1,018	ENSG00000115568	ZNF142	zinc finger protein 142
4,119	ENSG00000197279	ZNF165	zinc finger protein 165
1,013	ENSG00000166261	ZNF202	zinc finger protein 202
1,696	ENSG00000171940	ZNF217	zinc finger protein 217
1,212	ENSG00000165804	ZNF219	zinc finger protein 219
1,268	ENSG00000152454	ZNF256	zinc finger protein 256
1,96	ENSG00000185947	ZNF267	zinc finger protein 267
1,417	ENSG00000162702	ZNF281	zinc finger protein 281
2,826	ENSG00000170684	ZNF296	zinc finger protein 296
1,405	ENSG00000130803	ZNF317	zinc finger protein 317
1,479	ENSG00000198026	ZNF335	zinc finger protein 335
1,031	ENSG00000126746	ZNF384	zinc finger protein 384
1,353	ENSG00000160908	ZNF394	zinc finger protein 394
1,238	ENSG00000125945	ZNF436	zinc finger protein 436
1,014	ENSG00000083838	ZNF446	zinc finger protein 446
1,197	ENSG00000144026	ZNF514	zinc finger protein 514
1,188	ENSG00000101493	ZNF516	zinc finger protein 516
1,864	ENSG00000105732	ZNF574	zinc finger protein 574
1,668	ENSG00000166716	ZNF592	zinc finger protein 592
1,232	ENSG00000168916	ZNF608	zinc finger protein 608
1,001	ENSG00000102870	ZNF629	zinc finger protein 629
1,464	ENSG00000171163	ZNF692	zinc finger protein 692
1,04	ENSG00000140548	ZNF710	zinc finger protein 710
1,705	ENSG00000147180	ZNF711	zinc finger protein 711

1,01	ENSG00000178665	ZNF713	zinc finger protein 713
1,645	ENSG00000124201	ZNFX1	zinc finger NFX1-type containing 1
2,675	ENSG00000219891	ZSCAN12P1	zinc finger and SCAN domain containing 12 pseudogene 1
1,29	ENSG00000121413	ZSCAN18	zinc finger and SCAN domain containing 18
2,838	ENSG00000132003	ZSWIM4	zinc finger SWIM-type containing 4
1,924	ENSG00000130449	ZSWIM6	zinc finger SWIM-type containing 6
1,462	ENSG00000198205	ZXDA	zinc finger, X-linked, duplicated A
2,213	ENSG00000198455	ZXDB	zinc finger, X-linked, duplicated B
1,328	ENSG00000159840	ZYX	zyxin

Genes enriched in SP

Fold change vs liver	ID	Symbol	Entrez Gene Name
1,158	ENSG00000107331	ABCA2	ATP binding cassette subfamily A member 2
1,918	ENSG00000085563	ABCB1	ATP binding cassette subfamily B member 1
1,491	ENSG00000103222	ABCC1	ATP binding cassette subfamily C member 1
1,482	ENSG00000124574	ABCC10	ATP binding cassette subfamily C member 10
1,661	ENSG00000108846	ABCC3	ATP binding cassette subfamily C member 3
1,237	ENSG00000125257	ABCC4	ATP binding cassette subfamily C member 4
1,23	ENSG00000097007	ABL1	ABL proto-oncogene 1, non-receptor tyrosine kinase
1,172	ENSG00000099204	ABLIM1	actin binding LIM protein 1
1,051	ENSG00000159842	ABR	active BCR-related
1,262	ENSG00000087085	ACHE	acetylcholinesterase (Cartwright blood group)
1,335	ENSG00000154930	ACSS1	acyl-CoA synthetase short-chain family member 1
1,062	ENSG00000119640	ACYP1	acylphosphatase 1
1,184	ENSG00000137845	ADAM10	ADAM metallopeptidase domain 10
1,088	ENSG00000042980	ADAM28	ADAM metallopeptidase domain 28
1,216	ENSG00000151651	ADAM8	ADAM metallopeptidase domain 8
1,702	ENSG00000168615	ADAM9	ADAM metallopeptidase domain 9
1,689	ENSG00000105963	ADAP1	ArfGAP with dual PH domains 1
1,433	ENSG00000174233	ADCY6	adenylate cyclase 6
1,35	ENSG00000155897	ADCY8	adenylate cyclase 8
1,564	ENSG00000205336	ADGRG1	adhesion G protein-coupled receptor G1
1,794	ENSG00000148926	ADM	adrenomedullin
1,756	ENSG00000163485	ADORA1	adenosine A1 receptor
1,178	ENSG00000150594	ADRA2A	adrenoceptor alpha 2A
1,91	ENSG00000181026	AEN	apoptosis enhancing nuclease
1,306	ENSG00000196526	AFAP1	actin filament associated protein 1
2,08	ENSG00000130396	AFDN	afadin, adherens junction formation factor
1,662	ENSG00000157985	AGAP1	ArfGAP with GTPase domain, ankyrin repeat and PH domain 1
1,036	ENSG00000204149	AGAP6 (includes others)	ArfGAP with GTPase domain, ankyrin repeat and PH domain 5
1,005	ENSG00000026652	AGPAT4	1-acylglycerol-3-phosphate O-acyltransferase 4
1,883	ENSG00000106541	AGR2	anterior gradient 2, protein disulphide isomerase family member
1,029	ENSG00000173467	AGR3	anterior gradient 3, protein disulphide isomerase family member
1,211	ENSG00000135541	AHI1	Abelson helper integration site 1
1,057	ENSG00000106546	AHR	aryl hydrocarbon receptor
1,673	ENSG00000173209	AHSA2	activator of HSP90 ATPase homolog 2
1,213	ENSG00000196581	AJAP1	adherens junctions associated protein 1
1,22	ENSG00000140057	AK7	adenylate kinase 7
1,425	ENSG00000197976	AKAP17A	A-kinase anchoring protein 17A
2,002	ENSG00000118507	AKAP7	A-kinase anchoring protein 7
1,385	ENSG00000011243	AKAP8L	A-kinase anchoring protein 8 like
1,708	ENSG00000128918	ALDH1A2	aldehyde dehydrogenase 1 family member A2
2,099	ENSG00000196711	ALKAL1	ALK and LTK ligand 1
1,285	ENSG00000189292	ALKAL2	ALK and LTK ligand 2
1,324	ENSG00000262943	ALOX12P2	arachidonate 12-lipoxygenase pseudogene 2
1,503	ENSG00000178038	ALS2CL	ALS2 C-terminal like
2,674	ENSG00000114019	AMOTL2	angiomotin like 2
1,548	ENSG00000240038	AMY2B	amylase, alpha 2B (pancreatic)
1,242	ENSG00000254996	ANKHD1/ANKHD1-EIF4EBP3	ankyrin repeat and KH domain containing 1
1,107	ENSG00000176915	ANKLE2	ankyrin repeat and LEM domain containing 2
1,163	ENSG00000088448	ANKRD10	ankyrin repeat domain 10
1,149	ENSG00000167522	ANKRD11	ankyrin repeat domain 11
2,027	ENSG00000180071	ANKRD18A	ankyrin repeat domain 18A
1,334	ENSG00000230453	ANKRD18B	ankyrin repeat domain 18B
2,001	ENSG00000164236	ANKRD33B	ankyrin repeat domain 33B
1,806	ENSG00000135976	ANKRD36	ankyrin repeat domain 36
1,508	ENSG00000196912	ANKRD36B	ankyrin repeat domain 36B
1,223	ENSG00000137494	ANKRD42	ankyrin repeat domain 42
1,052	ENSG00000163516	ANKZF1	ankyrin repeat and zinc finger domain containing 1
1,624	ENSG00000185101	ANO9	anoctamin 9
1,171	ENSG00000122359	ANXA11	annexin A11
2,045	ENSG00000104537	ANXA13	annexin A13
2,056	ENSG00000138772	ANXA3	annexin A3
1,66	ENSG00000196975	ANXA4	annexin A4
1,17	ENSG00000143412	ANXA9	annexin A9
2,077	ENSG00000213983	AP1G2	adaptor related protein complex 1 gamma 2 subunit
1,969	ENSG00000129354	AP1M2	adaptor related protein complex 1 mu 2 subunit
1,318	ENSG00000154856	APCDD1	APC down-regulated 1
1,61	ENSG00000105290	APLP1	amyloid beta precursor like protein 1
2,663	ENSG00000179750	APOBEC3B	apolipoprotein B mRNA editing enzyme catalytic subunit 3B
1,653	ENSG00000047365	ARAP2	ArfGAP with RhoGAP domain, ankyrin repeat and PH domain 2
4,291	ENSG00000198576	ARC	activity regulated cytoskeleton associated protein
3,318	ENSG00000109321	AREG	amphiregulin
1,344	ENSG00000134884	ARGLU1	arginine and glutamate rich 1
1,283	ENSG00000138639	ARHGAP24	Rho GTPase activating protein 24
1,263	ENSG00000145819	ARHGAP26	Rho GTPase activating protein 26
2,026	ENSG00000248405	ARHGAP8/PRR5-ARHGAP8	Rho GTPase activating protein 8
1,565	ENSG00000130762	ARHGEF16	Rho guanine nucleotide exchange factor 16
1,058	ENSG00000142632	ARHGEF19	Rho guanine nucleotide exchange factor 19
1,454	ENSG00000236699	ARHGEF38	Rho guanine nucleotide exchange factor 38

1,406	ENSG00000165801	ARHGEF40	Rho guanine nucleotide exchange factor 40
1,171	ENSG00000050327	ARHGEF5	Rho guanine nucleotide exchange factor 5
1,588	ENSG00000150347	ARID5B	AT-rich interaction domain 5B
3,468	ENSG00000179674	ARL14	ADP ribosylation factor like GTPase 14
1,124	ENSG00000122644	ARL4A	ADP ribosylation factor like GTPase 4A
1,746	ENSG00000188042	ARL4C	ADP ribosylation factor like GTPase 4C
1,329	ENSG00000196503	ARL9	ADP ribosylation factor like GTPase 9
1,654	ENSG00000135931	ARMC9	armadillo repeat containing 9
1,8	ENSG00000172379	ARNT2	aryl hydrocarbon receptor nuclear translocator 2
1,146	ENSG00000029153	ARNTL2	aryl hydrocarbon receptor nuclear translocator like 2
1,388	ENSG00000250151	ARPC4-TTLL3	ARPC4-TTLL3 readthrough
1,304	ENSG00000105643	ARRDC2	arrestin domain containing 2
1,719	ENSG00000180801	ARSJ	arylsulfatase family member J
1,337	ENSG00000065802	ASB1	ankyrin repeat and SOCS box containing 1
1,073	ENSG00000116539	ASH1L	ASH1 like histone lysine methyltransferase
1,023	ENSG00000070669	ASNS	asparagine synthetase (glutamine-hydrolyzing)
1,832	ENSG00000174939	ASPHD1	aspartate beta-hydroxylase domain containing 1
1,572	ENSG00000148219	ASTN2	astrotactin 2
4,237	ENSG00000162772	ATF3	activating transcription factor 3
1,063	ENSG00000068650	ATP11A	ATPase phospholipid transporting 11A
1,325	ENSG00000127249	ATP13A4	ATPase 13A4
1,789	ENSG00000163399	ATP1A1	ATPase Na+/K+ transporting subunit alpha 1
1,356	ENSG00000203865	ATP1A1-AS1	ATP1A1 antisense RNA 1
1,914	ENSG00000248919	ATP5J2-PTCD1	ATP5J2-PTCD1 readthrough
1,256	ENSG00000054793	ATP9A	ATPase phospholipid transporting 9A (putative)
1,227	ENSG00000204842	ATXN2	ataxin 2
1,267	ENSG00000168488	ATXN2L	ataxin 2 like
1,377	ENSG00000184809	B3GALT5-AS1	B3GALT5 antisense RNA 1
1,025	ENSG00000179913	B3GNT3	UDP-GlcNAc:betaGal beta-1,3-N-acetylglucosaminyltransferase 3
3,022	ENSG00000176597	B3GNT5	UDP-GlcNAc:betaGal beta-1,3-N-acetylglucosaminyltransferase 5
1,236	ENSG00000156966	B3GNT7	UDP-GlcNAc:betaGal beta-1,3-N-acetylglucosaminyltransferase 7
1,677	ENSG00000139044	B4GALNT3	beta-1,4-N-acetyl-galactosaminyltransferase 3
1,177	ENSG00000158470	B4GALT5	beta-1,4-galactosyltransferase 5
1,355	ENSG00000182240	BACE2	beta-site APP-cleaving enzyme 2
5,482	ENSG00000151929	BAG3	BCL2 associated athanogene 3
1,245	ENSG00000006453	BAIAP2L1	BAI1 associated protein 2 like 1
1,253	ENSG00000128298	BAIAP2L2	BAI1 associated protein 2 like 2
2,181	ENSG00000095739	BAMBI	BMP and activin membrane bound inhibitor
1,456	ENSG00000138376	BARD1	BRCA1 associated RING domain 1
1,273	ENSG00000043039	BARX2	BARX homeobox 2
1,716	ENSG00000198604	BAZ1A	bromodomain adjacent to zinc finger domain 1A
1,084	ENSG00000076108	BAZ2A	bromodomain adjacent to zinc finger domain 2A
1,101	ENSG00000123636	BAZ2B	bromodomain adjacent to zinc finger domain 2B
3,366	ENSG00000105327	BBC3	BCL2 binding component 3
1,531	ENSG00000050820	BCAR1	BCAR1, Cas family scaffolding protein
1,629	ENSG00000142867	BCL10	B-cell CLL/lymphoma 10
2,18	ENSG00000171791	BCL2	BCL2, apoptosis regulator
1,09	ENSG00000171552	BCL2L1	BCL2 like 1
1,328	ENSG00000153094	BCL2L11	BCL2 like 11
1,588	ENSG00000110987	BCL7A	BCL tumor suppressor 7A
1,303	ENSG00000236824	BCYRN1	brain cytoplasmic RNA 1
1,005	ENSG00000165626	BEND7	BEN domain containing 7
2,082	ENSG00000133169	BEX1	brain expressed X-linked 1
1,334	ENSG00000133134	BEX2	brain expressed X-linked 2
1,259	ENSG00000102409	BEX4	brain expressed X-linked 4
2,7	ENSG00000123095	BHLHE41	basic helix-loop-helix family member e41
1,796	ENSG00000122870	BICC1	BicC family RNA binding protein 1
2,292	ENSG00000162069	BICDL2	BICD family like cargo adaptor 2
2,871	ENSG00000100290	BIK	BCL2 interacting killer
2,942	ENSG00000023445	BIRC3	baculoviral IAP repeat containing 3
2,786	ENSG00000125845	BMP2	bone morphogenetic protein 2
1,121	ENSG00000204177	BMS1P5	BMS1, ribosome biogenesis factor pseudogene 5
1,223	ENSG00000157764	BRAF	B-Raf proto-oncogene, serine/threonine kinase
1,084	ENSG00000100425	BRD1	bromodomain containing 1
1,416	ENSG00000141867	BRD4	bromodomain containing 4
1,286	ENSG00000119411	BSPRY	B-box and SPRY domain containing
1,208	ENSG00000133639	BTG1	BTG anti-proliferation factor 1
2,808	ENSG00000159388	BTG2	BTG anti-proliferation factor 2
1,434	ENSG00000154640	BTG3	BTG anti-proliferation factor 3
1,04	ENSG00000176236	C10orf111	chromosome 10 open reading frame 111
1,109	ENSG00000149179	C11orf49	chromosome 11 open reading frame 49
1,601	ENSG00000109944	C11orf63	chromosome 11 open reading frame 63
1,103	ENSG00000205177	C11orf91	chromosome 11 open reading frame 91
1,638	ENSG00000235162	C12orf75	chromosome 12 open reading frame 75
1,051	ENSG00000100557	C14orf105	chromosome 14 open reading frame 105
1,33	ENSG00000140104	C14orf79	chromosome 14 open reading frame 79
1,048	ENSG00000186073	C15orf41	chromosome 15 open reading frame 41
2,305	ENSG00000188549	C15orf52	chromosome 15 open reading frame 52
1,434	ENSG00000167644	C19orf33	chromosome 19 open reading frame 33
1,041	ENSG00000105072	C19orf44	chromosome 19 open reading frame 44
2,491	ENSG00000163362	C1orf106	chromosome 1 open reading frame 106

2,18	ENSG00000182795	C1orf116	chromosome 1 open reading frame 116
1,35	ENSG00000119280	C1orf198	chromosome 1 open reading frame 198
1,155	ENSG00000221953	C1orf229	chromosome 1 open reading frame 229
1,646	ENSG00000131094	C1QL1	complement C1q like 1
1,969	ENSG00000205502	C2CD4B	C2 calcium dependent domain containing 4B
1,189	ENSG00000183186	C2CD4C	C2 calcium dependent domain containing 4C
1,235	ENSG00000225556	C2CD4D	C2 calcium dependent domain containing 4D
3,777	ENSG00000114529	C3orf52	chromosome 3 open reading frame 52
1,17	ENSG00000181744	C3orf58	chromosome 3 open reading frame 58
1,032	ENSG00000205129	C4orf47	chromosome 4 open reading frame 47
1,03	ENSG00000197603	C5orf42	chromosome 5 open reading frame 42
1,138	ENSG00000178776	C5orf46	chromosome 5 open reading frame 46
1,054	ENSG00000188112	C6orf132	chromosome 6 open reading frame 132
3,28	ENSG00000197261	C6orf141	chromosome 6 open reading frame 141
1,607	ENSG00000189325	C6orf222	chromosome 6 open reading frame 222
1,243	ENSG00000137434	C6orf52	chromosome 6 open reading frame 52
1,623	ENSG00000176907	C8orf4	chromosome 8 open reading frame 4
1,714	ENSG00000160345	C9orf116	chromosome 9 open reading frame 116
1,306	ENSG00000204352	C9orf129	chromosome 9 open reading frame 129
2,169	ENSG00000164879	CA3	carbonic anhydrase 3
2,058	ENSG00000107159	CA9	carbonic anhydrase 9
1,47	ENSG00000116161	CACYBP	calcyclin binding protein
1,096	ENSG00000084774	CAD	carbamoyl-phosphate synthetase 2, aspartate transcarbamylase, and dihydroorotase
1,143	ENSG00000270419	CAHM	colon adenocarcinoma hypermethylated (non-protein coding)
1,504	ENSG00000130559	CAMSAP1	calmodulin regulated spectrin associated protein 1
1,258	ENSG00000076826	CAMSAP3	calmodulin regulated spectrin associated protein family member 3
1,167	ENSG00000108509	CAMTA2	calmodulin binding transcription activator 2
1,119	ENSG00000042493	CAPG	capping actin protein, gelsolin like
1,333	ENSG00000182472	CAPN12	calpain 12
1,114	ENSG00000162909	CAPN2	calpain 2
1,383	ENSG00000077274	CAPN6	calpain 6
1,669	ENSG00000105519	CAPS	calcyphosine
1,097	ENSG00000100065	CARD10	caspase recruitment domain family member 10
1,497	ENSG00000036828	CASR	calcium sensing receptor
2,287	ENSG00000130940	CASZ1	castor zinc finger 1
1,025	ENSG00000078699	CBFA2T2	CBFA2/RUNX1 translocation partner 2
1,631	ENSG00000141582	CBX4	chromobox 4
1,532	ENSG00000183741	CBX6	chromobox 6
1,04	ENSG00000158941	CCAR2	cell cycle and apoptosis regulator 2
1,226	ENSG00000135736	CCDC102A	coiled-coil domain containing 102A
1,377	ENSG00000167131	CCDC103	coiled-coil domain containing 103
1,38	ENSG00000151773	CCDC122	coiled-coil domain containing 122
1,171	ENSG00000175455	CCDC14	coiled-coil domain containing 14
1,84	ENSG00000163492	CCDC141	coiled-coil domain containing 141
1,126	ENSG00000153237	CCDC148	coiled-coil domain containing 148
1,05	ENSG00000165813	CCDC186	coiled-coil domain containing 186
1,547	ENSG00000196118	CCDC189	coiled-coil domain containing 189
1,99	ENSG00000159214	CCDC24	coiled-coil domain containing 24
1,329	ENSG00000160050	CCDC28B	coiled-coil domain containing 28B
1,595	ENSG00000152076	CCDC74B	coiled-coil domain containing 74B
1,122	ENSG00000105321	CCDC9	coiled-coil domain containing 9
2,586	ENSG00000108691	CCL2	C-C motif chemokine ligand 2
3,477	ENSG00000115009	CCL20	C-C motif chemokine ligand 20
1,446	ENSG00000151882	CCL28	C-C motif chemokine ligand 28
2,01	ENSG00000163660	CCNL1	cyclin L1
1,23	ENSG00000221978	CCNL2	cyclin L2
2,679	ENSG00000126353	CCR7	C-C motif chemokine receptor 7
1,124	ENSG00000117877	CD3EAP	CD3e molecule associated protein
2,615	ENSG00000112149	CD83	CD83 molecule
1,237	ENSG00000198752	CDC42BPB	CDC42 binding protein kinase beta
1,042	ENSG00000171219	CDC42BPG	CDC42 binding protein kinase gamma
1,138	ENSG00000128283	CDC42EP1	CDC42 effector protein 1
1,297	ENSG00000179604	CDC42EP4	CDC42 effector protein 4
2,241	ENSG00000163814	CDCP1	CUB domain containing protein 1
2,025	ENSG00000039068	CDH1	cadherin 1
2,265	ENSG00000113361	CDH6	cadherin 6
1,073	ENSG00000008128	CDK11A	cyclin dependent kinase 11A
1,243	ENSG00000156345	CDK20	cyclin dependent kinase 20
1,639	ENSG00000124762	CDKN1A	cyclin dependent kinase inhibitor 1A
1,556	ENSG00000129757	CDKN1C	cyclin dependent kinase inhibitor 1C
1,221	ENSG00000168564	CDKN2AIP	CDKN2A interacting protein
2,418	ENSG00000147883	CDKN2B	cyclin dependent kinase inhibitor 2B
1,001	ENSG00000109089	CDR2L	cerebellar degeneration related protein 2 like
1,655	ENSG00000163624	CDS1	CDP-diacylglycerol synthase 1
2,041	ENSG00000086548	CEACAM6	carcinoembryonic antigen related cell adhesion molecule 6
1,292	ENSG00000102901	CENPT	centromere protein T
1,28	ENSG00000141577	CEP131	centrosomal protein 131
1,026	ENSG00000121289	CEP89	centrosomal protein 89
2,026	ENSG00000163075	CFAP221	cilia and flagella associated protein 221
1,451	ENSG00000226312	CFLAR-AS1	CFLAR antisense RNA 1
2,852	ENSG00000001626	CFTR	cystic fibrosis transmembrane conductance regulator

1,256	ENSG00000143375	CGN	cingulin
1,051	ENSG00000159259	CHAF1B	chromatin assembly factor 1 subunit B
1,079	ENSG00000153922	CHD1	chromodomain helicase DNA binding protein 1
1,951	ENSG00000173575	CHD2	chromodomain helicase DNA binding protein 2
1,472	ENSG00000170004	CHD3	chromodomain helicase DNA binding protein 3
1,883	ENSG00000110721	CHKA	choline kinase alpha
1,207	ENSG00000203668	CHML	CHM like, Rab escort protein 2
1,447	ENSG00000101421	CHMP4B	charged multivesicular body protein 4B
1,867	ENSG00000110172	CHORDC1	cysteine and histidine rich domain containing 1
1,296	ENSG00000133019	CHRM3	cholinergic receptor muscarinic 3
1,101	ENSG00000122863	CHST3	carbohydrate sulfotransferase 3
1,372	ENSG00000140835	CHST4	carbohydrate sulfotransferase 4
1,066	ENSG00000127586	CHTF18	chromosome transmission fidelity factor 18
1,381	ENSG00000179862	CITED4	Cbp/p300 interacting transactivator with Glu/Asp rich carboxy-terminal domain 4
1,1	ENSG00000166165	CKB	creatine kinase B
1,044	ENSG00000223572	CKMT1A/CKMT1B	creatine kinase, mitochondrial 1B
1,875	ENSG00000131730	CKMT2	creatine kinase, mitochondrial 2
1,145	ENSG00000104859	CLASRP	CLK4 associating serine/arginine rich protein
3,396	ENSG00000175505	CLCF1	cardiotrophin like cytokine factor 1
1,115	ENSG00000114859	CLCN2	chloride voltage-gated channel 2
1,024	ENSG00000011021	CLCN6	chloride voltage-gated channel 6
1,827	ENSG00000163347	CLDN1	claudin 1
1,929	ENSG00000134873	CLDN10	claudin 10
1,183	ENSG00000253958	CLDN23	claudin 23
1,207	ENSG00000184697	CLDN6	claudin 6
1,154	ENSG00000181885	CLDN7	claudin 7
2,465	ENSG00000213937	CLDN9	claudin 9
1,007	ENSG00000038532	CLEC16A	C-type lectin domain containing 16A
1,945	ENSG00000159212	CLIC6	chloride intracellular channel 6
1,047	ENSG00000113282	CLINT1	clathrin interactor 1
2,27	ENSG00000013441	CLK1	CDC like kinase 1
1,543	ENSG00000153551	CMTM7	CKLF like MARVEL transmembrane domain containing 7
2,203	ENSG00000105427	CNFN	cornifelin
1,624	ENSG00000142675	CNKSR1	connector enhancer of kinase suppressor of Ras 1
1,593	ENSG00000158158	CNNM4	cyclin and CBS domain divalent metal cation transport mediator 4
1,058	ENSG00000080802	CNOT4	CCR4-NOT transcription complex subunit 4
1,246	ENSG00000170037	CNTROB	centrobin, centriole duplication and spindle assembly protein
1,339	ENSG00000106078	COBL	cordon-bleu WH2 repeat protein
1,819	ENSG00000196739	COL27A1	collagen type XXVII alpha 1 chain
1,473	ENSG00000134871	COL4A2	collagen type IV alpha 2 chain
2,175	ENSG00000049089	COL9A2	collagen type IX alpha 2 chain
1,53	ENSG00000214290	COLCA2	colorectal cancer associated 2
1,108	ENSG00000135678	CPM	carboxypeptidase M
2,099	ENSG00000205560	CPT1B	carnitine palmitoyltransferase 1B
1,817	ENSG00000177685	CRACR2B	calcium release activated channel regulator 2B
3,085	ENSG00000146592	CREB5	cAMP responsive element binding protein 5
1,16	ENSG00000005339	CREBBP	CREB binding protein
1,095	ENSG00000058453	CROCC	ciliary rootlet coiled-coil, rootletin
1,367	ENSG00000095713	CRTAC1	cartilage acidic protein 1
1,397	ENSG00000008405	CRY1	cryptochrome circadian clock 1
1,46	ENSG00000184371	CSF1	colony stimulating factor 1
2,293	ENSG00000108342	CSF3	colony stimulating factor 3
1,277	ENSG00000104218	CSPP1	centrosome and spindle pole associated protein 1
3,026	ENSG00000144655	CSRNP1	cysteine and serine rich nuclear protein 1
1,647	ENSG00000170373	CST1	cystatin SN
2,02	ENSG00000118523	CTGF	connective tissue growth factor
1,01	ENSG00000044115	CTNNA1	catenin alpha 1
1,318	ENSG00000198561	CTNND1	catenin delta 1
2,439	ENSG00000169862	CTNND2	catenin delta 2
1,375	ENSG00000077063	CTTNBP2	cortactin binding protein 2
1,024	ENSG00000178531	CTXN1	cortexin 1
1,692	ENSG00000180891	CUEDC1	CUE domain containing 1
1,4	ENSG00000044090	CUL7	cullin 7
2,101	ENSG00000006210	CX3CL1	C-X3-C motif chemokine ligand 1
4,043	ENSG00000163739	CXCL1	C-X-C motif chemokine ligand 1
3,846	ENSG00000081041	CXCL2	C-X-C motif chemokine ligand 2
4,81	ENSG00000163734	CXCL3	C-X-C motif chemokine ligand 3
2,604	ENSG00000163735	CXCL5	C-X-C motif chemokine ligand 5
2,487	ENSG00000124875	CXCL6	C-X-C motif chemokine ligand 6
3,5	ENSG00000169429	CXCL8	C-X-C motif chemokine ligand 8
1,073	ENSG00000008283	CYB561	cytochrome b561
1,245	ENSG00000235700	CYCSP52	cytochrome c, somatic pseudogene 52
3,026	ENSG00000142871	CYR61	cysteine rich angiogenic inducer 61
1,342	ENSG00000205795	CYS1	cystin 1
1,521	ENSG00000105443	CYTH2	cytohesin 2
1,24	ENSG00000222041	CYTOR	cytoskeleton regulator RNA
1,14	ENSG00000134780	DAGLA	diacylglycerol lipase alpha
1,404	ENSG00000178149	DALRD3	DALR anticodon binding domain containing 3
1,385	ENSG00000196730	DAPK1	death associated protein kinase 1
1,802	ENSG00000167657	DAPK3	death associated protein kinase 3
1,753	ENSG00000113758	DBN1	drebrin 1

1,086	ENSG00000003249	DBNDD1	dysbindin domain containing 1
2,168	ENSG00000146038	DCDC2	doublecortin domain containing 2
1,011	ENSG00000215301	DDX3X	DEAD-box helicase 3, X-linked
1,122	ENSG00000185163	DDX51	DEAD-box helicase 51
2,309	ENSG00000160570	DEDD2	death effector domain containing 2
2,015	ENSG00000203970	DEFB110	defensin beta 110
1,324	ENSG00000168350	DEGS2	delta 4-desaturase, sphingolipid 2
1,098	ENSG00000035499	DEPDC1B	DEP domain containing 1B
1,059	ENSG00000109606	DHX15	DEAH-box helicase 15
1,153	ENSG00000134815	DHX34	DExH-box helicase 34
1,275	ENSG00000135829	DHX9	DExH-box helicase 9
1,02	ENSG00000101191	DIDO1	death inducer-obliterator 1
1,487	ENSG00000165023	DIRAS2	DIRAS family GTPase 2
1,059	ENSG00000144535	DIS3L2	DIS3 like 3'-5' exoribonuclease 2
1,072	ENSG00000151208	DLG5	discs large MAGUK scaffold protein 5
1,024	ENSG00000170579	DLGAP1	DLG associated protein 1
1,112	ENSG00000100206	DMC1	DNA meiotic recombinase 1
1,269	ENSG00000161249	DMKN	dermokine
1,045	ENSG00000185800	DMWD	dystrophia myotonica, WD repeat containing
1,503	ENSG00000256061	DNAAF4	dynein axonemal assembly factor 4
2,467	ENSG00000086061	DNAJA1	DnaJ heat shock protein family (Hsp40) member A1
3,069	ENSG00000140403	DNAJA4	DnaJ heat shock protein family (Hsp40) member A4
5,728	ENSG00000132002	DNAJB1	DnaJ heat shock protein family (Hsp40) member B1
4,088	ENSG00000162616	DNAJB4	DnaJ heat shock protein family (Hsp40) member B4
1,807	ENSG00000244115	DNAJC25-GNG10	DNAJC25-GNG10 readthrough
1,032	ENSG00000106976	DNM1	dynamin 1
1,133	ENSG00000107554	DNMBP	dynamin binding protein
1,241	ENSG00000147459	DOCK5	dedicator of cytokinesis 5
1,011	ENSG00000129932	DOHH	deoxyhypusine hydroxylase
1,187	ENSG00000175920	DOK7	docking protein 7
1,675	ENSG00000104885	DOT1L	DOT1 like histone lysine methyltransferase
1,061	ENSG00000166171	DPCD	deleted in primary ciliary dyskinesia homolog (mouse)
1,173	ENSG00000171962	DRC3	dynein regulatory complex subunit 3
1,658	ENSG00000134755	DSC2	desmocollin 2
1,084	ENSG00000134762	DSC3	desmocollin 3
1,786	ENSG00000046604	DSG2	desmoglein 2
1,873	ENSG00000096696	DSP	desmoplakin
1,209	ENSG00000091073	DTX2	deltex E3 ubiquitin ligase 2
1,286	ENSG00000178498	DTX3	deltex E3 ubiquitin ligase 3
1,288	ENSG00000140279	DUOX2	dual oxidase 2
1,813	ENSG00000140274	DUOXA2	dual oxidase maturation factor 2
2,567	ENSG00000120129	DUSP1	dual specificity phosphatase 1
1,721	ENSG00000143507	DUSP10	dual specificity phosphatase 10
1,058	ENSG00000111266	DUSP16	dual specificity phosphatase 16
1,175	ENSG00000167065	DUSP18	dual specificity phosphatase 18
2,849	ENSG00000158050	DUSP2	dual specificity phosphatase 2
2,449	ENSG00000120875	DUSP4	dual specificity phosphatase 4
2,574	ENSG00000138166	DUSP5	dual specificity phosphatase 5
2,878	ENSG00000184545	DUSP8	dual specificity phosphatase 8
1,185	ENSG00000197102	DYNC1H1	dynein cytoplasmic 1 heavy chain 1
1,381	ENSG00000134874	DZIP1	DAZ interacting zinc finger protein 1
1,881	ENSG00000158163	DZIP1L	DAZ interacting zinc finger protein 1 like
1,056	ENSG00000088881	EBF4	early B-cell factor 4
1,388	ENSG00000038358	EDC4	enhancer of mRNA decapping 4
4,159	ENSG00000078401	EDN1	endothelin 1
2,536	ENSG00000127129	EDN2	endothelin 2
1,253	ENSG00000101210	EEF1A2	eukaryotic translation elongation factor 1 alpha 2
1,549	ENSG00000122547	EEPD1	endonuclease/exonuclease/phosphatase family domain containing 1
1,129	ENSG00000096093	EFHC1	EF-hand domain containing 1
1,112	ENSG00000090776	EFNB1	ephrin B1
1,2	ENSG00000146648	EGFR	epidermal growth factor receptor
3,348	ENSG00000235947	EGOT	eosinophil granule ontogeny transcript (non-protein coding)
3,87	ENSG00000120738	EGR1	early growth response 1
2,148	ENSG00000179388	EGR3	early growth response 3
1,344	ENSG00000135625	EGR4	early growth response 4
1,755	ENSG00000110047	EHD1	EH domain containing 1
1,2	ENSG00000103966	EHD4	EH domain containing 4
2,526	ENSG00000135373	EHF	ETS homologous factor
1,015	ENSG00000172071	EIF2AK3	eukaryotic translation initiation factor 2 alpha kinase 3
1,001	ENSG00000141543	EIF4A3	eukaryotic translation initiation factor 4A3
2,156	ENSG00000163435	ELF3	E74 like ETS transcription factor 3
1,165	ENSG00000102890	ELMO3	engulfment and cell motility 3
1,637	ENSG00000164181	ELOVL7	ELOVL fatty acid elongase 7
1,303	ENSG00000134531	EMP1	epithelial membrane protein 1
1,41	ENSG00000154380	ENAH	enabled homolog (Drosophila)
1,493	ENSG00000171617	ENC1	ectodermal-neural cortex 1
1,044	ENSG00000167280	ENGASE	endo-beta-N-acetylglucosaminidase
1,293	ENSG00000100393	EP300	E1A binding protein p300
1,059	ENSG00000088367	EPB41L1	erythrocyte membrane protein band 4.1 like 1
2,514	ENSG00000119888	EPCAM	epithelial cell adhesion molecule
1,077	ENSG00000183317	EPHA10	EPH receptor A10

2,966	ENSG00000142627	EPHA2	EPH receptor A2
1,11	ENSG00000177106	EPS8L2	EPS8 like 2
1,681	ENSG00000198758	EPS8L3	EPS8 like 3
3,051	ENSG00000124882	EREG	epiregulin
1,277	ENSG00000177459	ERICH5	glutamate rich 5
1,054	ENSG00000134398	ERN2	endoplasmic reticulum to nucleus signaling 2
1,615	ENSG00000116285	ERRFI1	ERBB receptor feedback inhibitor 1
1,622	ENSG00000213462	ERV3-1	endogenous retrovirus group 3 member 1, envelope
1,887	ENSG00000104413	ESRP1	epithelial splicing regulatory protein 1
1,06	ENSG00000196482	ESRRG	estrogen related receptor gamma
1,799	ENSG00000117036	ETV3	ETS variant 3
2,315	ENSG00000175832	ETV4	ETS variant 4
1,483	ENSG00000072840	EVC	EvC ciliary complex subunit 1
1,374	ENSG00000173040	EVC2	EvC ciliary complex subunit 2
1,167	ENSG00000187609	EXD3	exonuclease 3'-5' domain containing 3
1,098	ENSG00000144036	EXOC6B	exocyst complex component 6B
2,38	ENSG00000092820	EZR	ezrin
1,697	ENSG00000164251	F2RL1	F2R like trypsin receptor 1
1,555	ENSG00000117525	F3	coagulation factor III, tissue factor
2,271	ENSG00000103089	FA2H	fatty acid 2-hydroxylase
1,12	ENSG00000221968	FADS3	fatty acid desaturase 3
1,902	ENSG00000167106	FAM102A	family with sequence similarity 102 member A
1,159	ENSG00000184731	FAM110C	family with sequence similarity 110 member C
1,204	ENSG00000138439	FAM117B	family with sequence similarity 117 member B
1,579	ENSG00000164142	FAM160A1	family with sequence similarity 160 member A1
1,251	ENSG00000188163	FAM166A	family with sequence similarity 166 member A
1,569	ENSG00000148468	FAM171A1	family with sequence similarity 171 member A1
1,949	ENSG00000146067	FAM193B	family with sequence similarity 193 member B
1,076	ENSG00000204860	FAM201A	family with sequence similarity 201 member A
1,106	ENSG00000183844	FAM3B	family with sequence similarity 3 member B
1	ENSG00000198643	FAM3D	family with sequence similarity 3 member D
2,927	ENSG00000112773	FAM46A	family with sequence similarity 46 member A
2,298	ENSG00000158246	FAM46B	family with sequence similarity 46 member B
1,244	ENSG00000183508	FAM46C	family with sequence similarity 46 member C
2,124	ENSG00000120709	FAM53C	family with sequence similarity 53 member C
1,301	ENSG00000139146	FAM60A	family with sequence similarity 60 member A
1,418	ENSG00000168143	FAM83B	family with sequence similarity 83 member B
1,873	ENSG00000105523	FAM83E	family with sequence similarity 83 member E
2,746	ENSG00000188522	FAM83G	family with sequence similarity 83 member G
1,333	ENSG00000152767	FARP1	FERM, ARH/RhoGEF and pleckstrin domain protein 1
1,149	ENSG00000162458	FBLIM1	filamin binding LIM protein 1
1,146	ENSG00000156860	FBRS	fibrosin
2,239	ENSG00000171823	FBXL14	F-box and leucine rich repeat protein 14
1,054	ENSG00000130475	FCHO1	FCH domain only 1
1,062	ENSG00000197948	FCHSD1	FCH and double SH3 domains 1
1,602	ENSG00000145780	FEM1C	fem-1 homolog C
1,281	ENSG00000180263	FGD6	FYVE, RhoGEF and PH domain containing 6
1,386	ENSG00000162344	FGF19	fibroblast growth factor 19
1,029	ENSG00000138675	FGF5	fibroblast growth factor 5
1,406	ENSG00000066468	FGFR2	fibroblast growth factor receptor 2
2,122	ENSG00000068078	FGFR3	fibroblast growth factor receptor 3
1,001	ENSG00000160867	FGFR4	fibroblast growth factor receptor 4
2,356	ENSG00000168386	FILIP1L	filamin A interacting protein 1 like
1,963	ENSG00000004478	FKBP4	FK506 binding protein 4
1,845	ENSG00000136068	FLNB	filamin B
1,262	ENSG00000185070	FLRT2	fibronectin leucine rich transmembrane protein 2
2,014	ENSG00000125848	FLRT3	fibronectin leucine rich transmembrane protein 3
1,63	ENSG00000137942	FNBP1L	formin binding protein 1 like
1,392	ENSG00000109920	FNBP4	formin binding protein 4
1,507	ENSG00000110195	FOLR1	folate receptor 1
4,008	ENSG00000170345	FOS	Fos proto-oncogene, AP-1 transcription factor subunit
6,003	ENSG00000125740	FOSB	FosB proto-oncogene, AP-1 transcription factor subunit
4,17	ENSG00000175592	FOSL1	FOS like 1, AP-1 transcription factor subunit
1,985	ENSG00000075426	FOSL2	FOS like 2, AP-1 transcription factor subunit
1,404	ENSG00000129514	FOXA1	forkhead box A1
1,374	ENSG00000125798	FOXA2	forkhead box A2
1,98	ENSG00000129654	FOXJ1	forkhead box J1
1,345	ENSG00000150907	FOXO1	forkhead box O1
1,122	ENSG00000118689	FOXO3	forkhead box O3
1,172	ENSG00000114861	FOXP1	forkhead box P1
1,265	ENSG00000128573	FOXP2	forkhead box P2
2,905	ENSG00000164379	FOXQ1	forkhead box Q1
1,389	ENSG00000138759	FRAS1	Fraser extracellular matrix complex subunit 1
1,247	ENSG00000181274	FRAT2	FRAT2, WNT signaling pathway regulator
1,354	ENSG00000114541	FRMD4B	FERM domain containing 4B
1,252	ENSG00000106701	FSD1L	fibronectin type III and SPRY domain containing 1 like
2,087	ENSG00000070404	FSTL3	follistatin like 3
1,155	ENSG00000089280	FUS	FUS RNA binding protein
1,21	ENSG00000171124	FUT3	fucosyltransferase 3 (Lewis blood group)
1,745	ENSG00000196371	FUT4	fucosyltransferase 4
1,816	ENSG00000137731	FXYD2	FXYD domain containing ion transport regulator 2

1,284	ENSG00000089356	FXYD3	FXYD domain containing ion transport regulator 3
1,386	ENSG00000157240	FZD1	frizzled class receptor 1
1,499	ENSG00000102287	GABRE	gamma-aminobutyric acid type A receptor epsilon subunit
2,093	ENSG00000094755	GABRP	gamma-aminobutyric acid type A receptor pi subunit
1,487	ENSG00000116717	GADD45A	growth arrest and DNA damage inducible alpha
1,485	ENSG00000099860	GADD45B	growth arrest and DNA damage inducible beta
1,319	ENSG00000128242	GAL3ST1	galactose-3-O-sulfotransferase 1
1,678	ENSG00000119514	GALNT12	polypeptide N-acetylgalactosaminyltransferase 12
2,333	ENSG00000115339	GALNT3	polypeptide N-acetylgalactosaminyltransferase 3
1,486	ENSG00000257594	GALNT4	polypeptide N-acetylgalactosaminyltransferase 4
1,237	ENSG00000141448	GATA6	GATA binding protein 6
1,029	ENSG00000070610	GBA2	glucosylceramidase beta 2
1,055	ENSG00000107862	GBF1	golgi brefeldin A resistant guanine nucleotide exchange factor 1
1,096	ENSG00000117228	GBP1	guanylate binding protein 1
1,948	ENSG00000140297	GCNT3	glucosaminyl (N-acetyl) transferase 3, mucin type
2,12	ENSG00000130513	GDF15	growth differentiation factor 15
2,527	ENSG00000167741	GGT6	gamma-glutamyltransferase 6
1,195	ENSG00000106128	GHRHR	growth hormone releasing hormone receptor
1,085	ENSG00000146830	GIGYF1	GRB10 interacting GYF protein 1
1,62	ENSG00000188910	GJB3	gap junction protein beta 3
1,212	ENSG00000126603	GLIS2	GLIS family zinc finger 2
1,989	ENSG00000107249	GLIS3	GLIS family zinc finger 3
1,062	ENSG00000237009	GLIS3-AS1	GLIS3 antisense RNA 1
1,229	ENSG00000109738	GLRB	glycine receptor beta
1,203	ENSG00000120063	GNA13	G protein subunit alpha 13
1,978	ENSG00000215252	GOLGA8A/GOLGA8B	golgin A8 family member A
1,239	ENSG00000116580	GON4L	gon-4 like
1,037	ENSG00000186566	GPATCH8	G-patch domain containing 8
1,061	ENSG00000062194	GPBP1	GC-rich promoter binding protein 1
1,78	ENSG00000166073	GPR176	G protein-coupled receptor 176
1,265	ENSG00000169508	GPR183	G protein-coupled receptor 183
2,926	ENSG00000013588	GPRC5A	G protein-coupled receptor class C group 5 member A
2,376	ENSG00000167191	GPRC5B	G protein-coupled receptor class C group 5 member B
1,752	ENSG00000023171	GRAMD1B	GRAM domain containing 1B
2,575	ENSG00000141738	GRB7	growth factor receptor bound protein 7
1,062	ENSG00000196208	GREB1	growth regulation by estrogen in breast cancer 1
1,968	ENSG00000083307	GRHL2	grainyhead like transcription factor 2
1,03	ENSG00000084207	GSTP1	glutathione S-transferase pi 1
1,767	ENSG00000100226	GTPBP1	GTP binding protein 1
1,673	ENSG00000197273	GUCA2A	guanylate cyclase activator 2A
1,105	ENSG00000144366	GULP1	GULP, engulfment adaptor PTB domain containing 1
1,954	ENSG00000228315	GUSBP11	glucuronidase, beta pseudogene 11
1,068	ENSG00000241549	GUSBP2	glucuronidase, beta pseudogene 2
1,89	ENSG00000253203	GUSBP3	glucuronidase, beta pseudogene 3
1,204	ENSG00000184897	H1FX	H1 histone family member X
1,367	ENSG00000188486	H2AFX	H2A histone family member X
1,117	ENSG00000132475	H3F3A/H3F3B	H3 histone family member 3A
1,158	ENSG00000188375	H3F3C	H3 histone family member 3C
1,298	ENSG00000103044	HAS3	hyaluronan synthase 3
3,814	ENSG00000113070	HBEGF	heparin binding EGF like growth factor
1,084	ENSG00000048052	HDAC9	histone deacetylase 9
1,094	ENSG00000167674	HDGFL2	HDGF like 2
2,702	ENSG00000230267	HERC2P4	hect domain and RLD 2 pseudogene 4
1,005	ENSG00000114315	HES1	hes family bHLH transcription factor 1
1,902	ENSG00000188290	HES4	hes family bHLH transcription factor 4
1,454	ENSG00000186834	HEXIM1	hexamethylene bisacetamide inducible 1
1,139	ENSG00000100644	HIF1A	hypoxia inducible factor 1 alpha subunit
1,902	ENSG00000130787	HIP1R	huntingtin interacting protein 1 related
2,193	ENSG00000196866	HIST1H2AD	histone cluster 1 H2A family member d
1,409	ENSG00000124635	HIST1H2BJ	histone cluster 1 H2B family member j
2,318	ENSG00000233822	HIST1H2BN	histone cluster 1 H2B family member n
1,994	ENSG00000197409	HIST1H3D	histone cluster 1 H3 family member d
1,251	ENSG00000197153	HIST1H3J	histone cluster 1 H3 family member j
1,603	ENSG00000196890	HIST3H2BB	histone cluster 3 H2B family member b
1,456	ENSG00000197837	HIST4H4	histone cluster 4 H4
1,026	ENSG00000095951	HIVEP1	human immunodeficiency virus type I enhancer binding protein 1
1,831	ENSG00000010818	HIVEP2	human immunodeficiency virus type I enhancer binding protein 2
1,701	ENSG00000156510	HKDC1	hexokinase domain containing 1
2,612	ENSG00000137309	HMGA1	high mobility group AT-hook 1
1,443	ENSG00000113161	HMGCR	3-hydroxy-3-methylglutaryl-CoA reductase
1,823	ENSG00000112972	HMGCS1	3-hydroxy-3-methylglutaryl-CoA synthase 1
1,063	ENSG00000113716	HMGXB3	HMG-box containing 3
1,195	ENSG00000177733	HNRNPA0	heterogeneous nuclear ribonucleoprotein A0
1,352	ENSG00000169045	HNRNPH1	heterogeneous nuclear ribonucleoprotein H1
1,039	ENSG00000096746	HNRNPH3	heterogeneous nuclear ribonucleoprotein H3
1,595	ENSG00000153187	HNRNPU	heterogeneous nuclear ribonucleoprotein U
1,125	ENSG00000103942	HOMER2	homer scaffolding protein 2
7,313	ENSG00000257017	HP	haptoglobin
1,146	ENSG00000127252	HRASLS	HRAS like suppressor
1,477	ENSG00000002587	HS3ST1	heparan sulfate-glucosamine 3-sulfotransferase 1
1,022	ENSG00000099251	HSD17B7P2	hydroxysteroid 17-beta dehydrogenase 7 pseudogene 2

1,627	ENSG00000102878	HSF4	heat shock transcription factor 4
2,99	ENSG00000080824	HSP90AA1	heat shock protein 90 alpha family class A member 1
2,668	ENSG00000096384	HSP90AB1	heat shock protein 90 alpha family class B member 1
2,059	ENSG00000205940	HSP90AB2P	heat shock protein 90 alpha family class B member 2, pseudogene
1,081	ENSG00000170606	HSPA4	heat shock protein family A (Hsp70) member 4
1,319	ENSG00000164070	HSPA4L	heat shock protein family A (Hsp70) member 4 like
1,29	ENSG00000044574	HSPA5	heat shock protein family A (Hsp70) member 5
6,995	ENSG00000173110	HSPA6	heat shock protein family A (Hsp70) member 6
6,679	ENSG00000225217	HSPA7	heat shock protein family A (Hsp70) member 7
2,462	ENSG00000109971	HSPA8	heat shock protein family A (Hsp70) member 8
1,387	ENSG00000144381	HSPD1	heat shock protein family D (Hsp60) member 1
5,132	ENSG00000120694	HSPH1	heat shock protein family H (Hsp110) member 1
2,069	ENSG00000142149	HUNK	hormonally up-regulated Neu-associated kinase
1,038	ENSG00000157423	HYDIN	HYDIN, axonemal central pair apparatus protein
1,283	ENSG00000163596	ICA1L	islet cell autoantigen 1 like
2,229	ENSG00000090339	ICAM1	intercellular adhesion molecule 1
1,02	ENSG00000105371	ICAM4	intercellular adhesion molecule 4 (Landsteiner-Wiener blood group)
1,034	ENSG00000172201	ID4	inhibitor of DNA binding 4, HLH protein
2,429	ENSG00000232656	IDI2-AS1	IDI2 antisense RNA 1
1,349	ENSG00000127415	IDUA	iduronidase, alpha-L-
2,343	ENSG00000160888	IER2	immediate early response 2
3,488	ENSG00000162783	IER5	immediate early response 5
1,004	ENSG00000188483	IER5L	immediate early response 5 like
1,91	ENSG00000169991	IFFO2	intermediate filament family orphan 2
1,823	ENSG00000006652	IFRD1	interferon related developmental regulator 1
1,545	ENSG00000187535	IFT140	intraflagellar transport 140
1,056	ENSG00000138002	IFT172	intraflagellar transport 172
1,22	ENSG00000122970	IFT81	intraflagellar transport 81
1,495	ENSG00000073792	IGF2BP2	insulin like growth factor 2 mRNA binding protein 2
1,709	ENSG00000143061	IGSF3	immunoglobulin superfamily member 3
1,416	ENSG00000150782	IL18	interleukin 18
2,389	ENSG00000125538	IL1B	interleukin 1 beta
3,182	ENSG00000110944	IL23A	interleukin 23 subunit alpha
1,057	ENSG00000077238	IL4R	interleukin 4 receptor
2,2	ENSG00000136244	IL6	interleukin 6
1,345	ENSG00000145103	ILDR1	immunoglobulin like domain containing receptor 1
1,73	ENSG00000129351	ILF3	interleukin enhancer binding factor 3
1,323	ENSG00000203485	INF2	inverted formin, FH2 and WH2 domain containing
2,122	ENSG00000122641	INHBA	inhibin beta A subunit
1,32	ENSG00000148384	INPP5E	inositol polyphosphate-5-phosphatase E
1,312	ENSG00000164066	INTU	inturned planar cell polarity protein
1,017	ENSG00000161896	IP6K3	inositol hexakisphosphate kinase 3
2,335	ENSG00000132321	IQCA1	IQ motif containing with AAA domain 1
1,416	ENSG00000174628	IQCK	IQ motif containing K
1,083	ENSG00000164675	IQUB	IQ motif and ubiquitin domain containing
1,926	ENSG00000134070	IRAK2	interleukin 1 receptor associated kinase 2
1,855	ENSG00000125347	IRF1	interferon regulatory factor 1
1,064	ENSG00000168264	IRF2BP2	interferon regulatory factor 2 binding protein 2
1,628	ENSG00000117595	IRF6	interferon regulatory factor 6
1,124	ENSG00000167378	IRGQ	immunity related GTPase Q
2,15	ENSG00000185950	IRS2	insulin receptor substrate 2
1,812	ENSG00000105655	ISYNA1	inositol-3-phosphate synthase 1
1,497	ENSG00000164171	ITGA2	integrin subunit alpha 2
2,222	ENSG00000005884	ITGA3	integrin subunit alpha 3
1,317	ENSG00000161638	ITGA5	integrin subunit alpha 5
1,024	ENSG00000091409	ITGA6	integrin subunit alpha 6
1,949	ENSG00000132470	ITGB4	integrin subunit beta 4
1,493	ENSG00000115221	ITGB6	integrin subunit beta 6
2,461	ENSG00000105855	ITGB8	integrin subunit beta 8
1,874	ENSG00000123243	ITIH5	inter-alpha-trypsin inhibitor heavy chain family member 5
2,98	ENSG00000086544	ITPKC	inositol-trisphosphate 3-kinase C
1,333	ENSG00000096433	ITPR3	inositol 1,4,5-trisphosphate receptor type 3
1,074	ENSG00000171988	JMJD1C	jumonji domain containing 1C
1,973	ENSG00000070495	JMJD6	arginine demethylase and lysine hydroxylase
3,82	ENSG00000177606	JUN	Jun proto-oncogene, AP-1 transcription factor subunit
1,896	ENSG00000171223	JUNB	JunB proto-oncogene, AP-1 transcription factor subunit
3,112	ENSG00000130522	JUND	JunD proto-oncogene, AP-1 transcription factor subunit
2,83	ENSG00000146049	KAAG1	kidney associated antigen 1
1,851	ENSG00000162975	KCNF1	potassium voltage-gated channel modifier subfamily F member 1
1,212	ENSG00000182132	KCNIP1	potassium voltage-gated channel interacting protein 1
1,057	ENSG00000115474	KCNJ13	potassium voltage-gated channel subfamily J member 13
1,286	ENSG00000157551	KCNJ15	potassium voltage-gated channel subfamily J member 15
1,521	ENSG00000153822	KCNJ16	potassium voltage-gated channel subfamily J member 16
1,088	ENSG00000082482	KCNK2	potassium two pore domain channel subfamily K member 2
1,802	ENSG00000164626	KCNK5	potassium two pore domain channel subfamily K member 5
1,2	ENSG00000213859	KCTD11	potassium channel tetramerization domain containing 11
1,112	ENSG00000174943	KCTD13	potassium channel tetramerization domain containing 13
1,493	ENSG00000173120	KDM2A	lysine demethylase 2A
1,005	ENSG00000107077	KDM4C	lysine demethylase 4C
1,902	ENSG00000117139	KDM5B	lysine demethylase 5B
1,031	ENSG00000012817	KDM5D	lysine demethylase 5D

1,519	ENSG00000147050	KDM6A	lysine demethylase 6A
1,917	ENSG00000132510	KDM6B	lysine demethylase 6B
1,133	ENSG00000047578	KIAA0556	KIAA0556
1,054	ENSG00000164542	KIAA0895	KIAA0895
1,696	ENSG00000196123	KIAA0895L	KIAA0895 like
1,034	ENSG00000120549	KIAA1217	KIAA1217
1,984	ENSG00000162522	KIAA1522	KIAA1522
2,766	ENSG00000136883	KIF12	kinesin family member 12
1,136	ENSG00000197892	KIF13B	kinesin family member 13B
1,032	ENSG00000131437	KIF3A	kinesin family member 3A
1,571	ENSG00000167702	KIFC2	kinesin family member C2
1,041	ENSG00000151657	KIN	Kin17 DNA and RNA binding protein
1,051	ENSG00000174996	KLC2	kinesin light chain 2
1,584	ENSG00000104892	KLC3	kinesin light chain 3
1,818	ENSG00000155090	KLF10	Kruppel like factor 10
2,544	ENSG00000127528	KLF2	Kruppel like factor 2
2,324	ENSG00000136826	KLF4	Kruppel like factor 4
3,599	ENSG00000102554	KLF5	Kruppel like factor 5
3,471	ENSG00000067082	KLF6	Kruppel like factor 6
1,324	ENSG00000118263	KLF7	Kruppel like factor 7
1,037	ENSG00000174010	KLHL15	kelch like family member 15
1,351	ENSG00000187961	KLHL17	kelch like family member 17
3,227	ENSG00000162413	KLHL21	kelch like family member 21
1,503	ENSG00000119771	KLHL29	kelch like family member 29
1,493	ENSG00000129451	KLK10	kallikrein related peptidase 10
1,814	ENSG00000167757	KLK11	kallikrein related peptidase 11
1,082	ENSG00000005483	KMT2E	lysine methyltransferase 2E
1,023	ENSG00000171346	KRT15	keratin 15
4,214	ENSG00000128422	KRT17	keratin 17
1,259	ENSG00000111057	KRT18	keratin 18
2,836	ENSG00000171345	KRT19	keratin 19
1,097	ENSG00000185479	KRT6B	keratin 6B
2,644	ENSG00000135480	KRT7	keratin 7
1,573	ENSG00000170421	KRT8	keratin 8
2,57	ENSG00000167767	KRT80	keratin 80
1,931	ENSG00000205426	KRT81	keratin 81
1,802	ENSG00000170442	KRT86	keratin 86
1,158	ENSG00000187026	KRTAP21-2	keratin associated protein 21-2
1,066	ENSG00000157992	KRTCAP3	keratinocyte associated protein 3
2,084	ENSG00000159166	LAD1	ladinin 1
1,436	ENSG00000101680	LAMA1	laminin subunit alpha 1
1,326	ENSG00000053747	LAMA3	laminin subunit alpha 3
2,175	ENSG00000130702	LAMA5	laminin subunit alpha 5
1,504	ENSG00000172037	LAMB2	laminin subunit beta 2
2,997	ENSG00000196878	LAMB3	laminin subunit beta 3
2,117	ENSG00000135862	LAMC1	laminin subunit gamma 1
3,697	ENSG00000058085	LAMC2	laminin subunit gamma 2
1,573	ENSG00000165905	LARGE2	LARGE xylosyl- and glucuronyltransferase 2
1,961	ENSG00000166173	LARP6	La ribonucleoprotein domain family member 6
1,186	ENSG00000131023	LATS1	large tumor suppressor kinase 1
1,061	ENSG00000204381	LAYN	layilin
1,433	ENSG00000135338	LCA5	LCA5, lebercilin
1,622	ENSG00000148346	LCN2	lipocalin 2
1,047	ENSG00000243709	LEFTY1	left-right determination factor 1
1,085	ENSG00000174106	LEMD3	LEM domain containing 3
1,154	ENSG00000100079	LGALS2	galectin 2
1,342	ENSG00000145685	LHFPL2	lipoma HMGIC fusion partner-like 2
3,96	ENSG00000128342	LIF	LIF, interleukin 6 family cytokine
1,055	ENSG00000182541	LIMK2	LIM domain kinase 2
1,8	ENSG00000225880	LINC00115	long intergenic non-protein coding RNA 115
1,126	ENSG00000231028	LINC00271	long intergenic non-protein coding RNA 271
1,14	ENSG00000233237	LINC00472	long intergenic non-protein coding RNA 472
1,405	ENSG00000258169	LINC00485	long intergenic non-protein coding RNA 485
1,428	ENSG00000163898	LIPH	lipase H
1,797	ENSG00000073350	LLGL2	LLGL2, scribble cell polarity complex component
1,173	ENSG00000154359	LONRF1	LON peptidase N-terminal domain and ring finger 1
1,669	ENSG00000064547	LPAR2	lysophosphatidic acid receptor 2
1,837	ENSG00000139263	LRIG3	leucine rich repeats and immunoglobulin like domains 3
1,369	ENSG00000137269	LRRC1	leucine rich repeat containing 1
1,426	ENSG00000148814	LRRC27	leucine rich repeat containing 27
1,22	ENSG00000159708	LRRC36	leucine rich repeat containing 36
1,403	ENSG00000178026	LRRC75B	leucine rich repeat containing 75B
1,438	ENSG00000171017	LRRC8E	leucine rich repeat containing 8 family member E
2,434	ENSG00000175928	LRRN1	leucine rich repeat neuronal 1
1,692	ENSG00000125872	LRRN4	leucine rich repeat neuronal 4
1,296	ENSG00000007392	LUC7L	LUC7 like
1,241	ENSG00000153714	LURAP1L	leucine rich adaptor protein 1 like
1,69	ENSG00000150556	LYPD6B	LY6/PLAUR domain containing 6B
1,414	ENSG00000181541	MAB21L2	mab-21 like 2
1,089	ENSG00000173212	MAB21L3	mab-21 like 3
2,691	ENSG00000183742	MACC1	MACC1, MET transcriptional regulator

3,138	ENSG00000185022	MAFF	MAF bZIP transcription factor F
2,165	ENSG00000197063	MAFG	MAF bZIP transcription factor G
1,098	ENSG00000265688	MAFG-AS1	MAFG antisense RNA 1 (head to head)
1,949	ENSG00000198517	MAFK	MAF bZIP transcription factor K
1,362	ENSG00000187243	MAGED4/MAGED4B	MAGE family member D4B
1,559	ENSG00000151276	MAGI1	membrane associated guanylate kinase, WW and PDZ domain containing 1
1,996	ENSG00000251562	MALAT1	metastasis associated lung adenocarcinoma transcript 1 (non-protein coding)
1,278	ENSG00000184384	MAML2	mastermind like transcriptional coactivator 2
1,327	ENSG00000197769	MAP1LC3C	microtubule associated protein 1 light chain 3 gamma
1,492	ENSG00000130479	MAP1S	microtubule associated protein 1S
1,498	ENSG00000078018	MAP2	microtubule associated protein 2
1,157	ENSG00000034152	MAP2K3	mitogen-activated protein kinase kinase 3
1,524	ENSG00000095015	MAP3K1	mitogen-activated protein kinase kinase kinase 1
1,795	ENSG00000006062	MAP3K14	mitogen-activated protein kinase kinase kinase 14
2,519	ENSG00000107968	MAP3K8	mitogen-activated protein kinase kinase kinase 8
1,738	ENSG00000006432	MAP3K9	mitogen-activated protein kinase kinase kinase 9
1,027	ENSG00000047849	MAP4	microtubule associated protein 4
1,35	ENSG00000116871	MAP7D1	MAP7 domain containing 1
1,197	ENSG00000156711	MAPK13	mitogen-activated protein kinase 13
1,688	ENSG00000138834	MAPK8IP3	mitogen-activated protein kinase 8 interacting protein 3
1,198	ENSG00000173926	MARCH3	membrane associated ring-CH-type finger 3
1,385	ENSG00000145495	MARCH6	membrane associated ring-CH-type finger 6
1,464	ENSG00000175130	MARCKSL1	MARCKS like 1
1,133	ENSG00000072518	MARK2	microtubule affinity regulating kinase 2
1,031	ENSG00000140832	MARVELD3	MARVEL domain containing 3
1,136	ENSG00000164430	MB21D1	Mab-21 domain containing 1
1,057	ENSG00000166987	MBD6	methyl-CpG binding domain protein 6
1,11	ENSG00000172197	MBOAT1	membrane bound O-acyltransferase domain containing 1
2,004	ENSG00000177669	MBOAT4	membrane bound O-acyltransferase domain containing 4
1,199	ENSG00000258839	MC1R	melanocortin 1 receptor
1,568	ENSG00000076706	MCAM	melanoma cell adhesion molecule
1,158	ENSG00000126217	MCF2L	MCF.2 cell line derived transforming sequence like
2,387	ENSG00000143384	MCL1	MCL1, BCL2 family apoptosis regulator
1,202	ENSG00000055732	MCOLN3	mucolipin 3
1,081	ENSG00000156026	MCU	mitochondrial calcium uniporter
1,347	ENSG00000110492	MDK	midkine (neurite growth-promoting factor 2)
1,061	ENSG00000198625	MDM4	MDM4, p53 regulator
1,3	ENSG00000151376	ME3	malic enzyme 3
1,117	ENSG00000099917	MED15	mediator complex subunit 15
1,808	ENSG00000163975	MELTF	melanotransferrin
1,145	ENSG00000140406	MESDC1	mesoderm development candidate 1
1,068	ENSG00000166823	MESP1	mesoderm posterior bHLH transcription factor 1
1,178	ENSG00000105976	MET	MET proto-oncogene, receptor tyrosine kinase
2,038	ENSG00000214756	METTL12	methyltransferase like 12
1,04	ENSG00000144401	METTL21A	methyltransferase like 21A
1,495	ENSG00000176624	MEX3C	mex-3 RNA binding family member C
1,01	ENSG00000147324	MFHAS1	malignant fibrous histiocytoma amplified sequence 1
1,236	ENSG00000151690	MFSD6	major facilitator superfamily domain containing 6
1,086	ENSG00000185156	MFSD6L	major facilitator superfamily domain containing 6 like
1,789	ENSG00000243156	MICAL3	microtubule associated monooxygenase, calponin and LIM domain containing 3
1,35	ENSG00000167470	MIDN	midnolin
1,28	ENSG00000266192	mir-1260b	microRNA 1260b
4,592	ENSG00000207614	mir-193	microRNA 193a
6,182	ENSG00000207980	mir-23	microRNA 23a
7,78	ENSG00000207617	mir-3074	microRNA 3074
6,127	ENSG00000263628	mir-3155	microRNA 3155a
6,805	ENSG00000199023	mir-339	microRNA 339
4,279	ENSG00000264462	mir-3648	microRNA 3648-1
6,201	ENSG00000199032	mir-425	microRNA 425
3,211	ENSG00000207589	mir-506	microRNA 508
4,469	ENSG00000207946	mir-515	microRNA 520c
2,186	ENSG00000207650	mir-548	microRNA 579
5,715	ENSG00000207716	mir-572	microRNA 572
6,782	ENSG00000207561	mir-635	microRNA 635
3,98	ENSG00000207703	mir-7	microRNA 7-1
1,054	ENSG00000215417	MIR17HG	miR-17-92a-1 cluster host gene
7,535	ENSG00000266533	MIR3619	microRNA 3619
3,905	ENSG00000265558	MIR3918	microRNA 3918
3,744	ENSG00000265442	MIR3941	microRNA 3941
5,905	ENSG00000264171	MIR4305	microRNA 4305
4,259	ENSG00000263583	MIR4522	microRNA 4522
6,184	ENSG00000263834	MIR4635	microRNA 4635
6,799	ENSG00000263575	MIR4665	microRNA 4665
2,27	ENSG00000099812	MISP	mitotic spindle positioning
1,982	ENSG00000178053	MLF1	myeloid leukemia factor 1
1,33	ENSG00000171843	MLLT3	MLLT3, super elongation complex subunit
2,395	ENSG00000196611	MMP1	matrix metallopeptidase 1
1,701	ENSG00000166670	MMP10	matrix metallopeptidase 10
1,057	ENSG00000157227	MMP14	matrix metallopeptidase 14
1,259	ENSG00000102996	MMP15	matrix metallopeptidase 15
1,368	ENSG00000123342	MMP19	matrix metallopeptidase 19

1,195	ENSG00000125966	MMP24	matrix metallopeptidase 24
2,578	ENSG00000137673	MMP7	matrix metallopeptidase 7
1,124	ENSG00000130675	MNX1	motor neuron and pancreas homeobox 1
1,446	ENSG00000120162	MOB3B	MOB kinase activator 3B
1,216	ENSG00000161647	MPP3	membrane palmitoylated protein 3
1,061	ENSG00000133030	MPRIP	myosin phosphatase Rho interacting protein
1,382	ENSG00000160588	MPZL3	myelin protein zero like 3
1,037	ENSG00000174579	MSL2	male-specific lethal 2 homolog (Drosophila)
1,649	ENSG00000163132	MSX1	msh homeobox 1
1,109	ENSG00000120149	MSX2	msh homeobox 2
1,797	ENSG00000256618	MTRNR2L1	MT-RNR2-like 1
1,654	ENSG00000256045	MTRNR2L10	MT-RNR2-like 10
1,672	ENSG00000271043	MTRNR2L2	MT-RNR2-like 2
1,627	ENSG00000255823	MTRNR2L8	MT-RNR2-like 8
1,552	ENSG00000185499	MUC1	mucin 1, cell surface associated
1,553	ENSG00000176945	MUC20	mucin 20, cell surface associated
1,17	ENSG00000117983	MUC5B	mucin 5B, oligomeric mucus/gel-forming
3,706	ENSG00000184956	MUC6	mucin 6, oligomeric mucus/gel-forming
1,638	ENSG00000157502	MUM1L1	MUM1 like 1
2,732	ENSG00000059728	MXD1	MAX dimerization protein 1
2,2	ENSG00000179820	MYADM	myeloid associated differentiation marker
1,069	ENSG00000132382	MYBBP1A	MYB binding protein 1a
2,293	ENSG00000136997	MYC	MYC proto-oncogene, bHLH transcription factor
1,443	ENSG00000104177	MYEF2	myelin expression factor 2
1,423	ENSG00000105357	MYH14	myosin heavy chain 14
1,785	ENSG00000100345	MYH9	myosin heavy chain 9
2,022	ENSG00000007944	MYLIP	myosin regulatory light chain interacting protein
1,981	ENSG00000145555	MYO10	myosin X
1,007	ENSG00000197879	MYO1C	myosin IC
1,064	ENSG00000157483	MYO1E	myosin IE
1,324	ENSG00000095777	MYO3A	myosin IIIA
1,336	ENSG00000128833	MYO5C	myosin VC
1,155	ENSG00000196586	MYO6	myosin VI
1,196	ENSG00000138119	MYOF	myoferlin
1,202	ENSG00000263155	MYZAP	myocardial zonula adherens protein
1,396	ENSG00000099326	MZF1	myeloid zinc finger 1
1,46	ENSG00000172766	NAA16	N(alpha)-acetyltransferase 16, NatA auxiliary subunit
1,088	ENSG00000110583	NAA40	N(alpha)-acetyltransferase 40, NatD catalytic subunit
1,472	ENSG00000177694	NAALADL2	N-acetylated alpha-linked acidic dipeptidase like 2
1,111	ENSG00000172915	NBEA	neurobeachin
1,397	ENSG00000160796	NBEAL2	neurobeachin like 2
3,119	ENSG00000144959	NCEH1	neutral cholesterol ester hydrolase 1
1,98	ENSG00000071051	NCK2	NCK adaptor protein 2
2,972	ENSG00000111912	NCOA7	nuclear receptor coactivator 7
1,118	ENSG00000141027	NCOR1	nuclear receptor corepressor 1
1,536	ENSG00000245532	NEAT1	nuclear paraspeckle assembly transcript 1 (non-protein coding)
1,306	ENSG00000130202	NECTIN2	nectin cell adhesion molecule 2
2,153	ENSG00000143217	NECTIN4	nectin cell adhesion molecule 4
1,516	ENSG00000049759	NEDD4L	neural precursor cell expressed, developmentally down-regulated 4-like, E3 ubiquitin protein ligase
2,514	ENSG00000111859	NEDD9	neural precursor cell expressed, developmentally down-regulated 9
1,026	ENSG00000114670	NEK11	NIMA related kinase 11
1,029	ENSG00000160602	NEK8	NIMA related kinase 8
1,238	ENSG00000067141	NEO1	neogenin 1
1,215	ENSG00000173848	NET1	neuroepithelial cell transforming 1
2,183	ENSG00000163121	NEURL3	neuralized E3 ubiquitin protein ligase 3
1,374	ENSG00000102908	NFAT5	nuclear factor of activated T-cells 5
2,113	ENSG00000131196	NFATC1	nuclear factor of activated T-cells 1
1,915	ENSG00000050344	NFE2L3	nuclear factor, erythroid 2 like 3
1,484	ENSG00000165030	NFIL3	nuclear factor, interleukin 3 regulated
1,517	ENSG00000109320	NFKB1	nuclear factor kappa B subunit 1
1,655	ENSG00000077150	NFKB2	nuclear factor kappa B subunit 2
1,589	ENSG00000167604	NFKBID	NFKB inhibitor delta
2,028	ENSG00000146232	NFKBIE	NFKB inhibitor epsilon
4,124	ENSG00000144802	NFKBIZ	NFKB inhibitor zeta
1,387	ENSG00000001167	NFYA	nuclear transcription factor Y subunit alpha
1,089	ENSG00000101004	NINL	ninein like
1,114	ENSG00000167034	NKX3-1	NK3 homeobox 1
1,116	ENSG00000165246	NLGN4Y	neuroligin 4, Y-linked
1,597	ENSG00000197696	NMB	neuromedin B
2,061	ENSG00000112981	NME5	NME/NM23 family member 5
1,374	ENSG00000151014	NOCT	nocturnin
1,501	ENSG00000188747	NOXA1	NADPH oxidase activator 1
1,161	ENSG00000170485	NPAS2	neuronal PAS domain protein 2
2,111	ENSG00000141458	NPC1	NPC intracellular cholesterol transporter 1
1,154	ENSG00000015520	NPC1L1	NPC1 like intracellular cholesterol transporter 1
1,164	ENSG00000215440	NPEPL1	aminopeptidase-like 1
1,201	ENSG00000141279	NPEPPS	aminopeptidase puromycin sensitive
1,314	ENSG00000183426	NPIPA7 (includes others)	nuclear pore complex interacting protein family member A7
1,601	ENSG00000169246	NPIPB5 (includes others)	nuclear pore complex interacting protein family member B4
1,683	ENSG00000168743	NPNT	nephronectin
1,551	ENSG00000106236	NPTX2	neuronal pentraxin 2

1,606	ENSG00000131910	NR0B2	nuclear receptor subfamily 0 group B member 2
1,249	ENSG00000126368	NR1D1	nuclear receptor subfamily 1 group D member 1
1,974	ENSG00000123358	NR4A1	nuclear receptor subfamily 4 group A member 1
1,052	ENSG00000153234	NR4A2	nuclear receptor subfamily 4 group A member 2
1,869	ENSG00000198435	NRARP	NOTCH-regulated ankyrin repeat protein
1,08	ENSG00000091129	NRCAM	neuronal cell adhesion molecule
1,378	ENSG00000157168	NRG1	neuregulin 1
1,199	ENSG00000180530	NRIP1	nuclear receptor interacting protein 1
1,014	ENSG00000147548	NSD3	nuclear receptor binding SET domain protein 3
1,537	ENSG00000179299	NSUN7	NOP2/Sun RNA methyltransferase family member 7
1,044	ENSG00000074527	NTN4	netrin 4
4,455	ENSG00000163545	NUAK2	NUAK family kinase 2
1,207	ENSG00000108256	NUFIP2	NUFIP2, FMR1 interacting protein 2
1,511	ENSG00000137497	NUMA1	nuclear mitotic apparatus protein 1
1,372	ENSG00000137804	NUSAP1	nucleolar and spindle associated protein 1
1,334	ENSG00000162231	NXF1	nuclear RNA export factor 1
1,049	ENSG00000167693	NXN	nucleoredoxin
1,601	ENSG00000124006	OBSL1	obscurin like 1
1,142	ENSG00000122417	ODF2L	outer dense fiber of sperm tails 2 like
1,332	ENSG00000046651	OFD1	OFD1, centriole and centriolar satellite protein
1,627	ENSG00000173391	OLR1	oxidized low density lipoprotein receptor 1
2,071	ENSG00000169856	ONECUT1	one cut homeobox 1
1,813	ENSG00000079482	OPHN1	oligophrenin 1
1,264	ENSG00000177535	OR2B11	olfactory receptor family 2 subfamily B member 11
1,163	ENSG00000115947	ORC4	origin recognition complex subunit 4
1,644	ENSG00000165312	OTUD1	OTU deubiquitinase 1
1,084	ENSG00000189401	OTUD6A	OTU deubiquitinase 6A
2,736	ENSG00000172818	OVOL1	ovo like transcriptional repressor 1
2,629	ENSG00000180914	OXTR	oxytocin receptor
1,541	ENSG00000169860	P2RY1	purinergic receptor P2Y1
1,42	ENSG00000101104	PABPC1L	poly(A) binding protein cytoplasmic 1 like
1,223	ENSG00000100836	PABPN1	poly(A) binding protein nuclear 1
1,283	ENSG00000165912	PACSIN3	protein kinase C and casein kinase substrate in neurons 3
1,288	ENSG00000077264	PAK3	p21 (RAC1) activated kinase 3
1,634	ENSG00000187867	PALM3	paralemmin 3
1,185	ENSG00000135473	PAN2	PAN2 poly(A) specific ribonuclease subunit
1,026	ENSG00000152520	PAN3	PAN3 poly(A) specific ribonuclease subunit
1,697	ENSG00000100767	PAPLN	papilin, proteoglycan like sulfated glycoprotein
1,346	ENSG00000138801	PAPSS1	3'-phosphoadenosine 5'-phosphosulfate synthase 1
1,027	ENSG00000162073	PAQR4	progestin and adipoQ receptor family member 4
1,475	ENSG00000137819	PAQR5	progestin and adipoQ receptor family member 5
1,07	ENSG00000148498	PARD3	par-3 family cell polarity regulator
1,088	ENSG00000116117	PARD3B	par-3 family cell polarity regulator beta
2,084	ENSG00000124171	PARD6B	par-6 family cell polarity regulator beta
1,203	ENSG00000041880	PARP3	poly(ADP-ribose) polymerase family member 3
1,739	ENSG00000132849	PATJ	PATJ, crumbs cell polarity complex component
1,31	ENSG00000177425	PAWR	pro-apoptotic WT1 regulator
1,085	ENSG00000120327	PCDHB14	protocadherin beta 14
1,524	ENSG00000165494	PCF11	PCF11 cleavage and polyadenylation factor subunit
1,146	ENSG00000186472	PCLO	piccolo presynaptic cytomatrix protein
1,002	ENSG00000183036	PCP4	Purkinje cell protein 4
1,059	ENSG00000138735	PDE5A	phosphodiesterase 5A
1,205	ENSG00000160191	PDE9A	phosphodiesterase 9A
1,434	ENSG00000145431	PDGFC	platelet derived growth factor C
1,292	ENSG00000170962	PDGFD	platelet derived growth factor D
1,607	ENSG00000164951	PDP1	pyruvate dehyrogenase phosphatase catalytic subunit 1
1,008	ENSG00000196696	PDXDC2P-NPIPB14P	nuclear pore complex-interacting protein
1,5	ENSG00000172367	PDZD3	PDZ domain containing 3
1,121	ENSG00000165650	PDZD8	PDZ domain containing 8
1,071	ENSG00000162366	PDZK1IP1	PDZK1 interacting protein 1
1,572	ENSG00000242265	PEG10	paternally expressed 10
1,307	ENSG00000197329	PELI1	pellino E3 ubiquitin protein ligase 1
1,419	ENSG00000179094	PER1	period circadian clock 1
1,837	ENSG00000132326	PER2	period circadian clock 2
1,814	ENSG00000109272	PF4V1	platelet factor 4 variant 1
1,103	ENSG00000178921	PFAS	phosphoribosylformylglycinamidine synthase
2,111	ENSG00000170525	PFKFB3	6-phosphofructo-2-kinase/fructose-2,6-biphosphatase 3
1,442	ENSG00000067057	PFKP	phosphofructokinase, platelet
4,017	ENSG00000096088	PGC	progastricsin
2,25	ENSG00000142102	PGGHG	protein-glucosylgalactosylhydroxylysine glucosidase
1,078	ENSG00000165434	PGM2L1	phosphoglucomutase 2 like 1
1,104	ENSG00000204138	PHACTR4	phosphatase and actin regulator 4
1,62	ENSG00000111752	PHC1	polyhomeotic homolog 1
1,179	ENSG00000116273	PHF13	PHD finger protein 13
1,103	ENSG00000146247	PHIP	pleckstrin homology domain interacting protein
2,794	ENSG00000181649	PHLDA2	pleckstrin homology like domain family A member 2
1,012	ENSG00000183506	PI4KAP2	phosphatidylinositol 4-kinase alpha pseudogene 2
1,352	ENSG00000103335	PIEZO1	piezo type mechanosensitive ion channel component 1
1,671	ENSG00000162896	PIGR	polymeric immunoglobulin receptor
1,547	ENSG00000121716	PILRB	paired immunoglobin-like type 2 receptor beta
1,718	ENSG00000137193	PIM1	Pim-1 proto-oncogene, serine/threonine kinase

1,653	ENSG00000198355	PIM3	Pim-3 proto-oncogene, serine/threonine kinase
1,176	ENSG00000143398	PIP5K1A	phosphatidylinositol-4-phosphate 5-kinase type 1 alpha
1,089	ENSG00000241878	PISD	phosphatidylserine decarboxylase
1,992	ENSG00000134627	PIWIL4	piwi like RNA-mediated gene silencing 4
1,076	ENSG00000181191	PJA1	praja ring finger ubiquitin ligase 1
1,15	ENSG00000008710	PKD1	polycystin 1, transient receptor potential channel interacting
1,906	ENSG00000170927	PKHD1	PKHD1, fibrocystin/polyductin
1,535	ENSG00000184363	PKP3	plakophilin 3
1,621	ENSG00000243708	PLA2G4B	phospholipase A2 group IVB
1,281	ENSG00000184381	PLA2G6	phospholipase A2 group VI
1,007	ENSG00000126003	PLAGL2	PLAG1 like zinc finger 2
2,616	ENSG00000011422	PLAUR	plasminogen activator, urokinase receptor
1,36	ENSG00000161714	PLCD3	phospholipase C delta 3
1,233	ENSG00000129219	PLD2	phospholipase D2
1,009	ENSG00000143850	PLEKHA6	pleckstrin homology domain containing A6
1,432	ENSG00000166689	PLEKHA7	pleckstrin homology domain containing A7
2,222	ENSG00000021300	PLEKHB1	pleckstrin homology domain containing B1
1,372	ENSG00000126822	PLEKHG3	pleckstrin homology and RhoGEF domain containing G3
1,64	ENSG00000008323	PLEKHG6	pleckstrin homology and RhoGEF domain containing G6
1,145	ENSG00000214176	PLEKHM1P1	pleckstrin homology and RUN domain containing M1 pseudogene 1
2,923	ENSG00000145632	PLK2	polo like kinase 2
2,553	ENSG00000173846	PLK3	polo like kinase 3
1,086	ENSG00000102934	PLLP	plasmolipin
2,077	ENSG00000141934	PLPP2	phospholipid phosphatase 2
1,927	ENSG00000164050	PLXNB1	plexin B1
1,011	ENSG00000196576	PLXNB2	plexin B2
4,301	ENSG00000141682	PMAIP1	phorbol-12-myristate-13-acetate-induced protein 1
2,268	ENSG00000124225	PMEPA1	prostate transmembrane protein, androgen induced 1
1,332	ENSG00000132424	PNISR	PNN interacting serine and arginine rich protein
1,206	ENSG00000100941	PNN	pinin, desmosome associated protein
1,4	ENSG00000177666	PNPLA2	patatin like phospholipase domain containing 2
1,39	ENSG00000146278	PNRC1	proline rich nuclear receptor coactivator 1
1,508	ENSG00000124429	POF1B	premature ovarian failure, 1B
1,225	ENSG00000143442	POGZ	pogo transposable element derived with ZNF domain
1,655	ENSG00000181222	POLR2A	RNA polymerase II subunit A
1,099	ENSG00000009830	POMT2	protein O-mannosyltransferase 2
1,055	ENSG00000137709	POU2F3	POU class 2 homeobox 3
1,559	ENSG00000185668	POU3F1	POU class 3 homeobox 1
1,233	ENSG00000106536	POU6F2	POU class 6 homeobox 2
1,221	ENSG00000112033	PPARD	peroxisome proliferator activated receptor delta
2,242	ENSG00000118898	PPL	periplakin
1,059	ENSG00000170836	PPM1D	protein phosphatase, Mg2+/Mn2+ dependent 1D
1,593	ENSG00000088808	PPP1R13B	protein phosphatase 1 regulatory subunit 13B
2,1	ENSG00000104881	PPP1R13L	protein phosphatase 1 regulatory subunit 13 like
4,549	ENSG00000087074	PPP1R15A	protein phosphatase 1 regulatory subunit 15A
1,493	ENSG00000158615	PPP1R15B	protein phosphatase 1 regulatory subunit 15B
1,592	ENSG00000196422	PPP1R26	protein phosphatase 1 regulatory subunit 26
1,147	ENSG00000158528	PPP1R9A	protein phosphatase 1 regulatory subunit 9A
1,139	ENSG00000156475	PPP2R2B	protein phosphatase 2 regulatory subunit Bbeta
1,103	ENSG00000124224	PPP4R1L	protein phosphatase 4 regulatory subunit 1 like (pseudogene)
1,775	ENSG00000148840	PPRC1	peroxisome proliferator-activated receptor gamma, coactivator-related 1
1,148	ENSG00000196850	PPTC7	PTC7 protein phosphatase homolog
1,622	ENSG00000139174	PRICKLE1	prickle planar cell polarity protein 1
1,046	ENSG00000124593	PRICKLE4	prickle planar cell polarity protein 4
1,04	ENSG00000162409	PRKAA2	protein kinase AMP-activated catalytic subunit alpha 2
1,206	ENSG00000163932	PRKCD	protein kinase C delta
1,049	ENSG00000105287	PRKD2	protein kinase D2
2,385	ENSG00000007062	PROM1	prominin 1
1,413	ENSG00000110844	PRPF40B	pre-mRNA processing factor 40 homolog B
1,669	ENSG00000131188	PRR7	proline rich 7, synaptic
1,124	ENSG00000130723	PRRC2B	proline rich coiled-coil 2B
1,236	ENSG00000117523	PRRC2C	proline rich coiled-coil 2C
3,412	ENSG00000005001	PRSS22	protease, serine 22
1,771	ENSG00000206549	PRSS50	protease, serine 50
2,032	ENSG00000052344	PRSS8	protease, serine 8
1,399	ENSG00000125650	PSPN	persephin
1,086	ENSG00000011304	PTBP1	polypyrimidine tract binding protein 1
1,029	ENSG00000117569	PTBP2	polypyrimidine tract binding protein 2
1,442	ENSG00000244694	PTCHD4	patched domain containing 4
1,272	ENSG00000122420	PTGFR	prostaglandin F receptor
1,262	ENSG00000134247	PTGFRN	prostaglandin F2 receptor inhibitor
2,333	ENSG00000087494	PTHLH	parathyroid hormone like hormone
1,41	ENSG00000163629	PTPN13	protein tyrosine phosphatase, non-receptor type 13
1,031	ENSG00000152104	PTPN14	protein tyrosine phosphatase, non-receptor type 14
1,521	ENSG00000153707	PTPRD	protein tyrosine phosphatase, receptor type D
1,025	ENSG00000142949	PTPRF	protein tyrosine phosphatase, receptor type F
1,215	ENSG00000152894	PTPRK	protein tyrosine phosphatase, receptor type K
1,163	ENSG00000173482	PTPRM	protein tyrosine phosphatase, receptor type M
1,115	ENSG00000146676	PURB	purine rich element binding protein B
1,069	ENSG00000171813	PWWP2B	PWWP domain containing 2B
1,314	ENSG00000130508	PXDN	peroxidasin

1,547	ENSG00000100994	PYGB	glycogen phosphorylase B
2,867	ENSG00000156675	RAB11FIP1	RAB11 family interacting protein 1
1,234	ENSG00000135631	RAB11FIP5	RAB11 family interacting protein 5
1,167	ENSG00000146955	RAB19	RAB19, member RAS oncogene family
1,359	ENSG00000132698	RAB25	RAB25, member RAS oncogene family
1,109	ENSG00000100228	RAB36	RAB36, member RAS oncogene family
1,107	ENSG00000123892	RAB38	RAB38, member RAS oncogene family
1,738	ENSG00000127328	RAB3IP	RAB3A interacting protein
1,631	ENSG00000108557	RAI1	retinoic acid induced 1
1,493	ENSG00000131831	RAI2	retinoic acid induced 2
1,287	ENSG00000174373	RALGAPA1	Ral GTPase activating protein catalytic alpha subunit 1
1,442	ENSG00000160271	RALGDS	ral guanine nucleotide dissociation stimulator
1,249	ENSG00000136828	RALGPS1	Ral GEF with PH domain and SH3 binding motif 1
1,861	ENSG00000184672	RALYL	RALY RNA binding protein-like
1,355	ENSG00000100401	RANGAP1	Ran GTPase activating protein 1
1,327	ENSG00000076864	RAP1GAP	RAP1 GTPase activating protein
1,261	ENSG00000132359	RAP1GAP2	RAP1 GTPase activating protein 2
1,745	ENSG00000181467	RAP2B	RAP2B, member of RAS oncogene family
1,163	ENSG00000077092	RARB	retinoic acid receptor beta
1,513	ENSG00000172819	RARG	retinoic acid receptor gamma
1,332	ENSG00000111344	RASAL1	RAS protein activator like 1
1,495	ENSG00000108551	RASD1	ras related dexamethasone induced 1
2,469	ENSG00000165105	RASEF	RAS and EF-hand domain containing
1,578	ENSG00000107551	RASSF4	Ras association domain family member 4
1,16	ENSG00000169435	RASSF6	Ras association domain family member 6
1,492	ENSG00000123094	RASSF8	Ras association domain family member 8
3,392	ENSG00000198774	RASSF9	Ras association domain family member 9
1,12	ENSG00000161847	RAVER1	ribonucleoprotein, PTB binding 1
1,195	ENSG00000162437	RAVER2	ribonucleoprotein, PTB binding 2
1,791	ENSG00000122257	RBBP6	RB binding protein 6, ubiquitin ligase
1,046	ENSG00000101773	RBBP8	RB binding protein 8, endonuclease
1,097	ENSG00000239306	RBM14	RNA binding motif protein 14
1,554	ENSG00000162775	RBM15	RNA binding motif protein 15
1,091	ENSG00000122965	RBM19	RNA binding motif protein 19
1,053	ENSG00000119707	RBM25	RNA binding motif protein 25
1,549	ENSG00000132819	RBM38	RNA binding motif protein 38
1,283	ENSG00000131051	RBM39	RNA binding motif protein 39
1,27	ENSG00000004534	RBM6	RNA binding motif protein 6
1,112	ENSG00000076067	RBMS2	RNA binding motif single stranded interacting protein 2
1,043	ENSG00000157110	RBPMS	RNA binding protein with multiple splicing
1,198	ENSG00000135870	RC3H1	ring finger and CCCH-type domains 1
1,069	ENSG00000179051	RCC2	regulator of chromosome condensation 2
2,105	ENSG00000100918	REC8	REC8 meiotic recombination protein
1,936	ENSG00000162924	REL	REL proto-oncogene, NF-kB subunit
1,721	ENSG00000104856	RELB	RELB proto-oncogene, NF-kB subunit
1,525	ENSG00000139890	REM2	RRAD and GEM like GTPase 2
1,089	ENSG00000142599	RERE	arginine-glutamic acid dipeptide repeats
1,173	ENSG00000223638	RFPL4A/RFPL4AL1	ret finger protein like 4A
1,678	ENSG00000087903	RFX2	regulatory factor X2
1,783	ENSG00000205517	RGL3	ral guanine nucleotide dissociation stimulator like 3
1,599	ENSG00000169629	RGPD4 (includes others)	RANBP2-like and GRIP domain containing 5
1,148	ENSG00000091844	RGS17	regulator of G protein signaling 17
2,87	ENSG00000117152	RGS4	regulator of G protein signaling 4
1,711	ENSG00000007384	RHBDF1	rhomboid 5 homolog 1
1,634	ENSG00000129667	RHBDF2	rhomboid 5 homolog 2
1,676	ENSG00000158315	RHBDL2	rhomboid like 2
1,695	ENSG00000143878	RHOB	ras homolog family member B
1,009	ENSG00000140983	RHOT2	ras homolog family member T2
2,817	ENSG00000104140	RHOV	ras homolog family member V
1,746	ENSG00000131941	RHPN2	rhophilin Rho GTPase binding protein 2
1,155	ENSG00000166405	RIC3	RIC3 acetylcholine receptor chaperone
1,711	ENSG00000111785	RIC8B	RIC8 guanine nucleotide exchange factor B
1,963	ENSG00000104312	RIPK2	receptor interacting serine/threonine kinase 2
2,824	ENSG00000183421	RIPK4	receptor interacting serine/threonine kinase 4
1,548	ENSG00000117000	RLF	rearranged L-myc fusion
1,131	ENSG00000172602	RND1	Rho family GTPase 1
1,884	ENSG00000034677	RNF19A	ring finger protein 19A, RBR E3 ubiquitin protein ligase
1,719	ENSG00000116514	RNF19B	ring finger protein 19B
1,538	ENSG00000158286	RNF207	ring finger protein 207
1,113	ENSG00000173821	RNF213	ring finger protein 213
1,03	ENSG00000237330	RNF223	ring finger protein 223
1,322	ENSG00000108375	RNF43	ring finger protein 43
1,16	ENSG00000185946	RNPC3	RNA binding region (RNP1, RRM) containing 3
3,754	ENSG00000166592	RRAD	RRAD, Ras related glycolysis inhibitor and calcium channel regulator
1,47	ENSG00000052749	RRP12	ribosomal RNA processing 12 homolog
1,042	ENSG00000182841	RRP7BP	ribosomal RNA processing 7 homolog B, pseudogene
1,356	ENSG00000160188	RSPH1	radial spoke head 1 homolog
1,054	ENSG00000130363	RSPH3	radial spoke 3 homolog
1,259	ENSG00000111011	RSRC2	arginine and serine rich coiled-coil 2
1,906	ENSG00000117616	RSRP1	arginine and serine rich protein 1
1,151	ENSG00000185924	RTN4RL1	reticulon 4 receptor like 1

1,156	ENSG00000159216	RUNX1	runt related transcription factor 1
1,026	ENSG00000160753	RUSC1	RUN and SH3 domain containing 1
2,041	ENSG00000198853	RUSC2	RUN and SH3 domain containing 2
1,323	ENSG00000197747	S100A10	S100 calcium binding protein A10
1,258	ENSG00000163191	S100A11	S100 calcium binding protein A11
1,736	ENSG00000189334	S100A14	S100 calcium binding protein A14
1,255	ENSG00000188015	S100A3	S100 calcium binding protein A3
1,703	ENSG00000197956	S100A6	S100 calcium binding protein A6
1,34	ENSG00000160633	SAFB	scaffold attachment factor B
1,027	ENSG00000130254	SAFB2	scaffold attachment factor B2
1,169	ENSG00000141858	SAMD1	sterile alpha motif domain containing 1
1,182	ENSG00000020577	SAMD4A	sterile alpha motif domain containing 4A
1,118	ENSG00000205307	SAP25	Sin3A associated protein 25
1,05	ENSG00000100241	SBF1	SET binding factor 1
1,145	ENSG00000188322	SBK1	SH3 domain binding kinase 1
1,054	ENSG00000213079	SCAF8	SR-related CTD associated factor 8
1,058	ENSG00000140386	SCAPER	S-phase cyclin A associated protein in the ER
1,55	ENSG00000252010	SCARNA5	small Cajal body-specific RNA 5
4,481	ENSG00000251733	SCARNA8	small Cajal body-specific RNA 8
1,878	ENSG00000145284	SCD5	stearoyl-CoA desaturase 5
1,616	ENSG00000079689	SCGN	secretagogin, EF-hand calcium binding protein
1,528	ENSG00000111319	SCNN1A	sodium channel epithelial 1 alpha subunit
1,922	ENSG00000136193	SCRN1	secernin 1
2,981	ENSG00000080293	SCTR	secretin receptor
1,044	ENSG00000124145	SDC4	syndecan 4
1,416	ENSG00000185485	SDHAP1	succinate dehydrogenase complex flavoprotein subunit A pseudogene 1
1,146	ENSG00000215837	SDHAP2	succinate dehydrogenase complex flavoprotein subunit A pseudogene 2
1,088	ENSG00000214491	SEC14L6	SEC14 like lipid binding 6
1,519	ENSG00000091490	SEL1L3	SEL1L family member 3
4,02	ENSG00000007908	SELE	selectin E
1,658	ENSG00000170381	SEMA3E	semaphorin 3E
1,686	ENSG00000092421	SEMA6A	semaphorin 6A
1,332	ENSG00000137872	SEMA6D	semaphorin 6D
3,54	ENSG00000197632	SERPINB2	serpin family B member 2
1,261	ENSG00000170542	SERPINB9	serpin family B member 9
2,277	ENSG00000149257	SERPINH1	serpin family H member 1
1,862	ENSG00000197019	SERTAD1	SERTA domain containing 1
1,964	ENSG00000179833	SERTAD2	SERTA domain containing 2
1,049	ENSG00000167565	SERTAD3	SERTA domain containing 3
1,534	ENSG00000130766	SESN2	sestrin 2
1,746	ENSG00000174938	SEZ6L2	seizure related 6 homolog like 2
1,177	ENSG00000104897	SF3A2	splicing factor 3a subunit 2
1,13	ENSG00000198089	SFI1	SFI1 centrin binding protein
2,669	ENSG00000175793	SFN	stratifin
1,422	ENSG00000116560	SFPQ	splicing factor proline and glutamine rich
2,381	ENSG00000120057	SFRP5	secreted frizzled related protein 5
2,729	ENSG00000164023	SGMS2	sphingomyelin synthase 2
2,379	ENSG00000163082	SGPP2	sphingosine-1-phosphate phosphatase 2
1,846	ENSG00000125731	SH2D3A	SH2 domain containing 3A
1,07	ENSG00000130147	SH3BP4	SH3 domain binding protein 4
1,13	ENSG00000148341	SH3GLB2	SH3 domain containing GRB2 like, endophilin B2
1,888	ENSG00000154447	SH3RF1	SH3 domain containing ring finger 1
1,708	ENSG00000156463	SH3RF2	SH3 domain containing ring finger 2
1,19	ENSG00000035115	SH3YL1	SH3 and SYLF domain containing 1
1,152	ENSG00000107338	SHB	SH2 domain containing adaptor protein B
1,301	ENSG00000180730	SHISA2	shisa family member 2
2,283	ENSG00000237515	SHISA9	shisa family member 9
1,958	ENSG00000138771	SHROOM3	shroom family member 3
1,295	ENSG00000096717	SIRT1	sirtuin 1
1,649	ENSG00000100625	SIX4	SIX homeobox 4
2,885	ENSG00000064651	SLC12A2	solute carrier family 12 member 2
1,249	ENSG00000124067	SLC12A4	solute carrier family 12 member 4
1,206	ENSG00000113504	SLC12A7	solute carrier family 12 member 7
1,726	ENSG00000118596	SLC16A7	solute carrier family 16 member 7
1,116	ENSG00000146039	SLC17A4	solute carrier family 17 member 4
1,393	ENSG00000117479	SLC19A2	solute carrier family 19 member 2
1,006	ENSG00000144136	SLC20A1	solute carrier family 20 member 1
1,336	ENSG00000153291	SLC25A27	solute carrier family 25 member 27
1,899	ENSG00000197119	SLC25A29	solute carrier family 25 member 29
1,111	ENSG00000130304	SLC27A1	solute carrier family 27 member 1
1,796	ENSG00000197506	SLC28A3	solute carrier family 28 member 3
1,823	ENSG00000174669	SLC29A2	solute carrier family 29 member 2
1,196	ENSG00000117394	SLC2A1	solute carrier family 2 member 1
2,006	ENSG00000157765	SLC34A2	solute carrier family 34 member 2
1,085	ENSG00000100036	SLC35E4	solute carrier family 35 member E4
2,022	ENSG00000110660	SLC35F2	solute carrier family 35 member F2
1,197	ENSG00000183780	SLC35F3	solute carrier family 35 member F3
1,301	ENSG00000169507	SLC38A11	solute carrier family 38 member 11
1,667	ENSG00000168003	SLC3A2	solute carrier family 3 member 2
1,313	ENSG00000133065	SLC41A1	solute carrier family 41 member 1
1,438	ENSG00000143036	SLC44A3	solute carrier family 44 member 3

1,339	ENSG00000114923	SLC4A3	solute carrier family 4 member 3
2,101	ENSG00000080493	SLC4A4	solute carrier family 4 member 4
1,057	ENSG00000033867	SLC4A7	solute carrier family 4 member 7
1,66	ENSG00000100170	SLC5A1	solute carrier family 5 member 1
1,856	ENSG00000117834	SLC5A9	solute carrier family 5 member 9
2,472	ENSG00000174358	SLC6A19	solute carrier family 6 member 19
1,141	ENSG00000131389	SLC6A6	solute carrier family 6 member 6
2,202	ENSG00000139514	SLC7A1	solute carrier family 7 member 1
1,995	ENSG00000103257	SLC7A5	solute carrier family 7 member 5
2,005	ENSG00000260727	SLC7A5P1	solute carrier family 7 member 5 pseudogene 1
2,578	ENSG00000258186	SLC7A5P2	solute carrier family 7 member 5 pseudogene 2
1,426	ENSG00000090020	SLC9A1	solute carrier family 9 member A1
1,441	ENSG00000066230	SLC9A3	solute carrier family 9 member A3
1,055	ENSG00000180251	SLC9A4	solute carrier family 9 member A4
1,303	ENSG00000176463	SLCO3A1	solute carrier organic anion transporter family member 3A1
1,719	ENSG00000154760	SLFN13	schlafen family member 13
1,72	ENSG00000166949	SMAD3	SMAD family member 3
1,888	ENSG00000127616	SMARCA4	SWI/SNF related, matrix associated, actin dependent regulator of chromatin, subfamily a, member 4
3,569	ENSG00000172062	SMN1/SMN2	survival of motor neuron 1, telomeric
2,194	ENSG00000088826	SMOX	spermine oxidase
1,598	ENSG00000103056	SMPD3	sphingomyelin phosphodiesterase 3
1,679	ENSG00000130768	SMPDL3B	sphingomyelin phosphodiesterase acid like 3B
1,634	ENSG00000198742	SMURF1	SMAD specific E3 ubiquitin protein ligase 1
1,412	ENSG00000132639	SNAP25	synaptosome associated protein 25
1,062	ENSG00000197989	SNHG12	small nucleolar RNA host gene 12
1,123	ENSG00000234912	SNHG20	small nucleolar RNA host gene 20
1,569	ENSG00000163877	SNIP1	Smad nuclear interacting protein 1
5,44	ENSG00000206811	SNORA10	small nucleolar RNA, H/ACA box 10
7,427	ENSG00000206910	SNORA29	small nucleolar RNA, H/ACA box 29
3,876	ENSG00000206612	SNORA2A	small nucleolar RNA, H/ACA box 2A
4,564	ENSG00000207313	SNORA2B	small nucleolar RNA, H/ACA box 2B
5,76	ENSG00000199785	SNORA52	small nucleolar RNA, H/ACA box 52
1,216	ENSG00000212443	SNORA53	small nucleolar RNA, H/ACA box 53
3,867	ENSG00000206693	SNORA56	small nucleolar RNA, H/ACA box 56
2,131	ENSG00000199266	SNORA60	small nucleolar RNA, H/ACA box 60
2,57	ENSG00000207088	SNORA7B	small nucleolar RNA, H/ACA box 7B
4,278	ENSG00000206633	SNORA80B	small nucleolar RNA, H/ACA box 80B
3,059	ENSG00000239183	SNORA84	small nucleolar RNA, H/ACA box 84
3,521	ENSG00000238917	SNORD10	small nucleolar RNA, C/D box 10
8,337	ENSG00000199753	SNORD104	small nucleolar RNA, C/D box 104
2,796	ENSG00000207014	SNORD116-3	small nucleolar RNA, C/D box 116-3
5,891	ENSG00000239043	SNORD127	small nucleolar RNA, C/D box 127
2,638	ENSG00000207445	SNORD15B	small nucleolar RNA, C/D box 15B
5,671	ENSG00000200530	SNORD35B	small nucleolar RNA, C/D box 35B
5,249	ENSG00000202093	SNORD58C	small nucleolar RNA, C/D box 58C
6,486	ENSG00000209482	SNORD83A	small nucleolar RNA, C/D box 83A
1,719	ENSG00000212283	SNORD89	small nucleolar RNA, C/D box 89
2,627	ENSG00000221539	SNORD99	small nucleolar RNA, C/D box 99
1,253	ENSG00000144028	SNRNP200	small nuclear ribonucleoprotein U5 subunit 200
1,233	ENSG00000104852	SNRNP70	small nuclear ribonucleoprotein U1 subunit 70
1,254	ENSG00000159140	SON	SON DNA binding protein
1,12	ENSG00000198142	SOWAHC	sosondowah ankyrin repeat domain family member C
3,369	ENSG00000124766	SOX4	SRY-box 4
1,021	ENSG00000110693	SOX6	SRY-box 6
3,199	ENSG00000125398	SOX9	SRY-box 9
2,095	ENSG00000104450	SPAG1	sperm associated antigen 1
1,15	ENSG00000182957	SPATA13	spermatogenesis associated 13
1,136	ENSG00000152582	SPEF2	sperm flagellar 2
1,642	ENSG00000065526	SPEN	spen family transcriptional repressor
2,373	ENSG00000176170	SPHK1	sphingosine kinase 1
2,324	ENSG00000166145	SPINT1	serine peptidase inhibitor, Kunitz type 1
1,453	ENSG00000167642	SPINT2	serine peptidase inhibitor, Kunitz type 2
1,646	ENSG00000183018	SPNS2	sphingolipid transporter 2
1,615	ENSG00000118785	SPP1	secreted phosphoprotein 1
1,409	ENSG00000005206	SPPL2B	signal peptide peptidase like 2B
1,431	ENSG00000171621	SPSB1	splA/ryanodine receptor domain and SOCS box containing 1
1,199	ENSG00000197694	SPTAN1	spectrin alpha, non-erythrocytic 1
1,215	ENSG00000115306	SPTBN1	spectrin beta, non-erythrocytic 1
2,049	ENSG00000104549	SQLE	squalene epoxidase
1,048	ENSG00000197122	SRC	SRC proto-oncogene, non-receptor tyrosine kinase
1,214	ENSG00000198911	SREBF2	sterol regulatory element binding transcription factor 2
1,855	ENSG00000112658	SRF	serum response factor
1,365	ENSG00000196935	SRGAP1	SLIT-ROBO Rho GTPase activating protein 1
1,099	ENSG00000133226	SRRM1	serine and arginine repetitive matrix 1
1,709	ENSG00000167978	SRRM2	serine/arginine repetitive matrix 2
1,043	ENSG00000087087	SRRT	serrate, RNA effector molecule
1,27	ENSG00000154548	SRSF12	serine and arginine rich splicing factor 12
1,08	ENSG00000084112	SSH1	slingshot protein phosphatase 1
1,953	ENSG00000149418	ST14	suppression of tumorigenicity 14
1,123	ENSG00000166444	ST5	suppression of tumorigenicity 5
1,635	ENSG00000168439	STIP1	stress induced phosphoprotein 1

1,496	ENSG00000164543	STK17A	serine/threonine kinase 17a
2,118	ENSG00000130413	STK33	serine/threonine kinase 33
1,092	ENSG00000163482	STK36	serine/threonine kinase 36
1,097	ENSG00000198648	STK39	serine/threonine kinase 39
1,378	ENSG00000173320	STOX2	storkhead box 2
1,465	ENSG00000178750	STX19	syntaxin 19
1,747	ENSG00000166900	STX3	syntaxin 3
1,734	ENSG00000168952	STXBP6	syntaxin binding protein 6
1,5	ENSG00000064607	SUGP2	SURP and G-patch domain containing 2
1,894	ENSG00000198075	SULT1C4	sulfotransferase family 1C member 4
1,016	ENSG00000196235	SUPT5H	SPT5 homolog, DSIF elongation factor subunit
1,017	ENSG00000100647	SUSD6	sushi domain containing 6
1,23	ENSG00000197321	SVIL	supervillin
1,023	ENSG00000133789	SWAP70	SWAP switching B-cell complex subunit 70
1,762	ENSG00000205078	SYCE1L	synaptonemal complex central element protein 1 like
1,11	ENSG00000097096	SYDE2	synapse defective Rho GTPase homolog 2
1,02	ENSG00000054654	SYNE2	spectrin repeat containing nuclear envelope protein 2
1,251	ENSG00000100321	SYNGR1	synaptogyrin 1
1,42	ENSG00000078269	SYNJ2	synaptojanin 2
2,156	ENSG00000254806	SYS1-DBNDD2	SYS1-DBNDD2 readthrough (NMD candidate)
1,387	ENSG00000019505	SYT13	synaptotagmin 13
1,593	ENSG00000149043	SYT8	synaptotagmin 8
3,062	ENSG00000184292	TACSTD2	tumor associated calcium signal transducer 2
1,037	ENSG00000103168	TAF1C	TATA-box binding protein associated factor, RNA polymerase I subunit C
1,63	ENSG00000166012	TAF1D	TATA-box binding protein associated factor, RNA polymerase I subunit D
1,057	ENSG00000106290	TAF6	TATA-box binding protein associated factor 6
1,025	ENSG00000213977	TAX1BP3	Tax1 binding protein 3
1,598	ENSG00000065491	TBC1D22B	TBC1 domain family member 22B
1,672	ENSG00000204634	TBC1D8	TBC1 domain family member 8
1,032	ENSG00000165929	TC2N	tandem C2 domains, nuclear
1,072	ENSG00000113649	TCERG1	transcription elongation regulator 1
1,025	ENSG00000071564	TCF3	transcription factor 3
1,285	ENSG00000148737	TCF7L2	transcription factor 7 like 2
1,406	ENSG00000134827	TCN1	transcobalamin 1
1,097	ENSG00000187079	TEAD1	TEA domain transcription factor 1
1,669	ENSG00000092850	TEKT2	tektin 2
1,226	ENSG00000261408	TEN1-CDK3	TEN1-CDK3 readthrough (NMD candidate)
2,407	ENSG00000135269	TES	testin LIM domain protein
1,639	ENSG00000088992	TESC	tescalcin
1,439	ENSG00000175664	TEX26	testis expressed 26
1,817	ENSG00000087510	TFAP2C	transcription factor AP-2 gamma
1,089	ENSG00000112561	TFEB	transcription factor EB
1,965	ENSG00000160182	TFF1	trefoil factor 1
1,713	ENSG00000160181	TFF2	trefoil factor 2
2,834	ENSG00000105825	TFPI2	tissue factor pathway inhibitor 2
1,373	ENSG00000163235	TGFA	transforming growth factor alpha
3,105	ENSG00000092969	TGFB2	transforming growth factor beta 2
1,524	ENSG00000177426	TGIF1	TGFB induced factor homeobox 1
1,493	ENSG00000118707	TGIF2	TGFB induced factor homeobox 2
1,118	ENSG00000115970	THADA	THADA, armadillo repeat containing
1,931	ENSG00000137801	THBS1	thrombospondin 1
1,059	ENSG00000054118	THRAP3	thyroid hormone receptor associated protein 3
1,814	ENSG00000187720	THSD4	thrombospondin type 1 domain containing 4
1,627	ENSG00000146426	TIAM2	T-cell lymphoma invasion and metastasis 2
2,36	ENSG00000127666	TICAM1	toll like receptor adaptor molecule 1
1,038	ENSG00000142910	TINAGL1	tubulointerstitial nephritis antigen like 1
2,046	ENSG00000163659	TIPARP	TCDD inducible poly(ADP-ribose) polymerase
1,521	ENSG00000104067	TJP1	tight junction protein 1
1,873	ENSG00000119139	TJP2	tight junction protein 2
1,165	ENSG00000196781	TLE1	transducin like enhancer of split 1
1,336	ENSG00000065717	TLE2	transducin like enhancer of split 2
1,662	ENSG00000106829	TLE4	transducin like enhancer of split 4
1,095	ENSG00000187554	TLR5	toll like receptor 5
2,519	ENSG00000169908	TM4SF1	transmembrane 4 L six family member 1
1,498	ENSG00000103534	TMC5	transmembrane channel like 5
1,368	ENSG00000133069	TMCC2	transmembrane and coiled-coil domain family 2
1,801	ENSG00000006118	TMEM132A	transmembrane protein 132A
2,327	ENSG00000181264	TMEM136	transmembrane protein 136
2,375	ENSG00000249992	TMEM158	transmembrane protein 158 (gene/pseudogene)
1,135	ENSG00000152128	TMEM163	transmembrane protein 163
2,239	ENSG00000157111	TMEM171	transmembrane protein 171
1,458	ENSG00000164855	TMEM184A	transmembrane protein 184A
1,245	ENSG00000182796	TMEM198B	transmembrane protein 198B (pseudogene)
1,892	ENSG00000186329	TMEM212	transmembrane protein 212
1,135	ENSG00000172738	TMEM217	transmembrane protein 217
1,195	ENSG00000182107	TMEM30B	transmembrane protein 30B
1,573	ENSG00000171729	TMEM51	transmembrane protein 51
1,226	ENSG00000121900	TMEM54	transmembrane protein 54
1,481	ENSG00000196187	TMEM63A	transmembrane protein 63A
1,009	ENSG00000137103	TMEM8B	transmembrane protein 8B
1,567	ENSG00000137747	TMPRSS13	transmembrane protease, serine 13

1,337	ENSG00000184012	TMPRSS2	transmembrane protease, serine 2
2,294	ENSG00000160183	TMPRSS3	transmembrane protease, serine 3
2,362	ENSG00000041982	TNC	tenascin C
2,899	ENSG00000185215	TNFAIP2	TNF alpha induced protein 2
3,713	ENSG00000118503	TNFAIP3	TNF alpha induced protein 3
1,621	ENSG00000104689	TNFRSF10A	TNF receptor superfamily member 10a
1,999	ENSG00000120889	TNFRSF10B	TNF receptor superfamily member 10b
2,265	ENSG00000173530	TNFRSF10D	TNF receptor superfamily member 10d
1,97	ENSG00000006327	TNFRSF12A	TNF receptor superfamily member 12A
2,551	ENSG00000127863	TNFRSF19	TNF receptor superfamily member 19
2,088	ENSG00000146072	TNFRSF21	TNF receptor superfamily member 21
1,221	ENSG00000243509	TNFRSF6B	TNF receptor superfamily member 6b
1,759	ENSG00000248871	TNFSF12-TNFSF13	TNFSF12-TNFSF13 readthrough
3,102	ENSG00000125657	TNFSF9	TNF superfamily member 9
1,452	ENSG00000149115	TNKS1BP1	tankyrase 1 binding protein 1
1,417	ENSG00000183864	TOB2	transducer of ERBB2, 2
1,144	ENSG00000025772	TOMM34	translocase of outer mitochondrial membrane 34
1,016	ENSG00000198900	TOP1	topoisomerase (DNA) I
1,158	ENSG00000103460	TOX3	TOX high mobility group box family member 3
1,625	ENSG00000067369	TP53BP1	tumor protein p53 binding protein 1
3,191	ENSG00000143514	TP53BP2	tumor protein p53 binding protein 2
1,346	ENSG00000115129	TP53I3	tumor protein p53 inducible protein 3
2,516	ENSG00000146242	TPBG	trophoblast glycoprotein
1,649	ENSG00000171368	TPPP	tubulin polymerization promoting protein
2,594	ENSG00000159713	TPPP3	tubulin polymerization promoting protein family member 3
2,265	ENSG00000056558	TRAF1	TNF receptor associated factor 1
1,359	ENSG00000182606	TRAK1	trafficking kinesin protein 1
3,412	ENSG00000173334	TRIB1	tribbles pseudokinase 1
1,012	ENSG00000130726	TRIM28	tripartite motif containing 28
2,339	ENSG00000137699	TRIM29	tripartite motif containing 29
1,345	ENSG00000134253	TRIM45	tripartite motif containing 45
1,857	ENSG00000132481	TRIM47	tripartite motif containing 47
1,05	ENSG00000038382	TRIO	trio Rho guanine nucleotide exchange factor
1,498	ENSG00000125733	TRIP10	thyroid hormone receptor interactor 10
1,495	ENSG00000253368	TRNP1	TMF1-regulated nuclear protein 1
1,026	ENSG00000130529	TRPM4	transient receptor potential cation channel subfamily M member 4
1,099	ENSG00000165699	TSC1	tuberous sclerosis 1
1,086	ENSG00000103197	TSC2	tuberous sclerosis 2
2,906	ENSG00000196428	TSC22D2	TSC22 domain family member 2
1,607	ENSG00000099282	TSPAN15	tetraspanin 15
1,611	ENSG00000005379	TSPOAP1	TSPO associated protein 1
1,09	ENSG00000187189	TSPYL4	TSPY like 4
1,738	ENSG00000214021	TTLL3	tubulin tyrosine ligase like 3
1,381	ENSG00000135912	TTLL4	tubulin tyrosine ligase like 4
1,329	ENSG00000167553	TUBA1C	tubulin alpha 1c
1,129	ENSG00000137267	TUBB2A	tubulin beta 2A class IIa
1,396	ENSG00000137285	TUBB2B	tubulin beta 2B class IIb
1,161	ENSG00000104833	TUBB4A	tubulin beta 4A class IVa
1,367	ENSG00000188229	TUBB4B	tubulin beta 4B class IVb
1,311	ENSG00000128159	TUBGCP6	tubulin gamma complex associated protein 6
2,317	ENSG00000143367	TUFT1	tuftelin 1
1,082	ENSG00000253352	TUG1	taurine up-regulated 1 (non-protein coding)
1,023	ENSG00000137073	UBAP2	ubiquitin associated protein 2
1,005	ENSG00000143569	UBAP2L	ubiquitin associated protein 2 like
2,519	ENSG00000150991	UBC	ubiquitin C
1,187	ENSG00000186591	UBE2H	ubiquitin conjugating enzyme E2 H
1,809	ENSG00000108106	UBE2S	ubiquitin conjugating enzyme E2 S
1,01	ENSG00000177414	UBE2U	ubiquitin conjugating enzyme E2 U (putative)
1,292	ENSG00000158062	UBXN11	UBX domain protein 11
2,131	ENSG00000214049	UCA1	urothelial cancer associated 1 (non-protein coding)
1,319	ENSG00000148154	UGCG	UDP-glucose ceramide glucosyltransferase
2,083	ENSG00000174607	UGT8	UDP glycosyltransferase 8
1,013	ENSG00000124602	UNC5CL	unc-5 family C-terminal like
1,339	ENSG00000005007	UPF1	UPF1, RNA helicase and ATPase
1,016	ENSG00000114638	UPK1B	uroplakin 1B
1,055	ENSG00000106608	URGCP	upregulator of cell proliferation
1,988	ENSG00000006611	USH1C	USH1 protein network component harmonin
1,242	ENSG00000102226	USP11	ubiquitin specific peptidase 11
1,815	ENSG00000154914	USP43	ubiquitin specific peptidase 43
1,194	ENSG00000132952	USPL1	ubiquitin specific peptidase like 1
1,08	ENSG00000168140	VASN	vasorin
1,143	ENSG00000171724	VAT1L	vesicle amine transport 1 like
1,21	ENSG00000038427	VCAN	versican
2,057	ENSG00000112715	VEGFA	vascular endothelial growth factor A
1,574	ENSG00000197415	VEPH1	ventricular zone expressed PH domain containing 1
1,087	ENSG00000136451	VEZF1	vascular endothelial zinc finger 1
1,221	ENSG00000102243	VGLL1	vestigial like family member 1
1,346	ENSG00000206538	VGLL3	vestigial like family member 3
2,071	ENSG00000127831	VIL1	villin 1
1,029	ENSG00000136059	VILL	villin like
2,414	ENSG00000139722	VPS37B	VPS37B, ESCRT-I subunit

1,208	ENSG00000019102	VSIG2	V-set and immunoglobulin domain containing 2
1,366	ENSG00000132821	VSTM2L	V-set and transmembrane domain containing 2 like
1,831	ENSG00000134258	VTCN1	V-set domain containing T-cell activation inhibitor 1
2,958	ENSG00000199990	VTRNA1-1	vault RNA 1-1
7,969	ENSG00000202515	VTRNA1-3	vault RNA 1-3
1,034	ENSG00000062650	WAPL	WAPL cohesin release factor
2,239	ENSG00000227232	WASH7P	WAS protein family homolog 7 pseudogene
1,191	ENSG00000099290	WASHC2A/WASHC2C	WASH complex subunit 2C
1,07	ENSG00000157796	WDR19	WD repeat domain 19
1,537	ENSG00000184465	WDR27	WD repeat domain 27
1,206	ENSG00000085433	WDR47	WD repeat domain 47
1,88	ENSG00000178252	WDR6	WD repeat domain 6
1,222	ENSG00000152763	WDR78	WD repeat domain 78
1,634	ENSG00000161996	WDR90	WD repeat domain 90
1,194	ENSG00000105875	WDR91	WD repeat domain 91
3,19	ENSG00000166483	WEE1	WEE1 G2 checkpoint kinase
2,312	ENSG00000101443	WFDC2	WAP four-disulfide core domain 2
1,315	ENSG00000095397	WHRN	whirlin
2,225	ENSG00000165238	WNK2	WNK lysine deficient protein kinase 2
1,932	ENSG00000135925	WNT10A	Wnt family member 10A
1,167	ENSG00000188064	WNT7B	Wnt family member 7B
1,518	ENSG00000109046	WSB1	WD repeat and SOCS box containing 1
1,652	ENSG00000113645	WWC1	WW and C2 domain containing 1
1,249	ENSG00000198373	WWP2	WW domain containing E3 ubiquitin protein ligase 2
1,445	ENSG00000018408	WWTR1	WW domain containing transcription regulator 1
1,967	ENSG00000180667	YOD1	YOD1 deubiquitinase
1,355	ENSG00000175155	YPEL2	yippee like 2
1,658	ENSG00000205189	ZBTB10	zinc finger and BTB domain containing 10
1,518	ENSG00000109906	ZBTB16	zinc finger and BTB domain containing 16
1,809	ENSG00000181472	ZBTB2	zinc finger and BTB domain containing 2
2,039	ENSG00000181722	ZBTB20	zinc finger and BTB domain containing 20
1,355	ENSG00000177125	ZBTB34	zinc finger and BTB domain containing 34
1,114	ENSG00000184677	ZBTB40	zinc finger and BTB domain containing 40
2,362	ENSG00000169155	ZBTB43	zinc finger and BTB domain containing 43
1,268	ENSG00000178951	ZBTB7A	zinc finger and BTB domain containing 7A
4,604	ENSG00000163874	ZC3H12A	zinc finger CCCH-type containing 12A
1,272	ENSG00000105939	ZC3HAV1	zinc finger CCCH-type containing, antiviral 1
1,195	ENSG00000033030	ZCCHC8	zinc finger CCHC-type containing 8
1,105	ENSG00000159714	ZDHHC1	zinc finger DHHC-type containing 1
2,023	ENSG00000178381	ZFAND2A	zinc finger AN1-type containing 2A
1,408	ENSG00000133858	ZFC3H1	zinc finger C3H1-type containing
2,784	ENSG00000128016	ZFP36	ZFP36 ring finger protein
1,277	ENSG00000185650	ZFP36L1	ZFP36 ring finger protein like 1
2,543	ENSG00000162078	ZG16B	zymogen granule protein 16B
1,056	ENSG00000178764	ZHX2	zinc fingers and homeoboxes 2
1,475	ENSG00000108175	ZMIZ1	zinc finger MIZ-type containing 1
1,607	ENSG00000122515	ZMIZ2	zinc finger MIZ-type containing 2
1,151	ENSG00000115568	ZNF142	zinc finger protein 142
3,271	ENSG00000197279	ZNF165	zinc finger protein 165
1,758	ENSG00000171940	ZNF217	zinc finger protein 217
1,361	ENSG00000165804	ZNF219	zinc finger protein 219
1,079	ENSG00000159917	ZNF235	zinc finger protein 235
1,153	ENSG00000130856	ZNF236	zinc finger protein 236
1,307	ENSG00000152454	ZNF256	zinc finger protein 256
1,275	ENSG00000185947	ZNF267	zinc finger protein 267
1,307	ENSG00000162702	ZNF281	zinc finger protein 281
2,157	ENSG00000170684	ZNF296	zinc finger protein 296
1,023	ENSG00000130803	ZNF317	zinc finger protein 317
1,279	ENSG00000198026	ZNF335	zinc finger protein 335
1,239	ENSG00000197647	ZNF433	zinc finger protein 433
1,029	ENSG00000083838	ZNF446	zinc finger protein 446
1,679	ENSG00000105732	ZNF574	zinc finger protein 574
1,251	ENSG00000166716	ZNF592	zinc finger protein 592
1,352	ENSG00000168916	ZNF608	zinc finger protein 608
1,593	ENSG00000171163	ZNF692	zinc finger protein 692
1,531	ENSG00000147180	ZNF711	zinc finger protein 711
1,039	ENSG00000221874	ZNF816-ZNF321P	ZNF816-ZNF321P readthrough
1,375	ENSG00000257446	ZNF878	zinc finger protein 878
1,293	ENSG00000170044	ZPLD1	zona pellucida like domain containing 1
1,263	ENSG00000229956	ZRANB2-AS2	ZRANB2 antisense RNA 2 (head to head)
1,46	ENSG00000219891	ZSCAN12P1	zinc finger and SCAN domain containing 12 pseudogene 1
1,044	ENSG00000121413	ZSCAN18	zinc finger and SCAN domain containing 18
2,171	ENSG00000132003	ZSWIM4	zinc finger SWIM-type containing 4
1,534	ENSG00000130449	ZSWIM6	zinc finger SWIM-type containing 6
1,487	ENSG00000198455	ZXDB	zinc finger, X-linked, duplicated B
1,092	ENSG00000159840	ZYX	zyxin
